# A safety screening platform for individualized cardiotoxicity assessment

**DOI:** 10.1016/j.isci.2024.109139

**Published:** 2024-02-06

**Authors:** Verena Schwach, Rolf H. Slaats, Carla Cofiño-Fabres, Simone A. ten Den, José M. Rivera-Arbeláez, Maureen Dannenberg, Chiara van Boheemen, Marcelo C. Ribeiro, Sabina Y. van der Zanden, Edgar E. Nollet, Jolanda van der Velden, Jacques Neefjes, Lu Cao, Robert Passier

**Affiliations:** 1 Applied Stem Cell Technologies, TechMed Centre, University of Twente, Drienerlolaan 5, 7500 AE Enschede, the Netherlands; 2 BIOS Lab-on-a-Chip Group, MESA+ Institute for Nanotechnology, Technical Medical Centre, Max Planck Center for Complex Fluid Dynamics, University of Twente, Enschede, the Netherlands; 3 River BioMedics, Enschede, the Netherlands; 4 Department of Cell and Chemical Biology, ONCODE Institute, Leiden University Medical Center, 2333 ZC Leiden, the Netherlands; 5 Department of Physiology, Amsterdam UMC, Vrije Universiteit Amsterdam, Amsterdam Cardiovascular Sciences, Amsterdam, the Netherlands; 6 Leiden Institute of Advanced Computer Science (LIACS), Universiteit Leiden, Niels Bohrweg 1, 2333 CA Leiden, the Netherlands; 7 Department of Anatomy and Embryology, Leiden University Medical Centre, PO Box 9600, 2300 RC Leiden, the Netherlands

**Keywords:** Bioinformatics, Biological sciences, Natural sciences, Pharmacoinformatics

## Abstract

Cardiotoxicity remains a major cause of drug withdrawal, partially due to lacking predictability of animal models. Additionally, risk of cardiotoxicity following treatment of cancer patients is treatment limiting. It is unclear which patients will develop heart failure following therapy. Human pluripotent stem cell (hPSC)-derived cardiomyocytes present an unlimited cell source and may offer individualized solutions to this problem. We developed a platform to predict molecular and functional aspects of cardiotoxicity. Our platform can discriminate between the different cardiotoxic mechanisms of existing and novel anthracyclines Doxorubicin, Aclarubicin, and Amrubicin. Doxorubicin and Aclarubicin unlike Amrubicin substantially affected the transcriptome, mitochondrial membrane integrity, contractile force and transcription factor availability. Cardiomyocytes recovered fully within two or three weeks, corresponding to the intermittent clinical treatment regimen. Our system permits the study of hPSC-cardiomyocyte recovery and the effects of accumulated dose after multiple dosing, allowing individualized cardiotoxicity evaluation, which effects millions of cancer patients treated annually.

## Highlights

• We developed an *in-vitro* platform for cardiotoxicity testing utilizing hPSC-cardiomyocytes
• We tested a clinically relevant dosing regimen with tissue recovery between treatments
• This platform can discriminate between different cardiotoxic mechanisms

## Graphical abstract

**Figure:**
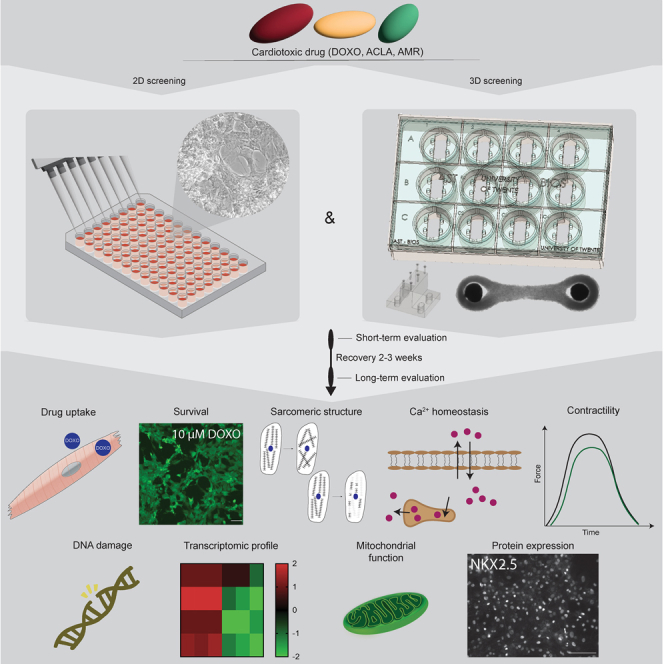

## Introduction

Anthracyclines are effective anti-cancer drugs, but their use is limited by dose accumulated cardiotoxicity. For this reason, patients are only receiving 4–6 courses of treatment. A major problem is that patients differ in their sensitivity implying that some patients can be treated longer. A possible explanation is that safety assessment of novel drugs is primarily evaluated in different animal models, which may not be sufficiently predictive for drug-induced cardiotoxicity in human patients. Human pluripotent stem cell (hPSC)-derived cardiomyocytes provide a reliable source of human cardiomyocytes and have already proven valuable for pharmacology screening of cardiotoxicity,^1^ however, any single hPSC-cardiomyocyte based assay cannot recapitulate all aspects of toxicity found *in vivo.* In order to formulate a weighted assessment of a compound’s toxicity without the use of animal testing, it is important to explore key mechanisms of compound-induced cardiotoxicity in a variety of human-based *in vitro* models that collectively approximate *in vivo* complexity.

The major anti-cancer drug is Doxorubicin (DOXO) which has multifaceted (cardio-)toxic side effects. Nevertheless, treatment with this “Red Devil” (based on its bright red color) has been associated with a number of toxic side effects, among which DOXO-induced cardiotoxicity is a prevalent and life-threatening complication. The exact mechanism of DOXO-induced toxicity to human cardiomyocytes remains unclear, but several mechanisms have been suggested.^1^ DOXO toxicity in proliferating cells is mostly mediated through interaction with Topoisomerase IIα, where it induces DNA damage and thus inhibits cell division. In adult cardiomyocytes, which do not proliferate, toxicity is thought to be induced via a combination of DOXO-mediated disturbances of cell processes. It was recently suggested that cardiotoxicity is triggered through 1) the inhibition of topoisomerase IIβ, leading to double-strand breaks (DSBs) in the DNA, 2) eviction of histones, causing chromatin damage, 3) mitochondrial dysfunction, triggering elevated release of reactive oxygen species (ROS) and reactive nitrogen species (NOS), 4) perturbed calcium ion (Ca^2+^) handling leading to Ca^2+^ overload and 5) the direct induction of apoptosis.^1^ All these effects may affect the contractile function of cardiomyocytes, and as such, DOXO-induced cardiotoxicity is mostly diagnosed by reductions in left ventricular ejection fraction (LVEF) in patients, indicating a loss of cardiac function.

Despite these known cardiotoxic elements, DOXO remains widely used due to its efficacy in combating neoplasms. For many tumors, there is no treatment alternative. Finding a solution for the DOXO-induced toxicity may therefore be of great value to oncology. Despite the development of several approaches to reduce DOXO-induced cardiotoxicity, through development of analogues, DOXO-encapsulation or co-treatment with protective compounds, DOXO-induced cardiotoxicity remains a substantial limitation of its use in the clinic.^2^ This may be due to the lack of an appropriate model to investigate the mechanisms of DOXO toxicity which only recently involved the use of hPSC-derived cardiomyocytes.

Aclarubicin (ACLA) and Amrubicin (AMR) represent two out of many chemical analogues of DOXO that have been developed over the years, in an attempt to find a molecular cousin to DOXO that is equally or more potent in combating malignancies, but less toxic to other organs, such as the heart.^3^^,^^4^ While DOXO, ACLA, and AMR are or have been used in the clinic, their mechanisms of action differ. ACLA, used for the treatment of acute myeloid leukemia (AML) in Japan and China, has been previously shown to evict histones and therewith causes chromatin damage, but does not induce DSBs.^5^ AMR, which is known to induce DSBs but does not trigger histone eviction, is used in clinical trials for treatment of small cell lung carcinoma and has been marketed in Japan since 2002.^6^^,^^7^ The Food and Drug Administration (FDA) has granted orphan drug designation to AMR for the treatment of small cell lung carcinoma in 2008.^8^ DOXO combines the activities of ACLA and AMR but has a different DNA specificity than ACLA.^9^

Individual patients respond differently to DOXO, and cardiotoxicity cannot be predicted upfront. There is a strong need for a screening platform allowing assessment of the sensitivity of individual patients to the cardiotoxic effects of DOXO. Here, we describe the development of a toxicity screening platform, delivering read-outs on several of the proposed toxicity mechanisms, which proved essential to distinguish between toxicity mechanisms of DOXO, AMR, and ACLA. We recreated a clinically relevant treatment regime including a three-week recovery period in between drug administrations to determine the cardiotoxic effects of accumulated dose. This recovery period proved sufficient to reverse the loss of transcription factor expression in the nuclei, the reorganization of myofibrils that was lost upon treatment, and the recovery of contractile force to initial, pre-treatment level. This repeated dosing regimen in our model can be used for long-term follow-up studies on cardiotoxicity and recovery using cardiomyocytes differentiated from individual patients.

## Results

### Versatile cardiotoxicity platform recapitulates cellular anthracycline uptake by hPSC-derived cardiomyocytes

**Figure 1:**
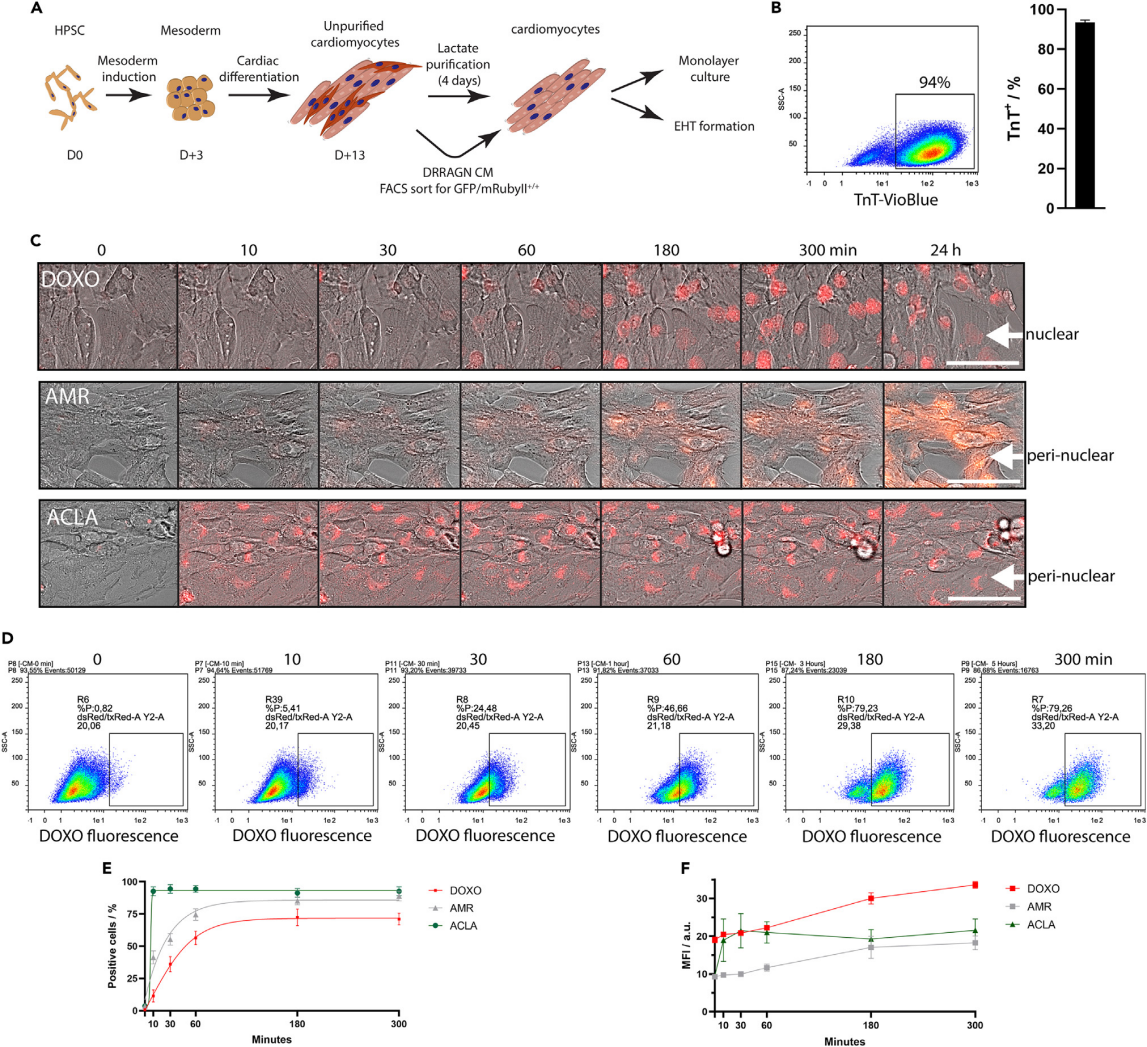
Rapid uptake of DOXO, AMR and ACLA in hPSC-cardiomyocytes (A) Cardiomyocytes were differentiated from hiPSCs and hESCs following a previously reported protocol. (B) Lactate-based purification enriched the cardiomyocyte population to an average 90% purity (n = 10, from 10 cardiomyocyte differentiations). (C) Incubation of hiPSC-cardiomyocyte monolayers with 1 μM DOXO, AMR or ACLA resulted in uptake of the drug as visualized by their auto-fluorescent property when excited at 542/20 nm and detected at 593/40 nm. Scale bar = 75 μm. (D) Representative flow cytometry plots depicting DOXO uptake into hiPSC-cardiomyocytes after exposure to 1 μM DOXO, confirming the uptake kinetics observed by microscopy. (E and F) Flow cytometry data quantified as percentage of positive cells or mean fluorescent intensity (MFI) in drug positive hiPSC-cardiomyocytes. Data shown as means ± s.e.m with interpolated data (n = 3, from 3 cardiomyocyte differentiations). See also Figure S1.

We developed a versatile platform for the assessment of cardiotoxicity utilizing hPSC-derived cardiomyocytes in 2D monolayer and 3D EHTs and applied it to evaluate the adverse effects of DOXO, ACLA and AMR on cardiomyocyte survival and function. HPSCs were differentiated to cardiomyocytes and purified by lactate metabolism selection as described previously,^10^ which resulted in a cell population comprising more than 90% cardiac Troponin T^+^ cells as quantified by flow cytometry (93.5 ± 1.2%; n = 10) (Figures 1A and 1B). We confirmed drug uptake by exposing the hPSC-cardiomyocytes to the drug compounds at a concentration of 1 μM over the course of 24 h while observing the intrinsic fluorescent properties of DOXO, AMR and ACLA in the cell by fluorescent microscopy (Figures 1C and S1A) or quantifying the fluorescent signal by flow cytometry at multiple time points (Figures 1D–1F). Intracellular ACLA was observed 10 min after start of exposure, while DOXO and AMR were detected only after 60 min (Figure 1C). Interestingly, we located the DOXO signal inside the nucleus, while AMR and ACLA autofluorescence was detected at a peri-nuclear location (Figure 1C). ACLA fluorescent signal is quenched by DNA binding, which could explain the absence of a nuclear signal.^11^ Flow cytometry revealed that the percentage of positive cells increased over the first 3 h, after which the maximum of 70% DOXO positive cells was maintained (10 min: 11.5 ± 4.7%; 300 min: 71.0 ± 4.5%; n = 3) (Figures 1D and 1E). The fraction of AMR^+^ cells similarly increased over the course of 3 h and reached a maximum of 89% (10 min: 41.4 ± 5.1%; 300 min: 88.9 ± 3.8%; n = 3) (Figure 1E). In contrast, ACLA reached a plateau after 10 min, at which more than 90% of the cells were ACLA^+^ (10 min: 92.6 ± 3.4%; 300 min: 92.6 ± 3.5%; n = 3) (Figure 1E). While the proportion of anthracycline positive cells did not change after 3 h, the mean fluorescent intensity (MFI) of the anthracycline inside a single cell continued to increase for DOXO and AMR until the end of the measurement at 5 h. ACLA saturation of the cardiomyocytes reached a plateau at 30 min and remained stable for the duration of the experiment. (Figure 1F). A higher concentration (5 μM) did not alter the uptake pattern of DOXO (Figure S1B). After medium refreshment 24 h post-treatment, the auto-fluorescent signal of DOXO, ACLA and AMR could be observed for another 6 days before reaching background levels, suggesting that drug removal from the hPSC-cardiomyocytes is similar for all three anthracyclines (Figure S1C). Note that the pharmacokinetic/pharmacodynamic (PKPD) of DOXO shows a serum half-life of 20–40 h.^12^^,^^13^ The maximum uptake of the drug depends on the uptake rate and the dose in the culture medium. It is possible to use these parameters in our system to simulate clinical peak dose and maximum uptake by adjusting the incubation time of the drug-spiked culture medium on the tissues.

### DOXO and ACLA induce loss of hPSC-cardiomyocytes viability and structural integrity in a dose-dependent manner

**Figure 2:**
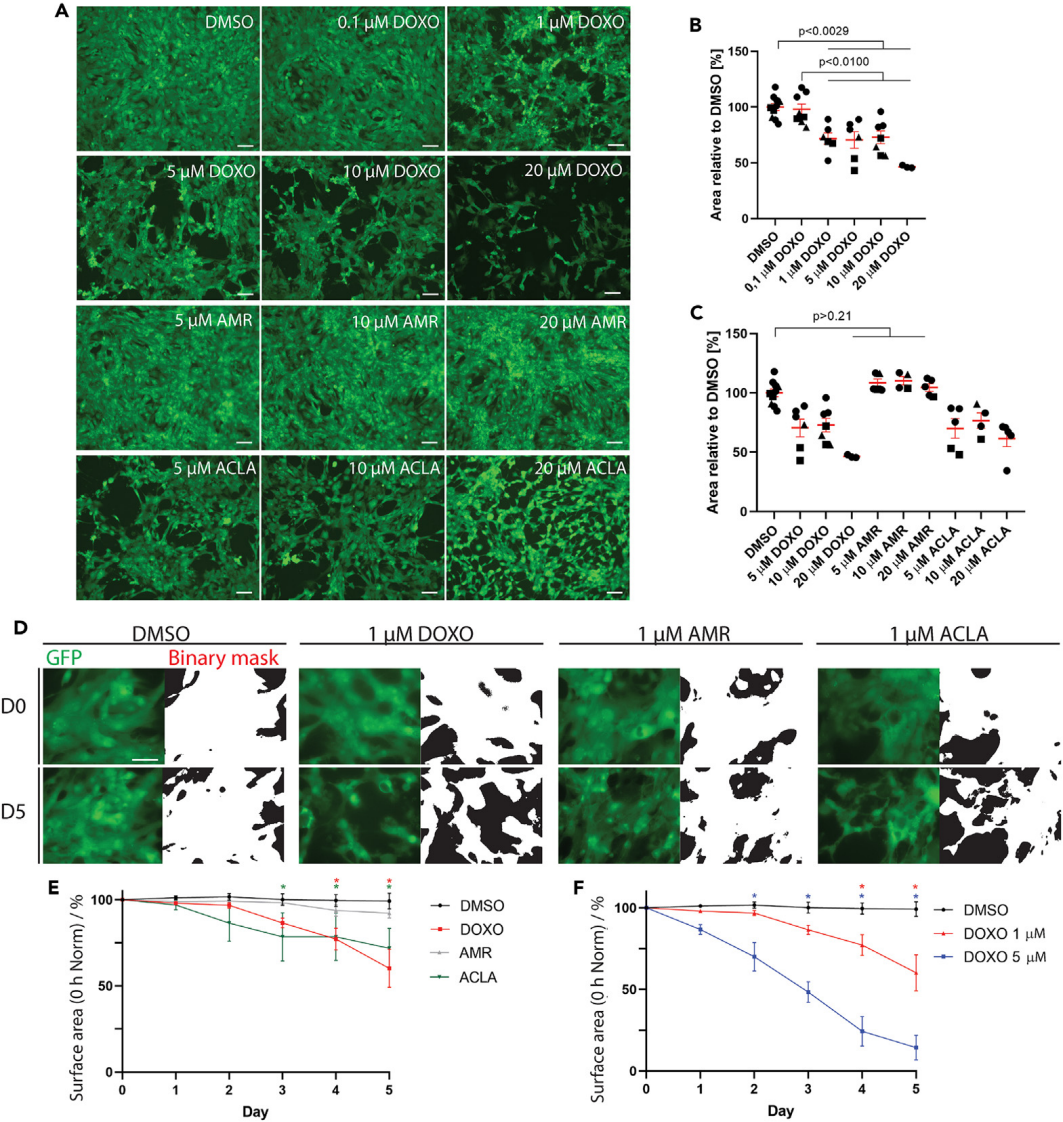
HPSC-cardiomyocyte survival upon acute exposure to DOXO, AMR or ACLA at different concentrations (A) Fluorescent micrographs Calcein live dye (green) to image the monolayer integrity of hiPSC-cardiomyocyte monolayers treated with DOXO, AMR or ACLA for 24 h. Scale bar = 100 μm. (B and C) Quantification of the area covered by hiPSC-cardiomyocyte treated with DMSO or a concentration range of DOXO, ACLA or AMR, after applying a custom ImageJ script to measure the occupied area. Data plotted as percentage means ± s.e.m, statistically tested by One-Way-ANOVA. Symbols indicate different biological replicates (n = 3–10). (D) Fluorescent micrographs of live cell imaging showing cytosolic GFP expression (left panels in green) of the NKX2.5-GFP reporter construct in DRRAGN hESC-cardiomyocytes immediately (day 0 (D0), upper panels) and after 5 days (D5, bottom panels) of exposure to 1 μM DOXO, AMR or ACLA. The right panels demonstrate the binary mask generated by the quantification macro of ImageJ. Scale bar = 50 μm. (E and F) Quantification of the surface area (as indicator of cell survival) covered by DRRAGN hESC-cardiomyocytes expressing GFP, measured daily up to 5 days after exposure to the compound. Data was normalized to timepoint 0 of the experiment. Data plotted as percentage means ± s.e.m.; DMSO and 1 μM DOXO (n = 22 and 23, from 7 cardiomyocyte differentiations), 1 μM AMR, ACLA (n = 14, from 4 cardiomyocyte differentiations) and 5 μM DOXO (n = 15, from 3 cardiomyocyte differentiations), statistically tested by two-Way ANOVA, ∗ marks statistically significant difference to DMSO control, unless indicated otherwise (Day 3 DMSO vs. ACLA: p value of 0.0357; day 4 DMSO vs. ACLA: p value of 0.0420 and DMSO vs. DOXO: p value of 0.0066; day 5: DMSO vs. ACLA p value of 0.0040 and DMSO vs. DOXO p value of <0.0001).

First, we validated the cytotoxicity of the anthracyclines by culturing undifferentiated hPSCs in medium containing 1 μM DOXO, AMR or ACLA and confirmed that the agents were highly cytotoxic to proliferating cells (Figure S1D). Then, we evaluated the effect of the drugs on hPSC-cardiomyocyte viability by exposing human induced pluripotent stem cell (hiPSC)-cardiomyocyte monolayer cultures to a range of drug concentrations and approximating the loss of cells 24 h post-treatment by quantifying the loss of surface area covered by living cells based on Calcein live cell staining (Figures 2A–2C). Loss of cells was observed in a dose-dependent manner, where a significantly lower percentage of the area was covered by viable cardiomyocytes at a DOXO concentration of 1 μM (71.74 ± 5.1%), 5 μM (70.55 ± 7.4%), 10 μM (72.89 ± 5.7%) and 20 μM (46.48 ± 0.7%), relative to Dimethyl sulfoxide (DMSO) control (100 ± 3.2%) and 0.1 μM DOXO (98.07 ± 4.7%). DOXO at 0.1 μM did not significantly reduce the cell area compared to DMSO control (Figures 2A and 2B). ACLA caused a similar loss of covered cell area at 5, 10 and 20 μM (70.07 ± 8.3%, 76.66 ± 6.5% and 61.55 ± 6.9% versus DMSO, respectively, Figures 2A and 2C). In contrast, AMR did not result in a loss of cardiomyocytes within 24 h of exposure at 5, 10 and even 20 μM (108.4 ± 3.3%, 110.2 ± 3.5% and 104.5 ± 3.1%, respectively), and confluency level was maintained at a level similar to DMSO control. This is in line with the markedly reduced cytotoxicity observed with anthracyclines only making DNA breaks (AMR) unlike those inducing nucleosome eviction (ACLA).^5^

Next, we used a previously described double fluorescent human embryonic stem cell (hESC) reporter line of mRubyII-ACTN2 and GFP-NKX2.5 (DRRAGN),^14^ which was differentiated to cardiomyocytes and purified by FACS sorting on GFP (NKX2.5) and mRubyII (ACTN2). Monolayers of hESC-cardiomyocytes were cultured in medium containing 1 μM DOXO, AMR or ACLA, which, according to the findings presented in Figure 2A, would allow for the induction of cell damage without causing immediate cell death, to evaluate cardiomyocyte survival and sarcomeric integrity over a 5-day time interval. Using the cytosolic GFP signal expressed by these cardiomyocytes, we evaluated cell survival by quantifying the total surface area covered by GFP signal, comparable to the Calcein quantification described previously (Figures 2D and 2E). Similar to the results in Figures 2A–2C, we observed that 1 μM ACLA and 1 μM DOXO caused gradual cell loss over time (surface area coverage of 71.8 ± 11.5%; n = 4 and 60.2 ± 11.0%; n = 7, respectively), although variability between biological replicates was observed for the 1 μM DOXO condition. Treatment with 5 μM DOXO resulted a more profound and reproducible loss of cell area (survival of 14.3 ± 7.5%; n = 3) (Figure 2F). DMSO control and 1 μM AMR did not significantly reduce the surface area covered by GFP^+^ cells (survival of 99.2 ± 4.5%; n = 7 and 91.2 ± 2.8%; n = 4, respectively) (Figure 2E). These findings corroborate our previous results and demonstrate that both hPSC lines respond in a similar manner.

**Figure 3:**
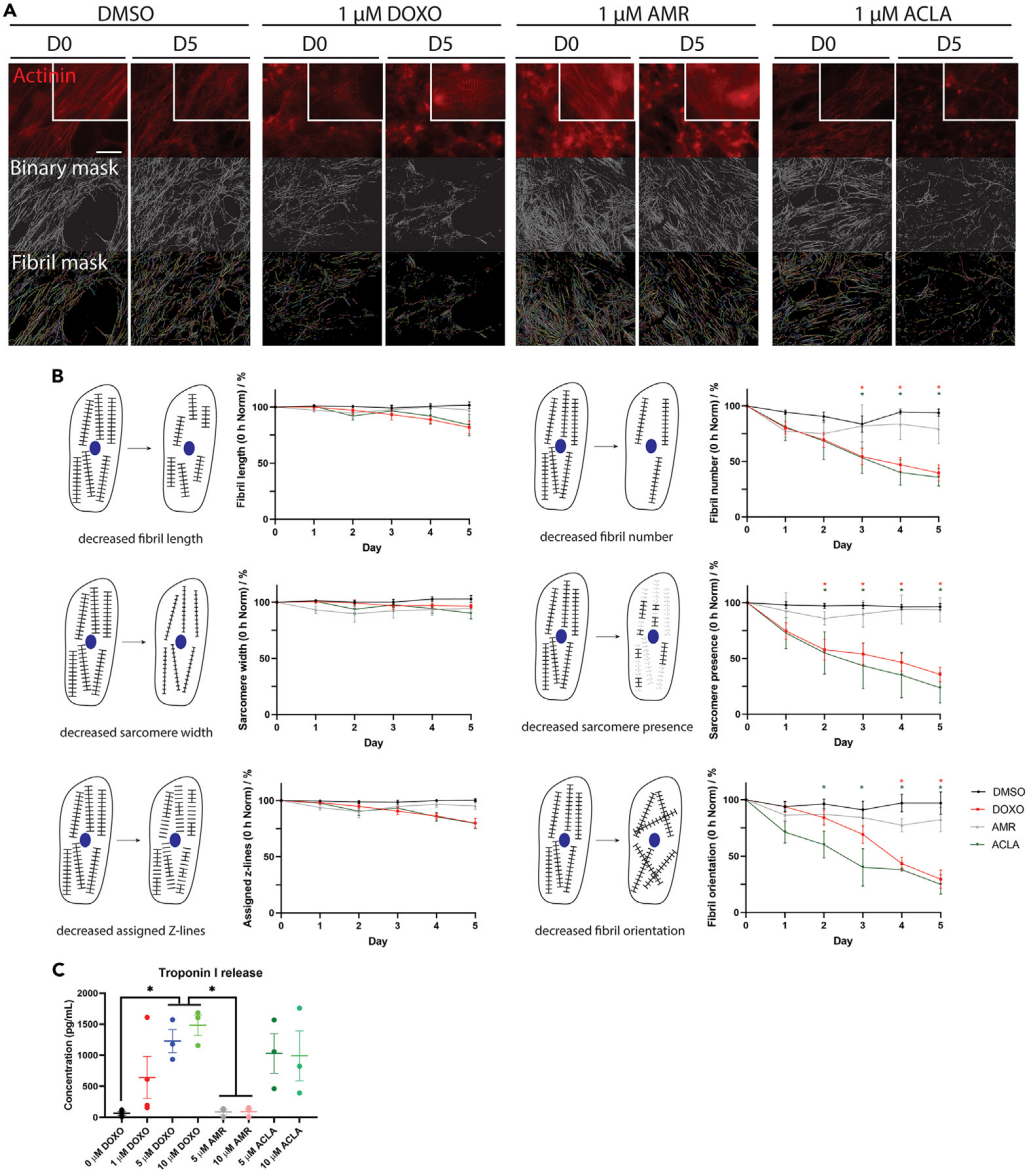
Sarcomeric disarray after exposure to 1 μM DOXO, AMR or ACLA (A) Fluorescent micrographs showing sarcomeric expression of α-actinin-mRubyII expression (upper panels in red) in DRRAGN hESC-cardiomyocyte monolayers prior to (D0, left panels) and after 5 days of exposure to 1 μM DOXO, AMR or ACLA (D5, right panels). Lower panels demonstrate the binary mask of the sarcomeric area or fibril mask. Scale bar = 50 μm. (B) Quantification of different parameters for assessment of sarcomeric disorganization after drug exposure, measured daily up to 5 days after exposure to the compound and normalized to timepoint 0 of the experiment. Data plotted as percentage means ± s.e.m., statistically tested by two-way ANOVA, ∗ marks statistically significant difference to DMSO control, unless indicated otherwise, DMSO and 1 μM DOXO (n = 22 and 23, from 7 cardiomyocyte differentiations), 1 μM AMR (n = 12, from 3 cardiomyocyte differentiations), ACLA (n = 14, from 4 cardiomyocyte differentiations)(Day 3 DMSO vs. ACLA: p value of 0.0357; day 4 DMSO vs. ACLA: p value of 0.0420 and DMSO vs. DOXO: p value of 0.0066; day 5: DMSO vs. ACLA p value of 0.0040 and DMSO vs. DOXO p value of <0.0001). (C) Cardiac troponin I release as measured by ELISA in the medium of hPSC-monolayers 4 days after drug exposure. Data presented as means ± s.e.m. plus individual values, statistically tested by One-Way-ANOVA, DMSO (n = 3 cardiomyocyte differentiations), 1 μM DOXO (n = 4 cardiomyocyte differentiations), 5 μM DOXO (n = 3 cardiomyocyte differentiations), 10 μM DOXO (n = 3 cardiomyocyte differentiations), 5 μM AMR (n = 3 cardiomyocyte differentiations), 5 μM ACLA (n = 3 cardiomyocyte differentiations), 10 μM AMR (n = 3 cardiomyocyte differentiations), 10 μM ACLA (n = 3 cardiomyocyte differentiations).

In addition to the cytosolic GFP expression in cardiomyocytes derived from the DRRAGN hESC reporter line, the fusion of red fluorescent protein mRubyII with the sarcomeric protein α-actinin allows the visualization and live imaging of the characteristic striated organization of cardiomyocytes. To assess whether exposure to 1 μM of either drug would affect the structural integrity of the cardiomyocyte, we evaluated the organization of sarcomeres using live cell imaging of the fluorescent α-actinin-mRubyII protein expression during 5 days of exposure (Figure 3). Through custom software,^15^ we determined that the myofibrillar organization was significantly affected upon exposure to DOXO and ACLA over the time course of 5 days, compared to the control (DMSO) group (Figures 3A and 3B). This is apparent in the loss of sarcomeric signal occurring over the course of the 5 days, losing up to 75% of sarcomeres when exposed to DOXO or ACLA, as opposed to AMR treatment, which did not affect sarcomere presence (sarcomere presence: DMSO: 96.5 ± 2.8%; n = 6; DOXO: 35.7 ± 6.6%; n = 7; AMR: 93.7 ± 10.8%; n = 3; ACLA: 23.8 ± 13.7%; n = 4). Accordingly, the number of fibrils and their orientation, which are an approximation of fibril organization, were substantially reduced by the exposure to DOXO and ACLA, while AMR and DMSO treatment did not affect these parameters (fibril number: DMSO: 94.0 ± 3.3%; n = 7; DOXO: 40.0 ± 7.3%; n = 7; AMR: 79.3 ± 13.4%; n = 3; ACLA: 36.7 ± 7.9%; n = 4); (fibril orientation: DMSO: 97.2 ± 9.8%; n = 7; DOXO: 30.0 ± 8.1%; n = 7; AMR: 82.5 ± 10.8%; n = 3; ACLA: 25.0 ± 8.6%; n = 4). This effect can be readily appreciated in the fluorescent images (Figure 3A). We continued to quantify sarcomere width (the length of the α-actinin fluorescent signal (Z lines)), the fibril length (the length of a continuous fibril) and assigned Z-lines (the percentage of assigned z-lines is the number of z-lines that are part of myofibrils divided by the total number of z-lines that are identified from the original image) during 5 days after exposure to a single dose of the compounds. Interestingly, we found that individual sarcomeres appear morphologically unaffected by the anthracyclines, in contrast to the myofibril as a whole (sarcomere width: DMSO: 103.0 ± 3.2%; n = 7; DOXO: 96.6 ± 1.9%; n = 7; AMR: 94.5 ± 3.7%; n = 3; ACLA: 90.3 ± 5.0%; n = 4); (fibril length: DMSO: 101.7 ± 2.9%; n = 6; DOXO: 81.8 ± 6.0%; n = 7; AMR: 97.3 ± 3.5%; n = 3; ACLA: 84.0 ± 9.6%; n = 4); (assigned z-lines: DMSO: 100.3 ± 1.5%; n = 7; DOXO: 80.0 ± 4.3%; n = 7; AMR: 95.3 ± 3.0%; n = 3; ACLA: 79.7 ± 4.6%; n = 4).

The detrimental effect of DOXO and ACLA on myofibril organization and cell viability was further supported by the release of troponin I (TnI) into the culture medium, a clinical biomarker for cardiomyocyte damage or death. We exposed hiPSC-cardiomyocyte monolayers to concentration ranges of DOXO, AMR and ACLA to quantify the amount of released TnI in the culture media after 4 days using an enzyme-linked immunosorbent assay (ELISA) (Figure 3C). In our monolayers, we observed a dose-dependent TnI release following exposure to DOXO, further confirming that DOXO actively damages hPSC-cardiomyocytes. ACLA clearly demonstrated an increased release of TnI at a concentration of 5 and 10 μM, which was comparable to the 5 μM DOXO-treated condition. In contrast, AMR at 5 μM and 10 μM did not trigger a higher release of TnI in the culture medium when compared to DMSO control (DMSO: 66.71 ± 31.63 pg/mL n = 3; 1 μM DOXO: 643.10 ± 339.0 pg/mL n = 4; 5 μM DOXO: 1229.0 ± 186.2 pg/mL n = 3; 10 μM DOXO: 1483.0 ± 16 4.0 pg/mL n = 3; 5 μM AMR: 87.70 ± 43.59 pg/mL n = 3; 10 μM AMR: 90.32 ± 46.28 pg/mL n = 3; 5 μM ACLA: 1030.00 ± 319.9 pg/mL n = 3; 10 μM ACLA: 991.80 ± 403.2 pg/mL n = 3).

### ACLA and DOXO, but not AMR, affect contractility in a dose-dependent manner

**Figure 4:**
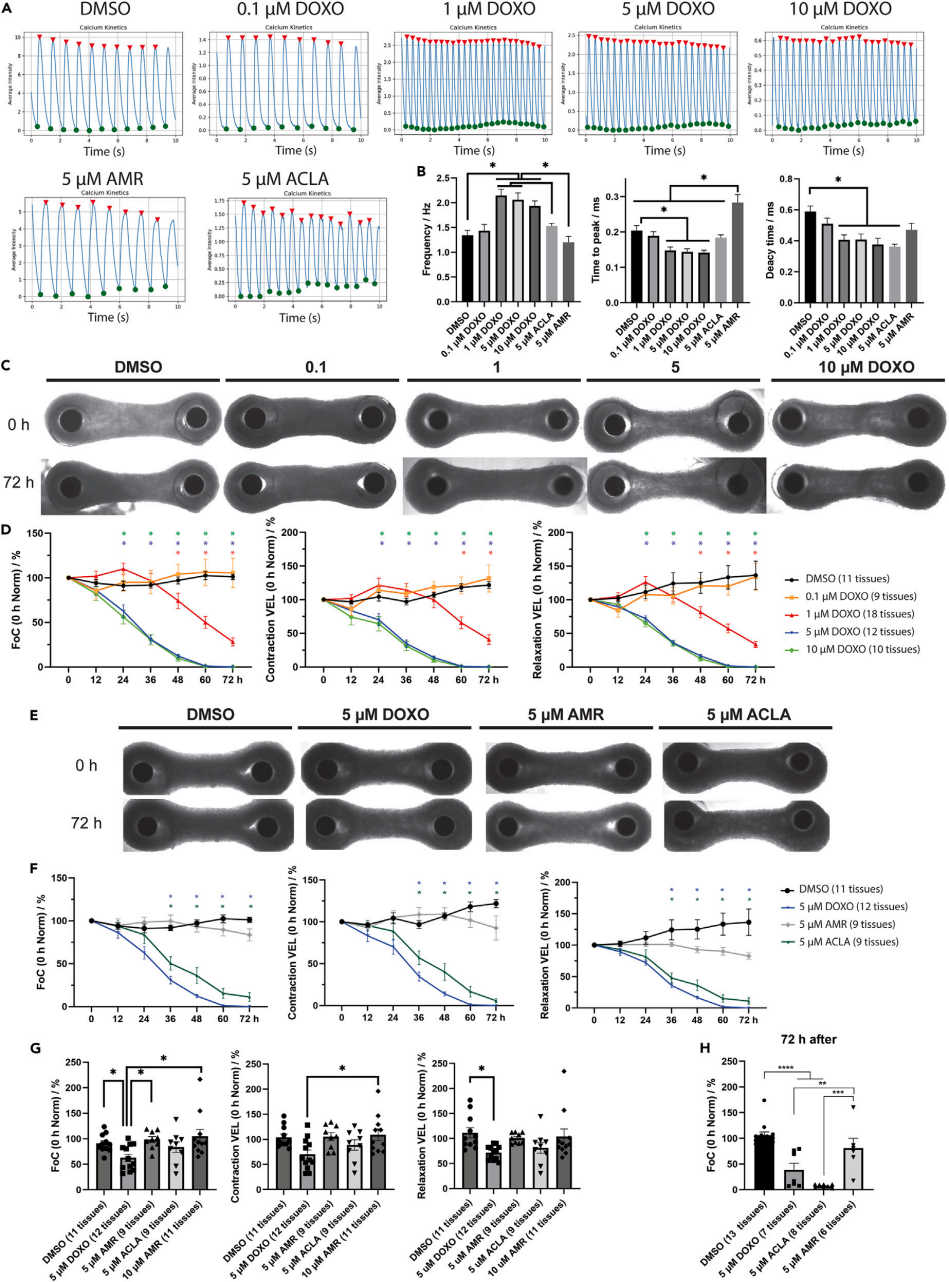
Cardiac function assessed by calcium kinetics and force of contraction (A) Representative frequency curve graphs. (B) Quantification of calcium kinetics in cardiomyocyte monolayers after 24 h exposure to different drug concentrations. Data plotted as means ± s.e.m. with DMSO (n = 24); 0.1 μM DOXO (n = 24); 1 μM DOXO (n = 25); 5 μM DOXO (n = 22); 10 μM DOXO (n = 14); 5 μM AMR (n = 16); 5 μM ACLA (n = 16), from 4 cardiomyocyte differentiations, statistically tested by One-Way-ANOVA. (C) Example micrographs of EHTs treated with DMSO or increasing concentrations of DOXO at the start (0 h) and end (72 h) of the experiment. (D) Quantification of force of contraction (FoC), contraction velocity (VEL) and relaxation VEL every 12 h for up to 72 h after drug exposure. Data was normalized to timepoint 0 of the experiment. Data plotted as percentage means ± s.e.m., statistically tested by One-Way-ANOVA (n = 8–18 EHTs per conditions, from 5 cardiomyocyte differentiations). (E) Example micrographs of EHTs treated with DMSO, or 5 μM DOXO, AMR or ACLA at the start (0 h) and end (72 h) of the experiment. (F) Quantification of FoC, contraction VEL and relaxation VEL every 12 h for up to 72 h after drug exposure. Data was normalized to timepoint 0 h of the experiment. Data plotted as percentage means ± s.e.m., statistically tested by two-Way ANOVA, ∗ marks statistically significant difference to DMSO control, unless indicated otherwise (n = 9–11 EHTs per condition, from 5 cardiomyocyte differentiations). (G) Quantification of FoC, contraction VEL and relaxation VEL 24 h after drug exposure. Data was normalized to timepoint 0 h of the experiment. Data plotted as percentage means ± s.e.m., statistically tested by One-Way-ANOVA. (H) Force of contraction measured in EHTs 72 h after a treatment of 5 μM DOXO, AMR or ACLA for only 2 h, followed by complete washout (n = 6–13 EHTs per conditions, from 3 cardiomyocyte differentiations), ∗p < 0.05; ∗∗p < 0.01; ∗∗∗p < 0.001; ∗∗∗∗p < 0.0001. See also Figure S2.

Since Calcium ions (Ca^2+^) overload and arrhythmic behavior is found *in vivo* after anthracycline exposure, we determined the kinetics of Ca^2+^ ions in hPSC-cardiomyocytes. Monolayers of hiPSC-cardiomyocytes were exposed to DOXO at various concentrations (ranging from 0.1 to 10 μM), 5 μM AMR, 5 μM ACLA, or DMSO as control for 24 h. The ACLA and AMR concentration of 5 μM instead of 1 μM used earlier was chosen to ensure a response of the cells within 24 h of exposure. Using Fluo-4 AM calcium dye, we quantified Ca^2+^ fluctuations inside the cell and used these measurements to determine Ca^2+^ ion kinetics and contraction frequency. DOXO concentrations above 1 μM substantially affected normal Ca^2+^ handling, as indicated by the significant increase in beating frequency, as well as the reduction of the time-to-peak and time-to-decay of Ca^2+^ signal (Figures 4A and 4B). Analogues ACLA and AMR did not significantly alter the contraction frequency, however, 5 μM AMR did trigger an elongated time-to-peak compared to all other conditions, indicating a delayed inflow of calcium ions into the cell. Because the time-to-decay remained low, this elevated time-to-peak caused by AMR did not result in an overall reduced frequency of contraction, although such a trend may be observed.

We proceeded to evaluate the effect of each drug on cardiomyocyte contractility in a 3D EHT format after repetitive treatment with a concentration range of DOXO, AMR, or ACLA (EHTs were treated with the compounds every 24 h). We quantified force of contraction (FoC) and contraction kinetics every 12 h for 3 days. Whereas on a macroscopic level the EHT integrity remained intact throughout the experiment (Figure 4C), contractility of the tissues was affected substantially (Figure 4D). FoC and contraction kinetics remained unaffected over three days of culture for the DMSO treated control and the 0.1 μM DOXO group (DMSO: FoC: 101.1 ± 3.1%, Contraction VEL: 121.8 ± 5.0%, Relaxation VEL: 136.5 ± 21.0%, n = 11; 0.1 μM DOXO: FoC: 105.5 ± 16.4%, Contraction VEL: 131.6 ± 20.1%, Relaxation VEL: 134.0 ± 20.0%, n = 9). 1 μM DOXO exposure triggered an intermediate decrease of force overtime with some variability between biological replicates, while 5 and 10 μM DOXO resulted in a complete loss of measurable force of contraction 60 h after drug exposure (at 72 h, 1 μM DOXO: FoC: 28.1 ± 4.6%, Contraction VEL: 40.8 ± 7.2%, Relaxation VEL: 33.5 ± 4.4%, n = 18) (Figure 4D). A similar trend can be observed for contraction and relaxation speed of the tissues, suggesting that DOXO affects the contractile capability of hPSC-cardiomyocytes in a dose-dependent manner. 5 μM AMR treatment did not affect force of contraction and only slightly affected contraction and relaxation velocities (at 72 h, 5 μM AMR: FoC: 83.5 ± 7.1%, Contraction VEL: 92.6 ± 14.4%, Relaxation VEL: 82.6 ± 5.2%, n = 9 (Figures 4E and 4F). Because the tissues treated with AMR did not appear to be affected in their contraction kinetics, we increased the concentration to determine if and at what level AMR would start to disrupt tissue functionality. We found that at 10 and 20 μM AMR, the tissues showed reduced FoC and contraction and relaxation velocities to 40% or 0% of the original value, respectively (10 μM AMR: FoC: 40.0 ± 6.9%, Contraction VEL: 50.7 ± 7.7%, Relaxation VEL: 40.8 ± 6.7%, n = 11; 20 μM AMR: FoC: 0.0 ± 0.0%, Contraction VEL: 0.0 ± 0.0%, Relaxation VEL: 0.0 ± 0.0%, n = 3) (Figure S2A*).* Similar to 5 μM DOXO, ACLA affected FoC and contraction kinetics at all tested concentrations (5 μM ACLA: FoC: 11.2 ± 5.2%, Contraction VEL: 5.8 ± 2.4%, Relaxation VEL: 11.1 ± 5.0%, n = 9) (Figures 4E and 4F). Interestingly, when AMR and ACLA were mixed together in a 1:1 ratio, the performance of the tissue was reduced to the equal value of ACLA alone at the corresponding concentration, suggesting that ACLA alone was responsible for a reduction in performance, and AMR could not alleviate or aggravate the effect of ACLA (2.5 μM AMR + ACLA: FoC: 10.1 ± 2.3%, Contraction VEL: 14.1 ± 3.3%, Relaxation VEL: 13.9 ± 2.9%, n = 11; 5 μM AMR + ACLA: FoC: 2.6 ± 1.3%, Contraction VEL: 3.7 ± 1.8%, Relaxation VEL: 3.7 ± 1.9%, n = 9) (Figures S2B and S2C). Important to note is that ACLA, in contrast to DOXO, did not cause any detrimental effect on EHT contraction within the first 24 h (Figures 4F and 4G), while over the course of 72 h, ACLA clearly affected contraction to a similar extent as DOXO at the same concentration (DMSO: FoC: 91.1 ± 5.3%, Contraction VEL: 104.3 ± 6.4%, Relaxation VEL: 111.6 ± 10.2%, n = 11; 5 μM DOXO: FoC: 62.5 ± 7.1%, Contraction VEL: 70 ± 8.8%, Relaxation VEL: 72.1 ± 4.0%, n = 12; 5 μM AMR: FoC: 98.4 ± 5.8%, Contraction VEL: 105.3 ± 7.9%, Relaxation VEL: 101 ± 3.5%, n = 9; 5 μM ACLA: FoC: 83.7 ± 10.5%, Contraction VEL: 88.7 ± 10.3%, Relaxation VEL: 81.6 ± 10.9%, n = 9; 10 μM AMR: FoC: 105.1 ± 13.2%, Contraction VEL: 109.2 ± 11.5%, Relaxation VEL: 104 ± 15.0%, n = 11) (Figure 4G).

Based on the uptake speed of the individual anthracyclines, we then postulated that the detrimental effect of either drug can be modulated by altering the exposure time to influence the maximum exposure of the cell. Thus, we incubated EHTs with 5 μM of DOXO, ACLA or AMR for 2 h, followed by a wash-out and subsequent force measurement 72 h post treatment. As seen in Figure 4H, a 2-h incubation with AMR (resulting in ∼80% saturation of the cells, see Figure 1F) did not result in a reduction of contraction force, as expected. However, this treatment with ACLA (corresponding to 100% saturation of the cells) resulted in a loss of FoC very similar to the loss we observed when repeating the incubation on a daily basis as seen in Figure 4F. DOXO incubation for 2 h at 5 μM (corresponding to ∼70% saturation, Figure S1) did not result in a complete loss of force that can be observed if the tissues were treated daily, however, the force decreased to 40% of initial level (DMSO: FoC: 106.6 ± 6.1%, n = 13; 5 μM DOXO: FoC: 38.4 ± 13.1%, n = 7; 5 μM AMR: FoC: 81.2 ± 18.9%, n = 6; 5 μM ACLA: FoC: 6.7 ± 1.2%, n = 8) (Figure 4H).

### A platform to assess different cardiotoxicity mechanisms

#### DOXO and AMR, but not ACLA cause double strand DNA breaks

**Figure 5:**
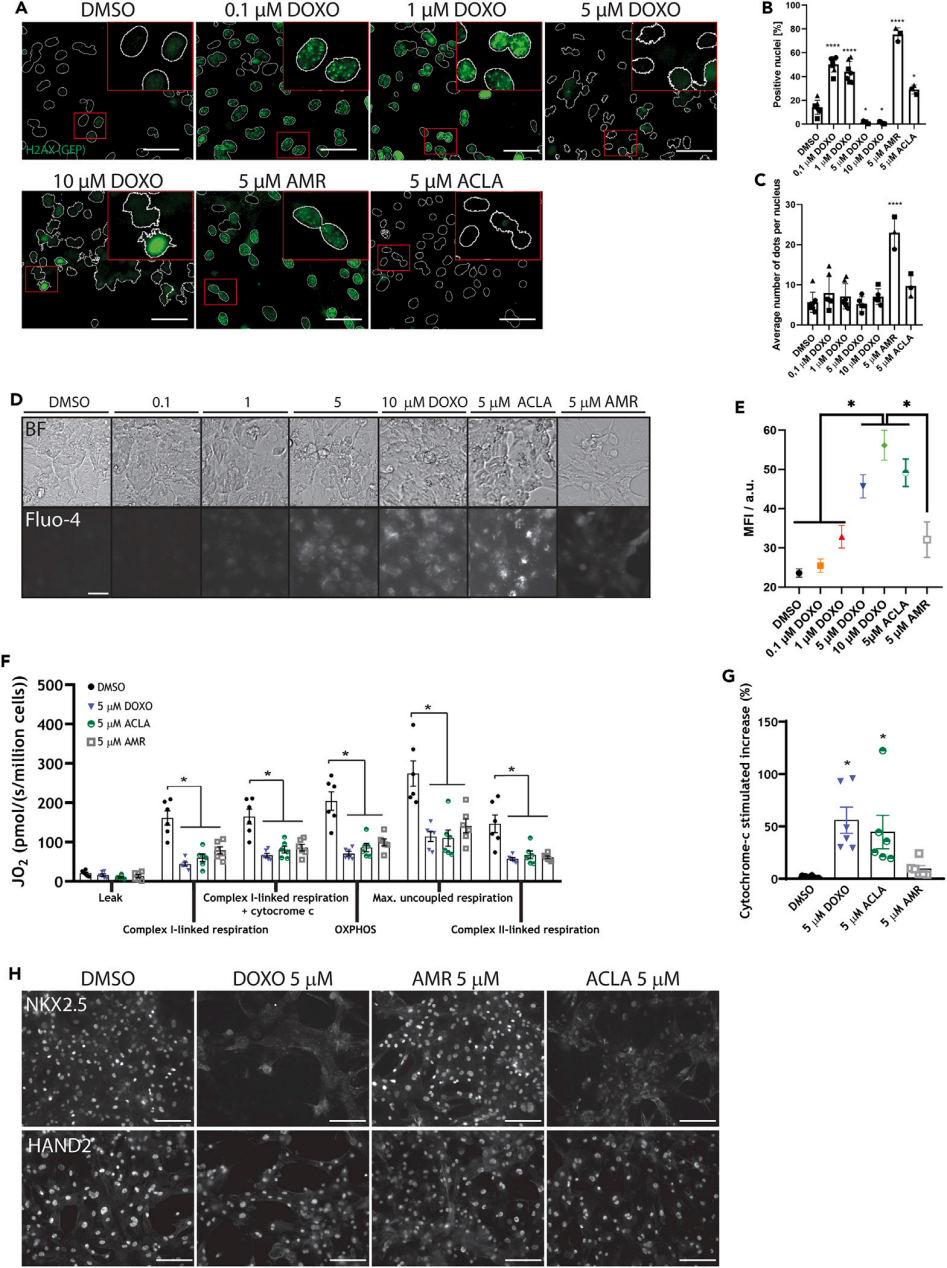
Indicators of cell damage by DOXO, AMR and ACLA (A) Micrographs of an immuno-labeled phosphorylated H2AX (gH2AX) in hiPSC-cardiomyocyte monolayers treated with DOXO, AMR or ACLA for 24 h. Inset displays a magnification of the area indicated with a rectangle. Green dots in the nuclei represent gH2AX. Scale bar = 50 μm. (B) Quantification of the number of nuclei positive for gH2AX and (C) The number of dots per positive nuclei. Data plotted as percentage means ± s.e.m, statistically tested by One-Way-ANOVA. Symbols represent biological replicates (n = 3–7, from 3 cardiomyocyte differentiations). ∗p < 0.05; ∗∗∗∗p < 0.0001. (D) Micrographs of Calcium dye Fluo-4a.m. live staining in monolayers. Scale bar = 50 μm. (E) Quantification of the fluorescent signal of Fluo-4a.m., as an indicator of accumulated Ca^2+^ in the cardiomyocyte monolayers, with DMSO (n = 34); 0.1 μM DOXO (n = 25); 1 μM DOXO (n = 27); 5 μM DOXO (n = 26); 10 μM DOXO (n = 25); 5 μM AMR (n = 17); 5 μM ACLA (n = 22), from 4 cardiomyocyte differentiations. (F) Oxygen consumption rate of hiPSC-EHTs after 24 h exposure to 5 μM DOXO, AMR or ACLA, specified per mitochondrial process. (G) Quantification of outer-mitochondrial membrane damage in EHTs. (F and G) Data plotted as means ± s.e.m., statistically tested by One-Way-ANOVA, ∗ marks statistically significant difference to DMSO control, unless indicated otherwise (n = 6 EHTs per condition, from 6 cardiomyocyte differentiations). (H) Immunohistochemical analysis of transcription factor protein expression following exposure to anthracyclines in cardiomyocyte monolayers. Scale bar = 100 μm. See also Figures S3 and S6.

Anthracyclines elicit their cardiotoxic effect through different underlying mechanisms, including apoptosis, DNA damage, calcium accumulation, metabolic dysfunction and reduced expression of cardiac markers.^1^ One aspect of DOXO induced cell damage is the development of DNA damage in the cell. For this reason, we assessed the induction of DNA DSBs in our platform by quantifying the number of cells expressing phosphorylated histone H2AX (gH2AX), which is known to be triggered upon DNA DSB repair. Monolayers of hPSC-cardiomyocytes were treated with a concentration range of DOXO, AMR or ACLA for 24 h, and then fixed for immunocytochemical analysis of gH2AX (Figures 5A–5C and S3). We found that, compared to DMSO-treated control cells, substantially more cardiomyocytes exposed to 0.1 μM or 1 μM DOXO were positive for gH2AX in the nucleus (Figures 5A, 5B, and S3). However, at higher concentrations of DOXO, the fluorescent signal of gH2AX as well as the DAPI counterstain appeared to fade. This effect may be due to histone eviction, which has been described before.^11^ Interestingly, AMR-treated cells displayed gH2AX signal at all concentrations, whereas ACLA did not show this DNA damage response (Figures 5A–5C and S3). Higher concentrations of AMR or ACLA did not change this observation, as 10 and 20 μM AMR and ACLA resulted in the same pattern or DSB induction compared to their respective 5 μM condition (Figure S3). Thus, DOXO induced DSBs at concentrations as low as 0.1 μM, a level that appeared non-toxic in the viability assay and in the EHT data. Similarly, AMR induced substantial phosphorylation of H2AX, while little to no toxicity was observed in previous assays.

#### DOXO and ACLA, but not AMR trigger accumulation of calcium ions inside the cell

Previous studies reported a disturbance in calcium ion handling by cardiomyocytes upon exposure to DOXO,^16^^,^^17^^,^^18^ which may be one contributing factor to the clinically described pro-arrhythmic properties of the drug. Consequently, we evaluated the handling of calcium ions in hPSC-cardiomyocytes by exposing a monolayer of the cardiomyocytes to a range of DOXO concentrations, 5 μM AMR or ACLA, or DMSO as control, using Fluo-4-AM Calcium imaging dye. We therefore recorded the flux of calcium ions into and out of the cell, selected the frame that represented complete relaxation (which should provide the baseline calcium load of the cell), and compared these between conditions (Figures 5D and 5E). We found an increase in fluorescent signal of the dye inside the cardiomyocytes in resting phase of contraction with increasing DOXO concentrations, indicating a higher concentration of calcium ions that are retained in the cell after relaxation (DMSO: 23.60 ± 1.05; 0.1 μM DOXO: 25.48 ± 1.70; 1 μM DOXO: 32.88 ± 2.89; 5 μM DOXO: 48.28 ± 3.83; 10 μM DOXO: 56.16 ± 3.82, Figure 5E). ACLA at 5 μM also increased the Ca^2+^ load of the cells comparable with the lead at 5 μM DOXO (5 μM ACLA: 49.15 ± 3.50 versus 5 μM DOXO = 48.28 ± 3.83). AMR in contrast only slightly increased retainment of Ca^2+^ equal to intensity to the level of DOXO 1 μM (5 μM AMR: 32.11 ± 4.53 versus 1 μM DOXO: 32.88 ± 2.89).

#### Anthracycline treatments alter mitochondrial function

Disruption of mitochondrial function is thought to be an important mechanism of anthracycline-induced cardiotoxicity.^19^ Therefore, we assessed mitochondrial respiratory performance after 24 h of DOXO, ACLA or AMR treatment in EHTs by respirometry. Respiration via complex I and II, OXPHOS capacity and uncoupled respiration were significantly reduced in all anthracycline conditions when compared to DMSO (Figure 5F and S4). This decrease in activity suggests an overall loss of mitochondrial respiratory function in hiPSC-cardiomyocytes treated with DOXO, ACLA or AMR to a similar degree. However, when cytochrome *c* was added to assess the integrity of the outer mitochondrial membrane, a significantly higher increase in complex I-linked respiration in hiPSC-cardiomyocytes exposed to DOXO and ACLA was observed (Figure 5G). Since the added cytochrome *c* could reach the respiratory complexes in the inner membrane, the elevated respiration indicates that treatment with DOXO and ACLA inflicted damage to the outer mitochondrial membrane, resulting in loss of cytochrome *c* from the intermembrane space.

#### Anthracycline treatment causes cardiac transcription factor depletion in hPSC-cardiomyocytes

The loss of nuclear localization of transcription factors (TFs), such as MEF2c and NKX2.5, upon DOXO exposure has been reported.^20^ This may represent another mechanism of toxicity to the heart, since these transcription factors are known to play a significant role not only in heart development *in vivo*, but also in cardiomyocyte homeostasis in adulthood. We therefore investigated whether analogues ACLA and AMR could also trigger the loss of TF localization in the nuclei of treated cells. To this end, we cultured hPSC-cardiomyocytes in monolayer format, treated them for 24 h with 5 μM DOXO, ACLA or AMR and fixed the cells after incubation for immunohistochemical analysis. To verify whether the loss of localization is limited to NKX2.5 and MEF2c, we included HAND2 and IRX4 as two additional cardiac TFs, as well as TBP as a universally expressed TF and not exclusive to the heart. Nuclear staining was performed with DAPI, however, since DOXO diminishes DAPI signal, we included the auto fluorescent nuclear signal of DOXO (in red) as well. As expected, NKX2.5 and MEF2c protein could not be detected in the nuclei of hPSC-cardiomyocytes 24 h after treatment with 5 μM DOXO, while the DMSO control demonstrated a clear colocalization with the nucleus (Figures 5H and S5). Whereas IRX4 nuclear localization was also lost, HAND2 was clearly present in the nuclei of cardiomyocytes 24 h after exposure to DOXO. In contrast, AMR did not abolish the TF colocalization with the nuclear signal for any of the tested TFs, while ACLA at 5 μM triggers a loss of transcription factors NKX2.5, MEF2c and IRX4, but not HAND2, similar to DOXO. TBP was retained in the nucleus in all treatment groups, similar to HAND2 (Figure S5). Subsequently, we performed a time lapse experiment to monitor the depletion of NKX2.5 in response to DOXO in 2-h intervals (Figure S6). Depending on the DOXO concentration, NKX2.5 is lost in the nucleus after 8 h with a concentration of 1 μM DOXO but depletes in only 4 h when the cells were exposed to 10 μM DOXO. This points toward a concentration-dependent knock down of the transcription factor, possibly connected to the kinetics of DOXO uptake into the cell.

#### Transcriptome analysis reveals differential expression patterns upon anthracycline treatment

**Figure 6:**
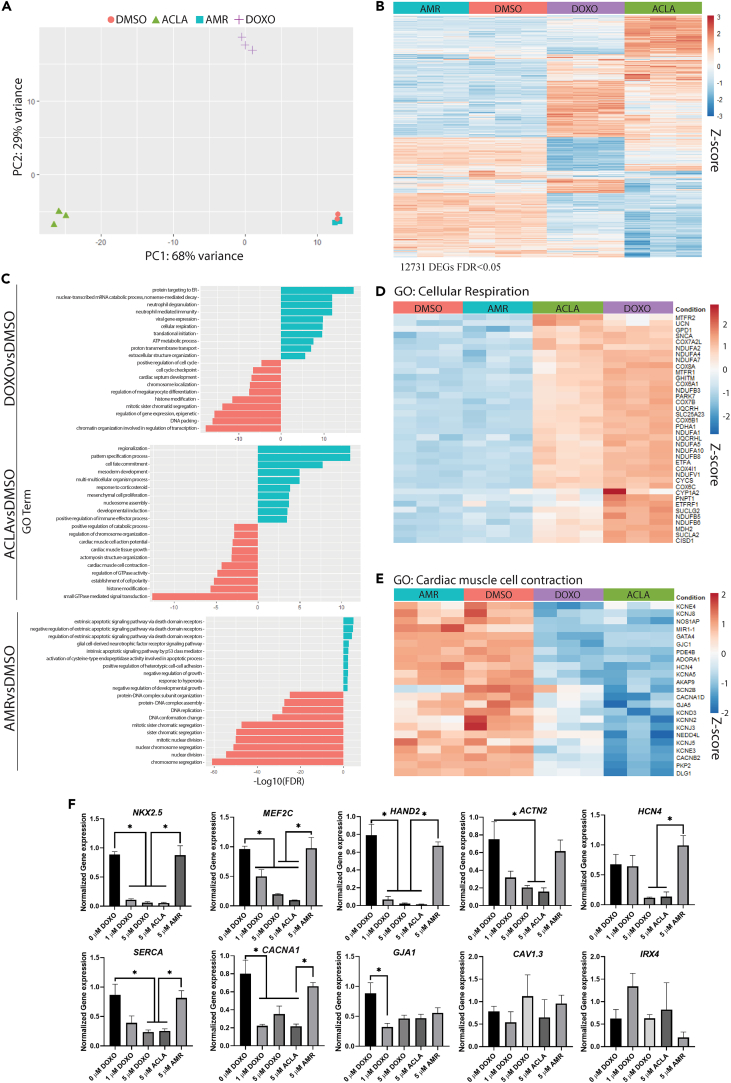
Transcriptional profiling of EHTs after exposure to anthracyclines (A) PC analysis of bulk RNA-seq of hiPSC-EHTs after 24 h of exposure to DMSO, 5 μM DOXO, AMR or ACLA (n = 3 cardiomyocyte differentiations, each with 3 EHTs per sample). Colors and symbols represent different samples. (B) Heatmap showing gene expression of the differentially expressed genes (12731 DEGs, selected with a False Discovery Rate (FDR) < 0.05) of each anthracycline versus control across all four groups (DMSO, DOXO, ACLA, AMR). (C) GO biological process terms up- and down-regulated in each treatment (DOXO, ACLA and AMR) versus control (DMSO) (p_adj_ < 0.05). (D and E) Heatmaps showing expression of genes selected from GO: cellular respiration (D) and GO: cardiac muscle cell contraction (E). (F) Gene expression analysis of selected cardiac genes by RT-qPCR in hiPSC-EHTs after 72 h of exposure to DMSO, 5 μM DOXO, AMR or ACLA (n = 3 cardiomyocyte differentiations, each with 1–2 EHTs per sample). Data plotted as means ± s.e.m., statistically tested by One-Way-ANOVA. See also Figure S7.

To further assess the cardiotoxicity induced by the different compounds at the molecular level, we performed transcriptomic analysis by RNA-sequencing in EHTs after 24 h of exposure to DMSO (control), 5 μM of DOXO, ACLA or AMR (Figure 6). With Principal component analysis (PCA) we found that global transcriptomic data of hPSC-EHTs treated with ACLA clustered independently of control and other treatments on principal component 1 (PC1, 68% variance) and hPSC-EHTs treated with DOXO also clustered independently from control and AMR-treated EHTs on principal component 2 (PC2, 29% variance). Control and AMR-treated EHTs remained clustered together indicating only a small difference in the transcriptome of AMR treated cells (Figures 6A and S7A). Compared to control, exposure to DOXO, ACLA or AMR for 24 h caused significant differential expression of 9461, 9882, and 1227 genes respectively (FDR <0.05) (Figure 6B), of which only 710 were shared among all three treatment conditions and 2 and 40 were significantly upregulated or downregulated, respectively (FDR <0.05 and LFC >1 or LFC < −1) (Figures S7B–S7H and Table S1). Interestingly, the two common upregulated genes, *MT1G* and *IGFBPL1*, were found to be involved in inhibiting DOXO-induced oxidative stress in the heart^21^ or in exhibiting tumor suppressor like properties,^22^ respectively. First exploratory analysis with gene ontology (GO) analysis of the differentially expressed genes (DEGs) in each treatment versus control revealed different terminologies in each condition. All three anthracyclines upregulated a subset of apoptotic related genes, but small differences were observed between treatments (Figures 6C and S7I*;* Table S2). Of note, while the *TP53* gene (encoding for p53) was upregulated only in ACLA-treated EHTs, several targets of this gene promoting apoptosis were enriched in DOXO or AMR (*TNFRS10A-D, ATM, FAS, E2F1* or *PMAIP1*). Only AMR-treated EHTs exhibited an upregulation of cell cycle progression genes regulated by TP53 (*CDKN1A, GADD45A*). DOXO and ACLA treatment promoted upregulation of terms related to cellular respiration, induction of ROS and downregulation of chromosome organization and histone modification (Figure 6C; Table S2). Genes associated with GO terms of cellular respiration (Figure 6D) showed dysregulation of mitochondrial electron transport components and proteins involved in the regulation of the oxidative phosphorylation in DOXO and ACLA compared to DMSO and AMR, such as genes encoding for complex I (*NDUFs*), III (*UQCRC2 and UQCRH*), IV (*COXAI1, COX8A, COX6A1, COX7B, COX6B1, COX6C*) and V (only significantly upregulated in DOXO: *ATP5F1B, ATP5MG, ATP5MF*). Interestingly, gene expression of these complexes is not significantly disturbed in AMR-treated tissues. Similarly, heatmaps representing terms associated with response to oxygen species (Figure S7J), revealed known genes involved in ROS production due to anthracycline exposure (*SOD2, NQO1* and *APOE*). Interestingly, a cascade of calpain genes (*CAPN10, CPN11, CAPN12* and *CAPN14*) were upregulated (Table S1) after exposure to both DOXO and ACLA. Calpains are activated in response of calcium overload and can cleave cytoskeletal and myofilament proteins, causing deterioration of myofibrillar proteins.^23^ In accordance, GO terms associated with cardiac muscle function, such as cardiac muscle contraction or cardiac muscle tissue growth were also downregulated in DEGs from DOXO and ACLA, (Figures 6C–6E; Table S2). Specifically, downregulation of ion channels and cardiac transcription factors, such as *GATA4*, *NKX2.5*, *MEF2C* and *HAND2* (Table S1) was observed. RT-qPCR analysis also showed that several of these genes (*NKX2.5*, *MEF2C*, *HAND2*, *ACTN2*, *HCN4*, *SERCA*, *CACNA1*, *GJA1*, *CAV1.3* and *IRX4*) remained downregulated during continuous exposure of the treatments for 3 days (Figure 6F), indicating that the first 24 h of exposure were crucial for inducing cardiomyocyte damage at gene expression level. Intriguingly, AMR-treated EHTs did not show alteration of these pathways but similar to DOXO-treated samples, GO terms related to DNA organization and damage were downregulated (Figures 6C, S7K, andS7L*;* Table S2). On the other hand, genes involved in mesoderm development and induction of development were highly upregulated exclusively in ACLA-treated EHTs, suggesting an activation of a fetal-like program of the cardiomyocytes after exposure to this drug (Figures 6C and S7M; Table S2).

Lastly, micro-RNAs (miRNAs) may also play a key role in assisting anthracycline-induced cardiotoxicity, since they can modulate gene expression. Moreover, circulating miRNAs hold great potential as biomarkers due to their stability in plasma, saliva or serum, evolutionary conservation among species and some of them are tissue specific.^24^ In agreement, we also found that miRNAs *miR-133a2, miR-208a*, and *miR-1-1* were upregulated after treatment with DOXO and ACLA. Previous studies showed that all three miRNAs were upregulated in plasma (*miR-133a2, miR1-1*) or myocardium (*miR208a)* after exposure to DOXO in mice, rat or human.^24^^,^^25^^,^^26^^,^^27^

#### Recapitulation of a clinically relevant treatment regimen reveals that sarcomeric organization and contraction of cardiomyocytes recover after exposure to DOXO or ACLA

**Figure 7:**
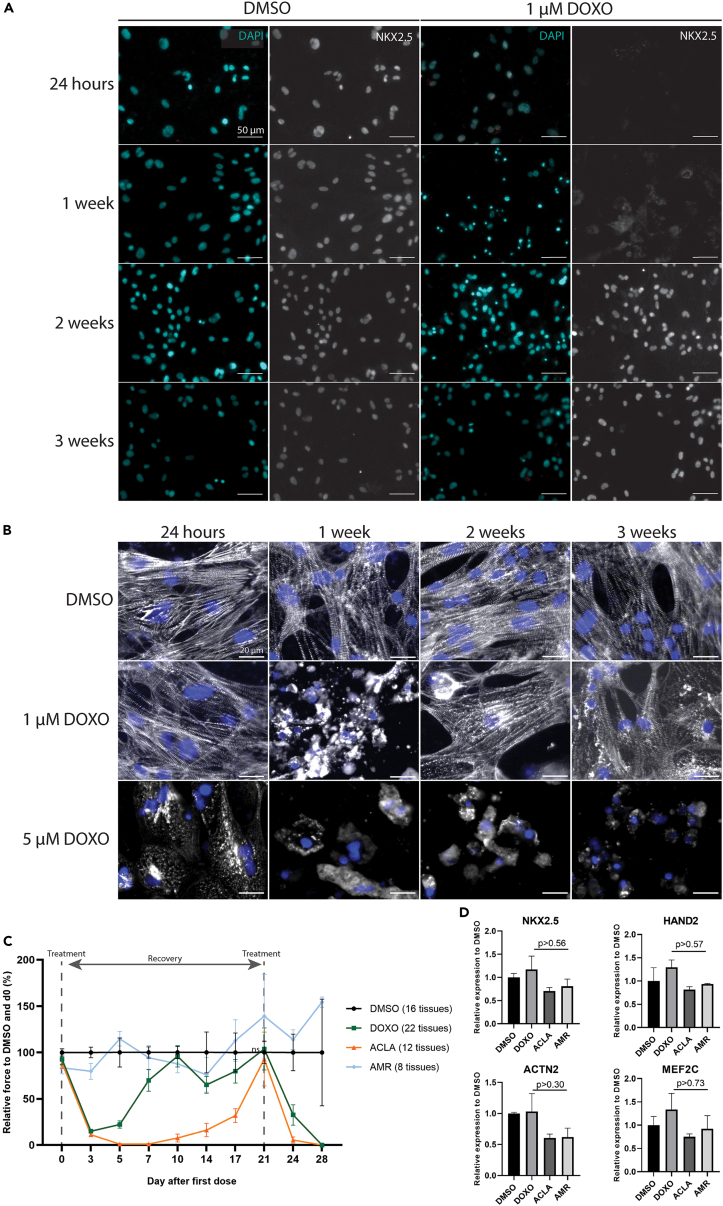
EHTs and monolayer cultures of hiPSC-cardiomyocytes recover from exposure to DOXO within 2–3 weeks post treatment (A and B) Immunostaining for NKX2.5 and α-Actinin in monolayer cardiomyocytes. (C) EHTs were exposed to 1 μM DOXO, ACLA or AMR for 24 h and force of contraction was measured every 3 days hence. Force of contraction was restored to initial level at day 21 post treatment, whereupon a second dose was administered. Data was normalized to timepoint 0 of the experiment and to DMSO. Data plotted as percentage means ± s.e.m and statistically tested by two-Way ANOVA. (n = 8–22 EHTs, from 3 cardiomyocyte differentiations). (D) Gene expression analysis of selected cardiac genes by RT-qPCR in hiPSC-EHTs after 21 days of exposure to DMSO, 1 μM DOXO, AMR or ACLA (n = 3 cardiomyocyte differentiations, each with 1–2 EHTs per sample). Data plotted as means ± s.e.m., statistically tested by One-Way-ANOVA. See also Figures S8 and S9.

In the clinic, DOXO is often administered multiple times at an interval of three weeks to allow for recovery of any affected tissues.^28^ We therefore postulated that, if hPSC-cardiomyocytes were allowed to recover between multiple treatments, the toxicity may be less severe than without recovery. Therefore, we started by determining how long the detrimental effects of DOXO, AMR and ACLA treatment on TF expression, sarcomeric organization and contraction force could be observed after treatment. To this end, cardiomyocyte monolayers were treated with 1 or 5 μM DOXO, 5 μM AMR or 5 μM ACLA for 24 h, then the complete culture medium was refreshed and the cells were maintained in culture for up to three weeks. As expected, immunostaining for TFs and sarcomeric α-Actinin showed that DMSO and AMR did not affect TF expression or sarcomere integrity over the full three-week period (Figures 7A, 7B, S8A, and S8B). In contrast, 5 μM DOXO and ACLA abolished TF expression and sarcomeric patterning of the monolayer, which could not recover in the three-week follow-up. Interestingly, at 1 μM DOXO, we observed that TF expression and α-Actinin patterning were lost up to one week post treatment, but TF expression was restored at the two-week timepoint, and sarcomeres started to re-organize (Figures 7A and 7B). These results indicate that, at sub-lethal doses of DOXO, cardiomyocytes initially lose their functional morphology and expression, but can recover within a time span of 2–3 weeks, which corresponds well with the clinical rest period between treatments.

We then mimicked the repeated dose regimen in 3D EHTs. We treated EHTs with DMSO, 1 μM DOXO, AMR or ACLA for 24 h and monitored tissue contraction force every 3 days after the initial treatment (day 0) over a course of 28 days, with a second treatment after 21 days from the first one (Figures 7C and S9A). We observed, in agreement with the previous results in monolayer cardiomyocytes, that both DOXO and ACLA initially caused a drop of force to 14% and 11% of pre-treatment and DMSO value, respectively, but demonstrated a recovery of force at 10 or 21 days after treatment, respectively. Additionally, RT-qPCR analysis of the EHTs after 21 days from the initial treatment confirmed recovery of the tissues on transcriptomic level, where no significant differences on the expression of sarcomeric, calcium handling or cardiac transcription factor genes were observed when compared to DMSO (Figures 7D and S9B). Therefore, after 21 days of recovery, a second treatment of 1 μM DOXO, ACLA or AMR was administered. DOXO and ACLA affected contraction force that could not be regained within 7 days after treatment (day 28). As expected, AMR did not affect contraction force at any of the treatments. Thus, both 2D and 3D models demonstrate a recovery period for DOXO treatment at 1 μM concentration, that can be of importance for repeated-dose treatment regimens.

## Discussion

DOXO is a cornerstone in the clinic to combat malignancies, yet after half a century of application, the drug’s therapeutic index is still limited due to severe (cardiac) side effects. Cardiotoxicity is accumulated dose dependent but there is also a striking patient variability. A personalized approach for individual patients in the future would allow selecting patients for higher doses vs. deselecting patients for application of DOXO at all. This requires an advanced system for validation. We describe such a platform of hPSC-cardiomyocyte-based assays, capable of predicting cardiotoxicity caused by anthracycline exposure, with readouts that match observations *in vivo*. We demonstrated that treatment with AMR, but not ACLA, is more favorable than DOXO in terms of cell viability and functional performance of hPSC-cardiomyocytes. Earlier studies have shown that AMR but also ACLA exhibit little to no cardiotoxicity in mice and man.^5^^,^^29^ The PKPD of ACLA reveals poor tissue penetration *in vivo*, an item that cannot be easily reproduced in minimal systems but may explain why ACLA shows toxicity of the hPSC-derived cardiomyocytes. Since the PKPD is known in the registration dossiers of all drugs, this factor can be easily incorporated in the decision making as to whether a drug will be cardiotoxic or not for an individual.

Low concentration of DOXO (0.1 μM) and higher concentrations of AMR (5 μM) had no detectable impact on hPSC-derived cardiomyocyte viability or function both in 2D and 3D within the time frame of our experiments, but induced substantial phosphorylation of H2AX, suggesting that the apparent DNA damage was not sufficient to disrupt cell function or induce apoptosis in these conditions. Therefore, although the onset of DNA damage may be measured at sub-toxic concentrations of DOXO, the presence of DSBs in our study is a poor predictor of acute cardiotoxicity caused by anthracyclines. In contrast, ACLA (5 μM) and higher doses of DOXO (1, 5 and 10 μM) induced substantial loss of viability, as also earlier observed.^5^^,^^30^ The histone eviction activity is apparently more cytotoxic than the DNA break activity. We observed the expression of a unique subset of apoptosis-related genes in ACLA, DOXO and AMR-treated cardiomyocytes. This may in part explain the loss of viability caused by DOXO and ACLA, which corresponds to previous studies reporting DOXO-induced apoptotic cell death due to p53 activation in H9c2 cardiomyocytes.^31^ However, despite triggering the expression of some pro-apoptosis genes, AMR did not cause cell death, which may be explained by the enhanced expression of *CDKN1A*, known to induce cell-cycle arrest instead of apoptosis.^32^^,^^33^ Both ACLA and DOXO, but not AMR, are known to induce histone eviction^5^ which has been associated with higher cytotoxic properties in cancer cell lines.^5^^,^^30^ Treatment with DOXO or ACLA at a histone-evicting concentration could be associated with the dysregulation of gene and protein expression found in our immunostainings, RT-qPCR and RNA sequencing data, and several functional deficiencies, such as mitochondrial dysfunction, calcium ion mishandling and loss of contraction force. In fact, the altered expression of genes involved in cellular respiration matched with the impaired mitochondrial function observed in EHTs treated with DOXO or ACLA. Conversely, AMR treatment, while still dampening respiration, did not cause apparent damage to the mitochondrial membrane, suggesting that perhaps this reflects a more physiological suppression of respiration while DOXO and ACLA induce a damage-mediated effect.

Similar to a previous study that compared the toxicity of DOXO, ACLA and AMR in hPSC-derived cardiac microtissues,^5^ we observed that only DOXO affected contractile force on our 3D EHTs within 24 h, while ACLA and AMR had no apparent effect on contractility within this time frame. However, ACLA induced a loss of contractile force equal to DOXO visible from the 36-h time point and onwards, which highlights the importance of evaluating drug-induced toxicity over a longer period of time.

When taken up by the cardiomyocytes, we showed that 1 μM DOXO abolishes the nuclear expression of NKX2.5 protein within a mere 8 h (Figure S6), whereupon it was undetectable for at least a week after treatment, which was also observed for TFs MEF2c and IRX4. NKX2.5 and possibly other TFs play important roles in cardiomyocyte homeostasis^34^^,^^35^ and their loss may therefore be a cause for cardiomyocyte dysfunction. For instance, we observed a loss of NKX2.5, IRX4 and MEF2c protein expression upon treatment with 5 μM ACLA or >1 μM DOXO, which coincided with altered gene expression patterns, mitochondrial dysfunction, calcium mishandling, and a structural disarray of the sarcomeres within 24 h, as well as a gradual loss of contraction force over the course of 3 days post-exposure in our 3D *in vitro* model. Additionally, our RT-qPCR data shows reduced expression of the Sarcoendoplasmic Reticulum Calcium ATPase (SERCA) pump in cardiomyocytes treated with DOXO or ACLA for 3 days. SERCA is responsible for the transport of calcium ions from the cytosol back to the sarcoplasmic reticulum following cardiac contraction. Reduced expression of SERCA could be related to increased accumulation of calcium ions in the cytosol after treatment with DOXO or ACLA. To what extent the loss of expression is causative or concurrent is unclear, however, aberrant Ca^2+^ handling has already been reported in a knock-out model of *NKX2.5* in hPSC-cardiomyocytes.^36^ Of note, calcium mishandling in DOXO and ACLA treated EHTs led to the upregulation of myofilament cleaving calpains, which may have led to the observed enhanced cardiac alpha actinin disorganization and contractile force reduction. The recovery time of cardiac TFs and other key elements may be crucial for the degree of adverse cardiac effects observed in patients. Patients treated with DOXO generally receive multiple administrations of the drug in intervals of roughly three weeks, with the intent to allow the patient to recover from the adverse effects of DOXO. This recovery could be recapitulated *in vitro* in our model both in terms of restored contractile force in EHTs (achieved in 10 days for 1 μM DOXO treatment) as well as in recovery of sarcomeric organization and TF expression in monolayer cultures (achieved in <2 weeks for 1 μM treatment), which may be a direct result of DOXO removal from the cell nucleus (∼7 days post treatment). The recovery of EHTs from ACLA treatment was substantially longer than DOXO (achieved in 21 days for 1 μM ACLA treatment), while initial functional impairment caused by ACLA and DOXO is highly similar. This, together with the lack of DSBs, a washout time equal to DOXO and AMR, and the upregulation of mesoderm related genes, points to a different damage mechanism of ACLA. In fact, the prolonged recovery time of the ACLA treated EHTs might be a direct result of the onset and reversal of the fetal-like reactivation.

For clinical treatment regimen formulation, it is important to determine how fast anthracyclines can enter the cell and nucleus to estimate how much of the adverse effects might be exerted. Likewise, the clearance of anthracyclines from the cardiomyocytes and the recovery time of the cells are crucial factors to consider for determining the degree of cardiotoxicity. We observed a clear difference in the uptake rate of the compounds by cardiomyocytes. Whereas ACLA reached a plateau phase in uptake after only 10 min, DOXO and AMR reached this phase after 5 h. This difference may be of importance in a clinical setting, since treatment with ACLA could result in rapid targeting of cardiomyocytes, while DOXO and AMR would not be taken up by the cardiomyocytes to full saturation if the treatment is performed in a short peak with high dose of the drug. For total drug uptake into cardiomyocytes, a short (<5 h) high dosage may therefore have a similar effect as a long (>5 h) lower peak dosage. We can observe this in our data when comparing the loss of force on EHTs exposed for 3 consecutive days at 1 μM DOXO (Figure 4D), at which point all positive cells have been saturated with DOXO, and the EHTs exposed for only 2 h at 5 μM DOXO (Figure 4H), where all cells have started taking up DOXO, but the maximum saturation has not yet been reached. In both cases, the loss of contraction force on day 3 after start of treatment is similar, despite the higher dose of 5 μM in the 2-h group. Additionally, we found that the loss of contraction force due to 3 consecutive days of exposure to DOXO (Figure 4D) is similar to the loss of force caused by a single day of exposure (Figure 7C), which suggests that repeated dosing before the cell is cleared of DOXO does not lead to a cumulative cardiotoxic effect, but rather adheres to an equilibrium that is dependent on compound concentration in the medium. Therefore, 3 consecutive days of 1 μM DOXO does not correspond to 1 day of 3 μM DOXO. This effect suggests that the peak concentration of DOXO determines the amount of cardiotoxicity, which corresponds well to clinical practice where vulnerable patients are treated with a lower dose for a longer period of time, rather than a higher but shorter peak concentrations.^28^ Interestingly, we observe in our hPSCs that AMR reaches maximum uptake within 10 min after exposure, while in cardiomyocytes this plateau is reached after 5 h, meaning that a short, but high peak dose of AMR could potentially preferentially target proliferative cells and spare the heart.

With our repeated treatment experiment with a recovery time of three weeks, we more closely recreate a clinical treatment regimen. In the clinic, the maximum-tolerable dose of DOXO is often determined by the cumulative dose received by the patient, specifically 450–550 mg/m^2^, despite there being recovery periods in between treatments. This implies that every treatment causes an amount of damage, that, when accumulated, would result in a higher risk of cardiac complications.^28^ In the current timeline of our experiments, we did not observe that repeated doses lead to cumulative cardiac stress over time, but instead we observed an acute cardiotoxic effect after anthracycline treatment with full recovery after 2–3 weeks from initial treatment. Additionally, this model enables a long term assessment of the cardiotoxic effects which could further help identifying possible early- and late-onset chronic effects and verify drug efficacy on the long term.

Although we can recapitulate drug-induced cardiotoxicity, additional parameters need to be considered to emulate the pharmacokinetics of pharmacodynamics of drugs. For example, the 2D and 3D models described here are not fully representative of the human heart. It has been previously suggested that DOXO may not only directly induce cardiotoxicity in cardiomyocytes, but also may exert an effect on heart rhythm via affecting cardiac subtypes such as nodal pacemaker cells and atrial cardiomyocytes^37^^,^^38^ and proliferating cell types of the heart, such as endothelial cells, fibroblasts, smooth muscle cells and others.^39^^,^^40^ Moreover, it has been reported that endothelial dysfunction as a result of DOXO may directly be responsible for the acute cardiotoxic response of the organ, demonstrating the importance of including other cell types of the heart to the platform.^1^

In conclusion, we demonstrate that our hPSC-cardiomyocyte-based platform for toxicity screening enables the early detection of cardiotoxic properties of compounds and recapitulates key aspects of clinically relevant drug treatment regimes *in vitro*, taking into account peak and repeated dosing and recovery time. Furthermore, we were able to clearly discriminate between cardiotoxic mechanisms of the tested anthracyclines.

### Limitations of the study

One limitation of our study is that the results are based on only two hPSC lines. In addition, both lines used have a female background. In the future, we plan to apply the model for assessment of cardiotoxicity in broader patient groups. Another limitation is that we only analyzed one timepoint in our RNA sequencing experiment which was 24 h after drug treatment. Since we observed transcriptional changes 3 days after drug exposure by qPCR, it would be valuable to include this, as well as later timepoints. Moreover, we evaluated tissue recovery at a functional level by contraction force and on transcriptional level by qPCR. Further characterization of tissue recovery using different assays would be of interest.

## STAR★Methods

### Key resources table

**Table:** 

REAGENT or RESOURCE	SOURCE	IDENTIFIER
**Antibodies**		

Anti-phospho-Histone H2A.X (Ser139) Antibody	Merck	05-636
NKX2.5 (E1Y8H)	Cell Signaling Technology	8792S
MEF2C (D80C1) XP	Cell Signaling Technology	5030S
Anti-dHAND Antibody (A-12)	Santa Cruz Biotechnology	sc-398167
Anti-IRX4 Antibody	Sino Biological	104135-T34-100
TBP (D5C9H) XP	Cell Signaling Technology	44059T
Donkey anti-Rabbit IgG (H+L) Highly Cross-Adsorbed Secondary Antibody, Alexa Fluor Plus 647	ThermoFisher Scientific	A32795
Goat anti-Mouse IgG (H+L) Cross-Adsorbed Secondary Antibody, Alexa Fluor 647	ThermoFisher Scientific	A21235
Goat anti-Mouse IgG (H+L) Cross-Adsorbed Secondary Antibody, Alexa Fluor 488	ThermoFisher Scientific	A11001
Anti-phospho-Histone H2A.X (Ser139) Antibody	1:200	Merck (05-636)
NKX2.5 (E1Y8H)	1:400	Cell Signaling Technology (8792S)
MEF2C (D80C1) XP	1:400	Cell Signaling Technology (5030S)
Anti-dHAND Antibody (A-12)	1:200	Santa Cruz Biotechnology (sc-398167)
Anti-IRX4 Antibody	1:200	Sino Biological (104135-T34-100)
TBP (D5C9H) XP	1:400	Cell Signaling Technology (44059T)
Donkey anti-Rabbit IgG (H+L) Highly Cross-Adsorbed Secondary Antibody, Alexa Fluor Plus 647	1:400	ThermoFisher Scientific (A32795)
Goat anti-Mouse IgG (H+L) Cross-Adsorbed Secondary Antibody, Alexa Fluor 647	1:400	ThermoFisher Scientific (A21235)
Goat anti-Mouse IgG (H+L) Cross-Adsorbed Secondary Antibody, Alexa Fluor 488	1:400	ThermoFisher Scientific (A-11001)

**Chemicals, peptides, and recombinant proteins**		

Fluo4-AM calcium dye	ThermoFisher Scientific	F14201
Dimethyl sulfoxide	Sigma-Aldrich	D2650-200
Doxorubicin hydrochloride	Sigma-Aldrich	D1515-10MG
10x TrypLe Select	ThermoFisher	10698118
Calcein-AM LIVE/DEAD™ Viability/Cytotoxicity Kit	ThermoFisher Scientific	L3224
FORMALDEHYDE SOLUTION ACS REAGENT	Sigma-Aldrich	252549-1L

**Critical commercial assays**		

Human Cardiac Troponin I ELISA kit	Abcam	ab200016

**Deposited data**		

RNAseq data	NCBI’s Gene Expression Omnibus (GEO)	GSE232331

**Experimental models: Cell lines**		

LUMC0020iCTRL-06	LUMC	N/A
hESC double Reporter mRubyII-ACTN2 and GFP-NKX2.5 (DRRAGN)	AST	N/A

**Oligonucleotides**		

*NKX2.5* qPCR Fw primer	IDT	fwd: AGCTCTTTCTTTTCGGCTCTAGG
*NKX2.5* qPCR Rv primer	IDT	rev: CCCGCCTTCTATCCACGTG
*MEF2C* qPCR Fw primer	IDT	fwd: CCAACTTCGAGATGCCAGTCT
*MEF2C* qPCR Rv primer	IDT	rev: GTCGATGTGTTACACCAGGAG
*HAND2* qPCR Fw primer	IDT	fwd: ACATCGCCTACCTCATGGAC
*HAND2* qPCR Rv primer	IDT	rev: TGGTTTTCTTGTCGTTGCTG
*IRX4* qPCR Fw primer	IDT	fwd: ATGCTTCAGGGTATCTGGCCTCTT
*IRX4* qPCR Rv primer	IDT	rev: TTGGACTCCTGGGAACATGGACAA
*CACNA1* qPCR Fw primer	IDT	fwd: CAATCTCCGAAGAGGGGTTT
*CACNA1* qPCR Rv primer	IDT	rev: TCGCTTCAGACATTCCAGGT
*HCN4* qPCR Fw primer	IDT	fwd: CAATGAGGTGCTGGAGGAGT
*HCN4* qPCR Rv primer	IDT	rev: GGTCGTGCTGGACTTTGTG
*GJA1* qPCR Fw primer	IDT	fwd: GGTGACTGGAGCGCCTTAG
*GJA1* qPCR Rv primer	IDT	rev: GCGCACATGAGAGATTGGGA
*ACTN2* qPCR Fw primer	IDT	fwd: CTGCTGCTTTGGTGTCAGAG
*ACTN2* qPCR Rv primer	IDT	rev: TTCCTATGGGGTCATCCTTG
*SERCA* qPCR Fw primer	IDT	fwd: GTCACTCCACTTCCTGATCC
*SERCA* qPCR Rv primer	IDT	rev: CTCCAGTATTGCAGGTTCCA
*CAV1.3* qPCR Fw primer	IDT	fwd: ATACCCAGGCAGAAACATCG
*CAV1.3* qPCR Rv primer	IDT	rev: TCATCGTCCTCCAAGAATCC
*hRPLPO* qPCR Fw primer	IDT	*fwd: CACCATTGAAATCCTGAGTGATGT*
*hRPLPO* qPCR Rv primer	IDT	*rev: TGACCAGCCCAAAGGAGAAG*

**Software and algorithms**		

FlowLogic software	Inivai Technologies	N/A
Custom software for calcium analysis	AST	N/A
Automated Sarcomere Structure Analysis	Lu Cao	Lu Cao, Linde Schoenmaker, Simone A Ten Den, Robert Passier, Verena Schwach, Fons J Verbeek, Automated Sarcomere Structure Analysis for Studying Cardiotoxicity in Human Pluripotent Stem Cell-Derived Cardiomyocytes, *Microscopy and Microanalysis*, Volume 29, Issue 1, February 2023, Pages 254–264

### Resource availability

#### Lead contact

Further information and requests for resources and reagents should be directed to and will be fulfilled by the lead contact, Robert Passier (robert.passier@utwente.nl).

#### Materials availability

This study did not generate new unique reagents.

#### Data and code availability

The RNA-sequencing data discussed in this publication are available at the NCBI’s Gene Expression Omnibus (GEO) under GEO accession number GSE232331. Additional data reported in this paper will be shared by the lead contact upon request.

All original code is available upon request.

Any additional information required to re-analyse the data reported in this paper is available from the lead contact upon request.

### Experimental model and study participant details

#### HPSC culture and cardiac differentiation

HiPSCs (LUMC0020iCTRL-06, female) and hESC double Reporter mRubyII-ACTN2 and GFP-NKX2.5 DRRAGN, female) were maintained in Essential 8 medium on vitronectin (5 μg/mL) (Thermo Fisher)^14^ maintained in a humidified incubator at 37°C and 5% CO_2_. Cardiomyocytes were differentiated as described previously. At day 13 of differentiation, hiPSC-cardiomyocytes were purified in glucose-free-lactate-containing medium for 4 days. The fraction of Troponin-T positive cardiomyocytes was assessed by flow cytometry after staining with Cardiac Troponin T Antibody-VioBlue (Miltenyi Biotech) on a MACSQuant VYB flow cytometer (Miltenyi Biotech) at ex405nm, em450/50nm. Plots were analyzed with FlowLogic software. DRRAGN-cardiomyocytes were dissociated around day 14 using 10x TrypLe Select (Thermo Fisher) and were FACS sorted for double expression of actinin^mRybyII/w^-NKX2.5^eGFP/w^ on a Sony SH800S Cell Sorter (Sony Biotechnology) (mRubyII at ex561nm, em617/30nm. After purification, all cardiomyocytes were cultured in cardiomyocyte-TDI medium.^1^ Stem cells and cardiomyocytes were maintained in a humidified incubator at 37°C and 5% CO_2_ and were refreshed daily with E8 medium, or with cardiomyocyte-TDI medium twice a week, respectively. Approximately 10 days after seeding, cardiomyocytes were treated with dimethylsulfoxide (DMSO 4.23 mM) as control, DOXO or its analogues.

### Method details

#### Anthracycline uptake measurements

HPSC-cardiomyocytes were seeded as full monolayers (30 K cells per well) onto vitronectin-coated CellCarrier-96 well special optics plates (PerkinElmer) and were treated with 1 μM of the anticancer drugs DOXO, AMR or ACLA 10-12 days after seeding. Images of hPSC-cardiomyocytes were acquired using the high-throughput automated EVOS FL Auto 2 (Thermo Fisher) microscope equipped with a 40x Super-apochromat Olympus objective (NA 0.95) (Thermo Fisher, AMEP4754). Anthracyclines were quantified based on auto-fluorescence using the RFP filter cube (ex531/20nm; em593/40nm, Thermo Fisher, AMEP4652). The cell culture area of the well was scanned by automatically acquiring 55 images per well at indicated timepoints. Cells were maintained during the 24 hours at 37°C and 5% CO_2_ on the EVOS FL auto 2 with the EVOS Onstage incubator (Thermo Fisher). For flow cytometry quantification, cardiomyocytes were treated in suspension with the compounds and autofluorescence was quantified (at ex561nm, em586/15) with a MACSQuant VYB flow cytometer (Miltenyi Biotech) at the indicated time points. Plots were analyzed with FlowLogic software.

#### Calcein imaging

HiPSC-cardiomyocytes cultured in monolayer in 96 well culture plates for imaging (CellCarrier-96, PerkinElmer) at a density of 30 K cells per well were exposed to 24 hours of anthracyclines at indicated concentrations of the compound. Calcein AM (ThermoFisher) live cell dye in PBS at a final concentration of 4 μM was added to the cells in the well after washing the well 1x with DPBS(-). The dye was incubated with the cells for 30 minutes at 37°C inside a CO_2_ incubator, followed by microscopic analysis using the EVOS auto II (ThermoFisher) imaging system. Keeping the acquisition settings constant, the Calcein AM fluorescent signal was captured using the GFP filter cube (ThermoFisher, AMEP4951, ex482/25nm; em524/24nm), making 12 images per culture well using a 10x Plan fluorite (NA 0.25) objective (ThermoFisher, AMEP4681). Calcein signal as a fraction of total surface area was then assessed by a custom ImageJ macro, selecting the Calcein signal using a hard (manual) threshold set constant per replicate batch, of which the area was divided over the total area of the image. This fraction represented the area covered by live cells, thus viability of the hPSC-cardiomyocytes.

#### Time lapse live imaging

After purification by FACS, the hESC-cardiomyocytes were immediately re-plated as monolayers onto vitronectin-coated CellCarrier-96 well special optics plates (PerkinElmer) in TDI medium for viability and myofibril integrity assessment. HPSC-cardiomyocytes were treated with dimethylsulfoxide (DMSO 4.23 mM) as control or with a single dose of 1 or 5 μM of the anthracycline DOXO, or 1 μM AMR or ACLA 10-12 days after seeding.

Images of hPSC-cardiomyocytes were acquired using the high-throughput automated EVOS FL Auto 2 (Thermo Fisher) microscope equipped with a 40x Super-apochromat Olympus objective (NA 0.95) (Thermo Fisher, AMEP4754). Fusion protein α-actinin_mRubyII was acquired using the RFP filter cube (Thermo Fisher, AMEP4652 ex531/20nm; em593/40nm). Cytosolic GFP expression was used as a proxy for cell viability, and was acquired using the GFP filter (ThermoFisher, AMEP4951, ex482/25nm; em524/24nm). The cell culture area of the well was scanned by automatically acquiring 55 images per well every 24 hours for 4 days. Cells were maintained during the 4 days at 37°C and 5% CO_2_ on the EVOS FL auto 2 with the EVOS Onstage incubator (Thermo Fisher). GFP signal as a fraction of total surface area was then assessed by a custom ImageJ macro, selecting the GFP signal using a hard (manual) threshold set constant per replicate batch, of which the area was divided over the total area of the image. This fraction represented the area covered by live cells, thus viability of the hPSC-cardiomyocytes.

#### Image analysis for myofibril integrity assessment

Two image preprocessing steps were performed, including background subtraction (ImageJ, Subtract Background, 50 for α-actinin channel) and contrast enhancement (ImageJ, Enhance Contrast, Saturated pixels: 0.3% for α-actinin channel). 2-dimensional fast Fourier transformation (2DFFT) is applied and a customized bandpass filter is used to filter out low and high frequency signals and only reserve the range of frequency that is representative to the range of sarcomere length (1.2-2 μm).^41^ Inverse 2DFFT is conducted to retrieve the fluorescent signal that is in the pre-defined frequency range. Finally, a hard thresholding (threshold=21 for 8-bit image) is used to filter out trivial signals and to acquire the binary mask representing organized sarcomere structure as depicted.^42^

#### ELISA

Troponin-I release was measured from hiPSC-cardiomyocytes seeded in monolayer onto vitronectin-coated CellCarrier-96 well plates. Compounds were added to the culture 10 days after seeding, and were left without refreshing for 4 consecutive days. Then, media of two culture wells with the same condition were pooled and stored at -20°C until measurement. Free TnI was quantified using the Human Cardiac Troponin I ELISA kit (Abcam) according to manufacturer’s instructions. Troponin quantity was determined using a Tecan Infinite 200 PRO plate reader using spectrometry at OD 450 nm.

#### Calcium imaging

HiPSC-cardiomyocytes were seeded as monolayers onto vitronectin-coated CellCarrier-96 well special optics plates (PerkinElmer) in TDI medium and treated with the anthracyclines 10-12 days after seeding at the indicated concentrations. To quantify calcium kinetics and accumulation, cardiomyocytes were stained with the Fluo4-AM calcium dye (Thermo Fisher Scientific) at a final concentration of 1 μM with Pluronic (0.2 mg/ml) for 30 min at 37°C. Imaging was performed 24 hours or 2 weeks after treatment at 37°C and 5% CO_2_ on a Nikon Eclipse TE2000-U Microscope with a 20x objective. Videos were taken at 70 fps, which were used to quantify Ca^2+^ fluxes into and out of the cell over time using a custom software script. The individual frame at the lowest fluorescent intensity (= complete relaxation of the cells) was used to determine the baseline Ca^2+^ load of the cardiomyocytes after drug treatment by measuring mean fluorescent intensity of the entire frame using ImageJ software.

#### Contraction parameters in EHTs

EHTs were generated as described previously^43^ and cultured in cardiomyocyte medium.^44^ Force of contraction (FoC) and contraction kinetics were recorded 10-12 days after tissue formation. The next day, a second force measurement was performed and treatment was started. To study a cumulative drug dose tissues were refreshed daily. Fresh DOXO, AMR or ACLA medium was prepared for each daily treatment. FoC and contraction kinetics were measured every 12 hours for 72 hours. During measurements tissues were paced at 2 Hz. For short term exposure experiments, tissues at day 10-12 after tissue formation were refreshed with 5 μM DOXO, AMR or ACLA for 2 hours, after which medium was fully changed to culture medium. FoC and contraction kinetics were measured every 24 hours for 72 hours. To study cardiotoxicity recovery, EHTs were treated at day 10 or day 21 with fresh 1 μM DOXO, AMR or ACLA or DMSO by fully replacing the culture media. After 24 hours, tissues were fully refreshed by placing them to a new well containing media.

#### Immunocytochemistry

Monolayer hPSC-cardiomyocytes were exposed to anthracyclines equal to the Calcein live cell quantification but were fixated in the culture plate with 4% formaldehyde for 15 minutes, after the anthracycline exposure was completed. After fixation, plates were stored at 4°C until immunostaining was performed. For this, cells were permeabilized with 0,1% Triton-x100 for 8 minutes at RT, followed by blocking with 10% normal goat serum + 2% BSA in PBS for 1 hour at RT. Next, primary antibodies were added in 2% BSA in PBS buffer, and incubated for 1,5 hours at RT, or overnight at 4°C. Secondary antibody was added after 3x washing with PBS in a 2% BSA in PBS buffer, and incubated for 1 hour at RT. Finally, the secondary antibody solution was washed off by 3x rinse in PBS of 5 minutes each, followed by a DAPI counterstain for 5 minutes at RT. Plates we kept at 4°C in PBS for microscopic analysis and further storage. A list of antibodies with their respective dilutions is provided in the key resources table.

#### DNA damage quantification

2D monolayers treated for 24 hours with DMSO, DOXO, AMR or ACLA were stained with Anti-phospho-Histone H2AX as described above, to quantify the level of double-stranded DNA damage.^45^ DAPI was used to determine the total number of nuclei per picture. Quantification of the percentage of positive nuclei for gH2AX and the average number of dots of gH2AX per nuclei was determined with a custom made script developed in CellProfiler™, using both gH2AX and DAPI images.

#### Mitochondrial functional assay

EHTs were harvested 24 hours after treatment with 5 μM DOXO, ACLA, AMR or DMSO (corresponding to day 11 after EHT formation) and preserved in ice-cold preservation solution (BIOPS solution), containing (in mM) K2EGTA (7.2), CaK2EGTA (2.8), ATP (5.8), MgCl2 (6.6), taurine (20), phosphocreatine (15), imidazole (20), dithiothreitol (0.5) and 2-(N-morpholino)ethanesulfonic acid (MES; 50); pH 7.1 adjusted with KOH. EHTs were permeabilized using saponin (50 μg/mL) in ice-cold preservation solution for 25 minutes and subsequently washed twice for 10 minutes in ice-cold mitochondrial respiration medium (MiR05), containing (in mM) EGTA (0.5), MgCl2 (3), potassium lactobionate (60), taurine (20), KH2PO4 (10) HEPES (20), sucrose (110) and 1 g/L fatty acid free bovine serum albumin, pH 7.1 adjusted with KOH. Per treatment condition 2 or 3 EHTs were pooled and inserted into a measurement chamber of a high-resolution respirometer (Oxygraph-2k; Oroboros Instruments, Innsbruck, Austria). All experimental protocols were carried out at 37°C under oxygen levels above 300 μM throughout the experiment to avoid oxygen supply limitations. Leak respiration was assessed in the presence of 10 mM sodium glutamate, 2 mM sodium malate and 5 mM sodium pyruvate, feeding electrons via complex I. Maximal complex I-linked respiration was determined upon adding 5 mM ADP. Cytochrome-c (10 μM) was added to evaluate outer mitochondrial membrane integrity. Maximal OXPHOS capacity, with maximum electron input via complexes I and II was stimulated by adding 10 mM succinate. Excess capacity of the electron transferring complexes (I – IV) was determined by titrating a protonophore (carbonyl cyanide p-trifluoro-methoxyphenyl hydrazine; FCCP) in 0.05 μM steps. Complex II-linked respiration was measured after blocking complex I with 0.5 μM rotenone. Antimycin-A (2.5 μM) was added to completely inhibit mitochondrial oxygen consumption and measure residual oxygen consumption, which was subtracted from all values as background. Oxygen flux was normalized to the amount of cells that was used to generate EHTs.

#### Transcriptional analysis

For RNAseq, RNA was isolated from EHTs 24 hours or 21 days after treatment using the Nucleospin mini kit (Macherey-nagel, 740955.250). Libraries and sorted samples were generated from 200 ng of RNA and processed as described in.^2^ Differential gene expression (Wald test) between conditions was performed with the R package DESeq2 (v1.34.0).^3^ The genes with an absolute log2 fold change >1.0 and false discovery rate (FDR) p < 0.05 were considered differentially expressed and visualized with pheatmap (v1.0.12) and EnhancedVolcano (v1.12.0). Log-scaled normalized counts were used to compare the gene expression levels between samples. For qPCR, RNA was isolated from EHTs 72 hours after treatment using the Nucleospin mini kit (Macherey-nagel, 740955.250). CDNA was synthesized using the iScript cDNA synthesis kit (Bio-Rad, 1708891) and qPCR was carried out using SensiMix SYBRGreen (Meridian Bioscience, QT615-05) on the CFX384 real-time PCR detection system (Bio-Rad). Primer sequences are provided in the key resources table.

#### Statistics

Statistical analysis was carried out with GraphPad Prism 9. Ordinary one-way ANOVA with Tukey’s multiple comparisons test was applied for differences in means between groups. Data are expressed as means ± s.e.m.. Statistical significance was defined as p < 0.05. All the statistical details of each experiment can be found in the figure legends.
