# Supplementary material for: A safety screening platform for individualized cardiotoxicity assessment
Source: iScience. 2024 Feb 6;27(3):109139. doi: 10.1016/j.isci.2024.109139 (PMC10879698; doi:10.1016/j.isci.2024.109139)
Supplement: Document S1. Figures S1–S9 and Tables S1 and S2 [file mmc1.pdf]

iScience, Volume *27*

Supplemental information

A safety screening platform for individualized cardiotoxicity assessment

Verena Schwach, Rolf H. Slaats, Carla Cofiño-Fabres, Simone A. ten Den, José M. Rivera-Arbeláez, Maureen Dannenberg, Chiara van Boheemen, Marcelo C. Ribeiro, Sabina Y. van der Zanden, Edgar E. Nollet, Jolanda van der Velden, Jacques Neefjes, Lu Cao, and Robert Passier

Supplementary Material

List of Supplementary material

1) **Supplementary figures and tables**

Supplemental item titles

Figure S1: DOXO uptake and removal in hPSC-cardiomyocytes and DOXO treatment of hPSC: cytotoxicity test, related to Figure 1
Figure S2: Quantification of force of contraction (FoC), contraction velocity (VEL) and relaxation VEL every 12 hours for up to 72 hours after drug exposure in hiPSC-EHTs, related to Figure 4.
Figure S3: gH2AX immunolabelling of hPSC-cardiomyocytes in monolayer cultures treated with 10 or 20 µM AMR or ACLA, respectively, related to Figure 5
Figure S4: Additional plots of mitochondrial function in hiPSC-EHTs after treatment for 24 hours with DMSO, 5 µM DOXO, ACLA or AMR, related to Figure 5.
Figure S5: Transcription factor immunostainings, related to Figure 5
Figure S6: Time lapse of NKX2.5 depletion in hiPSC-cardiomyocyte monolayers upon exposure to 1 or 10 µM DOXO, related to Figure 5., related to Figure 5
Figure S7: Bulk RNA sequencing on hiPSC-EHTs, related to Figure 6
Figure S8: Sarcomere recovery in monolayer and 3 week Time lapse of Transcription factor NKX2.5, related to Figure 7
Figure S9: Gene expression recovery in hiPSC-EHTs, related to Figure 7

Table S1: RNAseq_VennDiagram_Genes and TFs related to figure 6
Table S2: RNAseq_GeneOntology_Treatments_vs_CT related to figure 6

1) Supplementary figures

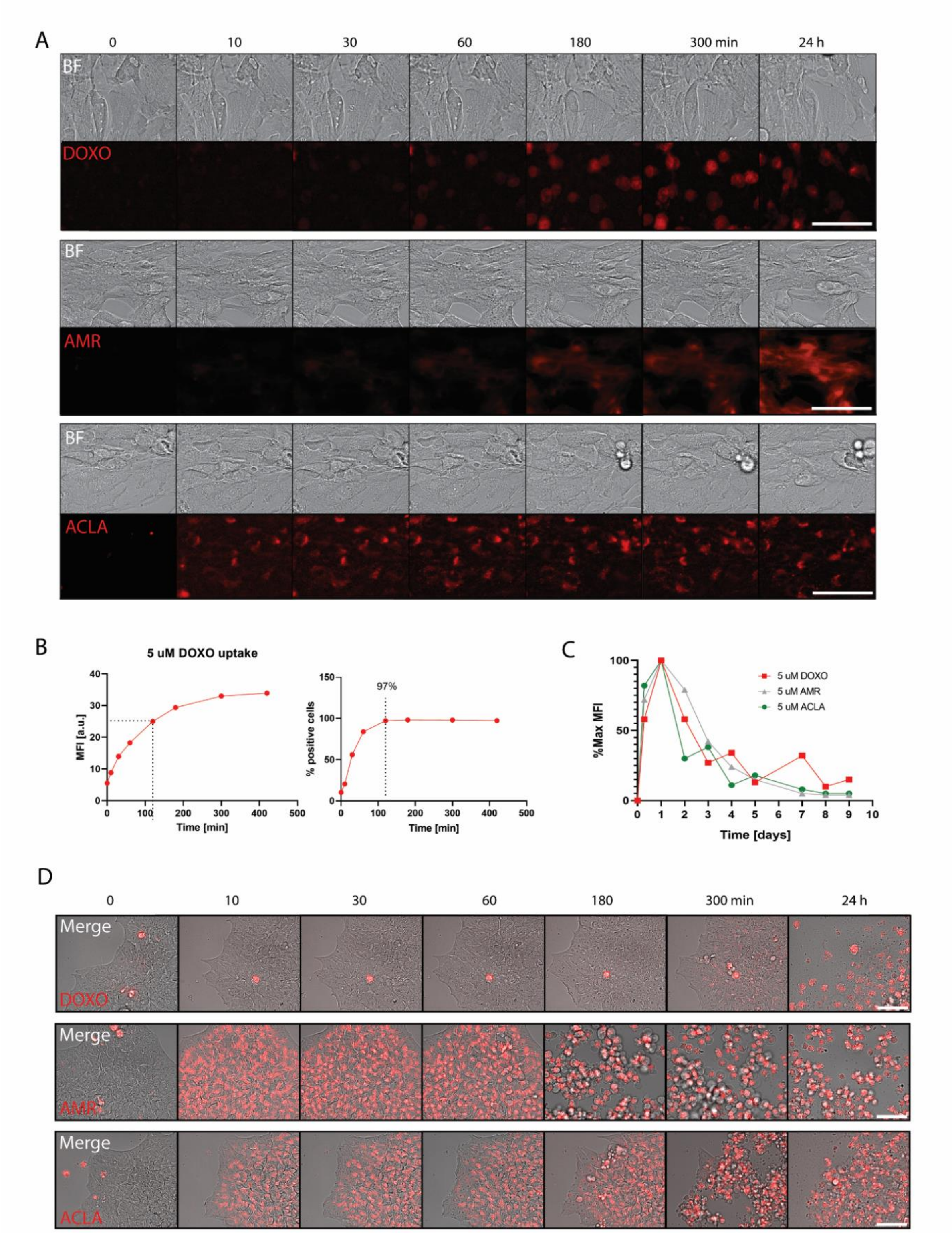

Figure S1: DOXO uptake and removal in hPSC-cardiomyocytes and DOXO treatment of hPSC: cytotoxicity test related to figure 1. *A) Representative overlay of auto-fluorescent signal of the compounds acquired at ex542/20 nm and em593/40 nm on brightfield micrograph during 24 hour incubation with 1 µM DOXO, AMR or ACLA. AMR and ACLA uptake can be observed as early as 10 minutes after start of the incubation, while fluorescent signal of DOXO and AMR gradually increase intensity up to 180 minutes after start of the treatment. Scale bar = 75 µm. B) Uptake of DOXO at 5 µM, (n=1). C) Quantification of the loss of fluorescent signal after washout of the drug at the 24-hour timepoint. Data is normalized to max intensity of the drug (at 24 hours), n=1. D) Cytotoxicity screen of anthracyclines on hPSCs over 24 hours at 1 µM concentration. HPSCs are organized in a colony at the start of exposure, but deteriorate upon incubation with the compounds, ultimately leading to total cell loss 24 hours after onset of exposure Scale bar = 75 µm.*

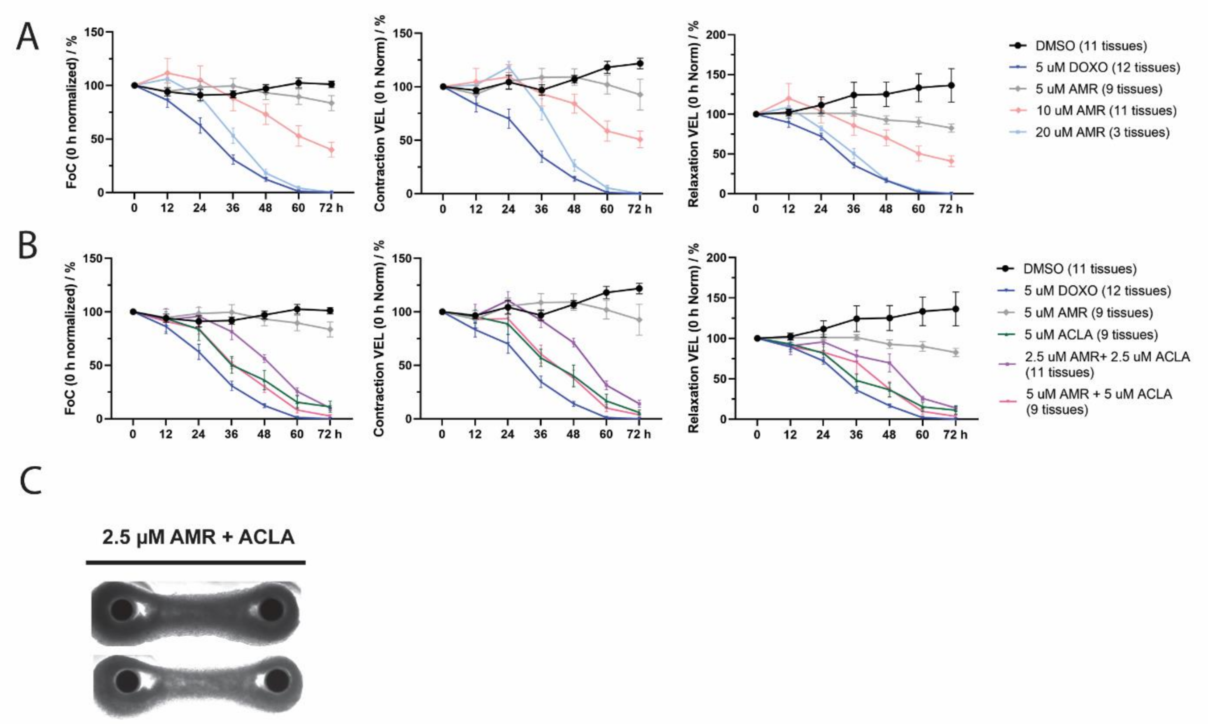

Figure S2: Quantification of force of contraction (FoC), contraction velocity (VEL) and relaxation VEL every 12 hours for up to 72 hours after drug exposure in hiPSC-EHTs, related to Figure 4. *Data was normalized to timepoint 0 of the experiment. Data plotted as percentage means ± s.e.m., statistically tested by Two-Way-ANOVA, * marks statistical significant difference to DMSO control, unless indicated otherwise (n=9-11 EHTs per condition, from 5 cardiomyocyte differentiations).*

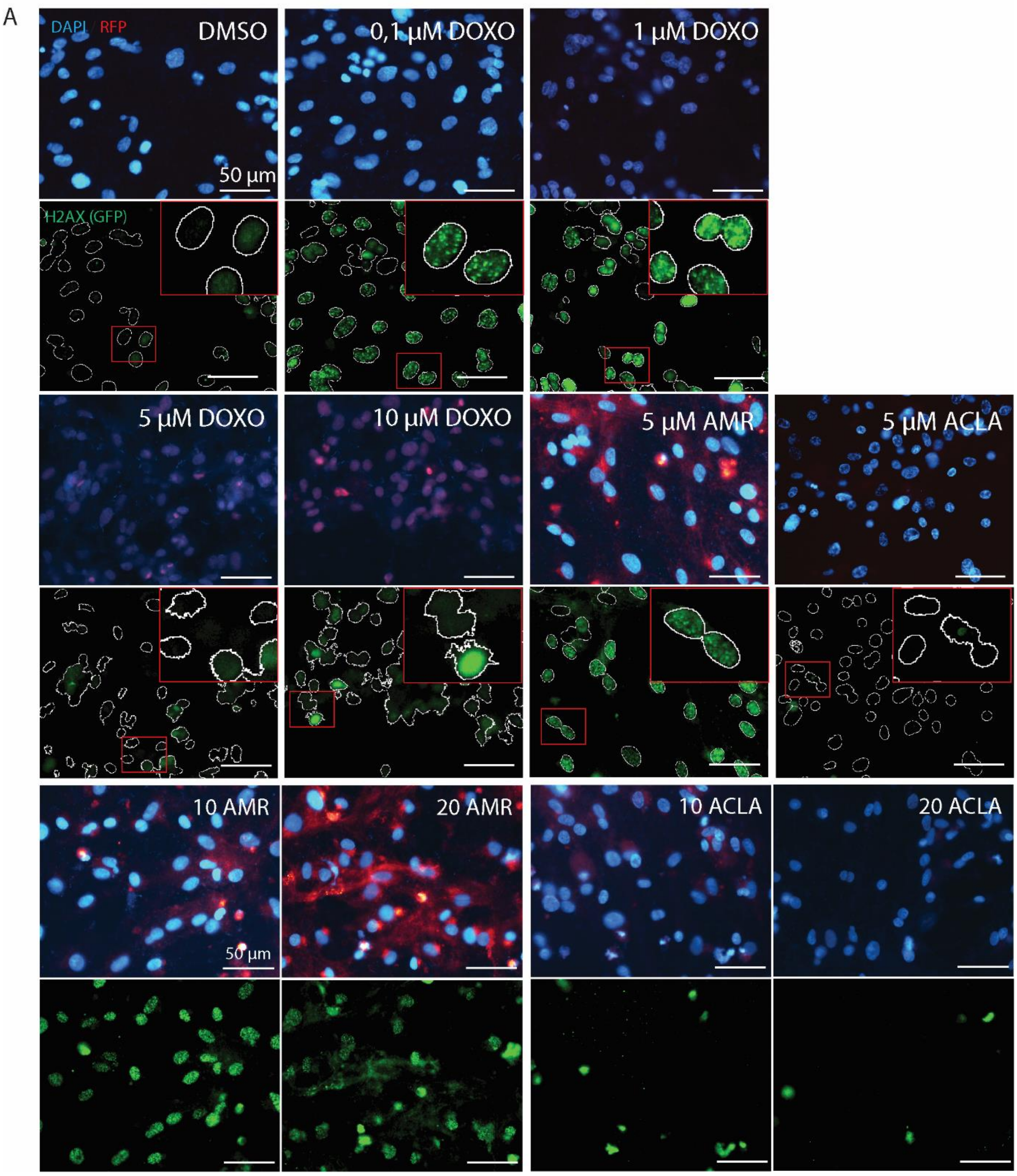

Figure S3: gH2AX immunolabelling of hPSC-cardiomyocytes in monolayer cultures treated with 10 or 20 µM AMR or ACLA, respectively, related to Figure 5. *At both concentrations, AMR clearly induces numerous DSBs in the nuclei of the cardiomyocytes, in contrast to ACLA, which does not induce more with increasing concentrations. Scale bar = 50 µm.*

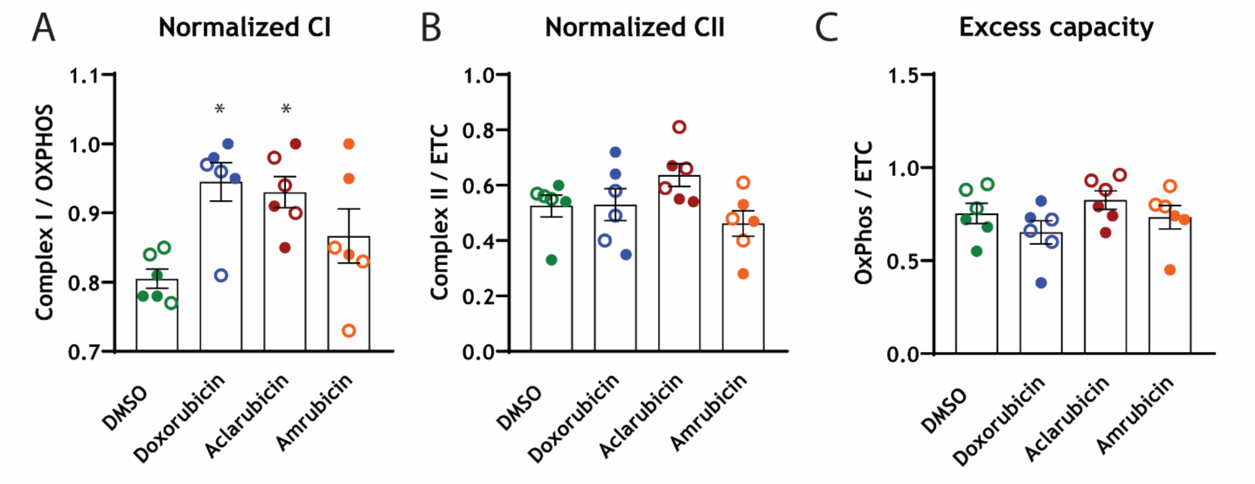

Figure S4: Additional plots of mitochondrial function in hiPSC-EHTs after treatment for 24 hours with DMSO, 5 µM DOXO, ACLA or AMR, related to Figure 5. *A) Normalized complex I-linked respiration to total OxPhos; B) normalized complex II-linked respiration to Electron Transfer Chain capacity (ETC); C) Excess capacity determined by the ratio of OxPhos to ETC capacity. Data plotted as means ± s.e.m., statistically tested by One-Way-ANOVA, * marks statistically significant difference to DMSO control, unless indicated otherwise (n=6 EHTs per condition, from 6 cardiomyocyte differentiations).*

A

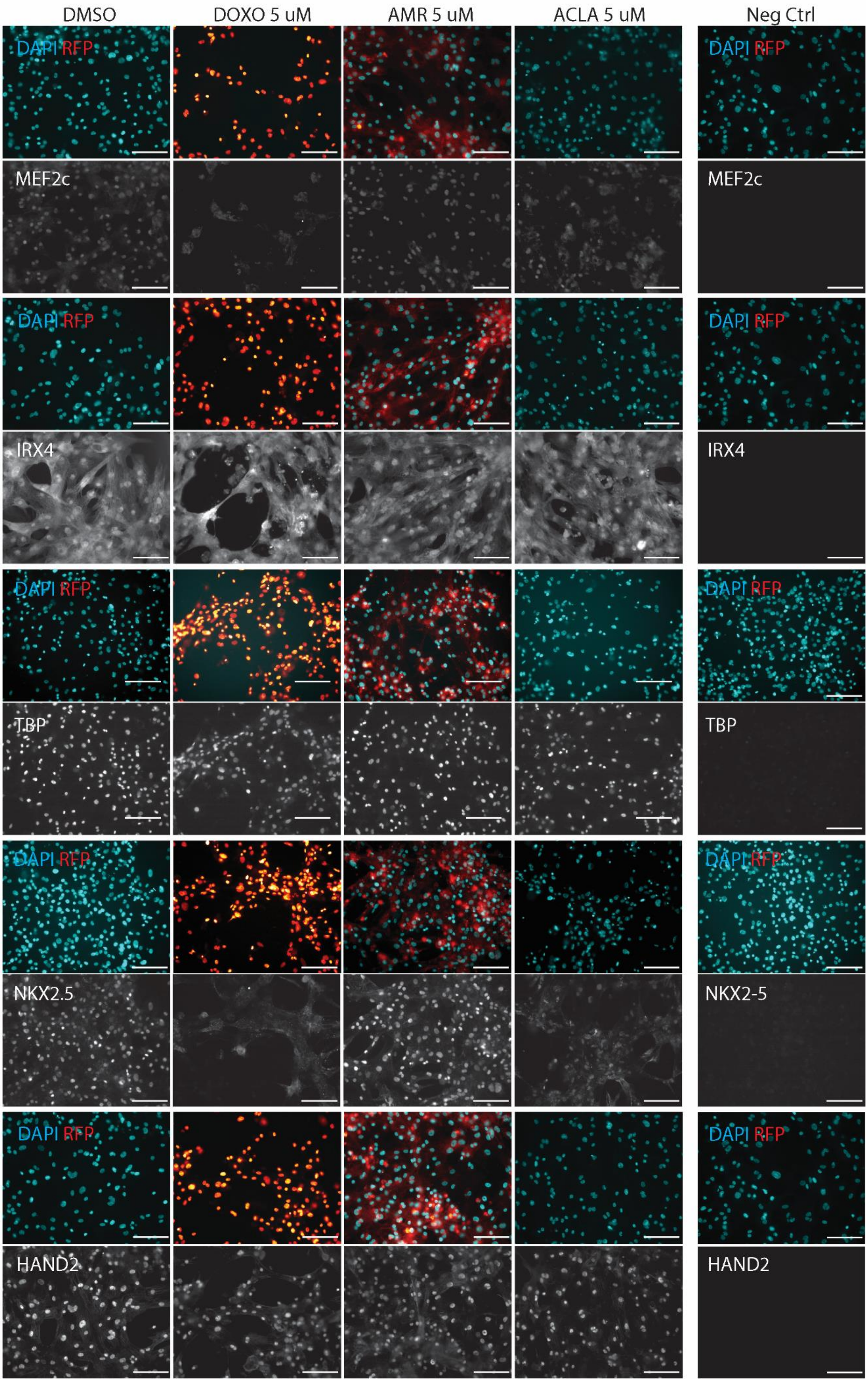

DMSO
DOXO 5 uM
AMR 5 uM
ACLA 5 uM
Neg Ctrl
DAPI RFP
MEF2c
IRX4
TBP
NKX2.5
NKX2-5
HAND2

Figure S5: Transcription factor immunostainings, related to Figure 5. *A) Transcription factor TBP, not exclusive to cardiomyocytes, and IRX4, partly expressed in the cytoplasm, remain detectable after 24 hours of exposure to DOXO, AMR or ACLA, in contrast to MEF2c, which is not detectable after DOXO or ACLA treatment in hiPSC-cardiomyocyte monolayers. Scale bar = 100 µm.*

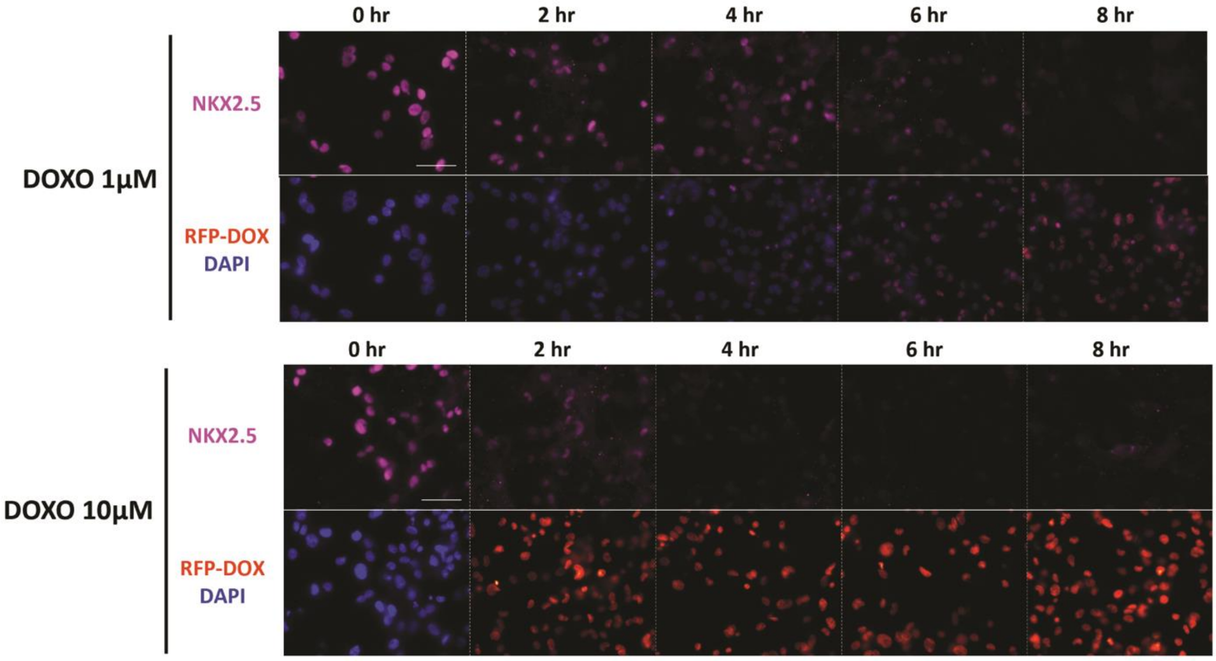

Figure S6: Time lapse of NKX2.5 depletion in hiPSC-cardiomyocyte monolayers upon exposure to 1 or 10 µM DOXO, related to Figure 5. *NKX2.5 can be seen fading gradually over the time course of 8 hours when cells were subjected to 1 µM DOXO, while the transcription factor all but disappeared in mere 4 hours when cells were incubated in 10 µM DOXO. Scale bar = 100 µm*

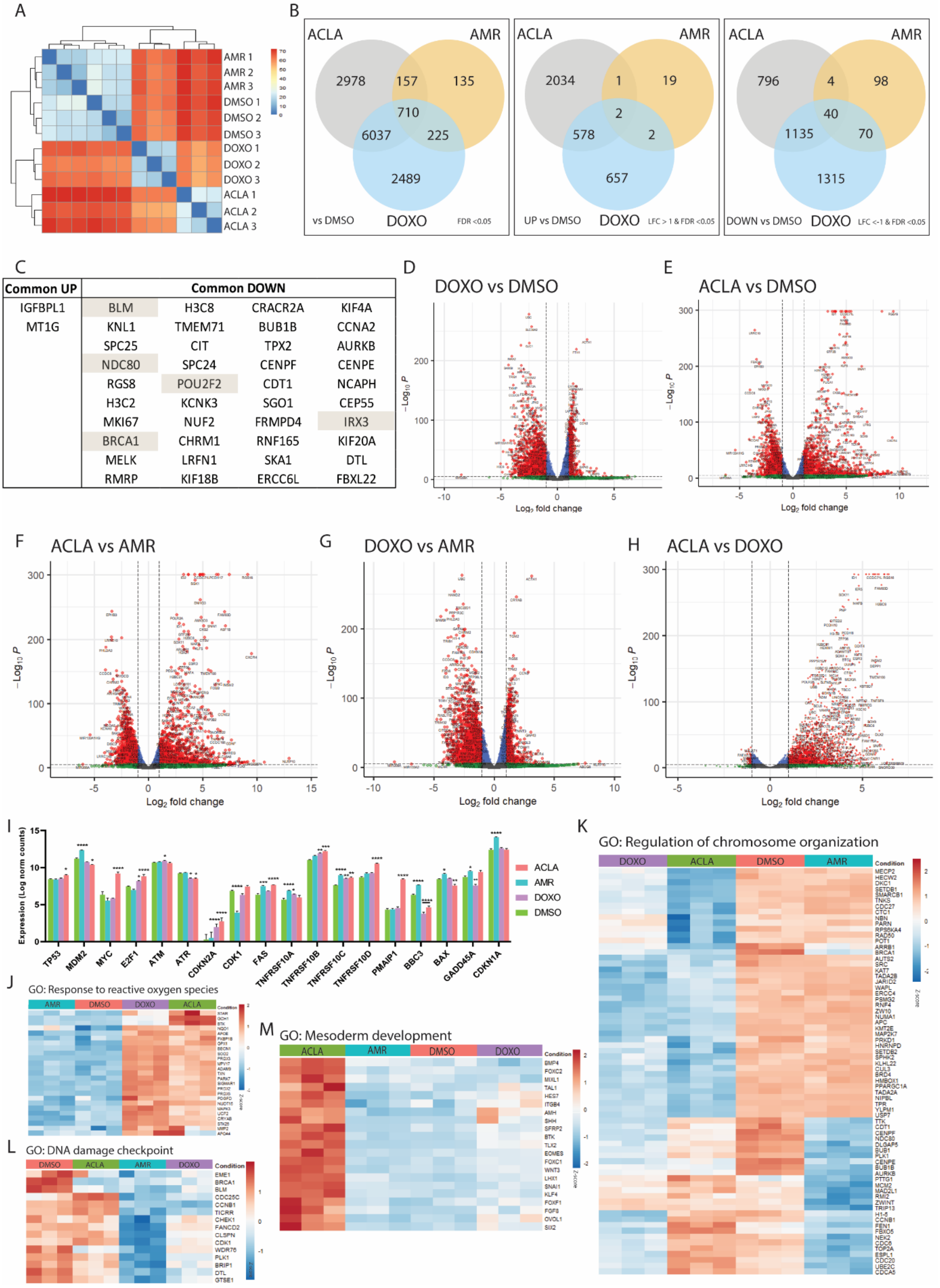

Figure S7: Bulk RNA sequencing on hiPSC-EHTs, related to Figure 6. *A) Spearman's correlation heatmap of hPSC-EHTs treated with DMSO, DOXO, ACLA or AMR for 24 hours. B) Venn Diagrams of genes up and down regulated (left), only upregulated (middle) or only downregulated (right) compared to DMSO. C) Shared genes among DOXO, ACLA and AMR compared to control from Venn Diagrams. Colored boxes indicate transcription factors. D-H) Volcano plot displaying log2-fold-change and adjusted p value genes from DOXO vs DMSO D), ACLA vs DMSO E), ACLA vs AMR F), DOXO vs AMR G) and ACLA vs DOXO H). I) Mean log expression of selected genes involved in regulation of apoptosis and cell cycle. J-M): Heatmap of selected gene*

ontology terms: Response to reactive oxygen species (J), Regulation of chromosome organization (K), DNA damage checkpoint (L) and Mesoderm development (M). n=3 cardiomyocyte differentiations, each with 3 EHTs per sample).

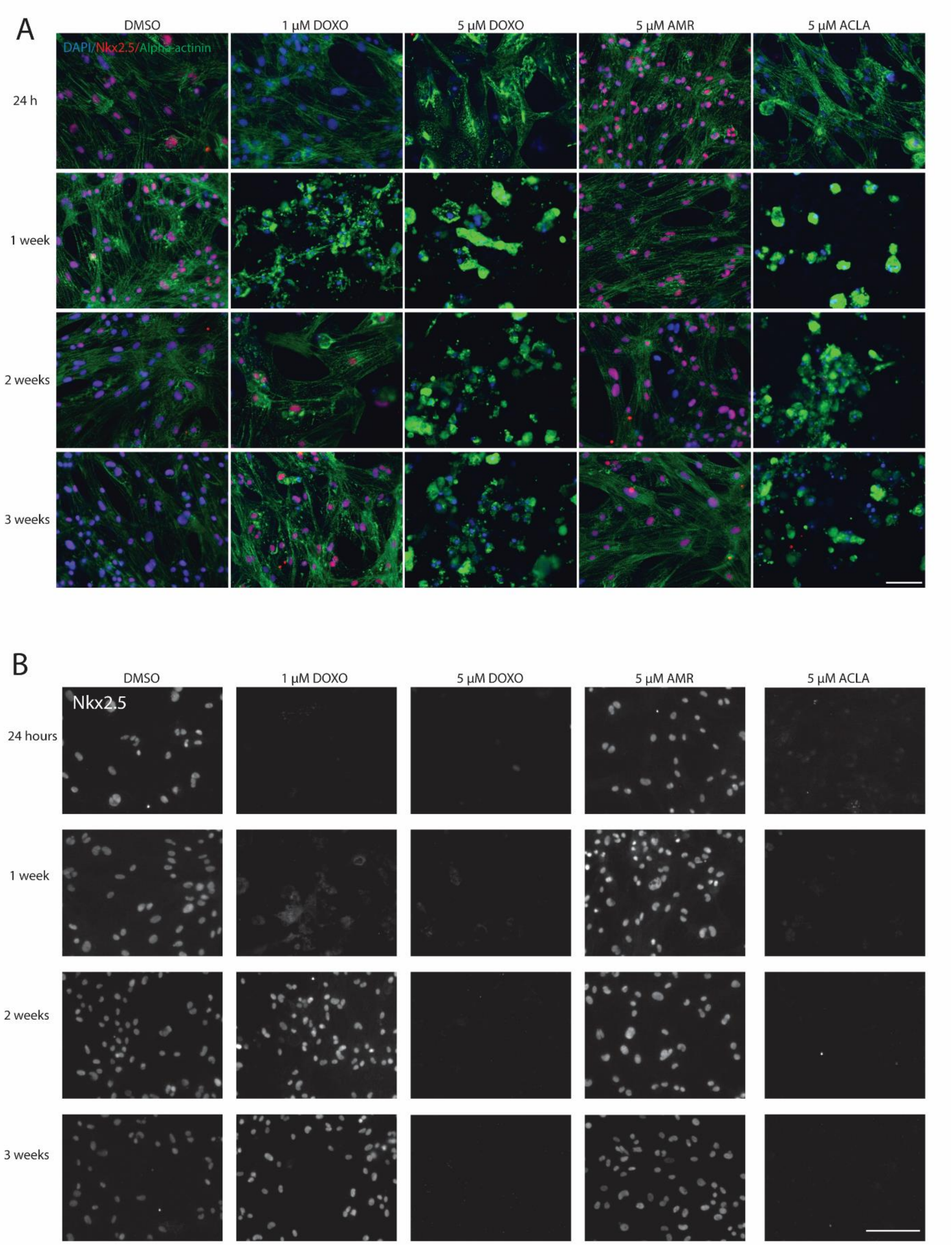

Figure S8: Sarcomere recovery in monolayer and 3 week Time lapse of Transcription factor NKX2.5, related to Figure 7. *A) Immunostaining for sarcomeric alpha-actinin reveals the loss of sarcomeric integrity upon 24 hours treatment with DOXO, and ACLA in cardiomyocyte monolayers. After treatment with 1 µM DOXO, the cardiomyocytes were able to recover over the subsequent culture period of 3 weeks, however 5 µM DOXO and ACLA treatment irreversibly disrupted and could not recover*

in the full duration of the experiment. Scale bar = 50 µm. B) Immunofluorescent staining of transcription factor NKX2.5 on hPSC-cardiomyocyte monolayer cultures treated for 24 hours with DMSO, 1 or 5 µM DOXO, 5 µM AMR or 5 µM ACLA and fixed for analysis 24 hours, 1 week, 2 weeks or 3 weeks post treatment. DMSO and AMR could not abolish NKX2.5 presence in the nucleus during the entire experiment, while 5 µM DOXO and ACLA depleted the transcription factor without recovery. Only 1 µM DOXO initially lowered NKX2.5 expression in the nucleus temporarily, and expression was restored upon continued culture for two weeks or more.

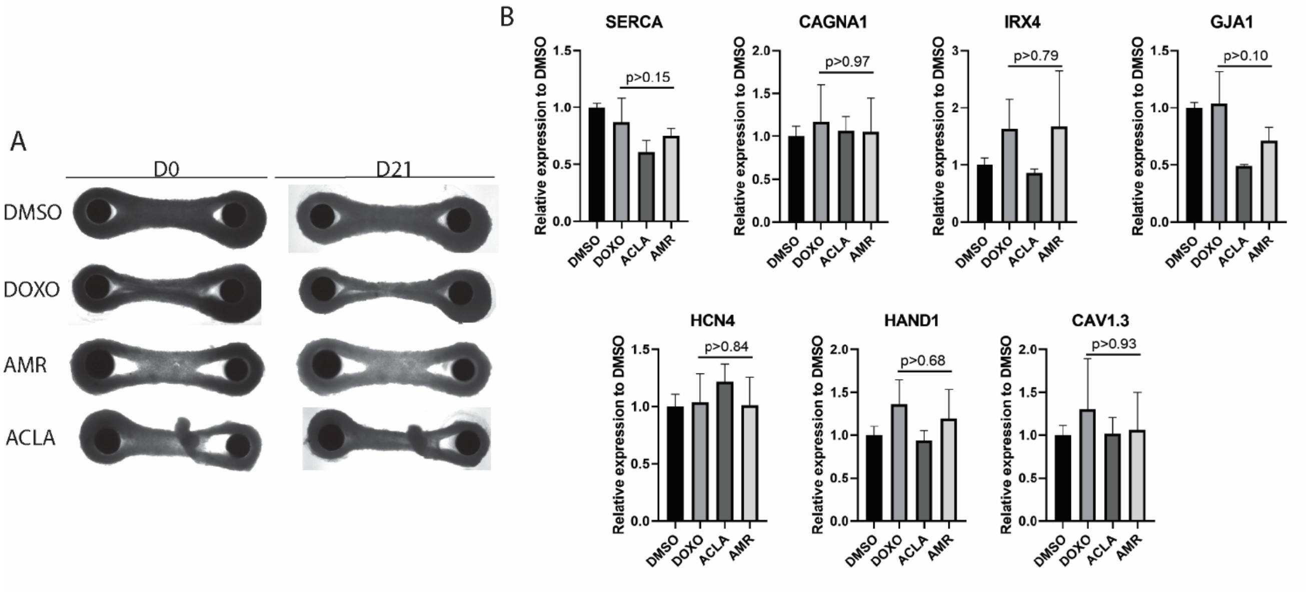

Figure S9: Gene expression recovery in hiPSC-EHTs, related to Figure 7. *A) Representative micrographs of hiPSC-EHTs before (D0) and after 21 days (D21) from the initial treatment with DMSO, 1 µM DOXO, ACLA or AMR at day 0 for 24 hours. Tissue integrity was maintained from D0 to D21. B) Additional gene expression analysis of selected cardiac genes by RT-qPCR in hiPSC-EHTs after 21 days of exposure to DMSO, 1 µM DOXO, AMR or ACLA (n=3 cardiomyocyte differentiations, each with 1-2 EHTs per sample). Data plotted as means ± s.e.m., statistically tested by One-Way-ANOVA*

Table S1: RNAseq_VennDiagram_Genes and TFs, related to Figure 6

Upregulated DEGs identified from VennDiagram. Genes upregulated in ACLA/DOXO/AMR compared to DMSO (control). Cuttof: LogFoldChange >1 & FDR <0.05

ACLA.DOXO.A	ACLA.AMR	AMR.DOXO	AMR	ACLA					DOXO	
IGFBPL1	FAS	GLS2	SPATA18	RGS2	LOC100134868	FTH1P3	SHISA3	SERPINE2	HCN2	PPP1CB
MT1G		FGB	GDF15	LHX1	LRRC37A3	RNF139-AS1	HES7	RAPSN	SLC34A1	MGAM
			ANK1	SCRT2	NECTIN4	DOK2	DLG1-AS1	STX11	TEX52	AKR1B1
			ALB	HES6	RDH10	TMED2-DT	GAS2L3	IRS4	DNASE1L1	SCARB2
			LNCTAM34A	MGC2889	NTNG2	CALB1	SPEM2	TTC32	CLDN18	GASK1B
			TNFRSF10A	CD274	TEX30	LINC01004	MIR155HG	POU4F1	TIMP2	DNAH3
			TNFRSF10C	NOTCH4	DUSP5	CFAP69	C7orf57	LOC100289361	IQCH	EID1
			EDA2R	PCOTH	SEMA3G	CBR3-AS1	HIF1A-AS1	ZNF252P-AS1	BBIP1	SH3GLB2
			BBC3	LOC100506804	LINC02574	CCDC144NL	AXIN2	ADAMTS20	MLXIPL	FOXR2
			TRIM22	ANKRD18DP	LOC101930370	LINC01186	HES2	DENND5B-AS1	COX7B	PNPT1
			GAS6-AS1	PTX4	MMP10	FAM76B	NDUFAF4P1	SHOX2	MALL	CCNI
			MDM2	DLL4	LINC01772	PGM5P2	GNRH1	LOC105375690	DNAJC10	TPD52
			WDR63	TFAP2C	TMSB15A	EGR3	GRIN2D	SOX11	SH3BGRL	LINC01624
			MUC19	MCM8	TAS1R2	LINC01138	RNU5E-1	PTGES2-AS1	TXN	HSPE1
			CDKN1A	IL21R-AS1	TBCC	LOC285626	MAFF	HP09053	GUF1	ST13
			SULF2	CHKB-DT	BTLA	CRYBA2	ARRDC3-AS1	GALR3	CTD-2194D22.4	CALM3
			KIAA1257	EGR4	EGR1	LEFTY1	CACNA1E	SCN4B	RASGRF1	CXADR
			FGA	TMEM60	BMP4	NTRK2	LOC145694	LOC101928058	GPI	S100A6
			NTN1	COL26A1	USP6	LOC100287467	KLF4	INTS6-AS1	UQCRHL	G6PD
				MIR3153	SNAI1	EGFL7	LOC339166	SNORA55	FBXL16	
				GFPT2	RNF138P1	SNORD166	ZNF295-AS1	KCTD15	FKBP3	
				RNF223	H2AC11	LOC102723493	SLC16A10	CEP83-DT	PHYH	
				LIPG	KDM7A-DT	TMEM198B	ZNNT1	SNORD88C	CASC4	
				PTX3	LINC00535	LOC652276	SLC35A2	LINC01191	SNX32	
				ADAMTSL4-AS1	EPB41L4A-DT	RABL2A	SLC9A3-AS1	MYRF-AS1	PGAM1	
				NUP35	GMFG	OR10A6	BAIAP2	SCN3B	CREG1	
				LOC101930421	ZNF77	PRDM1	ULBP2	PJVK	CD55	
				ADAM11	FZD10	RTP2	EZH2	FBXL14	GLB1L2	
				SAP30L-AS1	LINC00173	SLC13A5	RECQL5	LOC100287808	EIF2S3	
				SH3GL1P1	SERPINB9	PAX3	SNORD94	S100P	TAFA3	
				DIS3	MED19	THUMPD3-AS1	MYBL1	KBTBD11-OT1	SPG21	
				MGARP	LOC101928696	CABP4	HMGN2	MOSPD3	SHANK2-AS1	
				MYC	CBLN2	SNORA33	IL17RB	CBR3	FAM122B	
				FUT8-AS1	B4GALT1-AS1	HLA-F-AS1	SNHG19	FOSB	THSD1	
				GASAL1	TWIST1	MAFB	ZNF446	SPTY2D1	ABHD12	
				C9orf106	LRRC56	GEMIN7-AS1	CKMT2-AS1	AMER2	CATSPER4	
				LOC101927157	SGMS2	LINC01003	ARHGEF7-AS2	VSIG10L	EIF3I	
				LMNB1-DT	SMARCA5-AS1	SLC7A3	LEF1-AS1	LINC01949	COX7A2	
				RUNDC3A-AS1	ROM1	PCDHGA1	UBE3B	ARRDC4	SELENOW	
				SNORD17	SRMS	MYOC	NCL	PIF1	MYO7B	
				RAET1K	GDF7	PCDHA1	EDN1	TTC21A	GPN3	
				MYO1F	SIX2	C8orf33	MDGA1	SESN2	RPL19	
				NLRC3	LINC01743	PEG10	PARTICL	SNORD80	PITPNM1	
				MMP11	MPO	AFDN-DT	RSKR	ODC1	IGF2	
				ERO1B	HYKK	APTR	LOC101927418		LYPLA1	
				RPRM	POU4F2	PODNL1	CENPL		TMOD4	
				MAP3K21	GLA	LEAP2	RAX		CFL2	
				ZBTB11	WWC2-AS2	LETM2	HLA-L		SELENOF	

ACLA.DOXO

PILRA	ARHGAP30	TPT1	BST2	FTH1	AGAP2	LINC01554	FAM83A	H3P6	XKR7	HLA-DMA	P4HB
PMEL	PARGP1	B3GNT6	CLDN1	ZSCAN10	FAIM2	AVIL	HHLA3	FGFR4	CCDC17	TTLL3	CLSTN2
ACOXL-AS1	CFB	RPS11	VWA3A	FKBP1A	ITGB7	EPS8L1	RPS19	PLB1	RPS9	NEK8	GUK1
OVOL1	SCARNA21	SRD5A1	GABRR2	SNAP25	SLC26A10	RPS25	ZMYND15	CDHR2	CASC11	LINC01097	COL20A1
AOC2	NLRP10	SCARNA2	DNAJB11	STAG3	NEU1	ZFAS1	CRTAC1	B2M	ADPGK-AS1	TAC3	STAR
YEATS2-AS1	SCOC	UBA52	SCARNA7	PLEKHB1	EGR2	SYT5	GPX1	RPS24	SLC9A5	RHCE	SCNN1D
DUOX1	RPS17	OSTM1	IFI27	PTPRH	ATG12	SLC17A8	CXCR4	GAPDH	ALPL	SERPINF1	TMEM47
FOXF2	ST14	BNIP5	GFI1	STRIT1	PADI2	PPP2R1A	FAM3C	ICAM1	RPL18A	SKP1	LOC100129931
GOLGA8A	EVPLL	SRP14	FAM89A	SCARNA28	DSCAML1	PPP1R14B-AS1	MMP24OS	ZCCHC12	BMP8A	SLC8A2	CDIPT
SAMMSON	CHI3L1	DMKN	SLC17A7	NDUFA2	APLP2	GAP43	ATP2A1	MAP1LC3B	CIRBP	LOC401127	PLPP5
MTMR11	DDN	AQP3	LMAN2	ACAP1	NPC2	SLC43A2	SNHG32	EFCAB13	RPL22L1	TF	STIM2-AS1
GRIN2C	HLA-A	NPHS1	RPS20	DKKL1	PLEKHG4	LOC107986876	STPG4	FUCA1	WBP1	SLC23A1	LIN28B-AS1
SPATA21	LHX4	ATP6V0B	PARM1	IDS	CANX	COTL1	LAT2	TALAM1	GPNMB	FABP5	M6PR
PSMC3IP	PDGFD	LINC01039	HYOU1	PAX5	G6PC	CHCHD7	MGAT4B	PCDH10	CHRNE	MAGT1	GALC
AOC3	MDK	PDIA6	SLC39A6	TIPARP-AS1	PRDM8	ATP6V1G1	TBC1D2	PM20D1	PAXBP1-AS1	HGD	PAPLN
KIF9	TOR1B	TFPI2	RPS12	MFSD10	CYP26B1	CRISPLD1	INSM2	H1-1	CCN4	TSPAN1	LINC01149
IFITM1	ATP6V1E1	TBX1	LIME1	MAP3K7CL	CDNF	SAT1	RPL38	SERINC4	CHD5	LOC100130992	LTA
BEST1	KLRD1	CNIH3	CTSZ	HK3	ABHD14B	FAM177B	MICB	ITGAM	FST	NSG2	GPR83
CDH23	ENO2	ZNF236-DT	CYCS	FNDC4	LINC02014	GRN	CATSPERG	THBS1	CD68	HLA-B	DERL3
LYVE1	ANPEP	THCAT155	C9orf16	SEPTIN4	SOX9	PTOV1-AS1	SERF2	RPL9	GLO1	TFCP2L1	RWDD2B
KIAA1324	NXT2	CTSH	CA2	CNTD2	CELF6	IDI2	PINK1-AS	DHH	ITIH1	BMP8B	ITGA2B
OR2B6	RPLP0	QPCT	ADM5	RPS18P9	AP3B2	LINC02169	NXF1	CCDC168	C9orf152	CTSB	EPHX1
WDR49	GIPC3	GPR19	TMC8	RARG	MEIS1-AS3	SLC17A9	DNAAF3	BST1	NQO1	CRABP2	LINC02608
TSPAN13	COL9A2	CRELD1	HSPB6	ATP6AP2	LOX	BTK	CCN2	SLC16A6	SMIM24	NHLH1	TMSB10
ATP12A	GRTP1-AS1	TMA7	LINC01058	ABCG8	EVPL	PCK2	UBL5	DRAIC	KCTD13	PNOC	RPL37A
SCARNA9	C1orf162	GRAP	KLHL7-DT	HSP90B1	TNXB	CACNA2D4	TLX2	PHLDA2	HSBP1	SPEM3	MAFA
FLJ31356	FHDC1	TMEM167A	LOC100652758	BTN1A1	LAMP3	SNX31	GAL	TEX14	VAMP8	VHLL	OSCAR
C19orf67	MYH3	INE2	NANOS1	GRIA1	MYO1A	PLA2G4F	NAPB	AMPD1	LSMEM1	ARHGAP9	RUNX3
RGS5	SEC14L6	NEFH	RET	CFL1	HSPA7	MTFR2	RGS16	CLDN6	ACTA1	HOXA1	BSG
ELMO3	SNHG1	PNP	STAB1	MUC4	CT66	ANKRD35	SLC37A2	STXBP2	RSPH6A	RYR1	ATP6V1C2
PGRMC1	RERE-AS1	DNAH2	FENDRR	NR4A2	ARC	OLIG2	RHBDL1	HSPA1B	PTGDS	FAU	PDIA4
LOC100506358	BUD23	CYP26C1	APOC1	CCDC200	SNORA94	IGFLR1	LOC286059	CALB2	REEP3	OS9	SERPINB8
C1orf116	MYO15A	ATP6V0C	MATN3	WNT2B	PNLDC1	CCT6P1	TMEM253	LOC105379194	COL2A1	LOC101929457	DYNLT3
HAPLN3	FLJ31104	RHD	TENT5C	LOC102724814	MEFV	THBS4	CDC37	YBX1	ZNF687-AS1	LOC101928764	LINC00440
CYP27B1	FAM229A	RPL32	FAM131B	TIMP1	SLC5A5	PABPN1	POLR2A	SNORD22	LOC100507283	PKLR	SLFNL1
SLC39A8	TCTE1	CHRD	LINC01907	RPL12	NRIP3	SMCR2	TSPYL2	PTPRD-AS1	C6orf52	HAP1	GPSM3
GPC3	FGFBP3	TRAPPC6A	PGC	HSP90AA1	DLX1	NEXN-AS1	SHC1	SNHG29	HLA-H	C2	ADCY10
LGMN	TMX1	PDLIM3	HNRNPAB	GCH1	SLC25A34	PLTP	F2	DLL1	DMRT2	USHBP1	ROBO3
NMB	SLC4A11	PLCG1-AS1	COX7A2L	AMH	C3	AHSG	TCAM1P	ATP6V1D	GHITM	SNCA	ADAM8
FAM90A1	RPS28	PPDPF	LYG1	PABPC1L	APOE	RPL31	NOXO1	C8G	LOC101928617	MYLPF	PGPEP1L
RBM3	CD164	FAM135A-AS1	MIR1282	CILP2	CRYGN	RPS2	ITGB8	RBP5	SELENOK	TSTD1	SDE2
ADGRF3	FERMT3	LRTOMT	SCARNA17	IRAK1	SNORD116-18	SCML1	EHBP1L1	CIB2	KCNK12	CD14	CD79A
TMEM249	KITLG	GPR179	RPSA	ARL6IP1	FBXO2	CHRM4	INHBB	VAMP1	TACR1	LOC101929372	AZU1
DNAH10	GAL3ST4	EIF4G2	TAF10	TXNDC16	GOLGA8B	MATN1	DDAH1	ELMOD1	CCDC13	ADAM32	YPEL4
AIDA	SNHG6	LINC01503	LOC105374378	BECN1	HLA-E	LOC101927245	SEMA6B	IRF4	GNAT1	CLDN9	LOC101927551
CAPN12	PSMD6-AS2	NMRK2	MSH4	AGAP11	TRIM72	ZNF205-AS1	LOC441087	CTSL	TAC1	PAQR6	LOC101927974
CFAP43	MYCL	BIN1	C11orf91	U2AF1L4	CETN2	LOC105373311	GNAS	PIGT	UCN	TBR1	GNL1
LRRC23	RPLP1	CPNE9	NPPA	SRD5A3-AS1	GRM4	SRP9	KIAA1191	NWD1	LOC100130370	PCDH19	AGBL5
MAP4K1	RSRP1	ZNF124	CFAP157	SSR4	RPL41	LTB4R	SAP18	PSAPL1	RPS5	IFI30	

ACLA												
LOC101927322	TUBB3	ZNF90	FOXP3	FOXF1	SRP54-AS1	EPHA4	LOC439933	SCNN1G	SNAI2	HDC	CDC20
PTP4A1	ACTL6A	ZFP42	LOC105374952	CHGB	ACYP1	SS18L2	CALM2	ERICH3-AS1	FOXD3	ACTN1-AS1	LBX1
PIH1D2	DIO3	MEPCE	THBD	HSP90AB1	KBTBD8	FOXL1	LINC00426	FAM72A	SNORA119	ID1	EFHB
ZAR1L	CHST2	C5orf47	MKX	PGR	FAM43A	LCT	ZNF192P1	YPEL5	TMEM151A	IL17D	SCRT1
LOC642361	IL1RL1	ID4	AZIN2	CH25H	MEX3A	ADAMTS4	ELL3	LOC100288254	H2AC13	WNT1	GUCA1B
GREM1	TCAF2	LOC101060553	SNORD75	KCNIP2-AS1	LIMD1-AS1	CASS4	CATSPER2P1	AURKA	RPL13P5	PACRGL	MAB21L2
FCN3	HSH2D	NIPAL1	NPTX2	RNF122	ZBTB18	PTGS2	CSF3	CDC42BPG	SERPINB9P1	GMNC	NBL1
MCIDAS	TCF24	LOC100506100	PPP1R42	OR2K2	RUNX1	MYLIP	LOC105369332	USP12-AS2	LINC00472	LINC00167	AOX1
SNORD116-20	GJD3	LRATD1	LINC02569	RPP38-DT	TRPM6	TGIF1	CFL1P1	XRCC3	CDK6-AS1	PLVAP	TDRD6
RNF144A-AS1	TMEM269	DAB2	CCN1	H4C12	IL23A	VPREB3	UAP1	ZMYND10	MMP14	EXTL3-AS1	FAM117A
AK5	DNAJC25	TNFSF13	MICB-DT	LOC101593348	RIN1	MKLN1-AS	LOC105377879	KCNJ2-AS1	STPG3-AS1	LINC00652	LINC02018
BDNF	CNR2	H2AZ1	MELTF-AS1	LOC105379393	RTBDN	KCNG3	FAM83D	H2AC16	H3C11	LOC100287042	FOXD3-AS1
SCIN	HCG9	HDAC4-AS1	ZNF267	LOC100499489	C9orf163	LOC105369306	DCST2	LINC02506	MFSD2A	PDIA2	LOC100507634
LOC646903	GABPB1-IT1	AOC4P	LOC100128164	ELOVL3	RPS15AP10	SIRT7	SUV39H2	PSMB11	PPP4R1-AS1	MBLAC1	KMO
MRPS30-DT	LOC107984265	SYCP2	MARCKSL1	EIF5	H2BE1	MADCAM1	IQCF1	NLRC4	UBE2D3-AS1	NCF1	GAS5
MIR4684	NPB	SMPD3	LOC101929162	PGR-AS1	ANKRD7	FAM184B	C4orf47	CDR2	CELSR3	NOP14-AS1	EPHX3
AP4B1-AS1	STIL	SMCR5	LGR5	LINC01232	ISM1	GEMIN8P4	LINC00461	WDR5B	TINF2	LOC101928595	LYPD3
INAVA	LOC100130075	ARL9	CPXM1	PRSS47	PXN-AS1	ADAMTS1	CILP	LINC01623	CCDC62	EFCAB5	FMR1-AS1
ZNF547	DACT3-AS1	ACVR2B-AS1	FGD5	OTX1	WNT3	TBC1D10C	ZNF878	YTHDF3-AS1	N4BP2L1	GDF9	HMOX1
H2BC5	PLD6	GPBAR1	NKAPL	LOC730202	TMEM158	MIR4458HG	SPINT1	CHGA	SAXO1	FBXO48	RAD21-AS1
HNRNPA1P10	LINC02809	CFAP298-TCP1(	LOC102723544	LINC02251	LINC00574	TBX18	LINC01562	SOX2	LOC105370687	NSUN3	PYCARD-AS1
TDRKH-AS1	LHFPL3-AS2	FOS	MIR3175	SMIM4	LINC02289	RPS18	TFAP2A-AS1	SNORD99	MIR933	IHH	OR10A3
PAQR9	LOC107984507	EXD1	MYB	NPHP3-AS1	STAM-AS1	DENND6A-DT	LOC112543491	B3GNT5	ZYG11A	CHST8	NUAK2
ANKDD1B	SALL3	ASIC3	IRF1	NBPF18P	ABCC2	CPEB2-DT	EPB41L4A-AS1	MC5R	LOC100996419	KDM4A-AS1	ITGB4
GLYCTK	SOX18	DLX3	AKAP14	FSCN1	COMP	SP4	FGF19	DDIT3	LOC100996842	FKBP1AP1	TRIM17
ARL4D	PCDH17	SNORD79	C6orf120	ZIC5	PROC	LINC02610	LPGAT1-AS1	PLCD4	LOC101927446	H2BC18	CCNJ
C19orf38	INSYN2A	TEX43	NUTM2B	STK17B	LRRC4C	RPL21P28	MIER3	TSPEAR-AS1	MAB21L1	HRAT92	LOC101928725
FLJ38576	MID1IP1	TDRD10	IRF8	LHX6	YOD1	APLN	DNAJB5-DT	CTSK	C8orf89	RPL23A	LOC101928436
NOP56	TAX1BP3	MIR3064	ZNF665	CDK2AP2	ZNF695	LINC01391	RAPGEF4-AS1	LCT-AS1	BRWD1-AS1	SRGN	LOC729966
LOC283299	ESX1	KIF4B	LOC101929657	LOC105372483	DTHD1	SNORA57	TMSB15B	LINC00115	FUT3	CARMIL3	CYB561A3
GGN	LOC102723582	H1-6	RNU12	SERTAD1	DINOL	AURKC	SNORA79	LINC01430	GSTM3	ZNF830	FBXL6
MAGEB10	NUTM2D	GSTO2	KCND2	TBC1D26	LOC114483834	SPART-AS1	LOC100507551	ZNRD2-AS1	PRDX1	SLC7A5P2	C4orf36
DUSP1	ZNF273	TENT5A	PITX3	DACT1	RAMP2-AS1	LOC401176	TDH	SNHG3	ZNF670	DMBX1	HMGN2P46
GLT8D2	JPT1	HCP5	GRHL1	FAM166A	MIA	ANP32A-IT1	TSGA10	SNHG7	SNHG8	LRRC4	CLCNKA
PAN3-AS1	ARGLU1	GNLY	FOXB2	ZC3H12D	TSPEAR-AS2	IL18BP	SERTAD4-AS1	TG	HSP90B2P	NOXRED1	DUSP4
MALINC1	NUP210L	C1orf109	CETP	CSKMT	RPL3L	C4orf46	ZMAT2	PKD2L1	SCAANT1	TMEFF2	PTGER3
RND3	TRARG1	BOLA1	POLE3	THEMIS2	EIF2AK3-DT	POU3F3	TMEM121B	TBPL1	PHYHIP	LOC399975	VDR
HIPK1-AS1	KMT2E-AS1	IPMK	FAM185BP	GLRX	CTH	ASF1B	LINC01132	UBXN7-AS1	SNORD28	CDC42EP2	GABARAPL3
SLC20A1	C12orf60	TMCO1-AS1	CARD9	LINC01124	DISP3	GINS4	VASH2	RND1	CDC20B	DDIT4L	MARCHF10
RSRC2	LOC148709	YIPF7	VGLL2	MED29	PPEF2	SPATA5L1	ADAMTS5	RBM48	NKX6-1	MEG8	SLC26A4
THAP7-AS1	GBX2	FOXL2	EPOP	PTGS1	INSM1	CEBPA	CDKN2C	RBM12B-AS1	C14orf28	DCHS2	MAN1B1-DT
WFDC3	MIR3143	ILF2	CNR1	SPDYA	CCDC126	SNORA16A	TNFAIP3	ARHGAP11B	ELOA2	FZD9	UCN3
ZNF551	C20orf204	ESAM	H2AW	LOC101928053	RPL13AP5	SGO2	LCMT1-AS1	GPR101	CNIH2	SENP3-EIF4A1	IZUMO4
AK8	A4GNT	ERICH4	FAM209B	LFNG	MMADHC-DT	ARHGEF39	PDE4C	ALDH8A1	ING1	CCND2-AS1	SH2D5
QPCTL	CARMIL2	STC1	GTF2B	COL6A4P1	DUSP28	LOC100287015	NKX1-2	H2AC15	ZCCHC18	PPM1K-DT	HSD11B2
SFRP2	LOC101927932	H4C7	MIR6758	TCTE3	AVPR1B	PLEKHN1	RBP7	TNFSF9	RAD51AP2	BCAR4	VTRNA1-1
FLCN	TMEM243	LINC02150	CCDC71L	EFNA2	GADD45B	IRF3	GABPB1-AS1	TMEM236	LINC02168	CBARP	CFAP53
GATA3	H3-4	SEPTIN3	FSCN3	KLHL40	PBLD	LOC105375744	P2RY2	LINC01617	H2BC6	TMEM151B	LINC00634
SPAG6	SOWAHB	TBX3	MXD1	MUC13	RPS27	C21orf91-OT1	SOWAHA	CAND1.11	RHOV	KLF10	CFAP20

REM2	PLIN5	UPK1A	LOC646626	DHX58	LOC101927402	CCNE2	HAUS8	ERAS	MIR5047	SHH	CITED1
SPHK1	ZEB2-AS1	TBXAS1	SNORA48	DSC3	SOX21-AS1	CFAP57	LOC101926935	MOCS3	ZNF778	POLR1E	GATA2
CEMIP	SIX1	MIR503HG	MIR640	AURKAP1	HHIPL2	SCYL3	SP5	SLC25A34-AS1	LINC01626	MEX3B	SNORD48
KIF21B	ALOX15	LOC100128386	LBX2-AS1	MED30	SNORD12	SNHG5	TMEM225B	HTR7P1	ETNPPL	RRN3P3	FAM86HP
RFPL1	H2AZ1-DT	PRKCG	EN2	LOC112268124	LOC100335030	BNC1	SNORD12B	MGC12916	PRR3	C19orf48	RNU4-2
ARHGEF33	AMIGO2	LINC02158	HLA-F	OSM	FGF8	TAF13	ZNF442	LOC106660606	NUDT9P1	RB1-DT	ETV7
ERN1	MCOLN3	SBSN	DLX2	LOC100130027	CCDC170	PDCD4	ESR2	OASL	TFRC	GALR2	BTBD19
TRMT112	CTSW	LINC00184	TNFRSF10D	TNNC2	H4C13	MIR320A	PER2	LOC105376306	SPRED3	PCBP1-AS1	ADAD2
RELT	CD27-AS1	NR4A3	PCDH8	HAVCR2	SLC28A1	TMEM208	CD3EAP	HSPA5	ZSWIM4	DBR1	MFSD2B
LOC107985946	SLC6A13	LINC00641	SNIP1	RNASE10	MDP1	RPS3A	GAD1	MAK	SEMA3F	ELAVL3	ZDHHC19
TSPOAP1-AS1	CALCB	LOC107984208	CHRNA10	VGLL3	LMO7DN	MXD3	ZNF646	APOM	IDH1	LOC102724596	UBB
POLG2	HSPA2	FRAT1	SP6	LOC101928504	LINC02080	RAET1L	CLCF1	H2AC7	LOC101927164	CCDC189	MZT1
IL12A	C17orf98	GNG3	NPBWR1	FRMD6-AS1	CPB2-AS1	LOC101927354	GJB2	L3MBTL3	LINC01703	OTOP2	NEDD8-MDP1
NDNF	IQCN	CDH24	LTB4R2	SNORD55	NPC1L1	DEPDC7	LOC101927919	LINC01558	LINC00938	C12orf45	RGCC
KCNJ9	NME2	ZBTB6	H4C6	B4GALNT4	CCK	TMEM217	MIP	SEZ6L2	ZNF597	SNHG21	ZNRD1ASP
CDO1	ID2	LBX2	ANKRD42	PIM2	KLF1	PDCL	BIN3-IT1	CNPY4	LINC01285	TPH1	LINC01730
FNDC7	LOC107985184	ZNF674-AS1	FOXE1	C4BPB	DDIAS	SLC16A9	LOC107985820	USP17L7	ZMIZ1-AS1	TENM3-AS1	PANDAR
HERC5	OTULINL	EXOSC10-AS1	TMEM246-AS1	LOC101927620	GZF1	CYP26A1	SMIM32	GAS1	HHEX	SLED1	EREG
HPCAL4	SNORD29	ERRFI1	VN1R2	CDCA2	PKD1L3	RPL4	BPGM	LINC01356	MCL1	NTF3	S100A1
GANC	ALOXE3	NHLH2	SNHG9	NAA38	RBM15-AS1	ALOX12-AS1	KCTD5	TNFSF14	LOC100289230	SNORA24B	CD7
PLK2	NCBP2-AS1	DNAH12	OVGP1	SNORD78	ATRIP	MIR4453HG	VAX1	C14orf178	ERICH5	RHEBL1	LOC100379224
PRPH	NDRG1	PIH1D3	HSPA13	HSF4	PSD2-AS1	IQUB	SNORD27	LOC101928068	KCNA1	RPL13A	TESK2
FOXJ1	WNK4	UBXN8	POLR2K	LOC100289333	OSR2	SH3BGRL2	LOC100506679	WDR73	ABHD5	SREBF1	RNPC3
ADPRM	ART5	YJEFN3	PRSS41	CIART	UCP1	WDR74	CPEB1-AS1	IRGM	SNORD47	IER5L	C21orf58
RHOB	HEXIM2	JUNB	ODC1-DT	CHAC1	WNT9A	SDC4	HSP90AB4P	FLJ30679	MORF4L2-AS1	RIPPLY2	CRYBG1
KCNJ2	C21orf91	C3orf80	BZW1-AS1	DSTNP2	TTC22	RAMAC	LOC101929234	HMGN3-AS1	ULBP1	ENTPD3	SPATA1
GPR152	H2BC12	RGMA	PLEKHH2	ZFP36	SNHG12	CNNM3-DT	SNHG26	FLJ32255	CREBRF	RNF213-AS1	CAMTA1-DT
CXCL14	C11orf96	RASAL2-AS1	PPIEL	LDLR-AS1	SNAPC2	RECQL	FLJ37453	SNORD74	SOX4	PTCH1	LOC101928728
C2CD4A	CERNA3	SLC5A10	DDIT4	FLJ20021	DNAAF1	PDE5A	LINC00526	WDR46	ZNF425	CTSF	BHLHE22
FAM71E2	THEGL	GPR21	LOC389247	ATOH1	TAS1R3	KLHL15	COL12A1	DNAJC3-DT	LOC100133315	LOC101927420	NUDT10
PCDH9	GDNF	CLP1	LINC02586	SEMA6A-AS2	VSIG2	TRIM69	TSPAN10	HMGCL	POU3F1	LOC101928021	C6orf141
LMO7DN-IT1	LOC100505555	NRGN	HIC1	DCAF4L1	SGK2	MIF-AS1	HSPA1A	CCNL1	LOC644656	LOC91548	CDK5R1
ADAM20	LOC107985939	ARIH2OS	UBL7-AS1	ISG20	SERP1	LRAT	ACER2	FGFR3	RNU4-1	CCDC77	CORO1A
HMGN5	BAAT	CNOT2-DT	GLUD1P3	TMEM95	CRX	DNM1P46	FLJ13224	SNHG10	DOCK9-DT	UVRAG-DT	NCAN
SPAG5-AS1	LOC286437	CCDC183-AS1	PRAG1	FAM21EP	GATA3-AS1	LOC105375218	THAP10	CEMP1	MIR3142HG	COL28A1	LOC100128494
PNN	IL4I1	MED15P9	TPBG	LINC01133	RNF224	H2AJ	SEC24B-AS1	HIGD1B	EEF1B2	RMST	LY6G5B
LOC101928659	CCM2L	H1FX-AS1	LOC105747689	NGDN	LINC02551	SLFN11	SHC4	SNORD76	H2BC17	LOC101928847	MASTL
FBXW9	HOXD8	LRRC15	TRIL	DARS-AS1	INA	ACBD7	LOC112267983	LINC01431	FOXQ1	RASD1	NEFM
LINS1	CELP	CSRP2	RALY-AS1	LOC284241	ESCO2	PRPS2	ICAM5	SDCBP	SLC22A4	LDHC	SLC9A1
HS3ST2	ZSCAN12P1	PARD6G-AS1	PGM5P4-AS1	NIP7	LOC101928222	KCNA3	NEMP1	LAMTOR5-AS1	ANKRD26P1	SP2	KLHL11
ZNF560	ZIC3	FLVCR1	MIR425	MIR191	CPLX4	B4GALNT1	SNORA73A	BCL2L15	ITGA10	LOC401261	RAD51AP1
TFAP2E	SYCE1L	PTTG1	CCDC163	GPR1-AS	CDC25C	ESRP2	ADAMTS8	SNORD110	ITGA6	FAM107B	IRF7
MSX1	ZNF773	FBXL13	GAST	LOC100506885	NDUFA6-DT	LOC441086	H3C4	KCTD8	ADRA2A	FETUB	SLFN12L
DCXR	MAF	SOCS3	PTMA	NR0B1	SSTR2	C11orf16	TLR2	TMEM14A	RTN4RL2	RBM26-AS1	LOC105370943
C9	CSRNP2	ORC6	MIAT	NKX3-2	CHI3L2	CYP1B1	LINC01465	TMEM100	LINC02478	ZIC4	EAF1
RPS27A	CXCL3	ADCY10P1	ITFG1-AS1	CRNKL1	LINC00449	CCDC96	GNG4	LOC105372440	CEP164	C10orf143	H3C10
C5orf15	THAP2	LOC100505915	SEPTIN1	TWNK	SEPHS2	CES3	ST7-AS1	PNKY	SMG1P2	MST1	FGF4
ZC3H18-AS1	ADM	GUCY2C	CTRL	H2AC8	H2BC20P	LOC101929935	LOC100131257	CCNA1	ZNF230	FAM182B	TNFRSF4
LOC101929626	CTSV	FOXD4L1	CNTNAP2	CYP1A1	WNT9B	NRARP	WARS2-AS1	UBE2E2-AS1	ZNF280A	LRRIQ4	L3MBTL1
HSPH1	CRISPLD2	CA8	F3	FUT6	OXCT1-AS1	CCNB1IP1	EOMES	COCH	SLC6A20	HLX	LOC108783645

IL11	LINC02588	NPPC	OSMR	LCA5	RAB44	SGK1	SLU7	CGAS	MR1	SNORD26	FOXO4
LZTFL1	BTG2	OTX2	MAPK8IP2	FOXD2-AS1	MIR645	LOC100132356	PDK4	MIR4651	HSPA1L	LNP1	ACTG1P4
MIR3174	PRR18	RPSAP52	SNORD81	CCDC82	ATP2B1-AS1	E2F7	H1-10	LRP2BP	SOCS1	DEPP1	H2BC11
GATA6-AS1	CHODL	TM4SF19-AS1	STKLD1	RNASEL	AGAP1-IT1	MIR194-2HG	IL18R1	RFPL3S	IGFL3	PDC-AS1	NEUROG2
TPTEP2-CSNK1E	ZNF705A	ADRA2C	STMN1	TPH2	LOC101929679	MASP2	SLC30A1	PTHLH	ITPR1-DT	ACRBP	RGMB-AS1
ZSWIM8-AS1	H2BC21	SLC26A8	FJX1	DIRAS3	H3-3B	DBF4B	LOC105379143	LINC01033	NIPBL-DT	LOC647070	TMEM86A
TMEM91	RNF39	PPM1N	LRRN4CL	MIR17HG	ZNF488	GLI1	TRIML2	RBPMS-AS1	HIGD2B	CCNI2	C9orf64
MIR4308	UNK	TMEM119	SNORA80B	SLC5A3	SPEF1	LOC107985544	LINC01348	C2-AS1	SNORD134	ZSCAN29	LGALS8-AS1
PIK3R3	GADD45G	LOC730101	ZIC2	GRIN3A	PSPN	CWC25	GAL3ST1	ALOX15P1	ARAP2	KLLN	FAM53C
LGALS4	F13B	PPP1R27	SLC23A3	LOC344967	HMX3	SLC25A30	ARHGEF26	LONRF3	NDUFV2-AS1	FBXO5	NPTX1
LINC00921	STX17-AS1	FAM83A-AS1	H3C12	RCC1	PLA2G12B	SLITRK3	LINC01471	ZNF79	LINC00537	TSC22D1-AS1	DRC1
NELL2	SPRTN	PTF1A	SECISBP2	SLC22A31	ELF3	GPR137B	CR2	CYP51A1-AS1	RNFT1-DT	FBXO24	HAPLN1
CDKN2B	ZNF324	SSBP3-AS1	LOC100422781	ZNF818P	PIGW	CERS3	MEIG1	SEPTIN12	SBNO1-AS1	BTG3-AS1	SOWAHC
CLDN11	FOXC1	EMC3-AS1	CAHM	GLUL	C17orf67	CEBPB	LOC730183	MPIG6B	SLC34A3	ZNF222	PLIN4
TNFRSF10B	LOC101927051	ZC3H12A	HAS3	TUBB4B	LOC101927023	SPEM1	CFAP54	GCNT4	CARD14	PCAT7	LOC101929748
ADAM30	ACTR3-AS1	LOC107984813	LOC115308161	OR5AU1	H2AC17	RIPPLY1	GNAL	RNASEH1	PDCD4-AS1	HASPIN	MUC5B
KCNJ14	GLCCI1-DT	SLC6A4	H2BC8	TUSC8	GPRC5A	RTCA-AS1	PCDH18	PURG	LINC02043	KANK3	MARCKS
TLCD2	POLR1C	KLHL28	TLNRD1	ZNF550	MOBP	LOC101927391	PPP1R32	GAPLINC	LENG8-AS1	AGAP2-AS1	SIX4
SCARNA5	C16orf46	MPZL3	ESPN	DUSP2	JPH4	LINC02102	TXNL4B	LOC642366	SMCR8	TRHDE-AS1	KLHL33
LOC100130172	MUC6	CDKN1B	MISP	ID2-AS1	SOCS2-AS1	NABP1	RGMB	PLIN1	SOX3	FAM72B	S100A14
COA6-AS1	LINC01424	CD164L2	LINC00628	CCDC157	IRAIN	RNF112	ZIK1	RPS29	KRT18P55	ISL2	ODAPH
LOC101928370	PIMREG	BTG3	CAPN10-DT	C16orf92	DNAI2	KCNJ10	GPR150	MIPEPP3	NPL	LYG2	ATF3
CYP7A1	KCND1	STPG3	CHMP1B	SHISA2	FAM172BP	ARL4A	SETD6	LOC100506639	PTGER4	TAS1R1	PAX9
PCF11-AS1	IER5	ACTL10	MLNR	TMEM255B	HCG27	SNORD30	ALDOA	LOC105372795	LINC00862	C3orf86	LOC100272217
PITRM1	CEMIP2	SLC25A53	NTN4	SLFN12	SERPINF2	LOC105371433	TMEM52	RPL37	FGR	RHBDF1	C2orf42
E2F1	DHDH	ABCB1	SERPINC1	SNORA73B	CFLAR-AS1	H2AC12	HCN3	CGGBP1	EEF1AKMT3	PDGFRA	USP2-AS1
BATF3	PHOX2A	ASH1L-AS1	CACNG8	DKK2	FOXC2	GEM	EFCAB12	PDZK1	ARF4-AS1	LOC285638	CEL
HDAC2-AS2	PPP1R15A	SLFN5	SNHG30	WDR90	RPS16P5	CXCL2	LOC101927365	DHX34	ZNF821	MMRN2	HES1
ZNF844	IQCA1L	HAO1	AXDND1	FZD5	BROX	RASSF10	LOC100129203	RNU5B-1	AP1AR	FAM174C	LINC01599
LOC441052	FOXL2NB	FAM71A	CEBPA-DT	FEN1	ACSM1	MZB1	CSE1L-AS1	GMCL2	HEXIM1	LOC643072	IFRD1
LINC01970	CDKN2AIP	BFSP1	ACSL5	HTR1B	LOC100506282	LOC105370697	NNAT	FABP5P3	NPIPB6	IL15RA	LINC00637
HYPK	LINC01090	FTCD-AS1	PTAFR	PIP5KL1	LOC101928940	MAP3K20-AS1	LINC01647	NEURL3	GAGE10	LOC100128770	PET100
ZNF367	KBTBD7	C16orf91	EMC1-AS1	DBIL5P	RTL5	LOC100129917	RNU4ATAC	PNPLA1	DM1-AS	CCNG2	AMY2B
LOC729867	TIGD3	SNX22	RUBCNL	TMC4	ZNF280C	MIR6843	PIWIL2	CA3-AS1	SLFN14	LINC02072	TTC41P
CAGE1	LINC01311	LOC283028	LINC01583	C5orf34	SOST	AGBL5-AS1	FOXI3	ZNF460-AS1	EPB41L5	EPB42	ZNF850
LOC100652999	SNORD100	ZWILCH	LIF	SP9	MEF2C-AS1	SATL1	LOC729683	CCN3	TAL1	METTL21A	HOXD1
LOC101927278	CHST5	BABAM2-AS1	LOC100134391	GRM2	LOC102724708	ZNF416	DLEU2	DALRD3	C2orf80	PM20D2	LOC645967
LOC646548	ZNF788P	ANKRD49	C11orf95	NR2F2	FZD7	ZNF92	PDZD7	LINC01089	HMGB2	MCOLN1	NUSAP1
ABCC11	ZNF341-AS1	THAP9	RASL11A	TLE4	LINC01363	SLFNL1-AS1	TLCD3B	POU3F2	SH2D4B	ANXA2R	AVPR1A
MPP4	RLN3	PDZD3	ATOH7	C18orf54	LOC100289511	ZNF763	VASN	SNORD31	H2BC3	DCDC2B	CKS2
LINC00884	PARD3-AS1	KRTAP19-1	ZNF433-AS1	MIR1302-3	PMS2P4	GRK6P1	PMAIP1	RRH	NKPD1	KL	CLU
RAD21L1	CAPN11	WDR97	THSD7B	MOB4	MAP11	LINC00863	SPATA6L	PHYHD1	RPL23AP64	FAM209A	SCARNA6
LINC01762	RAB26	LOC107984341	AMIGO3	LOC101927666	AGBL3	TBX21	NKX3-1	METTL14	NECTIN3-AS1	ADRA2B	CCDC178
FAM110A	CBLN1	TRIM36	GOLGA2P5	PHETA1	PCDHGA2	PROCA1	CCNYL1	CDKL3	CA4	DPEP2NB	H2BC14
CCNO	FOXD2	LOC101928445	BCL2L11	H2BC13	GSKIP	CBX3P2	LRIT3	AIFM3	C9orf147	LOC101929229	MTF1
TUBA1C	MMP19	C9orf153	SYCE2	FRMD6	ZNF394	IER3	PGM5P4	LOH12CR2	FUS	USP17L2	SCX
RRS1-AS1	NT5DC4	KANSL1-AS1	LOC101929762	LINC01181	SEMA4A	TRPV2	CCT6B	CABYR	KLHL29	ZEB1-AS1	LINC00272
LOC106699570	DHX35	SMAD5-AS1	WDR88	YRDC	MTBP	HTT-AS	PRAM1	ZNF155	LOC105370362	IQCD	TRIM7
CEBPD	MAP1LC3B2	RGS4	MIXL1	NFKBID	WDR78	LINC02575	OAT	ZNF433	FOXA3	KLF5	CDH16
ZGLP1	COLEC11	HBEGF	IMPA1P1	LOC51145	MID1IP1-AS1	PERCC1	RASGEF1B	IFI6	TRIM45	POC1B-AS1	FUT5

DOXO

ARL2	STMP1	RASAL1	CPLX1	CALU	COX6C	NDUFA4	HLA-C	PIGK	EMC7	FKBP9P1	ITIH3
GET1	TCIRG1	BEX3	USB1	CCNL2	IGFBP2	ANKLE1	MROH2A	AP1G2	HSD17B14	NYX	MFGE8
PIDD1	LEPROT	INCA1	FLT3	HACD4	MTMR2	PRMT1	ITGB1	GSDME	CD5	IPO4	HHIP
CALR	MIF	NOMO2	BTBD1	LINC00926	ENO3	AP2M1	OTOF	SLC41A3	PFDN5	SEPTIN6	SEPTIN7
NDFIP2	CFH	LMAN1	IRF5	MED24	TMED7	APOA4	HSD17B12	NDUFA7	C20orf173	NDUFB8	LOC101929538
JAK3	FKBP8	COL13A1	OTOG	RPA3	NDUFV1	MYL12B	CSDE1	FADS1	CTTN	SLC14A2	SRM
GPC2	TIMM17B	KCNC1	MDFIC	FLOT2	PDLIM7	SHC2	CCDC124	UBE2E4P	ERGIC3	SLC13A1	TLE5
GLUD1	GDI1	JMJD8	UCP2	EIF5A	LBHD1	LOC105371414	KLHL1	DUSP13	ETFA	TSNAXIP1	GALNT7
DYNLRB1	VTA1	ATP5F1B	LINC00239	CPZ	CD9	RGS9	METTL9	ERH	CRIP2	PCBP2	TPGS2
CFAP61	P2RX1	RN7SL1	HMGA1	B4GALNT2	SUCLA2	MIR3654	FER1L5	WNT4	LOC101928855	COL9A3	ASB5
ANXA5	MUC16	CD46	OR7E14P	LOC221946	CREBL2	IGFBP3	SPCS1	LAPTM5	RPS10	UQCRH	PIN1
UXS1	GGT5	LACTB2	PLCB1	HIBCH	FUOM	CFAP65	SNX3	APP	TMEM59L	MICOS10	SOD2
HPRT1	TMED5	COX17	NSUN5P1	VTN	RNF5	RPS15	SIGMAR1	CAPN14	IL32	LOXL2	MMP2
LOC651337	MEST	GINM1	HOXA10	SMDT1	ACYP2	CDK16	STMN3	HIGD1A	MLLT11	SAMD14	LOC730098
EMC10	NME4	CLPTM1	VBP1	COPS9	GGH	MICU2	GNS	PERP	AFAP1-AS1	CACNA1F	EML2
MAPK3	NDUFB3	GET3	APLP1	BCAP31	SH3BGRL3	STK26	PDHA1	TMED10	ECM1	SMS	PSAP
TP53I11	TMEM97	SKOR1	PCDH20	RNF11	LAPTM4A	NDUFA5	CRYGS	SARAF	ADGRA1	PNCK	PRDX6
SH3KBP1	TNFRSF21	PHB	UBAC2-AS1	UPK1B	P3H2	USF2	CACNA1S	CHP1	KDELR2	ARMH1	RGS11
IFITM2	PAX8	PACS1	SERPIND1	CLIC4	MTFR1	PSME1	HS6ST2	RNF128	SUMF1	SLC66A3	NOS1
AGPAT1	RWDD4	MUCL3	DANCR	C1orf54	GPR137	VWA5B1	LOC101927851	CYB5R3	PROS1	ITM2B	IARS2
DNAH17	SLC6A12	OSTC	SEMA3E	LAMP2	SNRPD2	DAD1	MME	CAPZA1	PEBP1	MPV17	ENO1
STAC2	HPN	TEX9	PTS	BABAM1	PITPNM2-AS1	RNF141	NRXN2	SEC61G	ECH1	NIPSNAP3A	ACOT7
SLC22A18AS	MDH2	EIF1AX	GIPR	CDH15	PDIA3	DEK	PPIA	BCAT2	PPP1R14B	MOXD1	DDOST
LUCAT1	NPRL3	RPL29	AP1S2	ALDH3A1	FRMD1	SLC36A4	RAB13	RPS6KA2	PLOD3	SUCLG2	NUTF2
MAPRE3	PRR26	BCAT1	SPCS3	LEPR	IFITM10	SPOCK2	GGCT	CALR3	ACADVL	PRSS30P	TM2D1
ECHS1	TMEM63B	MORN2	AK3	PRDX2	TGOLN2	PPP1R1B	CA9	RINL	LAMP1	ATP5MG	TIMM17A
RPS8	UCHL1	CYSTM1	ARL1	MMD	HDGFL3	CDSN	TMEM230	YWHAE	SLC12A5	RMDN1	PRDX3
MYT1L	NUCB1	PLPPR2	LCP1	RRAS	TMEM59	UROC1	TMEM50A	VEGFB	RPL36	PGK1	GUSB
ALLC	COLGALT1	CYB5R1	TFAP2A	TAF9B	GABARAPL2	LSM4	RPLP2	WDFY4	SLC25A23	YBX3	CISD1
KDM1B	TMEM134	SEC23A	CD81	CD93	SGCA	C20orf203	S100A11	CLPTM1L	RNF5P1	NUDT15	ATP5MF
BPIFB4	CNTN2	TRIM52-AS1	ZAN	DPP3	PARK7	TMED4	TMEM54	MYL6B	YWHAQ	VDAC1	ACADM
SPAG17	NCSTN	TMED2	BRK1	COX6B1	CCDC68	TNNT3	PRSS50	CD151	KIFC2	R3HDM2	NDUFB5
LINC01168	ELOC	TMEM69	PKP1	SNORA104	RABAC1	TXNDC12	ADIPOR1	ADGRF5	HOXA11	POC1B	CDK5RAP3
HECTD2-AS1	METRN	EIF3H	CHMP5	OXCT1	DPY19L1	MAS1	STS	CENPX	COMMD6	IMPAD1	OSTF1
NECAB2	RN7SL2	SLC4A1	PRTFDC1	KCNMB2-AS1	CA11	COPS8	PRKACA	NDUFA10	PCMT1	ZNF358	C5orf64
TSTA3	LOC100506730	BOLA3	TMEM147	MT1H	REEP5	TMX3	CST3	ETFRF1	DNAH17-AS1	CRNDE	SELENOT
CYP1A2	TMEM14C	MACROD1	RPN2	NEK7	SDF4	ITGB3BP	STX1B	WDR17	SEPTIN2	DUS4L-BCAP29	TMED9
PPT1	GXYLT2	SNRNP27	WDR38	APOA1	PAIP2	HINT3	MIEN1	RCN2	CALD1	EFHD1	CNGB1
RPN1	PGGHG	CDC42	HAPLN2	TAF6	NRN1	TUBA1B	ANTXR2	APOB	ATP6V0E2	MYH16	PCSK4
PSD	VAC14-AS1	CCDC159	BSND	CDK2AP1	PAPPA2	BANF1	MZT2B	WDR77	LAPTM4B	SLC2A13	CASQ1
IL6ST	RFLNB	POGLUT1	PLS1	NDUFB6	DLGAP2	NRIP2	CCL21	RNPEP	NDUFA1	SIM2	PIK3R6
ME1	IFITM3	SHKBP1	COX4I1	SERPINI1	ABLIM3	KLHDC7B	ADGRL4	MBP	MBOAT7	VAV1	MGST1
CRYAB	CPXM2	NEDD8	REEP2	MAN2B2	C8B	HLA-DPA1	COX8A	COX6A1	EN1	FKBP1B	MORC4
ATP6AP1	NTAN1	DAZAP2	RPL30	SUB1	GAA	HADHB	MALAT1	FBXW5	ETV4	SLC25A39	ARL6IP5
UBE2N	FKBP11	ARPP19	ARL8B	NUPR1	TRAPPC1	SNRPE	GFM2	FAM177A1	LAMC3	LINC02298	GPD1
PARP6	WSB2	TPM2	PRDX5	RNASE4	SCUBE1	PON2	ARF5	TMEM41B	LAMA1	LARGE2	SPPL2A
BEST2	RPL39	IPW	LPCAT2	NSUN5P2	FBXO9	MELTF	QTRT1	NKX6-2	RTN3	CHURC1	TXNDC17
MAMDC4	TMEM30A	DDR1	CMPK1	HSPA6	RNLS	TFPI	FAH	TUFM	IMPA1	SKAP2	PTCH2
ATP11A-AS1	HSPD1	LAMTOR1	FKBP10	ARHGAP4	ITFG1	PEA15	RHOA	LINC00515	ADAM9	COX11	LOC283045

Downregulated DEGs identified from VennDiagram. Genes downregulated in ACLA/DOXO/AMR compared to DMSO (control). Cuttof: LogFoldChange <-1 & FDR <0.05

ACLA.DOXO.AMR	ACLA.AMR	AMR.DOXO		AMR			DOXO		
BLM	PBK	CCNB2	CDCA3	FOXM1	ZWINT	TK1	CENPBD1	RTL10	LINC01006
KNL1	MYOZ3	SAPCD2	H2BC13	DEPDC1	FANCD2	CDKN2C	KDM8	SCAMP2	CHD1
SPC25	RAD51	SUV39H1	H2BC11	OIP5	PIMREG	RAD54L	RWDD2A	ING2	MRPS12
NDC80	TENM2	NCAPG	CENPA	RMI2	GINS2	RPPH1	FAM220A	H2BC7	ZNF225
RGS8		E2F2	H2AC13	FAM83D	CENPU		TRIM52	TIGD6	VHL
H3C2		CDKN3	HASPIN	NUSAP1	ESCO2		UBB	DACT1	BLOC1S4
MKI67		ASPM	PKMYT1	STIL	ALK		ZBTB33	LURAP1L	C2orf49
BRCA1		H4C9	TRAIP	FGF12	DDIAS		UBC	ZNF329	H4C8
MELK		UBE2C	RACGAP1	CCNE2	PTTG1		KIAA2026	MCPH1	BAG4
RMRP		SKA3	GPER1	RRM2	MAD2L1		ZNF700	GTF3C5	ZNF850
H3C8		TCF19	KIF20B	RN7SL2	SHCBP1		CITED4	CYREN	NDE1
TMEM71		H3C12	UHRF1	BRIP1	CHEK1		HOMEZ	CCDC12	BCOR
CIT		TICRR	H2AC12	MCM2	MCM5		ZBTB49	NFXL1	ZNF696
SPC24		HJURP	KIFC1	KCNMA1	CDC45		ZBTB8B	NEU3	AMMECR1L
POU2F2		KIF23	CCNB1	ANLN	RN7SL3		CHD8	SMAD4	HDX
KCNK3		H2BC17	H2BC9	LEFTY2	CENPH		KBTBD3	TMEM185B	DYRK1B
NUF2		KIF11	XRCC2	PARPBP	MHRT		RAD9A	TAS2R31	CCDC59
CHRM1		CDCA8	CHAF1A	BIRC5	LOC100288637		SPATA25	LIN54	ZSCAN29
LRFN1		CDCA5	H2AC4	ZNF367	DSCC1		LRRC8C	CAPN15	ADAT3
KIF18B		KIF2C	TOP2A	RAD51AP1	CENPK		ALKBH2	ZNF383	MAP3K8
CRACR2A		SPAG5	CKAP2L	FEN1	FANCA		INTS9	ZNF263	INO80
BUB1B		H1-5	FBXO5	MND1	CDC25C		SFT2D3	MXD4	ZIK1
TPX2		CDCA2	KIF22	CHAF1B	ORC1		ZNF137P	DCAF16	SNX18
CENPF		H3C7		RN7SK	CDK1		ANKRD54	ZNF200	SETMAR
CDT1		EME1		ASF1B	GAS2L3		GRIN2A	PRR14L	NSMCE3
SGO1		H2AC11		MYH7	AGT		SMG8	CDK7	SP1
FRMPD4		ESPL1		NCAPG2	CENPW		ZNF565	GPKOW	EFNA3
RNF165		H2BC10		MCM10	TNNI3		CEP350	TUSC1	RAB39B
SKA1		FAM111B		HMMR	ERBB3		SAP130	RSBN1	TUT1
ERCC6L		PSRC1		CENPI	TROAP		RRAD	HARBI1	HERPUD1
KIF4A		IQGAP3		SERPINB2	CENPM		TIGD1	WDR20	TEDC1
CCNA2		BUB1		TNNT3	KLHL13		SPACA6	AMOTL2	CSRNP1
AURKB		PLK4		MYBL2	PRC1		USP42	FOSL2	MED7
CENPE		NEK2		WDR76	ANGPTL2		CCNE1	NFE2L2	
NCAPH		KIF14		MYH15	TGM2		ZNF460	ZNF253	
CEP55		TTK		HSPB2	TRIP13		BBS12	EIF2AK3	
IRX3		GTSE1		KIF15	NEIL3		METRNL	NATD1	
KIF20A		H4C4		MCM7	DSN1		MINCR	ZBTB43	
DTL		ARHGAP11A		POLQ	FANCI		GEMIN2	H2AC15	
FBXL22		CDC20		SNORD3A	PCLAF		ZXDA	SERTAD4	
		CDC6		BRCA2	C11orf21		TSKU	RHOB	
		POC1A		CKS1B	MCM6		LINC00663	MRPL20-AS1	
		DLGAP5		CLSPN	RN7SL1		TRMT5	ZNF888	
		PLK1		CDK18	PRR11		KLF6	ZNF226	
		H2AC16		HELLS	POLE2		H2BU1	CEBPB	
		KIF18A		GINS1	SUSD2		ZFP1	TTLL11	
		EXO1		FANCG	GINS4		ADAMTS1	RGS14	

ACLA.DOXO											
LINC01038	ZNF691	ZNF133	ATXN7L3B	ASXL3	ZNF445	EBAG9	ZNF717	SRRD	B3GALT4	CBFA2T2	N4BP3
HIVEP2	UBTD2	CCRL2	MKS1	PIM1	FANCE	LARP4B	ANKRD33B	C15orf61	RBSN	PIAS1	STAMBPL1
TSHZ3	TMEM74	MFSD5	LOC100287846	FAM83B	ZNF587	WBP1L	TMEM177	ARID1B	DANT2	ATOH8	TRAM2
ZNF527	KLHL26	PTCHD1	OSBPL6	FAM189A2	DUOXA2	MRTFA	RTN4RL1	SSH2	MIR490	H4C3	OPA3
SCAND2P	ARHGEF7	NUP62	ZNRF1	SNAP47	BCL3	THAP11	CNOT10	SLC30A2	H1-4	DAB2IP	TNS1
PDP2	MTMR3	RAB40C	ZNF227	CDPF1	LDLRAD4	METTL7A	EGLN3	PITPNC1	DPH5	ZNF710	APOBEC2
PPM1H	PKDCC	TRIB2	RPUSD3	TRPV3	SYNC	CCDC9B	HCN4	ZNF639	DAGLA	PTPRE	TRIM27
KCNA5	PHLPP1	CASKIN2	FILIP1	LPP-AS2	MRPL34	NRDE2	ZNF8	SMAD6	GATA4	WASF2	GPR146
ARFIP2	GAS2L1	MED27	NKD1	AKAP9	GSK3B	KAT7	FAM118A	VPS33B	FBXO31	SOX5	MEX3D
PRIM1	EIF1B	SEC24D	KIF21A	KLHL31	RAPGEF2	SPECC1L	NSD1	ZNF624	AP5S1	ZNF184	ZNF282
RNF41	ERCC4	CACNB2	SEC14L5	AUTS2	MAP7D1	DNAJC5	MSL3P1	PRICKLE1	MED9	RASD2	RAI1
EGFEM1P	ZNF397	EOLA1	H1-3	KANK1	H2BC4	TAOK1	GLIS2	RBM38	FAR2P1	MLLT3	IP6K1
RARB	POLD3	SRXN1	ZNF518B	CCDC85C	FIGNL2	WDR82	CNNM1	TOR2A	PRR5	CHST10	LRRC14B
FZD2	RPP25L	CORO1C	JARID2	KSR1	PROB1	A2M-AS1	CENPB	H1-2	GFOD1	TBC1D20	ALKBH4
BCAR1	MARS2	POLR3A	HEIH	HERC2P9	HS3ST3B1	GRAMD4	ZNF135	MAPK14	FBXO40	MN1	H4C2
FHL2	MCMBP	HOXB2	RREB1	ZFPM2	RP9	STK10	MAGEL2	AMIGO1	ZNF334	PIK3C2B	PLD1
ZMIZ1	XIRP2	RAD51D	TXNDC11	STARD13	HMGXB3	ZNF839	TACO1	NCKAP5	AMBRA1	TCEANC2	RNF4
BLOC1S2	PJA1	ZNF593	DHX32	MIEF1	H4C5	ZBTB7A	RAB36	F2RL3	GRB10	PEG13	INKA2
AFG3L2	HMBS	RBM5	ARHGEF10L	RAB30	NKRF	LINC01569	YLPM1	DYRK2	AHCTF1	PRR15	DERA
KIAA0232	CCDC120	SCO2	JPH2	MTHFR	NSUN5	PLAGL2	TBX5	LINC01000	MKRN2	ZSCAN30	PPARGC1B
CASZ1	FOXO3	RLF	PHACTR1	SLC31A2	B3GALT2	ZBTB4	ENOX1	NAV1	LSM2	TRAF2	TRIM11
RIOK1	ZNF233	TBC1D25	HUNK	TRAM1L1	PPP1R3C	IRF2BP2	EFNA1	CTNS	TRIM47	KIAA1586	PSMG3
KAT6B	ZNF607	ARHGEF3	TNFAIP8	THYN1	MC4R	KDM4B	NR2F6	GOLPH3	ZMYM1	TNS3	FAM241B
ZMAT4	CREB1	RFC1	ADPRH	ESRRB	HDDC3	RTN4R	ANKRD1	GLI3	SPATA13	PRDM16	C15orf39
ZNF609	BCL9	FEM1B	RTL6	POLA2	MBD5	MIR22HG	POGK	FBXL19	GLCCI1	RASSF9	ANKRD50
WRAP73	POLRMT	STON1	SMURF1	SPRED2	HR	NAB2	STBD1	ZNF280B	MSRB1	ZNF438	KRT10
ZFP37	SNPH	CCDC8	TAF4	RNA45SN2	PCDHA9	ABCG1	ZNF770	RGS3	WDR43	XYLT2	KCNJ3
IRF2BPL	ABL1	DIRAS1	LINC02693	PLPP3	GCNT2	PRSS44P	ARID3A	ADRB3	C11orf68	PDCD10	NEURL1B
CPTP	PRKD1	EBLN2	ROR2	MIR27B	GSPT2	ARMCX4	SRCAP	RORA	ZNF581	PLEKHA5	MSH6
SKIDA1	RCOR2	TRIM32	ARHGAP24	NUDT4	TCF7L1	SMC3	SOGA1	NUP153	FAM117B	NAA80	RFXANK
RGL1	TRAF3IP2	NIPBL	FOXK2	TPR	AFAP1	DCLRE1A	PITPNA-AS1	HAND2	C1orf226	C6orf136	PLEKHA2
ITPKC	VPS37B	ZNF146	ZNF780B	ORC5	GEMIN6	PRMT9	INKA2-AS1	ARF6	SH3BP4	CLUHP3	TRIM24
FUT10	VPS37C	KIZ	ARHGAP27	PPP1R26	TENM4	TTC3P1	RAB11FIP5	AKNA	MIR133A1HG	MDFI	SRGAP1
PTPN3	CCNQ	EXOSC9	LRRC8D	NUP85	LRRC75A	GEMIN7	GIHCG	MLLT1	UBTD1	MALSU1	VPS37D
ZFPM1	ARMCX5	INCENP	INSYN2B	EIF2B4	ZNF260	FAM53B	ARHGAP31	PCCA-DT	MADD	PHC2	RASSF5
NELFA	SPOP	NHLRC1	JMJD4	FAM13C	CSRNP3	NFKBIL1	PLPP7	ARL14EP	MBOAT1	SLBP	FAM122A
FRMD5	KRBA2	PWWP2B	CHRM2	ZNF879	BRINP1	MYOCD	KAZN	ZNF285	GDPGP1	RNF152	ZNF672
VGLL4	RASA1	PANK1	LINC00957	GRID1	RNF43	WAPL	UBOX5	HOXB4	MIR145	LMO4	DUSP14
ARFGEF1	USP12	ARHGAP39	YEATS2	FRMD3	RPP38	WAC	RAB42	DNAJC14	MAP2K7	KCNJ12	NSRP1
MIR210HG	GSEC	CCDC87	AHDC1	CEP295	PWAR1	UCK2	P2RY14	CHMP6	MIS18BP1	CNOT2	MOCS1
ANKRD34A	PPARGC1A	TANC2	CYTH3	NANP	CCNJL	ZNF671	NUP37	DNTTIP1	TFB1M	ZNF45	HEYL
KDM4A	EDA	ZCCHC24	FAM110B	BAHCC1	FITM2	BACE1-AS	COG8	PYM1	FLRT2	USP7	LRIG2
CDK8	DAPK1	WDR70	MGAT3	SLC25A37	ARRDC1	FYN	TAOK2	ZNF480	ABHD6	RGS6	SDHAF1
TBX20	BCL11A	ZNF169	CDH20	TIAM2	ADNP	BTBD8	OGFR	LOC101928961	COG2	LOC339975	TNKS1BP1
TIMM9	SYT9	XKR8	KDM6A	B3GALT6	SUFU	ZHX3	RPUSD1	ZNF638	PPWD1	ZNF484	SLC38A2
SMAD3	ZNF697	NCK1	PDS5B	ZFP64	FBXO42	ZNF71	SPRY4	SH3PXD2B	PEX12	STK11	PTPN9
SMTNL2	ST3GAL1	ZNF331	DCAF1	SAC3D1	RD3L	ZNRF3	RAB20	ZNF775	MYPOP	FGL2	FAM174B
LINC01182	ZNF746	PRRC2A	CSTF1	B3GNT9	PABPC5	TRIB1	KLF16	ZNF512	STK17A	MAGI1	SRGAP2
RIMS4	ASXL2	KCNA4	TICAM1	KCNJ8	A4GALT	MIOS	MRPL36	ARHGEF18	E2F8	FZR1	GJA5

ZNF572	NPY6R	LIPT2	PHLDA3	FADD	SSR4P1	THAP8	KIF26A	COA7	SRC	GPRC5B
ZNF521	KLHDC8B	TRIM16	SKI	ZMYM3	ATG4D	TAS2R19	KMT2C	NUDT18	LRRC10	HTATSF1P2
MAST4	ZCCHC3	FOXO6	GCK	MED18	SMARCAL1	DISC2	PDE1C	ABL2	ZNF382	PCARE
TAS2R30	OSGIN1	PAQR4	CMKLR1	SAP30	SPTLC2	WNT5B	DNAJC17	AHNAK	MIR1-1HG	MEIS1
LOC100506178	PPP4R3A	MCAT	ANKRD63	TSC22D4	FZD4	TRIM56	ASB16	C8orf58	DNMBP	DHFR2
ZNF112	CARMN	BICRA	F8A1	PAFAH1B2	SMIM38	JADE2	INE1	LDLRAP1	YPEL2	LINC00339
ZNF69	GTPBP2	BAK1	FAM20C	RRP1B	GTF2E1	ZNF579	JAKMIP2	WIPF2	RPRD2	TRIAP1
KAT5	C10orf71	POP7	BRWD1	RTKN	ORC2	CUL1	C8orf49	LOC374443	MIER2	SLC45A1
ZNF74	ZSCAN20	NUMA1	GPSM1	EXTL1	MIR29B2CHG	PPP1R3A	MINDY1	ZNF780A	MTCL1	BCORL1
IWS1	ZBTB20	TMEM102	PIP4P1	ZSWIM1	ASXL1	ZFP82	LMNB1	SNCAIP	GMPS	ZNF221
NIPAL4	APOL6	KIF26B	LOC101928841	IQSEC1	RIC1	ZNF865	MIR133A2	RHOU	GAREM1	SCYL1
USP35	MUS81	ABTB2	GIGYF2	CXXC4	ZNF292	REPS1	TLCD5	RBMX2	CACNA1C-IT3	EPN3
FAM78A	LURAP1	ZNF287	KLHL9	CHMP7	IFNLR1	FAM50B	NOS1AP	MAP4K3	PIGBOS1	ANKRD44
MIDEAS	MMACHC	MAEA	RALGDS	KAT14	UBE2O	GATA5	NR2C2	PPP3CC	UBE2CP5	GATA6
ITPKB	RNF220	EP300	SQLE	ZNF776	RELA	CCDC85B	FIGN	AKAP3	IFIT1	UBR5
TFEB	ZNF70	SHB	ZBTB34	MAML3	RFXAP	ZC3H4	MAP3K9	PGBD5	CLMN	GIT2
TBK1	MYO9A	SRSF9	ASB13	ZNF576	PDIK1L	ADCK2	TACC2	URGCP	ESS2	ADAMTS15
ETFBKMT	GTF2IRD1	VCAM1	TOB1	LOC108783654	RAP1GAP2	C1orf74	CTDSPL	EPHB3	DISP1	CREBBP
MYO10	MICAL3	NFKBIA	IRF2	KCNJ11	TEFM	ZNF787	OBI1	KY	RTN4IP1	B3GNT8
CTTNBP2NL	SPRY1	ASTN2	CCDC127	GJC1	TRIM68	PHF21A	MAML2	CMTR2	HRH2	CRTC3
BCDIN3D	RFX7	TADA2A	NFATC2	HDHD3	ZNF613	RXRA	TBILA	KEAP1	GDF2	MTSS1
EGLN1	TIGD5	HCG11	PELO	FOXK1	RNVU1-14	PDCD2L	TPPP	TFAP4	TSPYL5	SFMBT1
KLF13	IRS1	NUDT16P1	GRAMD1B	BMP2	JRK	LARP6	CNOT6L	TMEM131L	NOL8	INTS13
CDCA4	ZNF212	INTS5	DDI2	BNIP1	LINC01547	COMMD4P1	MAGEF1	CCDC71	SPSB4	DUSP27
BLID	KCTD21	TBC1D14	NECTIN1	TSHZ1	TRIM65	CLEC2D	AKAP13	RIPOR2	KCNK13	ZHX2
ZSCAN22	ZSWIM5	C6orf47	LINC02691	DBP	BAZ1B	EPHB4	TIFA	BHLHE40	ZNF483	NDST1
EPB41L4A	LOC339803	PFKFB4	PRSS42P	ETS2	RCSD1	TNFRSF1A	SEMA6D	MGA	ZNF792	TMEM51
S1PR3	KIAA1671	MVK	PHF3	TNFRSF19	ZNF30	STIMATE	DGKK	ZBTB45	PIM3	TOR4A
ESPNL	ANKRD17	RPTOR	PPP1R3B	LRCH1	LIMD1	E2F3	PUM2	ABHD8	B3GALT1	APC
CDCA7	SNRNP35	RBMXL1	FOXP1	TRIM63	GALNT18	IP6K3	RUNDC1	ZNF449	NAT8L	C12orf10
ZNF827	ZNF148	COP1	MYADML2	ZFAND5	POLH	SLC25A21-AS1	BBC3	ZNRF2P2	C17orf97	MRPL15
RABIF	ARNT2	NHSL1	SLC22A23	FBXO34	HYLS1	PDZRN3	TMEM115	KCNV1	LOC646762	BCL9L
LUZP1	FAM102A	SPRY4-IT1	NAV2-AS2	IRX1	PARP2	GATAD2B	PARD6B	SLC16A13	TRPS1	RAB35
ADORA1	BMPR1A	C19orf44	ISCA2	ZBTB12	AKAP6	ZW10	MIR208A	TCHP	MITF	ARNT
FPGT	RNA45SN3	TADA2B	N6AMT1	ZNF608	BRF1	ZNF584	COIL	PAG1	SEC24B	ZNF395
ASB2	SH3TC2	UBR2	GLI2	BICRAL	NOB1	LRRC61	GGNBP2	PRR12	PDLIM4	TNFAIP8L1
NACAD	C17orf58	AATK	ZNF501	CXXC5	ZNF616	BEND5	SMIM29	ZNF22	RAB33A	CHCHD4
PPP1R13B	SOX12	RGS19	ELFN1	CHSY1	DUSP18	SRSF4	ARHGEF2	CNN2	ZNF658	MARK3
SH3RF3	ZNF764	SIK2	PLEKHG1	NKX2-5	LOC100507516	DHRS9	FGF2	KIAA1217	GPATCH11	ZNF583
LHFPL2	ARMC7	DUSP7	ZFP30	NKX2-6	PIK3R1	VANGL1	KLHL30	DTX3L	THNSL1	PRPH2
MRGPRF	CIAO3	FAM210A	SNORA71B	USP2	GPRIN3	SALL2	C14orf132	SHROOM3	LINC00649	NCOA5
BRF2	PDE4B	ELL	FOXJ2	ZFP62	KCNJ4	PPP1R35	MVB12B	FOXO1	NAPEPLD	ZNF658B
LOC100506688	MRPS31	DFFB	MTFP1	MARK2	PALMD	FRMD4B	SPATA2L	IFFO1	PRICKLE2	KRT19
EDAR	TGFBRAP1	LRRC42	VPS72	MAVS	SAFB	MOB3A	RALA	POLR1B	PNMA8B	MAP2K5
SPRY2	SGMS1	MRPL9	KMT2E	GKAP1	ZBTB11-AS1	BRINP3	CHST3	POP1	KLHL3	FAM107A
LOC100287896	CCDC102A	PSMG2	ZNF182	HRK	PMP22	MEIS2	FAM124A	PEX14	LOC339260	ZNF559
LIPE	RCE1	ZNF552	UNC5C	LMOD2	ZNF784	CCDC107	MAST3	WDR24	TMEM99	POLR3B
JCAD	TRAF6	QRSL1	NAV2	THUMPD2	PROSER2	TET3	ZNF354C	ACVR1B	KLHL38	
DOK7	LOC100652768	UBE2QL1	SLC2A1	RARA	NUP98	RDH13	ZFP14	AMOTL1	SASH1	
ZNF205	MTERF3	MIR4697HG	ZNF747	DPF3	KCNE4	BSN	RAB9A	ARL10	MT1E	

ACLA											
LINC00987	PKN2	CLIC1	SLC35E2B	MYPN	B3GALT5	MYO1E	INTS2	SLC2A12	SPEN	FAM222B	LOC101928126
SACS	PHF20	PHF14	SETDB2	CES2	RNF24	NHSL2	ALKBH8	ZNF444	METTL11B	PPP2R5E	FRMD4A
PDZD2	SBNO2	DIP2B	ITSN2	ZZZ3	B3GNT10	TRIM33	KIF13A	C6orf163	MRPL14	TRIM54	DKC1
LOC1005071	LINC00382	TRERF1	MOB3B	GATAD2A	PRXL2B	FBXO27	FN3K	ERBIN	MLX	KIAA0825	CCBE1
SNHG18	BDNF-AS	MIR10526	NCOA2	PTPRK	USP34	KIAA1549	IFI16	NBN	MACROH2A2	LRRC75B	PLEKHM3
GBF1	RPP40	JADE3	DOCK9	TMF1	RHBDF2	CASK	SLC29A2	RBMS1	SLC27A3	LYSMD1	ZFHX3
TAFA4	MEAK7	DENND2B	CASP8	PAK4	CRYBG3	ESRRG	KCNJ5	IGSF9B	PXYLP1	TAS2R13	PRUNE1
PPP1R37	ARHGAP18	PARN	REXO4	PMEPA1	LRFN3	SMIM8	RASA3	TXLNB	TAS2R50	ARHGAP32	CORO2B
PARD3B	TMBIM1	GSE1	METTL5	MACF1	ZFYVE28	GAPVD1	TTLL1	GDPD1	KANK2	LRRC4B	KIF1B
LZIC	APOL2	BACH2	MARCHF11	TCF12	MIR99AHG	MIR100HG	GTF2F2	DHX57	SEPTIN9	SYNPO2	TRIP4
MLLT10	NGLY1	LRRN2	CLINT1	SH3GL1	SNX21	USP48	N4BP2	RAP2C-AS1	MAU2	TANGO6	TCF7L2
CMYA5	COPB1	ANKEF1	ELP6	ST6GAL1	SHROOM4	KRI1	MPG	BIRC6	ARHGAP44	WDR59	IGF2BP1
GOLGB1	EXOC6	DOP1B	CWF19L1	SIPA1L3	THUMPD3	TMEM164	CBR1	TBC1D4	KPNA3	OXTR	LRRN3
WDR33	ARAP1	MTX1	C4orf54	TRIM6	KLF12	STAG2	PCYT1B	DHX16	FLYWCH2	IGF2BP3	DIP2C
IKBKE	MSS51	EXOC6B	NCALD	PREB	EXD2	STRN3	SLC46A3	KLHL22	OFD1	SNX29	TUBGCP3
NCOA3	KCNN1	ZDHHC5	ZNF346	SMC4	ZNF767P	ANKRD11	NSMCE4A	MRPL27	CACNG4	PRSS43P	WWP2
SSH1	DACT3	STX18-AS1	BRD4	USP22	ATXN1	CYB561D2	LINC01061	NPAS2	SCN2A	ARHGEF11	IBTK
MYO1B	REV1	NCOA6	TSNARE1	CDK6	LINC01449	CHRM3	TANC1	LDLRAD3	NEDD4L	TFCP2	ARHGAP6
TAMM41	PREX1	WDFY3	LINC01224	FOXN3	SLC25A12	RFX1	FAM160A1	SMARCB1	ETV6	C1orf21	ART4
KCNN2	THRAP3	DHODH	MAGI3	INTS14	ANKS1A	LINC00260	HDAC7	GRK5	ROR1	SDSL	PLEKHG4B
ZNF362	PBX1	MYCBP2	TIAF1	EMP2	ASF1A	PDPK1	NUMB	SERPINE1	PPARA	DLG1	RBMS3
UBL4A	KIAA0355	DDX60	EFL1	OSBPL3	TNRC6B	MARCHF1	KIAA1958	METTL15	MCRS1	PITPNM2	ILVBL
PTPN4	HIP1	GPSM2	POLM	GPR22	CDH10	FGFR2	DMXL1	FCHSD2	RNF2	LOC10041958	TMCO4
NYNRIN	BMS1	MYLK4	ZMYND8	NPR2	ANKRD28	KDM2A	CYTH1	NCOR1	CDC27	MAST2	PPP1R12A
PEAK1	ERMAP	FAM102B	ARHGAP28	IPO8	SH3TC1	FUT8	PRRG3	CSNK1G1	EFCAB7	ARFGEF2	SMARCAD1
PIP4K2A	XPO1	FANCC	MTSS2	UBE2H	NCOA1	POPDC3	GTF2I	KDM4C	SAMD10	ZNF280D	SPATS2
LCAT	CHST11	PTBP2	IL4R	ALKBH3	CTIF	DET1	RHOBTB2	PCM1	IFIT3	SLC18B1	DAAM2
MEF2C	LOC1001303	FNTA	SPIRE1	CLMP	CHRAC1	BAIAP2-DT	TMCO6	CHCHD6	BICC1	FAM189B	SPATC1L
ST3GAL2	HDAC4	BTBD9	SLC39A3	RBM20	TMCC1	UNC5B	UBXN10	CTDSP1	C1QTNF1-AS	PPP1R9B	LOC729737
CTC1	KALRN	RASAL2	VAV2	LGR4	PRKX	NF1	SIMC1	THAP4	PLEC	TBL1X	CACNA1D
USP32	C12orf4	PARD3	LARGE1	NNT-AS1	FRY	GNA11	CHSY3	NF2	CUL3	TOMM40L	MECP2
NFIB	YEATS4	C14orf180	CARMIL1	EVA1B	HIKESHI	PHLDB2	RPS6KA4	WIPF1	RALGAPB	KIF2A	RSPO3
MYRF	KIAA1328	ABCF3	NXN	TOP3B	UST	FSD2	PDS5A	ZNF33B	PRKG1	PRDM11	IGF2BP2
LHX4-AS1	DHRS13	FBXL20	CLN8	MIR1-1	RRBP1	MTRES1	USP6NL	VPS26B	TP53BP1	USP25	PRPF19
UBE2G1	APOL3	CCM2	SNUPN	STRADA	INTS6L	CDKAL1	REV3L	SLC25A48	MRPL22	SPHK2	PLAA
RCOR1	DNAJA3	HRCT1	CGNL1	RHOT1	KIF16B	ZBTB40	SLC22A15	SYNE1	MPP7	RNF180	PTPN14
LOC1001296	EGFR	CTBP2	TMUB1	ARL15	MOB2	C6orf223	XPO4	CHCHD3	SLC9A3R2	NOD1	USP10
LOC257396	C2orf74	UBASH3B	CMAHP	PBX2	HDGFL2	PAFAH1B1	EHD4	SRSF1	DLGAP4	DPF1	TRIM2
PPP2R2A	ARCN1	CRBN	RNF216	PGPEP1	DCPS	CCDC85A	FLJ44635	FXN	KCNE3	PPP2R3A	RRP1
OBSCN	RIOX2	AFF3	CREB3L2	PKP2	DGKD	RNF214	ABLIM2	CAND2	DGKG	TRMT1L	TMEM168
ARRB1	KIAA0513	KIF7	CDK4	POT1	VSTM2L	AARD	NCOR2	POU2F1	MBNL1	IFT172	AGAP1
SH3PXD2A	HECTD3	TAB1	BORCS5	PTEN	NUDT12	EEA1	CHN2	WWC1	KCNQ5	RSF1	TRAPPC8
TAF3	FNIP2	LMOD1	PDE1B	TRAF3IP1	SMG9	ZBTB37	TMEM240	MPHOSPH6	HNRNPD	SYTL3	UBXN7
ARHGAP21	DTNBP1	TRDN-AS1	MRTFB	FZD4-DT	PLSCR1	TMEM70	TNKS	FERMT1	PNMA8A	SH3BP2	DLEU1
PRKCZ	CAPN7	ARHGAP10	SCN7A	CDH11	C10orf71-AS1	MARCHF3	CALCRL	NUDT19	COQ8A	COX19	STAG1
ORAI3	RBPJ	BTRC	SHROOM2	DUSP19	RAB3A	RAB3D	ATXN7L1	TTBK2	SERAC1	ELOVL6	VKORC1L1
KRBA1	LRRC27	NAA15	STK24	SERTM2	ZNF704	RASL12	LIX1L	HS3ST3A1	MTUS2	MTMR12	CCDC58
EXT1	TOX	SCN8A	DCLK2	GPHN	NUP210	BRD3	MCC	TRIP6	CAMKK1	PLXNA2	LMOD3
RIN2	LINC02082	PBX3	SYNE1-AS1	HOOK3	SRL	ALPK2	CNOT1	SETBP1	RHPN1-AS1	PPARD	TNFRSF12A

PLEKHA7	TTC28	GABRB2	TCF4	PPFIA1
AK6	RAD50	TLK1	TRIO	LRRC39
ZBTB42	ABCD2	MRPS18A	SNX9	RAPGEF4
ATXN2	LINC01411	RIPOR3	PIP5K1B	GNB1L
AGGF1	SLC45A4	CRTC1	DPH1	ACOT11
ARID2	OSBPL5	CLPX	MRPS26	CANT1
SAMD12	SOS1	OPHN1	VPS13D	NAV3
TEAD4	BOLA3-AS1	EBPL	TUT4	MRPL17
FAM193A	NOVA1	USP28	CRLF3	
PKNOX2	FBXL7	PSMG3-AS1	GNAQ	
ZFYVE9	PPP2R2B	EIF2AK4	PLPP1	
TAOK3	SCN2B	MPPED2	RNF150	
HDAC9	MTIF2	SYDE1	LMO3	
SMURF2	BCR	ANO4	TAF8	
DIRAS2	SAMD4A	JAG2	LPAR3	
NINJ1	PHF20L1	ARTN	BBX	
PRIMPOL	GTF3C3	SLC20A2	KIF25-AS1	
POU5F2	EHMT1	GAB1	DNAJC30	
SIK3	PIK3CB	ANKRD6	NACC2	
ARHGAP42	CERS4	INPP5B	CMTM4	
RIN3	PRKAG2	POU6F2	EMP1	
EDN2	DNAJC2	SIPA1L2	EIF3A	
PPME1	SPEG	C3orf18	BAZ2B	
CMTM7	AKAP5	RSBN1L	EEF1AKNMT	
EEFSEC	PDZRN3-AS1	LOC1079852	ADGRL2	
MCF2L-AS1	DENND6A	PIN4P1	FAM207A	
ARHGAP26	BICD1	SAMD9	MDN1	
PLCXD3	MAPK4	MEGF9	PDE10A	
SNX30	RANBP2	DRG1	GRIP1	
EDRF1-DT	LYPD6	CPT1A	IRAK1BP1	
WSCD1	NFIA	BMPER	AAK1	
STXBP6	TIMM21	SLC26A9	UBE2R2	
GPR1	HMBOX1	EDNRA	SETDB1	
SYNPO2L	PRKCE	DOK5	MYO18B	
HNRNPUL2	ACOX2	AGFG1	SF3A3	
UBAP1	SIPA1L1	MSH3	MRPS18B	
DOCK4	IL1R1	RAVER1	PWP1	
LOC643339	CCDC43	ERC2-IT1	ROCK2	
CTNND2	SLC35F6	TMEM161B-A	HECW2	
H1-0	GTF2IP1	STRN	FAM71F2	
XYLT1	REPIN1	FER	APCDD1	
ZMYM4	SGPP2	MAP7	HEATR3	
AGPAT4	HPSE2	CEP290	MRPL45	
EPB41	FARSA	RAI14	LSM10	
ZNF415	TRMU	AREG	NSUN4	
CMIP	ENOX2	LRP6	TRAPPC11	
TSPAN5	APOBEC3G	TNIK	MED13L	
ACAP2	KCND3	ARHGEF16	FRYL	
UFL1	FBXO11	ZFHX2	PSMC2	
MAP3K5	DENND4C	ACBD6	SSBP3	

DOXO											
RASL11B	RUNX1T1	H2BC18	FBXL3	AKIRIN2	TP53BP2	NDST2	LMCD1	KLHL21	AEBP2	GNL2	UBE2J2
ADNP2	RNF111	LTV1	INAFM2	NDEL1	SAP30L	BBS10	MUL1	SPATA2	H2BC8	KDM5B	MED17
RASSF1	KCNA6	DDX10	ZNF774	MTERF1	ZNF546	SLC25A29	FBXO46	LYAR	H3C4	ZNF24	ZNF414
ZSCAN32	KLHL25	GFER	ZNF41	ZNF669	H2BC15	ZBTB39	MRM2	OXSM	TTN	ID3	MIR938
CHERP	GRPEL2	SOCS6	ZNF426	ATG3	LINC00852	ZNF440	TERF2	PARS2	ZNF324B	JUNB	ASB7
ZNF181	KIN	EFNB2	NEDD9	PIK3R3	VCPIP1	FAM111A	FBXL12	SOX11	TMEM121B	MIR4506	APLN
ZNF320	ZNF526	ZNF425	FIP1L1	DAXX	LDB1	MIR421	ANKRD49	ZNF708	TMEM200C	PER3	FAM83G
WAC-AS1	IFIT5	ZNF740	MT1X	GCLC	RLIM	PHF13	TRAF4	SOS2	ZNF93	MEPCE	ZNF7
TP53INP1	OTUD1	ZNF12	DYRK1A	ANKRD27	AJUBA	NFX1	RNF139	ZNF844	CLP1	RANBP6	MYORG
ZSCAN2	H2BC5	H3C11	CASTOR1	GNE	NAIF1	POLR3D	ZNF627	PGBD2	HES1	C19orf48	HERPUD2
THAP3	ZKSCAN8	DUSP6	GADD45A	ZNF790	SF3B4	TNRC6C	RBBP6	PLEKHH3	SP3	ERCC6	GRWD1
FAM118B	TIGD2	TPRN	BAMBI	KBTBD7	FAM200A	ZFX	NET1	PYGO2	C1orf35	VPS18	KCTD7
MSX1	PIAS4	RPL39L	PAQR7	RAP2B	RBM23	DCP1B	SNN	OTUD7B	ZBED5	WDCP	ZBED9
KCTD6	DDX20	TAF7	TRAPPC2B	H2AC17	SLC35C1	ZNF319	ELF2	KLF15	ZNF354A	ZNF561	ZNF398
ABRA	ZNF564	C18orf21	TMEM223	NOA1	TAF5	ZNF823	ARRDC3	RBM14	RAB32	DEPDC5	ZNF19
AKAP1	HAUS3	TRIM62	ABRAXAS2	TPRA1	MTERF2	H2BC6	BCL2L11	LASP1	MBIP	E2F6	KBTBD8
KBTBD13	RNF138P1	GABPB1	TOGARAM1	KAT6A	ZNF667	MSANTD4	CTTNBP2	SMARCD1	JUND	ADPRS	PLEKHF2
SERTAD1	CLBA1	B3GNT2	GMEB2	SERTAD3	JMY	CEP19	MACO1	EED	TBC1D10A	ELOVL1	ERF
ZNF189	TGIF2	ZSCAN12	LBH	GPANK1	ZSCAN23	DUSP5	SIAH1	CYB561D1	FAM13A	KCNQ1OT1	URB2
ZSCAN21	IFFO2	TGIF1	DIDO1	MACIR	ZNF485	ZNF668	RNF216P1	LONRF1	FEM1C	FAM217B	FBXL18
ZNF699	AMER1	H3C1	EID2B	YRDC	C19orf81	ZNF44	PURB	PEX11B	ZNF250	CDKN1B	SH3RF1
DDX31	BTG2	CALHM5	ZNF275	BOK	NIF3L1	UTP23	ETS1	SETX	H3-3B	PPP1R10	MSL2
PCF11	KMT2D	PPP1R8	ZNF540	LRRK2	S1PR2	ZNF749	ZNF439	INTS6	UBALD1	GPR37	TRIM35
SUPT20H	GMNN	CDAN1	PAQR8	CNKSR3	VIRMA	ZNF142	C11orf95	FAM43B	MPHOSPH10	ARID5B	TWNK
ZSCAN9	CUTALP	ZNF778	ELOA-AS1	TENT4A	NCBP2AS2	CEBPZ	ZNF430	ZNF34	ID2	C1orf216	FAM110C
NOC4L	NUFIP2	RBM7	GTF2B	RAB7B	TOB2	LOC105371592	MIDN	C1orf229	ZNF837	BHLHE41	NOG
TNFAIP8L3	ZBTB9	CCDC117	ZNF586	PFKFB3	C18orf54	ZNF16	FAM43A	LSM8	HMGXB4	WDR3	ZNF606
MED26	UNC50	ITPRIP	ZNF589	PDP1	ZNF490	KLF10	ATAD2B	HSD17B7	GTF3C4	PPP1R18	NXT1
TK2	SLC52A2	TTI2	JRKL	TBP	WDR25	ISL1	ZNF223	DNAJC18	RNF144B	SLC25A25	SIX5
ZNF436	ZNF555	SLC35E4	ZC3H10	NRIP1	TSEN54	PSMA3-AS1	DUSP16	BMF	HYMAI	INIP	MAP3K14
TUFT1	ZNF266	ZNF318	CGRRF1	POGZ	RRP8	CWC22	ZNF131	WDR5B	ASTE1	ELAC1	GMEB1
MAGI1-IT1	ZSCAN16	SLX4	ZBED4	USP27X	FAM171A2	ZNF235	CWC25	SRSF3	MIEF2	C16orf72	PITX2
NR3C1	MED13	PATZ1	ZKSCAN4	BCAR3	DTX2	BLOC1S3	ZKSCAN3	ZNF491	ZBTB3	KMT5C	LEMD3
PABPC4L	ZNF396	ELF1	ZNF211	NFYA	IMP3	ZFC3H1	CDK12	FLRT3	CRK	MAD2L1BP	PIGC
PAK1IP1	PARP16	SAYSD1	CYP2R1	NOL11	TENT5B	AUNIP	PNRC2	MIS12	ZNF703	DKFZP586I1420	GOSR1
TEF	ZNF43	DDIT4	H2AX	ZNF566	TRMT12	KDM7A-DT	PROSER1	CDR2	DUSP10	PLEKHG3	ZNF587B
PTRH2	FZD1	ZBTB38	ZNF222	SOX7	NFRKB	R3HCC1	ZNF845	ZBTB24	LRRC8E	XBP1	ZFP36L2
ZNF891	ZNF682	OSER1	NAT10	JAGN1	VIPAS39	H2AC6	JMJD6	ZNF680	DNAJC28	NOP16	NR3C2
ZC3H11A	ARHGAP29	DCUN1D3	ZSCAN25	PUS3	ZNF232	ASAP1-IT1	ZNF524	H3C13	PCDH18	RESF1	ZNF836
TBX2	PDE12	HMGCR	LINC00654	H3C10	EXOSC4	TIMM22	PLEKHF1	LINC00472	TAPT1-AS1	EEPD1	HSF2
CCDC184	ZNF410	ZNF195	MAML1	PDE7A	PGBD1	TRIM26	RHOF	ZNF502	EDRF1	ZNF496	MYNN
TFPT	PAOX	MB21D2	MICOS10P1	OTUB2	RIOX1	SNIP1	BEND3	EBLN3P	FBXO30	PI4K2A	HSPA14
ING1	ZNF416	LINC01460	GPR183	WDR37	TCF20	RFWD3	TMEM37	MAGEE1	WTAP	EIF3J-DT	ZNF724
ST7-AS1	LOC105371763	ZNF180	CHD7	MCL1	EFNA4	EGR1	ZNF574	TSC22D1	WDR53	BLCAP	GPATCH3
ANKRD37	GAB3	ZBTB21	AASDH	IKZF2	PIKFYVE	LNX2	KNOP1	MEF2D	ZNF254	MIER3	MOB3C
DOK1	RIOK2	DMRTA1	PCGF1	RBM4B	RNF146	STC2	HROB	FBXO45	FAM181B	GORAB	BRD2
EPM2AIP1	LINC01996	PXDC1	ZNF772	IER3	ZNF660	AVPI1	FKBPL	ABT1	ZNF506	SPSB1	NRF1
LINC00526	FAM155A-IT1	KCNQ5-IT1	BORCS6	KBTBD4	FAM155B	SOWAHC	H4C1	LINC01355	NECAP2	ZNF687	EXOSC3
KBTBD6	KRCC1	ZNF174	JMJD1C	NUAK2	TMEM11	SIN3A	CHAMP1	PGBD4	ZNF518A	CAMK1G	ZKSCAN1

ZXDC	MTF2	ZNF2	ZNF230	ZC3HC1	SCAND1	ZNF432	ZNF768	CCNF	RNF25	TRIP11	DIPK1C
SESN2	FOXO3B	ZNF786	LCMT2	ZNF214	ZNF317	SLC66A1	GCC1	ZNF443	FICD	ZNF625	TRIB3
SNHG30	ADAMTS5	PHF23	PHETA2	AXIN1	ZNF350	GPRC5C	FGF18	H4-16	CTU1	SLC35F3	CENPO
ZNF799	RBAK	LOC100506472	ZNF503	FRG1	EDC3	MID1IP1	LENG1	CCDC121	RPL23AP32	PLEKHO2	TSC22D2
MIRLET7BHG	DAND5	XAB2	TIMM29	SIAH2	SESN1	MED19	LYSMD4	MFSD4B	POM121	ARID4B	MED31
RPP14	EAPP	ZNF777	TMEM201	EXO5	DCBLD1	ZNF473	ZNF689	LOC101928279	ZNF567	IER2	MIR34AHG
ZNF830	ZNF493	TLE3	C16orf70	PPRC1	ZNF441	DNAL4	MAD1L1	DVL2	ZNF84	WDR5	MSX2
HELZ2	KMT2A	EXOC8	STX5	CHST14	ZNF101	ZNF417	ZBTB22	ZBTB10	ZNF431	GCNT1	PPM1D
H3C6	ZNF442	DOLPP1	NFKBIE	AEN	PHLDA1	ZNF791	KATNA1	NUFIP1	AP5B1	DAPK3	ZKSCAN7
RBM12B	ZNF14	ASB6	BET1L	PAPOLG	ZNF615	YAE1	TIMM10B	XIRP1	KANSL2	N4BP2L2-IT2	ZNF281
LINC01770	MAPK7	PHF12	ZNF304	CLK3	RIF1	DOP1A	G0S2	THRB	TSPY26P	FIBIN	TP53RK
TOE1	DIMT1	EID2	SPRED1	FAT4	FBXO33	THADA	ZNF92	HMG20A	EPHA2	ZNF136	SLC30A4
MIR23B	H2AC14	TMEM186	ZMYM5	CTDSP2	TEC	SNAPC2	PLK2	ABHD17B	GIN1	TMEM138	TBL2
FOXD1	ZNF264	C1orf174	CDC42EP3	HEY2	SLC25A44	DKK1	S1PR1	MAP3K3	SOCS4	ZFYVE1	GVQW3
CLDN12	TMEM229A	SPSB2	TMEM251	HEY1	LOC284454	ZNF551	FKBP15	NEAT1	ZNF202	DBR1	ZNF394
MYLIP	BRIX1	IRX5	SGF29	RASGRP1	KCTD1	AP4B1	LRATD2	SGK1	ZNF598	PTENP1	POM121C
SMAD5	GZF1	ZSCAN26	CCDC174	NFKBIZ	DCLRE1B	NPAT	VEZF1	TRIM21	ATG101	KHNYN	CD2BP2
RBM22	ZNF728	PPP1R3D	RND3	MPV17L2	MIR181A1HG	MIR125B1	ENC1	DNAAF2	CCDC61	CBLB	NKX3-1
RBM15	GEMIN4	ZNF510	ZNF343	ZNF629	TEDC2	HAS2	ZNF543	ZNF35	FUT4	MPLKIP	ZNF808
SNX33	NFIL3	SPIN3	BTG1	ZNF284	ADRB1	TRIM61	C3orf38	TRIM59	ZNF649	PRXL2C	ZNRD1
ZNF619	NCBP3	CEP85	NRBF2	SLC16A1-AS1	LCORL	IRF2BP1	TIGD7	JUN	MYLK-AS1	CDC40	ZNF548
DPF2	FIGNL1	ZNF322	WDR81	TFIP11	NUAK1	ZNF507	RBM15B	LOC100499489	ZBTB48	DNTTIP2	TOPORS
TLE1	ZBTB7C	CDC42EP4	RAB11FIP1	ZNF140	ZNF134	DYRK3	ZNF653	C12orf65	KANSL1	LIN37	SMAD1
ZNF48	FANCF	ZNF610	TMEM203	ZNF138	PCTP	H4C13	TIPARP	DOHH	C12orf73	LINC00847	SNRNP40
LZTS2	ZBTB5	TGS1	ZNF628	CPEB2	MRM3	MC1R	SLIT2-IT1	THAP5	SNORA99	SEMA4C	ZNF100
ZBTB26	DDX28	LIPT1	ZNF570	CENPC	NFYC-AS1	TBCC	LRIF1	TENT5A	OTULIN	UBALD2	FOSL1
UBIAD1	ZBTB17	DUSP11	MED8	TTC30B	CCNT1	SNHG15	C7orf25	TTLL4	MAT2A	TSLP	TADA1
TLK2	FLJ37453	NUP214	ZNF248	B3GAT2	MAP10	ZNF274	BRPF1	PIGM	ZNF788P	TMEM187	ZMAT1
TTF1	ZNF736	IGHMBP2	LINC00880	ZNF256	ZNF654	TCEANC	ANGPTL4	KIAA0040	TIRAP	FZD8	RPP25
RIPPLY2	BAHD1	CBLL1	CCDC96	CBX2	SENP8	FRAT1	BCL10	JOSD1	N4BP1	KCTD10	IRF1
TXNIP	ZNFX1	TMEM170A	SYDE2	EMSLR	MAP7D3	ARID1A	ZNF557	ZNF17	DBF4	GPR157	LRRC4
ETAA1	ZSCAN31	TBX3	ZNF57	ZNF595	HPS6	EFNB1	MYRFL	KBTBD2	ARID5A	E2F5	SNHG7
ZKSCAN2	ZNF571	FAM104A	PLPP6	MTA2	HINFP	KDM3A	MEX3A	RPUSD2	C8orf48	PER1	CSTF2T
PALB2	KDM5A	ZNF781	KLF9	B9D2	CTDP1	ZBED8	SNRK	PIK3R4	CTCF	H2AC8	ZNF563
METTL18	FAM120C	DEPP1	C12orf66	ZNF217	ARL4D	RNF113A	IST1	RCL1	TMEM250	WNT5A	PHRF1
FAM222A	FAM110D	PCIF1	CEBPG	ZNF852	CBX4	CIPC	RAE1	TRIM66	PPP4R2	FAM110A	DUSP1
NGDN	MFHAS1	CNKSR2	EPC1	ATP6V0E2-AS1	ZNF766	IRX2	MIR4523	SMIM12	RNF19A	TMEM51-AS1	EIF1AD
ZNF10	LEO1	LINC00909	TSPYL4	H4C11	UTP18	SOX4	FLJ42393	HNRNPA0	INSIG1	YTHDF1	EPC2
H2AC20	ZNF549	YTHDF2	MAP1S	BTBD3	ARSI	SLC38A9	EMSY	HIC2	IQCC	YARS2	SCAF4
TMEM60	GOLPH3L	DEDD2	ZBTB6	TBC1D10B	ZNF622	CCNG2	CNOT8	ZNF529	TMEM169	RAD51-AS1	PNRC1
IGIP	TRANK1	ZFP3	MAP3K1	CCNT2	ETV3	LOC100291105	MKNK2	ZBTB2	PDGFB	RNF19B	ZNF709
KNSTRN	IP6K2	RRS1	JDP2	TMEM86A	ZBTB1	CITED2	SIRT1	LOC100287036	ZNF562	HILPDA	TSSK6
RNF145	ZNF175	USF3	ZNF623	ZNF114	USP51	DLC1	ZFP36L1	NOCT	ADRB2	FILIP1L	PPP1R15B
ZNF620	MRFAP1L1	RAPGEF6	ZNF813	PWWP2A	MOCS3	UBN1	DNAJB5	SIRT4	KLF2	EIF4ENIF1	GPBP1L1
CEP131	ZNHIT2	ARRDC4	CDC25A	AXIN2	ZNF347	BUD13	CEBPD	AREL1	ZNF229	PHLDB3	FASTKD5
USP21	TMEM39A	GAR1	WDR89	BCL6	SERTAD2	SIRT6	TRIM4	ZNF486	MYCN	KLF3	PLAG1
ALKBH7	IER5	BAG5	ARHGEF40	MEN1	SURF6	FRAT2	SPIN2B	CDC42EP2	SASS6	RHOG	NYAP1
CDKN2AIP	SMAD7	FBXO32	SNORD116-28	TMEM200B	CCDC112	PHF24	ZNF718	DUSP8	NFKB1	CBX8	RNF169
NOSIP	CISH	RNF26	ADM	THAP7	ZNF408	MAMSTR	DOLK	ZNF793-AS1	RNF227	ZC2HC1C	CRKL
BORA	CRAMP1	ZC3H18	H2AC21	LOC100128288	STK35	THUMPD1	ZNF805	ZNF3	GEM	TRIM13	NKAPD1

Table S2: RNAseq_GeneOntology_Treatments_vs_CT, related to Figure 6

GO Terms_DOXOvsDMSO_Upregulated							
**ID**	**Description**	**GeneRatio**	**BgRatio**	**pvalue**	**p.adjust**	**qvalue**	
GO:0045047	protein targeting to ER	40/1035	116/18214	2.5E-21	1.3E-17	1.16E-17	RPS2/RPLP0/RPL12/RPLP1/RPS20/RPS24/RPL37A/UBA52/RPS11/RPS12/RPSA/RPL32/RPS19/RPL31/RPL41/RPL19/RPS8/RPS9/RPLP2/RPS17/RPS28/RPL30/SRP14/RPS5/RPS25/RPL36/RPL29/RPL38/SPCS3/RPL9/SRP9/RPS15/GET3/RPL18A/RPL39/SPCS1/SEC61G/RN7SL1/RN7SL2/RPS10
GO:0043312	neutrophil degranulation	75/1035	483/18214	1.4E-15	1.2E-12	1.06E-12	FTH1/HSP90AA1/CTSB/MAGT1/PSAP/ATP6V0C/RHOA/B2M/GPI/PRDX6/LAMP2/ATP6AP2/MIF/CYB5R3/DDOST/GNS/CTSZ/LAMP1/PGAM1/SRP14/GRN/ATP6V1D/PPIA/NCSTN/TRAPPC1/PGRMC1/TIMP2/TMEM30A/NPC2/CYSTM1/CST3/GAA/LAMTOR1/CREG1/S100A11/NEU1/HLAC/MGST1/HSPA1B/DNASE1L1/HSPA6/FUCA1/CD55/OSTF1/TCIRG1/STXBP2/GUSB/HLAB/CTSH/P2RX1/COTL1/SNAP25/GGH/PNP/QPCT/C3/VAMP8/CD68/BST2/AHSG/BST1/MGAM/FABP5/MME/CD14/ITGAM/ANPEP/ARHGAP9/PKP1/ADAM8/HK3/CHI3L1/CD93/PADI2/OSCAR
GO:0002446	neutrophil mediated immunity	76/1035	497/18214	2.1E-15	1.3E-12	1.14E-12	FTH1/HSP90AA1/CTSB/MAGT1/PSAP/ATP6V0C/RHOA/B2M/GPI/PRDX6/LAMP2/ATP6AP2/MIF/CYB5R3/DDOST/GNS/CTSZ/LAMP1/PGAM1/SRP14/GRN/ATP6V1D/PPIA/NCSTN/TRAPPC1/PGRMC1/TIMP2/TMEM30A/NPC2/CYSTM1/CST3/GAA/LAMTOR1/CREG1/S100A11/NEU1/HLAC/MGST1/HSPA1B/DNASE1L1/HSPA6/FUCA1/CD55/OSTF1/TCIRG1/STXBP2/GUSB/HLAB/CTSH/P2RX1/COTL1/SNAP25/GGH/PNP/QPCT/C3/F2/VAMP8/CD68/BST2/AHSG/BST1/MGAM/FABP5/MME/CD14/ITGAM/ANPEP/ARHGAP9/PKP1/ADAM8/HK3/CHI3L1/CD93/PADI2/OSCAR
GO:0000184	nuclear-transcribed mRNA catabolic process, nonsense-mediated decay	34/1035	120/18214	2.2E-15	1.3E-12	1.14E-12	RPS2/RPLP0/RPL12/PPP2R1A/RPLP1/RPS20/RPS24/RPL37A/UBA52/RPS11/RPS12/RPSA/RPL32/RPS19/RPL31/RPL41/RPL19/RPS8/RPS9/RPLP2/RPS17/RPS28/RPL30/RPS5/RPS25/RPL36/RPL29/RPL38/RPL9/RPS15/RPL18A/RPL39/PNLDC1/RPS10
GO:0019080	viral gene expression	41/1035	198/18214	3.7E-13	1.7E-10	1.5E-10	RPS2/RPLP0/RPL12/PCBP2/RPLP1/CSDE1/RPS20/RPS24/POLR2A/RPL37A/UBA52/RPS11/RPS12/RPSA/RPL32/RPS19/RPL31/RPL41/RPL19/RPS8/RPS9/RPLP2/RPS17/RPS28/RPL30/RPS5/RPS25/RPL36/RPL29/RPL38/SPCS3/USF2/RPL9/RPS15/RPL18A/RPL39/SPCS1/IFITM3/MDFIC/HPN/RPS10
GO:0045333	cellular respiration	38/1035	174/18214	4.7E-13	1.9E-10	1.66E-10	GHITM/CYCS/NDUFA4/UQCRH/PDHA1/SLC25A23/COX4I1/COX6B1/NDUFV1/COX8A/COX7B/NDUFA1/MDH2/COX6C/COX6A1/NDUFA10/NDUFB8/ETFA/PARK7/COX7A2L/NDUFA5/SUCLA2/NDUFB5/NDUFB3/NDUFB6/SNCA/CISD1/SUCLG2/NDUFA2/NDUFA7/PNPT1/MTFR1/ETFRF1/UQCRHL/MTFR2/GPD1/CYP1A2/UCN
GO:0006413	translational initiation	40/1035	193/18214	6.9E-13	2.6E-10	2.28E-10	RPS2/RPLP0/RPL12/RPLP1/CSDE1/RPS20/RPS24/EIF4G2/RPL37A/UBA52/RPS11/RPS12/RPSA/RPL32/RPS19/RPL31/RPL41/EIF2S3/RPL19/RPS8/RPS9/TMED2/RPLP2/RPS17/RPS28/RPL30/RPS5/RPS25/RPL36/RPL29/RPL38/EIF3H/EIF3I/RPL9/PAIP2/RPS15/RPL18A/RPL39/EIF1AX/RPS10
GO:0046034	ATP metabolic process	47/1035	295/18214	1.3E-10	2.8E-08	2.44E-08	GHITM/CYCS/ENO2/GAPDH/ATP5F1B/APP/NDUFA4/ATP6V0C/UQCRH/SLC25A23/ATP5MG/GPI/COX4I1/COX6B1/NDUFV1/COX8A/PGK1/COX7B/NDUFA1/ENO3/COX6C/COX6A1/PGAM1/NDUFA10/COX7A2/NDUFB8/PARK7/COX7A2L/NDUFA5/NDUFB5/JMJD8/NDUFB3/NDUFB6/SNCA/NDUFA2/NDUFA7/HSPA1B/ATP5MF/ENO1/UQCRHL/PKLR/SLC4A1/MYH3/NUPR1/GPD1/HK3/MLXIPL
GO:1902600	proton transmembrane transport	30/1035	145/18214	5.4E-10	1.1E-07	9.66E-08	ATP5F1B/NDUFA4/ATP6V0C/CHP1/ATP5MG/COX4I1/ATP6V1E1/COX6B1/COX8A/COX7B/COX6C/COX6A1/ATP6AP1/COX7A2/ATP6V1D/PARK7/COX7A2L/ATP6V0B/ATP6V1G1/COX17/ATP6V0E2/UCP2/ATP5MF/TCIRG1/SLC9A5/ATP2A1/SLC4A11/ATP6V1C2/ATP12A/PM20D1
GO:0043062	extracellular structure organization	52/1035	393/18214	1.1E-08	2.2E-06	1.92E-06	BSG/CCN2/APP/ITGB1/FKBP10/THBS1/COLGALT1/DDR1/HSD17B12/TIMP1/TIMP2/PLOD3/SPOCK2/CTSL/LOXL2/CST3/MELTF/COL13A1/LAMA1/APLP1/SLC39A8/MPV17/COL9A2/COL2A1/TEX14/MMP2/LOX/KIF9/SPINT2/ICAM1/ITGB8/SOX9/COL9A3/ITGA2B/PAPLN/FGB/SCUBE1/TNXB/MATN1/FOXF2/HPN/ITGAM/ITGB7/NOXO1/NPHS1/ADAM8/VTN/LCP1/FGFR4/LAMC3/HAPLN2/MATN3
GO:0010466	negative regulation of peptidase activity	38/1035	255/18214	4.8E-08	8.2E-06	7.13E-06	CRYAB/GAPDH/APP/TMED10/APLP2/PRDX3/YWHAE/THBS1/PRDX5/GPX1/PROS1/BEX3/PEBP1/PARK7/SERPINI1/TIMP1/TIMP2/SPOCK2/CST3/ARL6IP1/SNCA/BIN1/TFPI2/SERPINF1/LAMP3/TFPI/SPINT2/SERPINB8/ECM1/SERPIND1/C3/PAPLN/GPC3/ITIH3/BST2/AHSG/VTN/ITIH1
GO:0006457	protein folding	33/1035	223/18214	4.3E-07	5.6E-05	4.92E-05	CRYAB/HSP90AA1/CANX/NPPA/PDIA6/HSP90B1/P4HB/CALR/HSPD1/FKBP8/FKBP1A/B2M/PDIA3/DNAJB11/LMAN1/PDIA4/GRN/PPIA/ST13/CDC37/SELENOF/PFDN5/DNAJC10/VBP1/HSPA1B/HSPB6/HSPA6/TOR1B/HSPE1/FKBP1B/GNAT1/CALR3/RGS9
GO:0010038	response to metal ion	44/1035	348/18214	5.5E-07	6.7E-05	5.87E-05	ACTA1/BSG/APP/CALR/SOD2/CLIC4/B2M/SLC25A23/CALM3/THBS1/GDI1/ADAM9/PARK7/NCSTN/LGMN/BECN1/NQO1/LOXL2/SNCA/MAPK3/IGFBP2/G6PD/SERPINF1/TCIRG1/GIPR/CA2/GGH/MT1G/PKLR/ICAM1/TF/RYR1/AQP3/FGB/MT1H/SYT5/STAR/SLC34A1/CD14/CLDN1/CYP1A2/CPNE9/RASAL1/TFAP2A

GO:0030641	regulation of cellular pH	19/1035	94/18214	1.1E-06	0.00013	0.000114	ATP5F1B/ATP6V0C/CLIC4/CHP1/ATP6V1E1/ATP6AP2/GRN/ATP6AP1/ATP6V1D/ATP6V0B/ATP6V1G1/ATP6V0E2/PPT1/MAPK3/TCIRG1/CA2/SLC9A5/SLC4A1/ATP6V1C2
GO:0051346	negative regulation of hydrolase activity	51/1035	451/18214	2.1E-06	0.00023	0.000202	CRYAB/GAPDH/APP/TMED10/CHP1/APLP2/FKBP1A/PRDX3/YWHAE/THBS1/PRDX5/GPX1/TMED2/PROS1/BEX3/ARPP19/PEBP1/PARK7/SERPINI1/TIMP1/TIMP2/SPOCK2/CST3/ARL6IP1/PPP1R14B/SNCA/BIN1/TFPI2/ARL2/APOC1/SERPINF1/LAMP3/TFPI/SPINT2/LEPR/SERPINB8/ECM1/FKBP1B/SERPIND1/C3/PAPLN/GPC3/ITIH3/GNAT1/BST2/AHSG/PPP1R1B/APOA1/NOS1/VTN/ITIH1
GO:0061615	glycolytic process through fructose-6-phosphate	10/1035	29/18214	2.5E-06	0.00027	0.000231	ENO2/GAPDH/GPI/PGK1/ENO3/PGAM1/ENO1/PKLR/SLC4A1/HK3
GO:0006898	receptor-mediated endocytosis	35/1035	269/18214	4.1E-06	0.00041	0.000359	HSP90AA1/CANX/HYOU1/AP2M1/SCARB2/HSP90B1/CALR/APOE/ITGB1/B2M/CD81/CTTN/M6PR/GRIA1/MTMR2/CTSL/SNCA/PDLIM7/PPT1/APOC1/CCL21/AP1G2/CD9/ARC/SNAP25/NECAB2/TF/DLL1/C3/APOB/CD14/APOA1/ITGAM/VTN/STAB1
GO:0030449	regulation of complement activation	13/1035	55/18214	9.3E-06	0.00084	0.000728	CD81/PROS1/PHB/CD46/CD55/CFB/C3/F2/C2/C8B/VTN/C8G/CFH
GO:0001501	skeletal system development	51/1035	483/18214	1.5E-05	0.00124	0.001076	GNAS/TLE5/CCN2/RHOA/ATP6AP1/RPL38/TIMP1/LOXL2/COL13A1/MAPK3/COL9A2/SRD5A1/COL2A1/MMP2/PAPPA2/IGF2/LOX/HHIP/ECM1/HAPLN3/RFLNB/ITGB8/CHRD/HOXA11/SOX9/RYR1/DSCAML1/FST/EN1/WNT2B/HOXA10/CCN4/HOXA1/TBX1/BMP8A/MATN1/AHSG/MDK/DLX1/PAX5/DMRT2/ALPL/RARG/BMP8B/CHI3L1/RUNX3/CYP26B1/TFAP2A/HAPLN2/MATN3/CLDN18
GO:0002863	positive regulation of inflammatory response to antigenic stimulus	6/1035	12/18214	2.3E-05	0.00177	0.001541	CD81/PARK7/HLA-E/C3/BTK/LTA
GO:0018958	phenol-containing compound metabolic process	18/1035	107/18214	3.1E-05	0.00231	0.002007	PARK7/AKR1B1/SNCA/HPRT1/SRD5A1/PMEL/MOXD1/FAH/GCH1/AOC2/LRTOMT/HPN/STAR/PAX8/DUOX1/SLC5A5/ITGAM/NR4A2
GO:0001503	ossification	43/1035	397/18214	3.8E-05	0.00275	0.00239	GNAS/CCN2/ATP5F1B/IL6ST/PRKACA/RHOA/ATP6AP1/RPL38/PHB/RPS15/COL13A1/PDLIM7/MAPK3/IGFBP3/OSTF1/TCIRG1/COL2A1/MMP2/IGF2/LOX/HSPE1/ECM1/RFLNB/CHRD/IFITM1/SOX9/RYR1/CYP27B1/GPC3/CCN4/BMP8A/MATN1/AHSG/EGR2/MDK/SLC34A1/ALPL/TAC1/BMP8B/DHH/RUNX3/WNT4/TFAP2A
GO:0098869	cellular oxidant detoxification	17/1035	101/18214	5.1E-05	0.00352	0.003067	SELENOW/PRDX2/APOE/SOD2/PRDX6/PRDX3/PRDX5/GPX1/PARK7/SELENOT/NQO1/TXNDC17/MGST1/TXN/GCH1/DUOX1/APOA4
GO:0000028	ribosomal small subunit assembly	7/1035	20/18214	7.6E-05	0.0049	0.00426	RPSA/RPS19/RPS28/RPS5/RPL38/RPS15/RPS10
GO:0007605	sensory perception of sound	21/1035	147/18214	8.7E-05	0.00559	0.004867	GPX1/RPL38/EML2/COL2A1/GSDME/ICAM1/GRAP/LRTOMT/MYO1A/OTOF/HOXA1/OTOG/TBX1/MYO15A/HPN/SLC17A8/MYO7B/MBP/CDH23/TFAP2A/UCN
GO:0006936	muscle contraction	38/1035	351/18214	0.00011	0.00653	0.005678	ACTA1/CRYAB/CALD1/NPPA/CCN2/PRKACA/TPM2/CALM3/HSBP1/CTTN/MYL12B/VEGFB/GAA/CASQ1/BIN1/MYL6B/SGCA/CXCR4/HSPB6/ENO1/PABPN1/TNNT3/P2RX1/FKBP1B/TRIM72/RYR1/ATP2A1/MYH3/LTB4R/TACR1/TMOD4/CACNA1S/NOS1/STRIT1/MYLPF/CHRNE/STAC2/UCN
GO:0071871	response to epinephrine	6/1035	15/18214	0.00011	0.00653	0.005678	PRKACA/SNCA/SRD5A1/RNLS/PKLR/STAR
GO:0021762	substantia nigra development	10/1035	43/18214	0.00012	0.00682	0.005931	RHOA/YWHAQ/CALM3/COX6B1/YWHAE/CDC42/GLUD1/G6PD/MBP/PADI2
GO:0051651	maintenance of location in cell	26/1035	207/18214	0.00012	0.00703	0.006113	FTH1/HSP90B1/CALR/APOE/PRKACA/OS9/FKBP1A/SLC25A23/CALM3/SKP1/TMSB10/PARK7/KDELR2/GAA/SNCA/CASQ1/TXN/ARL2/CCL21/FKBP1B/RYR1/F2/ATP2A1/HAP1/LIME1/NOS1
GO:0007584	response to nutrient	22/1035	164/18214	0.00015	0.00844	0.007344	SOD2/COX4I1/USF2/BECN1/OXCT1/DAD1/NQO1/IGFBP2/GIPR/PKLR/FKBP1B/ABCG8/CYP27B1/AQP3/C2/STAR/SLC34A1/APOA1/ALPL/ALDH3A1/LTA/CYP26B1
GO:0034378	chylomicron assembly	5/1035	11/18214	0.0002	0.01038	0.009032	P4HB/APOE/APOB/APOA1/APOA4
GO:0035722	interleukin-12-mediated signaling pathway	10/1035	47/18214	0.00025	0.01202	0.010456	RPLP0/P4HB/SOD2/CFL1/MIF/CDC42/CAPZA1/PPIA/PLCB1/LCP1
GO:0051402	neuron apoptotic process	27/1035	230/18214	0.00028	0.01275	0.011092	GAPDH/APP/APOE/SOD2/CDC42/GRN/PARK7/NCSTN/LGMN/TNFRSF21/PIN1/NQO1/SIGMAR1/PPT1/G6PD/EN1/AGAP2/NUPR1/STAR/FAIM2/MDK/DLX1/ITGAM/NR4A2/ADAM8/GRM4/TFAP2A

GO:0034341	response to interferon-gamma	24/1035	197/18214	0.00035	0.01521	0.013234	GAPDH/B2M/HLAA/CDC42/CDC37/SNCA/HLAE/HLAC/IFITM3/CCL21/HLAB/IFITM2/GCH1/ICAM1/IFITM1/IRF5/CYP27B1/BST2/STAR/MEFV/IFI30/HLA-DPA1/IRF4/CLDN1
GO:0045454	cell redox homeostasis	9/1035	43/18214	0.00058	0.02295	0.019968	PRDX2/PRDX6/PRDX3/PRDX5/GPX1/SELENOT/NQO1/TXN/NOS1
GO:0048265	response to pain	7/1035	27/18214	0.00061	0.02314	0.020137	THBS1/THBS4/GCH1/TACR1/RET/TAC1/UCN
GO:1903036	positive regulation of response to wounding	12/1035	72/18214	0.00069	0.02571	0.022369	PRDX2/ITGB1/THBS1/GRN/CXCR4/FKBP1B/F2/CCN4/MDK/DUOX1/CLDN1/VTN
GO:0006986	response to unfolded protein	22/1035	183/18214	0.00072	0.02674	0.023269	HSP90AA1/CANX/HYOU1/PDIA6/HSP90B1/CALR/HSPD1/DNAJB11/THBS1/ACADVL/TMED2/SHC1/GET3/CDK5RAP3/HSPA1B/HSPA6/TOR1B/THBS4/TSPYL2/DERL3/HSPE1/CALR3
GO:0034976	response to endoplasmic reticulum stress	31/1035	295/18214	0.00073	0.02676	0.023289	CANX/HYOU1/PDIA6/HSP90B1/P4HB/CALR/OS9/PDIA3/DNAJB11/THBS1/ACADVL/PDIA4/TMED2/BCAP31/PARK7/SHC1/DNAJC10/GET3/SELENOK/CDK5RAP3/RNF5/TXNDC12/TMX1/THBS4/TSPYL2/DERL3/FBXO2/ATP2A1/NUPR1/CALR3/RASGRF1
GO:0019216	regulation of lipid metabolic process	39/1035	401/18214	0.00076	0.02739	0.023829	PSAP/APOE/CHP1/CD81/ACADVL/CDC42/ME1/FADS1/ADIPOR1/TNFRSF21/NPC2/MTMR2/ACADM/LAMTOR1/MBOAT7/CREBL2/APOC1/CCL21/GAL/PSAPL1/SOX9/CYP27B1/C3/F2/AGAP2/APOB/AVIL/STAR/FABP5/DKKL1/APOA1/ADGRF5/FLT3/FGFR4/DHH/WNT4/MLXIPL/GFI1/APOA4
GO:0010872	regulation of cholesterol esterification	5/1035	14/18214	0.00076	0.02739	0.023829	APOE/LAMTOR1/APOC1/APOA1/APOA4
GO:0019882	antigen processing and presentation	26/1035	233/18214	0.00079	0.02794	0.024311	CANX/AP2M1/CALR/SEC23A/B2M/PDIA3/HLAA/THBS1/BCAP31/CAPZA1/LGMN/AP1S2/CTSL/HLAE/PSME1/HLA/CCL21/HLAB/CTSH/ICAM1/VAMP8/CD68/IFI30/HLA-DPA1/WDFY4/HLA-DMA
GO:0000302	response to reactive oxygen species	25/1035	221/18214	0.00081	0.02846	0.024768	CRYAB/PRDX2/APOE/SOD2/PRDX3/PRDX5/GPX1/ADAM9/PDGFD/PARK7/BECN1/STK26/NQO1/SIGMAR1/MAPK3/UCP2/MPV17/TXN/NUDT15/MMP2/GCH1/FKBP1B/STAR/BTK/APOA4
GO:0042110	T cell activation	44/1035	473/18214	0.00091	0.03103	0.027003	PRDX2/IL6ST/HSPD1/FKBP1A/B2M/HLAA/GPNMB/CD81/DDOST/CDC42/CD151/CLPTM1/FLOT2/CXADR/NCSTN/TNFRSF21/CD46/CTSL/SELENOK/HLAE/IGFBP2/CD55/TCIRG1/CCL21/IGF2/PNP/LEPR/ICAM1/FKBP1B/MICB/LAPTM5/MDK/HLADPA1/JAK3/VAV1/IRF4/PIK3R6/WDFY4/CD5/ADAM8/LCP1/RUNX3/CYP26B1/WNT4
GO:0120009	intermembrane lipid transfer	9/1035	46/18214	0.00098	0.0329	0.028626	APOE/PITPNM1/NPC2/PLTP/ABCG8/APOB/STAR/APOA1/APOA4
GO:0002685	regulation of leukocyte migration	23/1035	204/18214	0.00134	0.04027	0.035037	APP/CALR/RHOA/CD81/THBS1/MIF/PDGFD/LGMN/PLCB1/VEGFB/SELENOK/MAPK3/CCL21/THBS4/KITLG/ECM1/ICAM1/ITGA2B/GPSM3/BST1/MDK/ADAM8/PADI2
GO:0097066	response to thyroid hormone	6/1035	23/18214	0.00145	0.04192	0.036475	CTSB/AKR1B1/CTSL/CTSH/HPN/SLC34A1
GO:2001233	regulation of apoptotic signaling pathway	34/1035	348/18214	0.00149	0.04225	0.036763	GHITM/HYOU1/TPT1/PRDX2/P4HB/SOD2/PEA15/YBX3/PDIA3/THBS1/MIF/GPX1/CTTN/BCAP31/PPIA/PARK7/BECN1/HSPA1B/TXNDC12/ENO1/TAF9B/COL2A1/GSDME/ICAM1/FGB/TMC8/SEPTIN4/NUPR1/FAIM2/RET/NR4A2/INCA1/INHBB/WNT4
GO:0019885	antigen processing and presentation of endogenous peptide antigen via MHC class I	5/1035	16/18214	0.00151	0.04277	0.037211	B2M/HLA-A/HLA-E/HLA-C/HLA-B
GO:1903039	positive regulation of leukocyte cell-cell adhesion	25/1035	232/18214	0.00161	0.04487	0.039041	IL6ST/HSPD1/RHOA/IRAK1/HLA-A/CD81/CDC42/FLOT2/CD46/SELENOK/HLA-E/IGFBP2/CD55/CCL21/IGF2/PNP/ICAM1/MDK/HLA-DPA1/JAK3/VAV1/PIK3R6/CD5/ADAM8/RUNX3
GO:0050867	positive regulation of cell activation	33/1035	338/18214	0.00175	0.04651	0.040468	CCN2/IL6ST/HSPD1/HLA-A/CD81/THBS1/MIF/CDC42/LAMP1/FLOT2/SH3KBP1/CD46/SELENOK/HLA-E/IGFBP2/CD55/CCL21/IGF2/PNP/VAMP8/BST1/MDK/HLA-DPA1/JAK3/VAV1/PIK3R6/CD5/BTK/ITGAM/TAC1/ADAM8/RUNX3/TAFA3
GO:0070665	positive regulation of leukocyte proliferation	18/1035	148/18214	0.00186	0.04798	0.041748	IL6ST/HLA-A/CD81/MIF/CD46/SELENOK/HLA-E/MAPK3/IGFBP2/CD55/KITLG/IGF2/PNP/BST2/BST1/HLA-DPA1/JAK3/TAC1
GO:0070661	leukocyte proliferation	31/1035	313/18214	0.0019	0.04809	0.041845	PRDX2/IL6ST/HSPD1/HLAA/GPNMB/CD81/MIF/CD151/NCSTN/TNFRSF21/CD46/SELENOK/HLAE/MAPK3/IGFBP2/HPRT1/CD55/TCIRG1/GAL/KITLG/IGF2/PNP/FKBP1B/BST2/BST1/HLA-DPA1/JAK3/BTK/TAC1/FLT3/WNT4

GO Terms_DOXOvsDMSO_Downregulated							
ID	Description	GeneRatio	BgRatio	pvalue	p.adjust	qvalue	geneID
GO:0034401	chromatin organization involved in regulation of transcription	66/2210	152/18214	3.5E-22	1.9E-18	1.7E-18	H1-4/H4C5/ZMIZ1/H2AC20/KCNQ1OT1/H1-2/SERTAD2/KMT2D/ARID1A/H4C2/TPR/UBR2/H4-16/SIN3A/H3C2/ZNFX1/H4C3/H4C8/H1-5/BAHD1/MTHFR/H2AC11/CDCA4/ARID1B/H3-3B/H2AC8/RESF1/H3C8/H3C7/H2AC17/ATAD2B/H2AX/RIF1/PPM1D/H1-3/ZNF304/H2AC6/H4C9/H4C4/SERTAD1/SMARCD1/RRP8/H2AC13/H3C6/SIRT1/MSL3P1/H2AC12/H2AC4/H4C1/H3C10/H2AC21/H3C1/EED/SUV39H1/NRDE2/SIRT6/H3C11/PCGF1/H3C12/H3C4/BEND3/H4C11/H2AC16/H3C13/H2AC15/H4C13
GO:0006323	DNA packaging	83/2210	237/18214	3.2E-20	8.9E-17	7.77E-17	H1-4/H4C5/TTN/H1-2/H4C2/BRD2/TPR/H2BC4/WAPL/H4-16/KAT6B/H3C2/ZNFX1/UBN1/H4C3/H4C8/H1-5/BAHD1/CTCF/H2BC9/DAXX/H2BC8/H3-3B/TOP2A/TSPYL5/KAT6A/RESF1/H3C8/H3C7/CCNB1/PHF13/H2AX/TSPYL4/TENT4A/H1-3/ZNF304/H4C9/H4C4/H2BC15/H2BC5/H2BC18/RRP8/GRWD1/H2BC11/MCPH1/H3C6/H2BC17/INCENP/H2BC6/CENPC/SIRT1/HJURP/H2BC7/TSPY26P/LIN54/GPER1/H4C1/H3C10/H3C1/CDAN1/KNL1/H2BC10/SUV39H1/NRDE2/SIRT6/H2BC13/H3C11/CENPO/NCAPG/H3C12/MIS18BP1/CHAF1A/H3C4/NCAPH/BEND3/CDCA5/CENPA/DFFB/H2BU1/H4C11/H3C13/H4C13/TSSK6
GO:0040029	regulation of gene expression, epigenetic	74/2210	201/18214	1.3E-19	2.5E-16	2.15E-16	H1-4/EP300/H4C5/ZMIZ1/H2AC20/KCNQ1OT1/H1-2/SERTAD2/KMT2D/BAZ1B/ARID1A/H4C2/TRIM27/UBR2/H4-16/SIN3A/RBM15B/H3C2/H4C3/H4C8/H1-5/MTHFR/CTCF/H2AC11/CDCA4/ARID1B/RLIM/RBM15/H3-3B/H2AC8/H3C8/H3C7/H2AC17/ATAD2B/POLR1B/H2AX/RIF1/PPM1D/H1-3/JARID2/H2AC6/H4C9/H4C4/EPC1/SERTAD1/SMARCD1/LRIF1/H2AC13/H3C6/SIRT1/MSL3P1/MTF2/AEBP2/H2AC12/H2AC4/H4C1/ERCC6/H3C10/H2AC21/H3C1/KLF2/EED/SIRT6/TBP/H3C11/PCGF1/H3C12/BRCA1/H3C4/H4C11/H2AC16/H3C13/H2AC15/H4C13
GO:0000070	mitotic sister chromatid segregation	62/2210	164/18214	2.7E-17	1.9E-14	1.63E-14	NUMA1/TTN/TPR/CENPF/APC/POGZ/NIPBL/MAD2L1BP/CHAMP1/CHMP6/CCNB1/PHF13/TENT4A/RIOK2/PDS5B/NDC80/CENPE/DUSP1/KIF22/CHMP7/RACGAP1/RRS1/INO80/BUB1/NUP62/INCENP/CENPC/KIFC1/CDC6/BUB1B/KNSTRN/KIF23/CDCA8/KIF18B/DLGAP5/MAP10/KIF2C/PSMG2/CDC20/ZW10/MIS12/TTK/MAD1L1/CDT1/SPAG5/PLK1/NCAPG/UBE2C/HASPIN/KIF14/NCAPH/FBXO5/KIF4A/AURKB/WRAP73/PSRC1/CDCA5/KIF18A/NEK2/ESPL1/NUF2/SGO1
GO:0016570	histone modification	113/2210	448/18214	1E-14	3.7E-12	3.22E-12	MYOCD/EP300/BCOR/KDM6A/PPARGC1A/KMT2E/WAC/WDR82/ASXL1/CREBBP/KMT2D/BAZ1B/SRCAP/UBR5/KDM5B/NSD1/KDM4B/TADA2B/LDB1/UBR2/KMT2A/JMJD1C/KMT2C/KAT6B/PYGO2/SIN3A/USP7/GTF3C4/BCL6/H1-5/SKI/MIDEAS/MSL2/KDM3A/MTHFR/CTCF/IWS1/SMAD4/KAT7/RLF/NIPBL/TET3/KDM5A/TAF7/KAT5/RCOR2/KDM4A/HR/AUTS2/KAT6A/KAT14/JMJD6/YEATS2/CCNB1/LEO1/RIF1/MTA2/KANSL1/BRPF1/JARID2/ZNF304/TADA1/RIOX1/USP51/ZNF274/EPC1/RBM14/EPC2/PER1/WDR5/TADA2A/DCAF1/TRIM16/GTF2B/INCENP/THAP7/MIER2/KANSL2/SIRT1/MSL3P1/MTF2/PRDM16/MEN1/WDR70/DTX3L/KMT5C/CBX8/FBXL19/EED/JADE2/SUV39H1/JDP2/PRKD1/SIRT6/MBIP/PCGF1/USP21/N6AMT1/ING2/CCNA2/HASPIN/SETMAR/TAF5/BRCA1/SGF29/AURKB/BEND3/UHRF1/OTUB2/LRRK2/WDR5B/ISL1/KDM8
GO:0045652	regulation of megakaryocyte differentiation	33/2210	83/18214	1.5E-10	3E-08	2.63E-08	EP300/H4C5/KMT2E/KMT2D/H4C2/KMT2A/H4-16/KMT2C/SIN3A/H3C2/H4C3/H4C8/RBM15/H3-3B/H3C8/H3C7/H4C9/H4C4/TNRC6C/WDR5/H3C6/ZNF16/H4C1/H3C10/H3C1/RAB7B/H3C11/ZFPM1/H3C12/H3C4/H4C11/H3C13/H4C13
GO:0050000	chromosome localization	32/2210	80/18214	2.4E-10	4.5E-08	3.99E-08	NUMA1/CENPF/CHAMP1/CHMP6/NDEL1/CCNB1/NDC80/CENPE/KIF22/CHMP7/RRS1/NUP62/INCENP/CENPC/KIFC1/IFFO1/CDCA8/DLGAP5/KIF2C/ZW10/MIS12/MAD1L1/CDT1/SPAG5/NDE1/KIF14/AURKB/PSRC1/CDCA5/KIF18A/NUF2/GEM
GO:0003279	cardiac septum development	35/2210	96/18214	7E-10	1.1E-07	9.77E-08	GATA4/CITED2/LUZP1/SMAD6/GATA6/TBX20/MAML1/FZD2/TBX5/SMAD4/ZFPM2/NKX2-5/NDST1/CHD7/HES1/SMAD7/LMO4/RBM15/TBX2/BMPR1A/WNT5A/SOX11/SUFU/TRIP11/HEY1/TBX3/SOX4/HEY2/ZFPM1/NOG/RARB/GJA5/ISL1/DAND5/HEYL
GO:0000075	cell cycle checkpoint	57/2210	208/18214	1.7E-09	2.3E-07	1.99E-07	EP300/TNKS1BP1/PPP1R10/WAC/BTG2/TAOK1/FEM1B/TPR/CENPF/APC/MAPK14/TAOK2/MAD2L1BP/CNOT2/CNOT6L/FZR1/ARID3A/CCNB1/H2AX/SOX11/CNOT8/CDKN1B/ZNF830/NDC80/MUS81/DUSP1/BUB1/FBXO31/DTL/SOX4/GADD45A/CDC6/BUB1B/E2F8/TRIAP1/PSMG2/ERCC6/CNOT10/CDC20/ZW10/RFWD3/PLK2/TTK/ETAA1/TICRR/GTSE1/MAD1L1/DCLRE1B/CDT1/PLK1/HINFP/SETMAR/BRCA1/AURKB/BLM/RAD9A/EME1

GO:0045787	positive regulation of cell cycle	83/2210	393/18214	2.5E-07	1.9E-05	1.71E-05	CITED2/EP300/TNKS1BP1/PPP1R10/RARA/NUMA1/ABL1/KMT2E/GATA6/BTG2/TPR/MEIS2/ADAMTS1/DAB2IP/ANKRD17/TRIM32/SIN3A/USP2/CCNT1/DYRK3/NIPBL/HES1/MAD2L1BP/CNOT2/ID2/CNOT6L/ARID3A/RPTOR/CCNB1/WNT5A/CEP295/CNOT8/CDKN1B/CDC25A/CCNT2/NDC80/SRC/RRP8/RACGAP1/TBX3/INO80/DTL/NUP62/SOX4/GADD45A/KCNA5/CDC6/E2F8/ZBED9/KIF23/ZNF16/MEPCE/TRIAP1/DLGAP5/MAP10/GPER1/CNOT10/PLK2/KIF20B/CIT/MIR208A/PDGFB/GTSE1/CDT1/SPAG5/ZBTB17/BCL2L11/UBE2C/SETMAR/BRCA1/DBF4/KIF14/NKX3-1/FBXO5/TRIM21/AURKB/PSRC1/CDCA5/PLK4/ESPL1/TFAP4/MSX1/FOSL1
GO:0009896	positive regulation of catabolic process	91/2210	448/18214	4.2E-07	2.8E-05	2.47E-05	TIPARP/TRIB1/PRICKLE1/ZFP36L1/GIGYF2/WAC/TRIB2/SH3BP4/BTG2/TRIM27/TOB1/PFKFB3/DAB2IP/TRIM32/NFE2L2/FOXO1/APC/FOXO3/GSK3B/MYLIP/KEAP1/MAGEF1/YTHDF2/TRIM13/PAFAH1B2/AREL1/TFEB/YTHDF1/HERPUD1/SNX33/SMAD7/ZFP36L2/KAT5/COP1/VGLL4/AMBRA1/TBK1/EIF4ENIF1/TRIM65/RNF19A/ZBTB20/CNOT6L/PIAS1/FZR1/DAPK1/NKD1/RNF139/TP53INP1/ADORA1/SESN1/SOCS4/IRS1/WNT5A/GPSM1/SESN2/SMURF1/RNF41/AMER1/PFKFB4/RNF19B/SPTLC2/CNOT8/CDKN1B/STK11/ARNT/AXIN1/MEX3D/TNRC6C/RNF152/DTL/CBLB/SIRT1/TRIM68/TICAM1/STX5/DTX3L/CDC20/PRXL2C/PLK2/PRKD1/SIRT6/ESRRB/DACT1/PLK1/ADRB2/BCL2L11/RNF144B/TRIB3/TRIM21/PLEKHF1/LRRK2
GO:0071900	regulation of protein serine/threonine kinase activity	95/2210	489/18214	1.9E-06	0.00011	9.47E-05	UBC/INKA2/TRIB1/MYOCD/AKAP13/RAPGEF2/SPRY2/ABL1/FZD4/DUSP7/TRIB2/SPRY4/CRKL/TAOK1/CCNJL/RGS3/MUL1/DAB2IP/MADD/APC/MAPK14/UBB/TAOK2/DUSP5/CCNT1/AJUBA/DAXX/MAP2K7/DUSP16/DUSP14/TRAF6/CDK12/DUSP6/KSR1/BMP2/RPTOR/CCNB1/SPRY1/WNT5A/SESN2/CDKN1B/CDC25A/ROR2/CCNT2/SERTAD1/MAP4K3/DUSP1/SRC/MAP3K9/PARP16/SPRED2/DVL2/NUP62/SASH1/CCNE1/INCENP/PDCD10/CDK7/SIRT1/GADD45A/CDC6/CCNF/FGF18/MEN1/SPRED1/TRAF2/DKK1/DUSP10/ERCC6/CIT/MAP2K5/CCNB2/PDGFB/CCNQ/ETAA1/AVPI1/DUSP18/PLK1/ADRB2/RGS14/CCNA2/DUSP8/DBF4/IQGAP3/TRIB3/PSRC1/BLM/CCNG2/TFAP4/RASGRP1/FGF2/LRRK2/CDKN3/S1PR2/PKMYT1
GO:0006303	double-strand break repair via nonhomologous end joining	29/2210	98/18214	3E-06	0.00017	0.000146	H4C5/H4C2/H4-16/H4C3/H4C8/KAT5/ZBTB7A/TFIP11/H2AX/RIF1/CYREN/H4C9/H4C4/USP51/ERCC4/PIAS4/IFFO1/SMARCAL1/H4C1/DTX3L/DCLRE1A/ERCC6/KMT5C/DCLRE1B/SETMAR/BRCA1/H4C11/H4C13/AUNIP
GO:0038111	interleukin-7-mediated signaling pathway	14/2210	30/18214	3.1E-06	0.00017	0.000149	H3C2/BRWD1/H3C8/H3C7/IRS1/H3C6/H3C10/H3C1/H3C11/CISH/H3C12/H3C4/H3C13/TSLP
GO:0051098	regulation of binding	73/2210	356/18214	4E-06	0.00021	0.000185	HAND2/BAMBI/MYOCD/EP300/RARA/RAPGEF2/ABL1/XIRP1/NSD1/MARK3/WAPL/PYGO2/SIN3A/ARHGEF7/SKI/GSK3B/SMAD4/LARP6/SMAD3/NFKBIA/JUN/HES1/MARK2/RIPOR2/TRAF6/ZBTB7A/TFIP11/BMP2/MSX2/CRK/WNT5A/SOX11/GPSM1/ZNF304/PEX14/CEBPG/BCL3/IFIT1/HEY1/ERCC4/SRC/LDLRAP1/GTF2B/CBLB/GMNN/HJURP/TICAM1/HEY2/MEPCE/MEN1/DKK1/DTX3L/PLK2/PDGFB/CDT1/DACT1/PLK1/ZFPM1/ZNF593/ADRB2/NKX3-1/TRIB3/TRIM21/NOG/AURKB/NEK2/MDFI/TFAP4/LRRK2/MSX1/ZBTB7C/ISL1/MIR27B
GO:0000281	mitotic cytokinesis	23/2210	70/18214	4.5E-06	0.00023	0.000203	APC/KLHDC8B/SNX33/LZTS2/RAB35/CHMP6/KIF20A/RTKN/RHOB/CHMP7/RACGAP1/RASA1/SNX18/NUP62/INCENP/KIF23/KIF20B/CIT/PLK1/CEP55/KIF4A/ESPL1/CENPA
GO:0071459	protein localization to chromosome, centromeric region	12/2210	25/18214	1.1E-05	0.00052	0.000461	CTCF/CHAMP1/NDC80/BUB1B/ZW10/MIS12/KNL1/TTK/CDT1/HASPIN/AURKB/CENPA
GO:0045598	regulation of fat cell differentiation	34/2210	136/18214	2.6E-05	0.00103	0.000905	ZFP36L1/XBP1/TRIB2/ASXL1/ASXL2/RREB1/FOXO1/MAPK14/ZFPM2/SMAD3/HES1/PIM1/ZFP36L2/CREB1/RUNX1T1/METRNL/BMP2/WNT5A/INSIG1/BBS12/RORA/SIRT1/GPER1/DUSP10/NAPEPLD/CCDC85B/JDP2/ZFPM1/CEBPB/TRIB3/NOCT/ZBTB7C/MIR27B/CMKLR1
GO:0043923	positive regulation by host of viral transcription	9/2210	16/18214	2.9E-05	0.0011	0.000968	EP300/CCNT1/RRP1B/JUN/CHD1/SP1/CTDP1/ZNF639/TFAP4
GO:0071560	cellular response to transforming growth factor beta stimulus	53/2210	250/18214	3.2E-05	0.00117	0.001032	UBC/BAMBI/CITED2/MYOCD/BCL9L/EP300/ZFP36L1/ZMIZ1/SPRY2/PPARGC1A/ZNF703/SMAD6/CREBBP/RASL11B/CRKL/GCNT2/SKI/FYN/UBB/SMAD4/SMAD3/JUN/SMAD7/ZFP36L2/RNF111/ZBTB7A/CREB1/BMPR1A/LDLRAD4/SPRY1/CRK/MAPK7/WNT5A/SOX11/LEMD3/SMURF1/EID2/NR3C1/STK11/SMAD5/SMAD1/SRC/TGFBRAP1/SPRED2/ARHGEF18/SIRT1/ANKRD1/PRDM16/MEN1/SPRED1/ING2/SOX5/DAND5
GO:0006275	regulation of DNA replication	28/2210	107/18214	5.5E-05	0.00185	0.001624	ID3/WAPL/ANKRD17/BCL6/KAT7/SMC3/JUN/GDF2/ZBTB38/RBBP6/TTF1/ZNF830/INO80/OBI1/GMNN/GLI2/CDC6/SMARCAL1/E2F8/CDAN1/TICRR/CDT1/CCNA2/DBF4/ORC5/FBXO5/BLM/BCAR3

GO:0000082	G1/S transition of mitotic cell cycle	56/2210	274/18214	5.6E-05	0.00188	0.001652	EP300/TNKS1BP1/KMT2E/CTDSP2/BTG2/ACVR1B/ADAMTS1/ANKRD17/APC/CUL1/CNOT2/ID2/CNOT6L/PIAS1/ARID3A/RPTOR/CCNB1/E2F3/CNOT8/CDKN1B/CDC25A/INO80/FBXO31/CCNE1/DCUN1D3/GMNN/SOX4/CDK7/GADD45A/KCNA5/CDC6/E2F6/E2F8/MEPCE/TRIAP1/NPAT/CNOT10/RFWD3/PLK2/ORC2/POLA2/MIR208A/CTDSPL/ESRRB/GTSE1/CDT1/DACT1/HINFP/DBF4/KIF14/ORC5/IQGAP3/FBXO5/PRIM1/FAM107A/CDKN3
GO:0007623	circadian rhythm	46/2210	212/18214	5.7E-05	0.00188	0.001652	EP300/PPARGC1A/ID3/KLF9/BHLHE40/KMT2A/NRIP1/SIN3A/USP7/JUND/DYRK1A/GSK3B/USP2/SETX/JUN/RAI1/KDM5A/TOP2A/CREB1/ID2/NFIL3/ADORA1/SRRD/NR2F6/DBP/CIPC/PER1/ADRB1/RORA/SIAH2/FBXL3/SIRT1/SPSB4/FBXL22/PHLPP1/GPR157/KLF10/PER3/SUV39H1/FBXL12/MAGEL2/BHLHE41/RBM4B/NOCT/EGR1/KDM8
GO:0001701	in utero embryonic development	65/2210	334/18214	7.2E-05	0.00221	0.001943	HAND2/BCOR/ZFP36L1/ZMIZ1/ZFAND5/CHD8/GATA6/ACVR1B/TANC2/GGNBP2/SIN3A/KEAP1/SMAD4/ZFPM2/SMAD3/PELO/CHD7/GLI3/HES1/TFEB/CNOT2/TRAF6/BMPR1A/ADM/BMP2/SP3/NDEL1/CCNB1/OTUD7B/RUNDC1/SEC24D/RBBP6/SCO2/ARNT/ZNF830/HEY1/TBX3/TERF2/XAB2/KRT19/GLI2/E2F8/HEY2/NSRP1/NPAT/JUNB/KLF2/DNAAF2/CCNB2/PDGFB/TTLL4/XRCC2/HINFP/CEBPB/BCL2L11/ELL/PALB2/NOG/ARNT2/PLK4/NEK2/MDFI/AKAP3/MSX1/FOSL1
GO:0006109	regulation of carbohydrate metabolic process	44/2210	206/18214	0.00012	0.00334	0.002933	PPP1R3C/PPP1R3B/EP300/DYRK2/FOXK1/PPARGC1A/MIDN/SOGA1/PFKFB3/TPR/USP7/NUP153/DDIT4/GSK3B/NUP98/EPM2AIP1/FOXK2/ZBTB7A/ZBTB20/POM121C/POM121/NUP214/IRS1/SESN2/NFKB1/PPP1R3A/PFKFB4/PPP1R3D/MAEA/ARNT/RAE1/SRC/RORA/WDR5/NUP62/HAS2/SIRT1/GCK/GPER1/NUP85/PRXL2C/PDGFB/NUP37/ESRRB
GO:0032259	methylation	66/2210	355/18214	0.00025	0.00635	0.005578	MAT2A/BCOR/KDM6A/KMT2E/WDR82/CREBBP/KMT2D/BTG2/NSD1/VIRMA/KMT2A/KMT2C/PYGO2/RBM15B/H1-5/KDM3A/MTHFR/WTAP/CTCF/IWS1/SMAD4/RLF/TET3/RBM15/AUTS2/CMTR2/RIF1/LCMT2/MTA2/PPM1D/JARID2/ZNF304/ZNF274/BCDIN3D/RRP8/TRMT12/WDR5/TRMT5/SIRT1/PCIF1/MTF2/PRDM16/MEPCE/MEN1/TGS1/PRMT9/THADA/MRM2/CBLL1/NSUN5/KMT5C/EED/SUV39H1/MRM3/METTL7A/DPH5/TFB1M/THUMPD2/N6AMT1/SETMAR/BRCA1/DIMT1/METTL18/BEND3/ETFBKMT/WDR5B
GO:0060562	epithelial tube morphogenesis	59/2210	309/18214	0.00026	0.00641	0.005629	HAND2/GATA4/PRICKLE1/LUZP1/SPRY2/ABL1/DLC1/BRD2/TBX20/KDM5B/FZD2/SEC24B/SKI/MTHFR/FOXP1/SMAD4/SMAD3/NKX2-5/GLI3/HES1/GDF2/LMO4/RBM15/STARD13/LZTS2/TRAF6/TBX2/ADM/BMP2/FAT4/SPRY1/BCL10/WNT5A/SOX11/KIF26B/SEMA4C/SUFU/SHROOM3/RHOB/MTSS1/EPHA2/SRC/IRX3/TBX3/DVL2/RALA/KLHL3/SOX4/GLI2/IRX1/FOXD1/GZF1/KIF20B/SIRT6/NKX3-1/IRX2/NOG/FKBPL/FGF2
GO:0000271	polysaccharide biosynthetic process	21/2210	78/18214	0.0003	0.00701	0.00616	PPP1R3C/PPP1R3B/DYRK2/GSK3B/NDST1/EPM2AIP1/IRS1/NFKB1/PPP1R3A/PPP1R3D/B3GNT2/B3GNT9/HAS2/GCK/PDGFB/B3GNT8/NDST2/ESRRB/B3GALT2/NHLRC1/B3GALT1
GO:2001020	regulation of response to DNA damage stimulus	45/2210	221/18214	0.00031	0.00732	0.006425	PPP1R10/MCL1/ABL1/UBR5/FEM1B/TRIM32/DYRK1A/MAGEF1/KAT7/DYRK3/RNF169/TFIP11/H2AX/PPP4R2/RIF1/IER3/CYREN/USP51/CEBPG/ERCC4/TERF2/SPRED2/AXIN2/PIAS4/SIRT1/ANKRD1/POLH/TRIAP1/SPRED1/DTX3L/ERCC6/KMT5C/CBX8/RFWD3/ETAA1/SIRT6/ING2/SETMAR/BRCA1/NKX3-1/FIGNL1/FBXO5/OTUB2/MSX1/AUNIP
GO:0034616	response to laminar fluid shear stress	7/2210	13/18214	0.00034	0.0076	0.006675	SMAD6/NFE2L2/SMAD7/MAPK7/KLF2/MAP2K5/ETS1
GO:0043467	regulation of generation of precursor metabolites and energy	35/2210	160/18214	0.00035	0.00772	0.006784	PPP1R3C/PPP1R3B/EP300/DYRK2/PPARGC1A/PFKFB3/TPR/NUP153/TACO1/DDIT4/GSK3B/NUP98/EPM2AIP1/ZBTB7A/ZBTB20/POM121C/POM121/NUP214/CCNB1/IRS1/PPP1R3A/PFKFB4/PPP1R3D/ARNT/RAE1/NUP62/PDE12/PRDM16/GCK/NUP85/PRXL2C/NUP37/NOA1/ESRRB/ETFBKMT
GO:0030010	establishment of cell polarity	31/2210	136/18214	0.00035	0.00772	0.006784	NUMA1/SPRY2/ARF6/CRKL/AMOTL2/GOLPH3/KANK1/AMOTL1/FRMD4B/GSK3B/ARFGEF1/MYO9A/HES1/MARK2/RIPOR2/NDEL1/SPRY1/CRK/WNT5A/KIF26B/RNF41/STK11/NDC80/MCPH1/CYTH3/ZW10/KIF20B/PLK1/NDE1/CENPA/SAPCD2
GO:0006110	regulation of glycolytic process	21/2210	79/18214	0.00036	0.00772	0.006784	EP300/PPARGC1A/PFKFB3/TPR/NUP153/DDIT4/NUP98/ZBTB7A/ZBTB20/POM121C/POM121/NUP214/PFKFB4/ARNT/RAE1/NUP62/GCK/NUP85/PRXL2C/NUP37/ESRRB
GO:0051438	regulation of ubiquitin-protein transferase activity	16/2210	53/18214	0.00038	0.00794	0.006977	ARRDC3/ABL1/FEM1B/BAG5/SMAD7/TOPORS/FZR1/AXIN1/DCUN1D3/ARRDC4/DTX3L/CDC20/PLK1/UBE2C/FBXO5/TRIB3
GO:0060021	roof of mouth development	22/2210	86/18214	0.00047	0.00957	0.008402	HAND2/TIPARP/BCOR/EPHB3/FZD2/PYGO2/SKI/SMAD4/CHD7/GLI3/TBX2/BMPR1A/WNT5A/SOX11/PKDCC/INSIG1/TSHZ1/CSRNP1/TBX3/ARID5B/PAK1IP1/MSX1

GO:2000060	positive regulation of ubiquitin-dependent protein catabolic process	25/2210	103/18214	0.00047	0.0096	0.008436	TRIB1/PRICKLE1/TRIB2/NFE2L2/GSK3B/KEAP1/MAGEF1/HERPUD1/SMAD7/COP1/RNF19A/PIAS1/FZR1/RNF139/SOCS4/SMURF1/RNF19B/AXIN1/CDC20/PLK2/SIRT6/PLK1/RNF144B/TRIB3/LRRK2
GO:0042254	ribosome biogenesis	57/2210	303/18214	0.00048	0.0096	0.008436	RMRP/ZNF622/IMP3/CHD7/RRP1B/MPHOSPH10/THUMPD1/GNL2/GEMIN4/RPP25/ABT1/URB2/RPUSD2/WDR43/SURF6/RIOK2/RPP38/DDX28/NOL8/RRP8/NOL11/RRS1/NAT10/EXOSC9/NOP16/NOB1/MALSU1/FRG1/DDX10/MRM2/RPUSD3/NSUN5/ZNF658/LYAR/WDR3/RIOK1/RPUSD1/DDX31/NGDN/EXOSC4/UTP23/MPV17L2/SUV39H1/MRM3/POP7/TFB1M/LTV1/BRIX1/MTERF3/MRPL36/DIMT1/NOC4L/RCL1/EXOSC3/PAK1IP1/UTP18/GAR1
GO:0044766	multi-organism transport	18/2210	66/18214	0.00066	0.0126	0.011063	UBC/VPS37B/TPR/NUP153/UBB/NUP98/PIKFYVE/POM121C/POM121/NUP214/IFIT1/RAE1/VPS37C/NUP62/MVB12B/NUP85/NUP37/VPS37D
GO:1902579	multi-organism localization	18/2210	66/18214	0.00066	0.0126	0.011063	UBC/VPS37B/TPR/NUP153/UBB/NUP98/PIKFYVE/POM121C/POM121/NUP214/IFIT1/RAE1/VPS37C/NUP62/MVB12B/NUP85/NUP37/VPS37D
GO:0000288	nuclear-transcribed mRNA catabolic process, deadenylation-dependent decay	20/2210	77/18214	0.00067	0.0128	0.011245	TNKS1BP1/ZFP36L1/BTG2/TOB1/ZFP36L2/CNOT2/EIF4ENIF1/CNOT6L/TENT4A/CNOT8/TNRC6C/EDC3/EXOSC9/PDE12/CNOT10/EXOSC4/LSM2/NOCT/EXOSC3/DCP1B
GO:0010212	response to ionizing radiation	31/2210	142/18214	0.00077	0.01425	0.012515	TNKS1BP1/VCAM1/MAPK14/TRIM13/NIPBL/AEN/KAT5/COP1/TSPYL5/BAK1/H2AX/STK11/TLK2/RHOB/INO80/NET1/DCUN1D3/SIRT1/GADD45A/MEN1/ERCC6/RFWD3/TICRR/INIP/XRCC2/BRCA1/FIGNL1/BLM/CYP2R1/EGR1/RAD9A
GO:0001570	vasculogenesis	20/2210	78/18214	0.00081	0.01478	0.012983	GJC1/TIPARP/MYOCD/ZFP36L1/ZMIZ1/RAPGEF2/FZD4/TBX20/TBX5/ZFPM2/NKX2-5/GDF2/ADM/HEY1/EPHA2/RASA1/HAS2/HEY2/SPRED1/JUNB
GO:0070988	demethylation	19/2210	73/18214	0.00089	0.01609	0.014131	KDM6A/KDM5B/KDM4B/APOBEC2/JMJD1C/USP7/KDM3A/TET3/KDM5A/KDM4A/HR/JMJD6/ALKBH4/JARID2/RIOX1/MMACHC/FBXL19/ALKBH2/KDM8
GO:0042149	cellular response to glucose starvation	14/2210	47/18214	0.001	0.01768	0.015525	XBP1/NFE2L2/MTMR3/FOXO3/SLC2A1/PIK3R4/SIK2/SESN1/SESN2/EIF2AK3/RRP8/NUAK1/TBL2/NUAK2
GO:2001224	positive regulation of neuron migration	7/2210	15/18214	0.00102	0.01782	0.015654	RAPGEF2/ARHGEF2/DAB2IP/ZNF609/NIPBL/FBXO31/KIF20B
GO:0098732	macromolecule deacylation	25/2210	109/18214	0.00115	0.01992	0.017492	EP300/SIN3A/BCL6/SKI/MIDEAS/DYRK1A/TPPP/NIPBL/KDM5A/RCOR2/MTA2/RBM14/PER1/SPRED2/ABHD17B/MIER2/SIRT1/MSL3P1/SPRED1/JDP2/PRKD1/SIRT6/ING2/SIRT4/LRRK2
GO:0051053	negative regulation of DNA metabolic process	27/2210	122/18214	0.00132	0.02227	0.019562	H1-4/MSH6/H1-2/UBR5/KMT2A/BCL6/H1-5/MAGEF1/GDF2/RNF169/TFIP11/RIF1/H1-3/CYREN/ERCC4/DUSP1/SRC/TERF2/NAT10/ANKRD1/MEN1/ERCC6/SLX4/ZNF93/BLM/OTUB2/AUNIP
GO:0051145	smooth muscle cell differentiation	18/2210	70/18214	0.00139	0.0233	0.020462	MYOCD/GATA6/ANKRD17/MRTFA/HES1/TBX2/PIAS1/NFATC2/ADM/EPC1/HEY1/TBX3/SIRT1/HEY2/GPER1/PDGFB/MIR145/MIR125B1
GO:0061036	positive regulation of cartilage development	10/2210	29/18214	0.00153	0.02471	0.021706	SMAD3/GLI3/GDF2/SMAD7/BMP2/WNT5A/PKDCC/SMAD1/FGF18/SOX5
GO:0061311	cell surface receptor signaling pathway involved in heart development	10/2210	29/18214	0.00153	0.02471	0.021706	HAND2/BMPR1A/BMP2/WNT5A/HEY1/HEY2/DKK1/NOG/MSX1/HEYL
GO:1900101	regulation of endoplasmic reticulum unfolded protein response	10/2210	29/18214	0.00153	0.02471	0.021706	XBP1/PIK3R1/DAB2IP/PPP1R15B/BAK1/NCK1/PIGBOS1/BCL2L11/BOK/FICD
GO:0002088	lens development in camera-type eye	19/2210	77/18214	0.00176	0.02746	0.024121	SPRY2/KDM5B/PYGO2/SKI/SMAD3/MEIS1/FZR1/WNT5B/SPRY1/WNT5A/SOX11/EPHA2/SPRED2/SIX5/CTNS/SPRED1/NECTIN1/FGF2/BCAR3

GO:0001655	urogenital system development	57/2210	320/18214	0.00182	0.0284	0.024945	TIPARP/MYOCD/ID3/SMAD6/ASXL1/CRKL/FEM1B/ADAMTS1/CENPF/EPHB3/PYGO2/SMAD4/KCNJ8/SMAD3/NIPBL/GLI3/HES1/SMAD7/LZTS2/FADD/JMJD6/BMP2/FAT4/SPRY1/WNT5A/SOX11/KIF26B/AMER1/STK11/SMAD5/MTSS1/SMAD1/IRX3/KLF15/GLIS2/PLAG1/KLHL3/HAS2/SOX4/ARID5B/GLI2/IRX1/FOXD1/GZF1/NUP85/PDGFB/BCL2L11/ZNF354A/NKX3-1/IRX2/NOG/RARB/FGF2/EGR1/LRRK2/HEYL/GCNT1
GO:0051170	import into nucleus	33/2210	162/18214	0.00184	0.02858	0.025099	TXNIP/PRICKLE1/PPP1R10/PIK3R1/MAVS/UBR5/TPR/MAPK14/NUP153/SMAD3/NUP98/RANBP6/NFKBIA/GLI3/EIF4ENIF1/FAM53B/POM121C/POM121/DDX20/NUP214/GEMIN4/ABRA/RBM22/E2F3/SUFU/GEMIN7/NUP62/CBLB/GEMIN6/NXT1/NUP85/GEMIN2/LRRK2
GO:0014855	striated muscle cell proliferation	18/2210	72/18214	0.00196	0.02989	0.026256	TENM4/GATA6/TBX20/MAPK14/TBX5/ZFPM2/PIM1/VGLL4/MEIS1/TBX2/BMPR1A/CCNB1/JARID2/SMAD1/SIX5/HEY2/NOG/FGF2
GO:0048538	thymus development	13/2210	45/18214	0.00204	0.03055	0.026834	HAND2/ABL1/ASXL1/CRKL/EPHB3/HES1/LMO4/FADD/JARID2/ZBTB1/CCNB2/MAD1L1/BCL2L11
GO:0010667	negative regulation of cardiac muscle cell apoptotic process	10/2210	30/18214	0.00204	0.03055	0.026834	HAND2/GATA4/MYOCD/PPP1R10/NFE2L2/NKX2-5/AMBRA1/HEY2/SIRT4/MIR145
GO:0019827	stem cell population maintenance	30/2210	146/18214	0.00252	0.0363	0.031881	BCL9L/LDB1/BCL9/ZHX2/SKI/KDM3A/FOXP1/SMAD4/PELO/SMC3/NIPBL/HES1/ZFP36L2/CNOT2/EIF4ENIF1/BMPR1A/LEO1/RIF1/WDR43/VPS72/TBX3/MCPH1/SOX4/MTF2/KLF10/MED7/ESRRB/NOG/FGF2/MIR145
GO:0031648	protein destabilization	13/2210	46/18214	0.00253	0.0363	0.031881	EP300/CREBBP/MUL1/MYLIP/BMP2/RNF139/SIAH1/SRC/FBXL3/SIRT1/PLK1/TRIM21/KDM8
GO:0033598	mammary gland epithelial cell proliferation	9/2210	26/18214	0.00253	0.0363	0.031881	ZNF703/KDM5B/RREB1/PYGO2/ID2/WNT5A/EPHA2/CEBPB/IQGAP3
GO:0035331	negative regulation of hippo signaling	6/2210	13/18214	0.00254	0.0363	0.031881	MARK3/MAPK14/AJUBA/LIMD1/VGLL4/CIT
GO:0043551	regulation of phosphatidylinositol 3-kinase activity	15/2210	57/18214	0.00265	0.03746	0.0329	PIK3R1/SOCS6/DAB2IP/PIK3R4/AMBRA1/SOCS4/IRS1/SRC/WDR81/PDGFB/PRKD1/CISH/PIK3R3/FGF2/TNFAIP8L3
GO:0051348	negative regulation of transferase activity	52/2210	294/18214	0.00327	0.04412	0.038756	INKA2/TRIB1/MYOCD/SPRY2/MIDN/ABL1/DUSP7/TRIB2/MLLT1/SPRY4/TRIM27/RGS3/DAB2IP/GGNBP2/CORO1C/APC/GSK3B/DUSP5/BAG5/AJUBA/TAF7/SMAD7/DUSP16/DUSP14/DUSP6/CHMP6/RPTOR/SPRY1/SOCS4/CEP85/ITPRIP/CDKN1B/NCK1/DUSP1/SRC/SPRED2/NUP62/CBLB/SIRT1/GADD45A/MEN1/SPRED1/DTX3L/DUSP10/CIT/DUSP18/PLK1/RGS14/DUSP8/FBXO5/TRIB3/TFAP4
GO:0071496	cellular response to external stimulus	53/2210	301/18214	0.00331	0.04456	0.039137	SLC38A2/MYOCD/MN1/XBP1/VCAM1/TNFRSF1A/MAP3K1/NFE2L2/FOXO1/MTMR3/FOXO3/SLC2A1/CNN2/TRIM24/JUN/PIM1/GAS2L1/TFEB/PIK3C2B/PIK3R4/RIPOR2/FADD/AMBRA1/SIK2/RPTOR/BAK1/SESN1/PPM1D/BCL10/MIOS/ZFYVE1/SESN2/NFKB1/EIF2AK3/RRP8/NUAK1/RNF152/KIF26A/SIRT1/GADD45A/ANKRD1/KLF10/DEPDC5/WDR24/IRF1/PRKD1/TBL2/MAP3K14/LRRK2/FAM107A/FOSL1/MIR125B1/NUAK2
GO:0070507	regulation of microtubule cytoskeleton organization	28/2210	137/18214	0.00372	0.04899	0.043033	NUMA1/ABL1/ARHGEF2/AKAP9/TAOK1/TPR/APC/DYRK1A/TPX2/GSK3B/TPPP/RNF4/GAS2L1/MARK2/MID1IP1/MAP1S/RAE1/TOGARAM1/NUP62/SKA1/SPAG5/PLK1/RASSF1/PSRC1/KIF18A/BORA/FAM107A/SKA3

GO Terms_ACLAvsDMSO_Upregulated							
ID	Description	GeneRatio	BgRatio	pvalue	p.adjust	qvalue	geneID
GO:0007389	pattern specification process	58/780	421/18214	2.8E-15	8.3E-12	6.77E-12	MAFB/ARC/PCDH8/SNAI1/LFNG/FOXC1/PTCH1/FZD5/MSX1/DLL1/NKX3-1/FOXC2/DLX2/RARG/DLL4/NEK8/NRARP/FST/WNT3/CFAP53/BMP4/ZIC3/GDNF/RIPPLY2/EDN1/C3/LHX1/EOMES/CYP26B1/NKX3-2/GBX2/EGR2/NR2F2/HES7/WNT1/ITGAM/CHRD/WNT2B/GREM1/SEMA3F/PLD6/GLI1/SFRP2/SIX2/TBR1/HHEX/LEFTY1/HOXD8/FGF8/FOXF1/RIPPLY1/CYP26C1/SIX1/SHH/DRC1/DNAI2/HES2/DLX1
GO:0003002	regionalization	50/780	326/18214	3.4E-15	8.3E-12	6.77E-12	MAFB/ARC/PCDH8/SNAI1/LFNG/FOXC1/PTCH1/FZD5/MSX1/DLL1/NKX3-1/FOXC2/DLX2/RARG/DLL4/NRARP/WNT3/BMP4/ZIC3/GDNF/RIPPLY2/EDN1/C3/LHX1/CYP26B1/GBX2/EGR2/NR2F2/HES7/WNT1/ITGAM/CHRD/WNT2B/GREM1/SEMA3F/PLD6/GLI1/SFRP2/SIX2/TBR1/HHEX/HOXD8/FGF8/FOXF1/RIPPLY1/CYP26C1/SIX1/SHH/HES2/DLX1
GO:0045165	cell fate commitment	38/780	251/18214	1.1E-11	1.8E-08	1.44E-08	ID2/SOX9/FZD7/PTCH1/EPOP/DLL1/FOXC2/KLF4/DLX2/GATA2/DLL4/GAS1/WNT3/BMP4/GATA3/IRF4/MCL1/EOMES/CYP26B1/SOX2/NR2F2/WNT1/WNT2B/DHH/OLIG2/PTF1A/SFRP2/SIX2/ATOH1/TBR1/FGF8/GDF7/POU3F2/SIX1/SHH/TAL1/DLX1/ISL2
GO:0007498	mesoderm development	21/780	123/18214	5.6E-08	9E-06	7.39E-06	SNAI1/FOXC1/FOXC2/KLF4/WNT3/BMP4/MIXL1/LHX1/EOMES/BTK/TLX2/HES7/OVOL1/ITGB4/AMH/SFRP2/SIX2/FGF8/FOXF1/SHH/TAL1
GO:0044706	multi-multicellular organism process	28/780	205/18214	5.7E-08	9E-06	7.39E-06	RGS2/H3-3B/TAC1/JUNB/FOSB/MAFF/CYP1A1/VDR/EDN1/GJB2/THBD/CNR1/OVGP1/CYP27B1/ADRA2C/AVPR1A/NR2F2/ITGB4/ABCC2/FOS/PTHLH/SLC6A4/HMX3/DKKL1/HAVCR2/PGR/PTAFR/TAC3
GO:0031960	response to corticosteroid	21/780	151/18214	1.9E-06	0.00014	0.000111	CCN2/SGK1/ERRFI1/ZFP36/DDIT4/BCL2L11/CDO1/FOSB/CLDN1/HTR1B/EDN1/GJB2/GPR83/AVPR1A/ABCC2/FOS/STAR/SSTR2/TPH2/PTAFR/SCN N1G
GO:0006334	nucleosome assembly	20/780	143/18214	3.1E-06	0.00019	0.000157	ASF1B/H2BC12/H2BC21/H2BC8/H3-3B/SOX9/TSPYL2/H3C10/H2BC17/CENPL/H2BC6/H2BC14/H3C4/H3C12/H1-1/H1-6/H4C7/H2BE1/H3-4/H4C13
GO:0010463	mesenchymal cell proliferation	10/780	38/18214	3.1E-06	0.00019	0.000157	SOX9/MSX1/MYC/BMP4/CHRD/SIX2/DCHS2/FOXF1/SIX1/SHH
GO:0031128	developmental induction	9/780	32/18214	5.3E-06	0.00029	0.000238	SOX9/FZD5/WNT3/BMP4/GDNF/WNT1/WNT2B/FGF8/SIX1
GO:0002699	positive regulation of immune effector process	25/780	216/18214	6.4E-06	0.00033	0.000273	HLA-E/FZD5/GATA2/HLA-B/NR4A3/GATA3/VAMP8/KLRD1/C3/NFKBID/BTK/MYB/TNFSF13/ITGAM/CLCF1/FGR/HLX/DHX58/IL12A/PTAFR/FOXF1/TNFRSF4/PGC/USP17L2/MZB1
GO:1901214	regulation of neuron death	31/780	303/18214	6.9E-06	0.00035	0.000283	CEBPB/DDIT4/BCL2L11/MDK/SIX4/NR4A3/NR4A2/NDNF/GATA3/PM20D1/GDNF/FOXQ1/MCL1/EN2/PITX3/CHGA/FZD9/MYB/WNT1/ITGAM/CLCF1/BDNF/FOS/EGR1/PRKCG/STAR/CSF3/FGF8/SIX1/CORO1A/DLX1
GO:0050867	positive regulation of cell activation	33/780	338/18214	9.5E-06	0.00044	0.000359	CCN2/PNP/HLA-E/TAC1/TNFSF9/GATA2/EGR3/MDK/IL1RL1/CEBPA/NR4A3/GATA3/VAMP8/SOX4/SOCS1/NFKBID/BTK/MYB/TNFSF13/ITGAM/CLCF1/FGR/TNFSF14/HAVCR2/BST1/BTLA/HLX/IL12A/PTAFR/TNFRSF4/CORO1A/SHH/RUNX3
GO:0002675	positive regulation of acute inflammatory response	8/780	27/18214	1.2E-05	0.00052	0.000424	HLA-E/TAC1/C2CD4A/C3/CNR1/BTK/PTGS2/OSM
GO:0019233	sensory perception of pain	15/780	99/18214	2E-05	0.00077	0.000633	TMEM100/SLC9A1/CXCR4/TAC1/KCNA1/EDN1/GRIN2D/FABP5/CNR1/ADRA2C/KCND2/HOXD1/PTAFR/ALOXE3/CCK
GO:0042401	cellular biogenic amine biosynthetic process	9/780	38/18214	2.5E-05	0.00094	0.000771	SAT1/GCH1/AZIN2/NR4A2/GATA3/TPH2/TPH1/INSM1/HDC
GO:0007219	Notch signaling pathway	21/780	186/18214	5E-05	0.00157	0.001284	SNAI1/TMEM100/LFNG/SOX9/FOXC1/DLL1/MYC/FOXC2/DLX2/GATA2/DLL4/CHAC1/MDK/NRARP/CEBPA/RIPPLY2/HES7/WNT1/PRAG1/ATOH1/DLX1
GO:0051953	negative regulation of amine transport	7/780	26/18214	8.3E-05	0.00235	0.00192	RGS2/SLC43A2/HTR1B/ADRA2A/CNR1/CHGA/ADRA2C

GO:0071772	response to BMP	19/780	166/18214	9.3E-05	0.00252	0.00206	SOX11/TMEM100/SOX9/MSX1/FST/BMP4/GATA3/WNT1/CHRD/GREM1/GDF9/EGR1/AMH/SFRP2/LEFTY1/GDF7/COMP/SMPD3/DLX1
GO:0071773	cellular response to BMP stimulus	19/780	166/18214	9.3E-05	0.00252	0.00206	SOX11/TMEM100/SOX9/MSX1/FST/BMP4/GATA3/WNT1/CHRD/GREM1/GDF9/EGR1/AMH/SFRP2/LEFTY1/GDF7/COMP/SMPD3/DLX1
GO:0006775	fat-soluble vitamin metabolic process	9/780	45/18214	0.0001	0.00267	0.002182	SNAI1/CYP1A1/VDR/CYP26B1/CYP27B1/CYP26A1/LRAT/GFI1/CYP26C1
GO:0051090	regulation of DNA-binding transcription factor activity	36/780	443/18214	0.00019	0.00429	0.0035	ID1/ID2/SGK1/PTCH1/HSPA1B/NKX3-1/KLF4/HSPA1A/CTH/ZC3H12A/CYP1B1/RGCC/NR0B1/EDN1/EOMES/ZIC2/BTK/SPHK1/WNT1/NEUROG2/GREM1/FOS/TRIML2/AMH/RHEBL1/GFI1/HAVCR2/CSF3/NKX6-1/NWD1/NHLH2/TNFRSF4/NLRC4/ESR2/SHH/NLRC3
GO:0001819	positive regulation of cytokine production	35/780	435/18214	0.00027	0.0058	0.004737	CEBPB/HLA-E/HSPA1B/FZD5/IRF7/HSPA1A/MDK/IL1RL1/NR4A3/PTGER4/CYP1B1/NLRP10/ADRA2A/GATA3/RGCC/IRF4/C3/CD14/MYB/SPHK1/EREG/EGR1/PTGS2/FGR/HAVCR2/CGAS/INAVA/DHX58/IL12A/AZU1/OSM/PTAFR/NLRC4/SERPINF2/GPSM3
GO:0035265	organ growth	18/780	166/18214	0.00028	0.00581	0.004742	RGS2/SOX9/FOXC1/DLL1/FOXC2/BCL2L11/RARG/EDN1/PSAPL1/MATN1/NPPC/SLC6A4/GLI1/HLX/FGF8/COMP/SHH/SMPD3
GO:0045833	negative regulation of lipid metabolic process	13/780	100/18214	0.00034	0.00676	0.005519	SNAI1/SOX9/PLIN5/MFSD2A/ADRA2A/NR0B1/CNR1/CYP27B1/SPHK1/FGF19/DKKL1/GFI1/CYP7A1
GO:0032965	regulation of collagen biosynthetic process	8/780	42/18214	0.00035	0.00701	0.005724	CCN2/ERRFI1/BMP4/RGCC/MYB/NPPC/SERPINF2/CYP7A1
GO:2000241	regulation of reproductive process	17/780	157/18214	0.00041	0.00787	0.006426	SNAI1/SOX9/MSX1/NKX3-1/PHLDA2/BMP4/TACR1/CNR1/OVGP1/OVOL1/RNASE10/NPPC/GDF9/INHBB/AMH/PTAFR/SHH
GO:0000353	formation of quadruple SL/U4/U5/U6 snRNP	4/780	10/18214	0.00057	0.00988	0.008066	RNU4-1/RNU4-2/RNU5E-1/RNU5B-1
GO:0000365	mRNA trans splicing, via spliceosome	4/780	10/18214	0.00057	0.00988	0.008066	RNU4-1/RNU4-2/RNU5E-1/RNU5B-1
GO:0045291	mRNA trans splicing, SL addition	4/780	10/18214	0.00057	0.00988	0.008066	RNU4-1/RNU4-2/RNU5E-1/RNU5B-1
GO:0001558	regulation of cell growth	32/780	403/18214	0.00062	0.01049	0.008563	RGS2/SGK1/HBEGF/SLC9A1/H3-3B/NANOS1/CXCR4/CDKN2AIP/TSPYL2/HSPA1B/MSX1/HSPA1A/CRABP2/MFSD2A/WNT3/EDN1/SEMA3G/CYP27B1/AVPR1A/SPHK1/GREM1/BDNF/GDF9/SEMA3F/SFRP2/IGFBPL1/NKX6-1/BST2/ESR2/TMC8/CPNE9/GNG4
GO:0018958	phenol-containing compound metabolic process	13/780	107/18214	0.00065	0.01097	0.008957	GCH1/GCNT4/AOC2/NR4A2/GATA3/SLC5A5/ITGAM/DIO3/STAR/TPH2/TPH1/INSM1/HDC
GO:0007584	response to nutrient	17/780	164/18214	0.00068	0.01122	0.009158	AQP3/CDKN2B/LIPG/CYP1A1/VDR/C2/CYP26B1/CNR1/CYP27B1/SLC6A4/CYP26A1/LRAT/STAR/SFRP2/ABCG8/PKLR/SERPINC1
GO:0003018	vascular process in circulatory system	22/780	245/18214	0.00088	0.01385	0.011303	RGS2/GCH1/FOXC1/FOXC2/C2CD4A/MFSD2A/HTR1B/ADRA2A/EDN1/SLC5A5/FABP5/ADRA2C/AVPR1A/SLC6A20/SLC8A2/ABCB1/ABCC2/SLC6A4/AZU1/PTAFR/COMP/SERPINF2
GO:0002822	regulation of adaptive immune response based on somatic recombination of immune receptors built from immunoglobulin superfamily domains	16/780	153/18214	0.00088	0.01385	0.011303	HLA-E/FZD5/IL1RL1/HLA-B/ZC3H12A/NLRP10/GATA3/KLRD1/C3/NFKBID/BTK/TNFSF13/CLCF1/HAVCR2/HLX/IL12A
GO:0045576	mast cell activation	9/780	60/18214	0.00096	0.01462	0.011931	NDRG1/GATA2/NR4A3/VAMP8/CNR1/CHGA/BTK/FGR/FOXF1

GO:0000079	regulation of cyclin-dependent protein serine/threonine kinase activity	12/780	99/18214	0.00106	0.01588	0.012962	HEXIM1/CKS2/CCNE2/CCNG2/CDKN2B/CEBPA/RGCC/NR2F2/CCNA1/CCNI2/HERC5/HHEX
GO:0045933	positive regulation of muscle contraction	7/780	39/18214	0.00119	0.0173	0.014119	CCN2/RGS2/EDN1/CHGA/SPHK1/ATP2A1/PTAFR
GO:0014072	response to isoquinoline alkaloid	6/780	29/18214	0.00123	0.01741	0.014212	CXCR4/TAC1/FOSB/CNR1/PITX3/PRKCG
GO:0043278	response to morphine	6/780	29/18214	0.00123	0.01741	0.014212	CXCR4/TAC1/FOSB/CNR1/PITX3/PRKCG
GO:0062014	negative regulation of small molecule metabolic process	12/780	101/18214	0.00126	0.0175	0.014284	SNAI1/SOX9/PLIN5/DDIT4/MFSD2A/CNR1/CYP27B1/FGF19/DKKL1/GFI1/PDZD3/CYP7A1
GO:0048638	regulation of developmental growth	26/780	318/18214	0.00127	0.01757	0.01434	RGS2/CXCR4/FOXC1/PTCH1/DLL1/FOXC2/BCL2L11/CRABP2/MFSD2A/SIX4/WNT3/BMP4/EDN1/SEMA3G/DIO3/BDNF/NPPC/SEMA3F/SLC6A4/GLI1/HLX/NKX6-1/FGF8/POU3F2/SIX1/CPNE9
GO:0030277	maintenance of gastrointestinal epithelium	5/780	22/18214	0.00204	0.02466	0.020128	SOX9/MUC6/MUC13/INAVA/MUC4
GO:0031280	negative regulation of cyclase activity	5/780	22/18214	0.00204	0.02466	0.020128	LTB4R2/GALR3/GALR2/PDZD3/GRM2
GO:0043300	regulation of leukocyte degranulation	7/780	43/18214	0.00215	0.02563	0.020915	GATA2/VAMP8/ITGAM/PRAM1/FGR/PTAFR/FOXF1
GO:0045840	positive regulation of mitotic nuclear division	7/780	43/18214	0.00215	0.02563	0.020915	NUSAP1/RGCC/EDN1/SPHK1/EREG/FGF8/SMPD3
GO:0019731	antibacterial humoral response	8/780	57/18214	0.00281	0.0313	0.02555	H2BC12/H2BC21/H2BC8/HLA-E/H2BC6/TF/WFDC3/PGC
GO:0001906	cell killing	16/780	175/18214	0.00354	0.03677	0.030008	H2BC12/HMGN2/HLA-E/BCL2L11/HLA-B/ULBP2/KLRD1/C3/ULBP1/CHGA/ITGAM/C9/HAVCR2/IL12A/AZU1/CORO1A
GO:0002448	mast cell mediated immunity	7/780	47/18214	0.00363	0.03735	0.030482	GATA2/NR4A3/VAMP8/CHGA/BTK/FGR/FOXF1
GO:0035902	response to immobilization stress	5/780	25/18214	0.0037	0.03735	0.030482	CYP1A1/NR0B1/PITX3/FOS/TPH1
GO:0099560	synaptic membrane adhesion	5/780	25/18214	0.0037	0.03735	0.030482	PCDH8/PCDH17/NTNG2/MDGA1/SLITRK3
GO:0030033	microvillus assembly	4/780	16/18214	0.00402	0.03995	0.032605	KLF5/FSCN1/MYO1A/ESPN
GO:0009914	hormone transport	24/780	313/18214	0.00438	0.04261	0.034777	SOX11/MAFA/TAC1/NKX3-1/NMB/ADRA2A/GATA3/VAMP8/SOX4/NNAT/EDN1/CNR1/CHGA/ADRA2C/BTK/MYB/ABCC2/GDF9/INHBB/NKX6-1/OSM/IL11/SMPD3/CACNA1E
GO:0006817	phosphate ion transport	5/780	26/18214	0.00442	0.04261	0.034777	SLC20A1/CEBPB/SLC17A7/SLC34A3/SLC37A2
GO:0051782	negative regulation of cell division	4/780	17/18214	0.00508	0.04741	0.038694	TEX14/PTCH1/MYC/E2F7
GO:0045823	positive regulation of heart contraction	6/780	38/18214	0.00517	0.0479	0.039094	CCN2/RGS2/GCH1/EDN1/CHGA/AVPR1A
GO:0042267	natural killer cell mediated cytotoxicity	8/780	63/18214	0.00527	0.04794	0.039126	HLA-E/HLA-B/ULBP2/KLRD1/ULBP1/HAVCR2/IL12A/CORO1A

GO Terms_ACLAvsDMSO_Downregulated							
ID	Description	GeneRatio	BgRatio	pvalue	p.adjust	qvalue	geneID
GO:0007264	small GTPase mediated signal transduction	108/1707	497/18214	4.1E-17	2.3E-13	1.98E-13	RAPGEF2/ARHGAP31/WASF2/AKAP13/CGNL1/DAB2IP/SYNPO2L/KANK1/ITPKB/SIPA1L2/ARHGAP24/ARHGEF2/USP28/MYO9A/OBSCN/SRGAP1/RREB1/RHOBTB2/MAPK14/SOS1/IQSEC1/ROCK2/DOCK9/KANK2/STARD13/SRGAP2/ARFGEF1/ARHGEF7/MADD/PIK3CB/RIPOR2/RALGAPB/GBF1/SPRY4/PLD1/ARHGAP26/AUTS2/KSR1/TIAM2/NF1/TRIO/RALGDS/DENND4C/RGL1/ERBIN/KALRN/ARHGEF11/RIN2/OPHN1/SIPA1L1/CUL3/ARHGAP21/ARFIP2/CHN2/ARAP1/SETDB1/VAV2/RAB33A/ARFGEF2/CYTH3/ABL1/SYDE1/RHOT1/BCR/ARHGAP42/ARF6/SIPA1L3/ARRB1/SPRY2/DNAJA3/RASA3/RAB9A/RALA/RAB35/ABL2/ARHGEF18/NUP62/RASA1/ARHGAP10/RTKN/RFXANK/PRKD1/SRC/RAB30/ARHGAP39/CYTH1/RABIF/ARHGAP32/ARHGAP6/GPSM2/PREX1/RAP1GAP2/ARHGAP44/SPRY1/INPP5B/ARHGEF3/CCNA2/ARHGEF16/DOCK4/RAPGEF4/F2RL3/ARHGAP18/FGF2/RGS19/RAB42/RTN4R/ARHGAP28/RHOU
GO:0016570	histone modification	83/1707	448/18214	1E-09	1.9E-06	1.67E-06	MYOCD/KDM6A/NSD1/UBR5/PPARGC1A/KDM4B/KMT2C/KAT6B/ASXL1/SKI/CREBBP/KMT2E/USP7/TBL1X/UBR2/BAZ1B/HR/NIPBL/EP300/HDAC9/USP22/EHMT1/SRCAP/NCOA1/MTHFR/MCRS1/WDR82/WAC/RCOR1/NCOA3/AUTS2/PHF20L1/MECP2/YEATS2/KDM2A/RCOR2/KAT14/TET3/IWS1/KAT7/BRD4/KDM4A/SMARCB1/TRIM16/KAT5/TADA2B/SETDB1/DCAF1/TADA2A/KDM4C/RLF/MIER2/ARRB1/HDAC7/SMARCAD1/ZZZ3/UFL1/MIDEAS/PHF20/SPHK2/PRDM16/JARID2/RIOX2/MSL3P1/PHF14/PWP1/PRKD1/INCENP/HDAC4/TRERF1/FBXL19/BRCA1/JADE3/WDR70/DTX3L/N6AMT1/RPS6KA4/RNF2/JADE2/CCNA2/AURKB/SETDB2/YEATS4
GO:0030010	establishment of cell polarity	36/1707	136/18214	6.3E-09	5.8E-06	5.11E-06	PAFAH1B1/KIF26B/GOLPH3/GSK3B/NUMA1/FRMD4B/KANK1/MYO9A/ALPK2/AMOTL1/ROCK2/ARFGEF1/MARK2/PARD3/RIPOR2/GBF1/MPP7/ARHGEF11/OPHN1/RNF41/FERMT1/STK11/CYTH3/ARF6/SIPA1L3/SPRY2/PRKCZ/CYTH1/PARD3B/GPSM2/FRMD4A/ZW10/NDC80/SPRY1/UST/WWC1
GO:0043087	regulation of GTPase activity	84/1707	485/18214	2.1E-08	1.5E-05	1.3E-05	EPHB3/RAPGEF2/ARHGAP31/GSK3B/TBC1D14/DAB2IP/SH3BP4/SIPA1L2/RGS3/ARHGAP24/MYO9A/RANBP2/SRGAP1/CORO1C/AGAP1/SOS1/IQSEC1/DOCK9/ARHGEF10L/STARD13/SRGAP2/ARFGEF1/ARHGEF7/RIPOR2/SNX9/RASAL2/RALGAPB/ARHGAP26/TBC1D4/GIT2/GAPVD1/TIAM2/MTSS2/NF1/RRP1B/KALRN/ARHGEF11/RIN2/OPHN1/SIPA1L1/ARHGAP21/AGFG1/TBC1D20/GPSM1/CHN2/TBC1D25/ARAP1/VAV2/PLXNA2/SYDE1/ADPRH/LRCH1/BCR/RIC1/ARHGAP42/SIPA1L3/PREB/ARRB1/SPRY2/RASA3/ACAP2/RUNDC1/RASA1/ARHGAP10/USP6NL/RTKN/GNAQ/ARHGAP39/PRKG1/ARHGAP32/ARHGAP6/PREX1/RAP1GAP2/ARHGAP44/RIN3/SPRY1/ARHGAP27/RGS8/RGS6/ARHGEF16/DOCK4/ARHGAP18/RTN4R/ARHGAP28
GO:0086003	cardiac muscle cell contraction	23/1707	72/18214	8.2E-08	4.6E-05	3.98E-05	GATA4/GJC1/HCN4/KCNA5/CACNB2/PKP2/AKAP9/KCNJ8/PDE4B/ADORA1/DLG1/CACNA1D/KCNN2/NEDD4L/KCNJ5/NOS1AP/GJA5/KCNJ3/SCN2B/KCNE4/KCND3/MIR1-1/KCNE3
GO:0031032	actomyosin structure organization	39/1707	190/18214	2.2E-06	0.00071	0.000619	WASF2/AKAP13/FRMD3/PIK3R1/ANKRD1/CGNL1/SYNPO2L/SMAD3/LMOD2/OBSCN/PHACTR1/CNN2/NF2/ROCK2/ARHGEF10L/MYPN/FRMD5/LMOD1/CUL3/CARMIL1/ARAP1/ABL1/EPB41/EPB41L4A/PHLDB2/KRT19/PPFIA1/ARRB1/ARHGEF18/RTKN/SRC/LMOD3/ARHGAP6/LURAP1/ALKBH4/CIT/CORO2B/MIR1-1/ARHGAP28
GO:0055017	cardiac muscle tissue growth	24/1707	92/18214	2.7E-06	0.00079	0.000694	TENM4/GATA4/AKAP6/AKAP13/TBX20/ZFPM2/BMPR1A/MAPK14/TBX5/PPARA/PIM1/MEF2C/VGLL4/PTEN/ARID2/MEIS1/PARP2/GATA6/RBPJ/JARID2/FGFR2/MIR208A/FGF2/MIR1-1
GO:0086001	cardiac muscle cell action potential	21/1707	76/18214	4.2E-06	0.00097	0.000852	GJC1/HCN4/KCNA5/CACNB2/PKP2/AKAP9/KCNJ8/DLG1/CACNA1D/KCNN2/NEDD4L/KCNJ5/NOS1AP/GJA5/MIR208A/KCNJ3/SCN2B/KCNE4/KCND3/MIR1-1/KCNE3
GO:0033044	regulation of chromosome organization	49/1707	273/18214	6.7E-06	0.00143	0.001255	NUMA1/PPARGC1A/YLPM1/TPR/KMT2E/USP7/NIPBL/APC/CDC27/AUTS2/CENPF/MECP2/RAD50/WAPL/HNRNPD/KAT7/BRD4/MAP2K7/SMARCB1/CUL3/TADA2B/SETDB1/TNKS/RNF4/TADA2A/HMBOX1/ARRB1/HECW2/SPHK2/NBN/DKC1/BUB1B/JARID2/PARN/PRKD1/CENPE/SRC/CTC1/ERCC4/KLHL22/BRCA1/PSMG2/ZW10/NDC80/RPS6KA4/POT1/AURKB/SETDB2/CDT1
GO:0009896	positive regulation of catabolic process	71/1707	448/18214	7.2E-06	0.00146	0.00128	PRICKLE1/IRS1/GSK3B/DAB2IP/SH3BP4/CNOT1/TNRC6B/KEAP1/TRIB1/FOXO1/ZBTB20/MAGEF1/ROCK2/VPS13D/NKD1/APC/PIK3CB/SNX9/COP1/WAC/GIGYF2/SMURF2/PPARA/PSMC2/DAPK1/TFEB/VGLL4/HNRNPD/PTEN/PAFAH1B2/FOXO3/TRIM32/RNF41/PIP4K2A/GPSM1/CPT1A/SPTLC2/SMURF1/KAT5/ADORA1/ARNT/STK11/PIAS1/CNOT6L/EIF2AK4/TRIB2/AMBRA1/TRIM27/WWP2/PFKFB4/FZR1/HECW2/ESRRB/UFL1/NEDD4L/RNF180/MEX3D/TOB1/PRKCE/PRKD1/TICAM1/TRIM65/TBK1/RNF152/DTX3L/NOD1/ABCD2/DTL/TRIM68/DET1/TRIM6
GO:0001701	in utero embryonic development	56/1707	334/18214	1.3E-05	0.00201	0.001758	HAND2/ZMIZ1/MYO18B/TANC2/SMAD3/CNOT1/ZFPM2/KEAP1/ACVR1B/BMPR1A/ZFAND5/EDNRA/NCOA1/BIRC6/TFEB/GLI2/NSRP1/SH3PXD2A/CMIP/GLI3/CUL3/GATA6/MBNL1/ARNT/SEC24D/MYO1E/TAF8/SLC35E2B/SMG9/CNOT2/PELO/KRT19/TAB1/E2F8/NXN/GGNBP2/PRPF19/RBPJ/NBN/RUNDC1/BMP2/JAG2/FGFR2/CCM2/CHST11/RSPO3/TRAF6/TTLL1/FUT8/INPP5B/ARNT2/MDFI/ELL/SCO2/CASP8/AKAP3

GO:0006352	DNA-templated transcription, initiation	44/1707	244/18214	1.7E-05	0.00239	0.002093	NKX2.5/PPARGC1A/MITF/CREBBP/NCOA6/RARA/RXRA/NR2C2/TBX5/MAML3/CREB1/CDK8/PPARA/PSMC2/MECP2/H4C5/NR2F6/PPARD/MAML2/PTEN/BRF1/SMARCB1/RSF1/TAF8/POLR1B/ESRRG/RORA/CDK4/ESRRB/RBPJ/ZNF45/TAF4/GTF2E1/H4C2/E2F3/POLRMT/BRF2/GTF2F2/TAF3/MED27/RARB/TEAD4/TFB1M/H4C3
GO:0043470	regulation of carbohydrate catabolic process	22/1707	90/18214	2.1E-05	0.00276	0.002416	PPARGC1A/PPP1R3B/TPR/RANBP2/NCOR1/ZBTB20/EP300/PRKAG2/PPARA/NUP153/NUP210/NUP98/SLC25A12/GCK/ARNT/NUP85/PFKFB4/ESRRB/NUP62/ZBTB7A/NUP37/HDAC4
GO:0014855	striated muscle cell proliferation	19/1707	72/18214	2.4E-05	0.00307	0.002685	TENM4/TBX20/ZFPM2/BMPR1A/MAPK14/TBX5/PIM1/MEF2C/PPARD/VGLL4/PTEN/ARID2/MEIS1/GATA6/RBPJ/JARID2/FGFR2/FGF2/MIR1-1
GO:0006110	regulation of glycolytic process	20/1707	79/18214	2.9E-05	0.00342	0.002996	PPARGC1A/TPR/RANBP2/NCOR1/ZBTB20/EP300/PRKAG2/PPARA/NUP153/NUP210/NUP98/GCK/ARNT/NUP85/PFKFB4/ESRRB/NUP62/ZBTB7A/NUP37/HDAC4
GO:0043467	regulation of generation of precursor metabolites and energy	32/1707	160/18214	3E-05	0.00342	0.002996	IRS1/GSK3B/PPARGC1A/PPP1R3B/TPR/RANBP2/NCOR1/PPP1R3C/ZBTB20/TACO1/EP300/PRKAG2/DYRK2/PPARA/NUP153/NUP210/NUP98/SLC25A12/GCK/PPP1R3A/ARNT/NUP85/PFKFB4/ESRRB/NUP62/SPHK2/PRDM16/DNAJC30/ZBTB7A/NUP37/HDAC4/ETFBKMT
GO:0086009	membrane repolarization	14/1707	45/18214	3.9E-05	0.00402	0.003519	AKAP6/KCNA5/AKAP9/KCNJ8/CACNA1D/KCNN2/NEDD4L/KCNJ5/NOS1AP/KCNJ3/KCNE4/KCND3/MIR1-1/KCNE3
GO:1903829	positive regulation of cellular protein localization	49/1707	295/18214	5.5E-05	0.00495	0.00433	PIK3R1/GSK3B/UBR5/PPP1R13B/MAVS/NUMA1/SMAD3/TPR/MAPK14/FYN/NIPBL/ROCK2/PTPN9/APC/SSH1/ZDHHC5/MCRS1/PPP1R9B/PDPK1/KAT7/GLI3/EGFR/STK11/LRIG2/EPB41/BICD1/ARF6/DLG1/PPP3CC/AKAP5/MGAT3/KCNJ11/MIEF1/NUP62/DKC1/GLIS2/MTCL1/MEAK7/PRKCE/SRC/DTX3L/GPSM2/NDC80/BBC3/ARHGEF16/EFCAB7/CASP8/CDT1/RHOU
GO:0071559	response to transforming growth factor beta	44/1707	256/18214	5.7E-05	0.00505	0.004415	MYOCD/ZMIZ1/ANKRD1/PPARGC1A/BCL9L/SMAD3/SKI/CREBBP/GCNT2/SMAD6/BMPR1A/FYN/LDLRAD4/ROCK2/EP300/ZFYVE9/PARD3/CREB1/PTPRK/SMURF2/PPARA/MEF2C/PDPK1/PMEPA1/ZFHX3/SMURF1/FERMT1/STK11/TAB1/TRIM33/SPRY2/FNTA/ARHGEF18/SPRED2/PRDM16/ZBTB7A/TGFBRAP1/PRKCZ/FGFR2/SRC/CHST11/SPRY1/FUT8/SOX5
GO:0007623	circadian rhythm	38/1707	212/18214	7.2E-05	0.00621	0.005434	USP2/GSK3B/PPARGC1A/USP7/NCOR1/ROCK2/EP300/BTRC/RAI1/NCOA2/CREB1/BHLHE40/THRAP3/PPARA/KDM2A/LGR4/NR2F6/BTBD9/HNRNPD/PTEN/ZFHX3/MYCBP2/GNA11/CPT1A/SRRD/EGFR/SPSB4/ADORA1/DBP/RORA/SERPINE1/CDK4/GNAQ/FBXL22/PHLPP1/MAGEL2/CRTC1/NPAS2
GO:0070988	demethylation	18/1707	73/18214	0.0001	0.00813	0.007109	KDM6A/KDM4B/APOBEC2/USP7/HR/KDM2A/TET3/MMACHC/KDM4A/PPME1/KDM4C/JARID2/RIOX2/FBXL19/ALKBH3/ALKBH4/TOX/APOBEC3G
GO:0000075	cell cycle checkpoint	37/1707	208/18214	0.0001	0.00813	0.007109	TAOK1/TNKS1BP1/FOXN3/USP28/TPR/CNOT1/MAPK14/EP300/APC/FEM1B/WAC/CENPF/ARID3A/TP53BP1/TAOK3/TAOK2/CNOT6L/MUS81/CNOT2/EIF2AK4/E2F8/FZR1/PRPF19/FBXO31/CNOT10/NBN/BUB1B/TRIAP1/KLHL22/BRCA1/PSMG2/ZW10/NDC80/DTL/AURKB/BLM/CDT1
GO:0042692	muscle cell differentiation	56/1707	361/18214	0.00012	0.00813	0.007109	LRRC10/FBXO40/MYOCD/NKX2-5/GATA4/RBM38/AKAP6/FHL2/AKAP13/ANKRD1/SYNPO2L/MYO18B/SKI/PLPP7/SYNE1/LMOD2/ANKRD17/OBSCN/AFG3L2/MAPK14/MRTFA/SPEG/MRTFB/ALPK2/HDAC9/WNT5B/TBX5/RIPOR2/PPARA/MECP2/NFATC2/MEF2C/MYPN/BCL9/LMOD1/TRIM32/TANC1/ASF1A/PARP2/POPDC3/GATA6/PIAS1/ABL1/KRT19/RORA/RBPJ/BMP2/IL4R/FGFR2/HDAC4/LMOD3/RARB/MIR208A/NKX2-6/MIR145/MIR1-1
GO:0060968	regulation of gene silencing	28/1707	142/18214	0.00012	0.00813	0.007109	MYOCD/H1-4/TPR/RANBP2/TNRC6B/NCOR1/PUM2/LIMD1/H1-0/H1-2/NUP153/NCOR2/NUP210/H4C5/NUP98/IPO8/ASF1A/EGFR/NUP85/H3C2/NUP62/H4C2/NUP37/H1-3/BCDIN3D/H3C8/H4C3/RMRP
GO:0030048	actin filament-based movement	29/1707	150/18214	0.00013	0.00851	0.007442	GATA4/WASF2/GJC1/HCN4/KCNA5/CACNB2/PKP2/AKAP9/EMP2/KCNJ8/PDE4B/MYO1B/WIPF2/ADORA1/MYO1E/DLG1/CACNA1D/KCNN2/NEDD4L/KCNJ5/NOS1AP/GJA5/WIPF1/KCNJ3/SCN2B/KCNE4/KCND3/MIR1-1/KCNE3
GO:0097581	lamellipodium organization	20/1707	89/18214	0.00017	0.00995	0.008707	WASF2/GOLPH3/PIK3R1/KANK1/RREB1/CORO1C/SRGAP2/ARHGEF7/SPATA13/AUTS2/MTSS2/ARFIP2/CARMIL1/VAV2/FER/NUP85/NCK1/SRC/HDAC4/ABLIM2
GO:0075733	intracellular transport of virus	15/1707	57/18214	0.00018	0.00995	0.008707	TPR/RANBP2/VPS37B/NUP153/NUP210/NUP98/XPO1/UBAP1/NUP85/MVB12B/KPNA3/NUP62/VPS37C/NUP37/VPS37D
GO:0001952	regulation of cell-matrix adhesion	25/1707	124/18214	0.00018	0.01009	0.00883	PIK3R1/GSK3B/MACF1/PEAK1/SMAD3/CASK/CORO1C/EMP2/PLEKHA2/NF2/ROCK2/PIK3CB/CDK6/NF1/PTEN/RIN2/FERMT1/ABL1/PHLDB2/SERPINE1/RASA1/PRKCZ/SRC/ARHGAP6/FAM107A
GO:2001224	positive regulation of neuron migration	7/1707	15/18214	0.00021	0.01085	0.00949	RAPGEF2/DAB2IP/ZNF609/ARHGEF2/NIPBL/PLAA/FBXO31

GO:1901990	regulation of mitotic cell cycle phase transition	64/1707	437/18214	0.00022	0.0112	0.0098	PAFAH1B1/TAOK1/KCNA5/TNKS1BP1/FOXN3/AKAP9/TPR/CNOT1/KMT2E/CUL1/ANKRD17/PBX1/KANK2/EP300/BTRC/RPTOR/APC/PCM1/CDC27/NACC2/CENPF/PSMC2/MECP2/ARID3A/FBXL7/PPP1R9B/CDK6/BRD4/PTEN/CUL3/TAOK3/TAOK2/EGFR/OFD1/TPX2/CNOT6L/CNOT2/E2F8/DLG1/FZR1/CTDSPL/HECW2/LSM10/CDK4/FBXO31/CNOT10/NBN/BUB1B/CEP290/TRIAP1/CENPE/CTC1/KLHL22/BRCA1/PSMG2/ZW10/NDC80/MIR208A/DTL/AURKB/BLM/FAM107A/TFAP4/CDT1
GO:0001570	vasculogenesis	18/1707	78/18214	0.00026	0.01265	0.011063	MYOCD/NKX2-5/RAPGEF2/ZMIZ1/GJC1/TBX20/MYO18B/ZFPM2/EMP2/GDF2/TBX5/RIN2/FZD4/AGGF1/MYO1E/RASA1/CCM2/MIR1-1
GO:0150115	cell-substrate junction organization	22/1707	106/18214	0.00029	0.01326	0.011599	PIK3R1/MACF1/PEAK1/SMAD3/CORO1C/PLEC/IQSEC1/ROCK2/ARHGEF7/PTPRK/PDPK1/PTEN/TAOK2/ABL1/PHLDB2/BCR/ARF6/TRIP6/SRC/ARHGAP6/CORO2B/FAM107A
GO:0009135	purine nucleoside diphosphate metabolic process	26/1707	135/18214	0.0003	0.0136	0.011896	FOXK1/PPARGC1A/TPR/RANBP2/FOXK2/CASK/NCOR1/ZBTB20/EP300/PRKAG2/PPARA/NUP153/NUP210/NUP98/GCK/ARNT/NUP85/MAGI3/DLG1/PFKFB4/ESRRB/NUP62/ZBTB7A/NUP37/HDAC4/NUDT18
GO:0001933	negative regulation of protein phosphorylation	54/1707	360/18214	0.00036	0.01528	0.013366	MYOCD/PPARGC1A/DAB2IP/KLHL31/MLLT1/RGS3/BAK1/INKA2/SMAD6/TRIB1/CORO1C/DUSP7/NF2/LDLRAD4/RPTOR/PARD3/APC/PIK3CB/PRKAG2/SPRY4/CHMP6/PDPK1/NF1/PTEN/PMEPA1/TAOK3/LRP6/DUSP14/ABL1/NCK1/TRIB2/IBTK/ARRB1/SPRY2/GGNBP2/TRIM27/CTDSPL/SPRED2/NUP62/PLPP3/PRKCZ/GNAQ/PWP1/TRAF3IP1/SPRY1/DUSP19/DTNBP1/UBASH3B/CIT/ZFYVE28/PBK/TFAP4/DUSP18/MIR27B
GO:0051983	regulation of chromosome segregation	19/1707	87/18214	0.00037	0.01534	0.013417	NUMA1/TPR/MKI67/PUM2/APC/CDC27/CENPF/WAPL/CUL3/HECW2/BUB1B/CENPE/KLHL22/PSMG2/ZW10/NDC80/AURKB/PLSCR1/CDT1
GO:0070507	regulation of microtubule cytoskeleton organization	26/1707	137/18214	0.00038	0.01539	0.01346	TAOK1/GSK3B/NUMA1/ARHGEF2/AKAP9/TPR/MARK2/APC/TPPP/GAS2L1/MECP2/DRG1/TRIM54/TTBK2/TPX2/RNF4/ABL1/PHLDB2/BICD1/PRUNE1/NUP62/NAV3/TRAF3IP1/GPSM2/SKA1/FAM107A
GO:0016233	telomere capping	14/1707	55/18214	0.00042	0.01658	0.014504	USP7/RAD50/H4C5/HNRNPD/MAP2K7/TNKS/NBN/DCLRE1A/H4C2/CTC1/ERCC4/POT1/AURKB/H4C3
GO:1903578	regulation of ATP metabolic process	23/1707	116/18214	0.00042	0.01665	0.014566	PPARGC1A/TPR/RANBP2/NCOR1/ZBTB20/EP300/PRKAG2/PPARA/NUP153/NUP210/NUP98/SLC25A12/GCK/ARNT/NUP85/PFKFB4/ESRRB/NUP62/SPHK2/DNAJC30/ZBTB7A/NUP37/HDAC4
GO:0030166	proteoglycan biosynthetic process	15/1707	62/18214	0.00048	0.01837	0.016069	NDST1/EXT1/TCF7L2/XYLT2/CHST10/CHST3/CHSY1/CANT1/B3GALT6/XYLT1/CHST11/CHSY3/HS3ST3B1/PXYLP1/UST
GO:0032970	regulation of actin filament-based process	56/1707	382/18214	0.00051	0.01929	0.016872	GATA4/WASF2/SSH2/HCN4/AKAP13/TAOK1/PIK3R1/CGNL1/SYNPO2L/KANK1/PKP2/SMAD3/SYNPO2/AKAP9/LMOD2/CNN2/RHOBTB2/NF2/ROCK2/ARHGEF10L/ARFGEF1/PDE4B/SSH1/SNX9/SPIRE1/LMOD1/FCHSD2/ARFIP2/CARMIL1/TAOK2/ARAP1/ADORA1/FER/ABL1/NCK1/PHLDB2/ARF6/DLG1/PPFIA1/TRIM27/ABL2/ARHGEF18/RASA1/PRKCE/GJA5/LMOD3/ARHGAP6/PREX1/ARHGAP44/MAGEL2/CORO2B/FAM107A/ARHGAP18/NAA80/MIR1-1/ARHGAP28
GO:0032506	cytokinetic process	11/1707	38/18214	0.00053	0.01972	0.017252	KLHDC8B/SNX9/SPIRE1/CHMP6/CHMP7/NUP62/KIF20A/RTKN/ALKBH4/CEP55/AURKB
GO:0060562	epithelial tube morphogenesis	47/1707	309/18214	0.00062	0.02174	0.019022	NKX2.5/PRICKLE1/HAND2/GATA4/KIF26B/TBX20/IRX3/SMAD3/SKI/PBX1/FOXP1/LUZP1/BTRC/EDNRA/STARD13/GDF2/EXT1/SEC24B/MTHFR/MEF2C/GLI2/LGR4/SHROOM3/MTSS1/GLI3/LRP6/LMO4/KLHL3/ABL1/DLG1/SUFU/SPRY2/RALA/RBPJ/FZD2/CEP290/BMP2/FGFR2/CCM2/SRC/IFT172/TRAF6/SPRY1/SETDB2/IRX1/FGF2/PRKX
GO:0033692	cellular polysaccharide biosynthetic process	16/1707	71/18214	0.00073	0.02396	0.020965	IRS1/GSK3B/NDST1/PPP1R3B/PPP1R3C/EXT1/DYRK2/GCK/PPP1R3A/B3GNT9/ESRRB/B3GALT2/B3GNT8/B3GALT1/B3GALT5/NHLRC1
GO:0032200	telomere organization	30/1707	174/18214	0.00077	0.02473	0.021633	TNKS1BP1/YLPM1/USP7/RFC1/RAD50/H4C5/HNRNPD/MAP2K7/TNKS/HMBOX1/H3C2/NBN/DKC1/POLA2/DCLRE1A/H4C2/RAD51D/PARN/SRC/CTC1/ERCC4/SMARCAL1/POLD3/POT1/H3C8/PRIM1/AURKB/BLM/H4C3/RAD51
GO:2000738	positive regulation of stem cell differentiation	7/1707	18/18214	0.00079	0.02516	0.022007	NKX2-5/GATA4/TBX5/GATA6/PWP1/HOXB4/SOX5
GO:0000289	nuclear-transcribed mRNA poly(A) tail shortening	10/1707	35/18214	0.00105	0.03045	0.026635	TNKS1BP1/CNOT1/SAMD4A/TNRC6B/CNOT6L/CNOT2/TUT4/CNOT10/TOB1/PARN
GO:0051170	import into nucleus	28/1707	162/18214	0.00109	0.03136	0.027435	PRICKLE1/PIK3R1/UBR5/MAVS/SMAD3/TPR/RANBP2/FAM53B/MAPK14/NUP153/GLI3/NUP98/IPO8/NFKBIA/GEMIN7/FERMT1/NUP85/KPNA3/HEATR3/SUFU/NUP62/HIKESHI/BACH2/E2F3/SNUPN/GEMIN6/EFCAB7/TMCO6
GO:0016358	dendrite development	37/1707	235/18214	0.00119	0.03304	0.028903	EPHB3/RAPGEF2/GSK3B/DAB2IP/TANC2/PHACTR1/FYN/TNIK/IQSEC1/SRGAP2/FOXO6/MECP2/MEF2C/PPP1R9B/PTEN/STRN/KALRN/SIPA1L1/TAOK2/PAK4/ZMYND8/STK11/ARF6/HECW2/FBXO31/NEDD4L/BCL11A/CTNND2/RTN4IP1/PRKG1/PREX1/GRIP1/EFNA1/ARHGAP44/CRTC1/DTNBP1/DGKG

GO:0051348	negative regulation of transferase activity	44/1707	294/18214	0.00126	0.0342	0.02992	MYOCD/GSK3B/DAB2IP/MLLT1/RGS3/INKA2/TRIB1/CORO1C/DUSP7/NF2/RPTOR/APC/PIK3CB/PRKAG2/SPRY4/CHMP6/PDPK1/NF1/PTEN/PIP4K2A/TAOK3/LRP6/DUSP14/ABL1/NCK1/TRIB2/IBTK/SPRY2/GGNBP2/TRIM27/SPRED2/NUP62/GNAQ/SRC/DTX3L/POT1/SPRY1/DUSP19/DTNBP1/UBASH3B/CIT/ZFYVE28/TFAP4/DUSP18
GO:0090287	regulation of cellular response to growth factor stimulus	44/1707	294/18214	0.00126	0.0342	0.02992	MYOCD/GATA4/NUMA1/DAB2IP/BCL9L/SMAD3/SKI/CREBBP/CASK/SMAD6/LDLRAD4/JCAD/EP300/UBE2O/GDF2/PIK3CB/SMURF2/SPRY4/PPARA/PDPK1/PMEPA1/FZD4/GRB10/SMURF1/ARNT/FERMT1/STK11/ABL1/PELO/TRIM33/SPRY2/RBPJ/SPRED2/PRDM16/ZBTB7A/BMPER/TOB1/RNF165/CHST11/SPRY1/FGF2/DOK5/CCBE1/MIR1-1
GO:0003012	muscle system process	62/1707	452/18214	0.00147	0.03813	0.03336	MYOCD/NKX2.5/CHRM2/HAND2/GATA4/RCSD1/AKAP6/GJC1/HCN4/AKAP13/TRIM63/TBX20/KCNA5/PPARGC1A/CACNB2/PKP2/TNFRSF1A/SMAD3/AKAP9/LMOD2/FOXO1/KCNJ8/ROCK2/EDNRA/KCNJ12/PDE4B/PPARA/ARHGEF11/LMOD1/FOXO3/PARP2/GATA6/GTF2IRD1/ADORA1/ARHGAP42/DLG1/CACNA1D/KCNN2/NEDD4L/JARID2/KCNJ5/NOS1AP/GJA5/GATA5/HDAC4/LMOD3/PRKG1/MIR208A/KCNJ3/SCN2B/KCNE4/SCO2/DOCK4/KCND3/OXTR/CALCRL/MIR145/EDN2/CHRM3/SCN7A/MIR1-1/KCNE3
GO:0051098	regulation of binding	51/1707	356/18214	0.00147	0.03813	0.03336	MYOCD/HAND2/RAPGEF2/NSD1/GSK3B/SMAD3/SKI/MARK3/RARA/EP300/MARK2/ARHGEF7/RIPOR2/TCF7L2/H1-0/PPARA/IFIT1/WAPL/NFKBIA/GPSM1/PEX14/RSF1/TNKS/TTBK2/CTBP2/ABL1/EPB41/HMBOX1/LARP6/BCL3/BMP2/ZBTB7A/TICAM1/SRC/HDAC4/ERCC4/LDLRAP1/TRAF6/DTX3L/IFI16/ZFPM1/DTNBP1/UBASH3B/MDFI/AURKB/ZNF593/TRIM6/TFAP4/MIR27B/CDT1/ARHGAP28
GO:0000729	DNA double-strand break processing	8/1707	25/18214	0.00148	0.03813	0.03336	RAD50/EXD2/KAT5/SMARCAD1/NBN/BRCA1/WDR70/BLM
GO:0016197	endosomal transport	37/1707	238/18214	0.0015	0.0383	0.033508	TBC1D14/USP7/CORO1C/UBE2O/ZFYVE9/ANKRD50/EEA1/SNX9/VPS37B/GBF1/CHMP6/VPS26B/AP5S1/EHD4/CHMP7/SRL/UBAP1/ITSN2/REPS1/MVB12B/RIC1/ARF6/TRIM27/RBSN/ACAP2/RAB9A/AKAP5/RAB35/VPS37C/TMCC1/GRIP1/ARHGAP44/MAGEL2/BLOC1S2/BTBD8/RASSF9/VPS37D
GO:0006606	protein import into nucleus	25/1707	142/18214	0.0015	0.0383	0.033508	PRICKLE1/PIK3R1/UBR5/MAVS/SMAD3/TPR/RANBP2/FAM53B/MAPK14/NUP153/GLI3/NUP98/IPO8/NFKBIA/FERMT1/NUP85/KPNA3/HEATR3/SUFU/NUP62/HIKESHI/E2F3/SNUPN/EFCAB7/TMCO6
GO:0046321	positive regulation of fatty acid oxidation	6/1707	15/18214	0.0016	0.03976	0.034787	IRS1/PPARGC1A/PPARA/PPARD/CPT1A/ABCD2
GO:1902850	microtubule cytoskeleton organization involved in mitosis	25/1707	143/18214	0.00166	0.04053	0.035454	PAFAH1B1/TACC2/NUMA1/TPR/STAG2/STAG1/MECP2/DRG1/KIF2A/TNKS/TPX2/SMC3/SPRY2/NUP62/INTS13/CENPE/WRAP73/KIF4A/GPSM2/ZW10/NDC80/SPRY1/AURKB/NUF2/SPC25
GO:0016311	dephosphorylation	65/1707	482/18214	0.00176	0.04174	0.036515	CCDC8/SSH2/GSK3B/PPP1R3B/SMAD3/PLPP7/PHACTR1/DUSP7/PPP1R3C/ROCK2/BTRC/PPP2R2B/CTDSP1/PTPN9/CSRNP3/PPP1R12A/SSH1/PTPRK/PPP2R5E/MEF2C/SGPP2/PPP1R26/PPP1R9B/RRP1B/PPARGC1B/PTPN3/PPP2R2A/PTEN/MTMR3/PPME1/PPP2R3A/ADORA1/STK11/PLPP1/DUSP14/PTPN4/NCK1/DLG1/PRUNE1/PDP2/PFKFB4/PPP3CC/CTDSPL/PTPN14/CTTNBP2NL/PPP1R37/LRRC39/BMP2/PLPP3/MTMR12/SRC/PTPRE/ELFN1/PPM1H/PHLPP1/PPP1R35/PIP4P1/KNL1/DUSP19/UBASH3B/INPP5B/NANP/PXYLP1/ELL/DUSP18
GO:0043254	regulation of protein-containing complex assembly	60/1707	438/18214	0.00179	0.04234	0.037046	WASF2/SSH2/GSK3B/TBX20/NUMA1/DAB2IP/KANK1/AKAP9/BAK1/LMOD2/SMAD6/LDLRAD4/EP300/ARFGEF1/SSH1/CREB1/SNX9/TPPP/SPIRE1/CPTP/PSMC2/MECP2/MPP7/DRG1/STXBP6/PMEPA1/LMOD1/FCHSD2/ARFIP2/CARMIL1/FNIP2/FERMT1/FER/LMO4/RNF4/ABL1/NCK1/HRK/ARF6/DLG1/PREB/PRUNE1/TRIM27/NAV3/RASA1/PRKCZ/PRKCE/TICAM1/SRC/ERCC4/LMOD3/TRAF3IP1/TRAF2/RAB3A/PREX1/MAGEL2/ARHGAP18/LCAT/CDT1/ARHGAP28
GO:0002088	lens development in camera-type eye	16/1707	77/18214	0.00182	0.04247	0.037152	SMAD3/SKI/NF2/NECTIN1/WNT5B/CRYBG3/SHROOM2/MEIS1/DLG1/SPRY2/FZR1/CDK4/SPRED2/CTNS/SPRY1/FGF2
GO:0006023	aminoglycan biosynthetic process	21/1707	114/18214	0.00195	0.04441	0.038851	ST3GAL1/NDST1/GCNT2/EXT1/ST3GAL2/XYLT2/HS3ST3A1/CHST3/CHSY1/B3GNT9/B3GALT6/XYLT1/CHST11/B3GALT2/CHSY3/B3GNT8/HS3ST3B1/PXYLP1/UST/B3GALT1/B3GALT5
GO:2000392	regulation of lamellipodium morphogenesis	5/1707	11/18214	0.00205	0.04521	0.039551	KANK1/RREB1/CORO1C/ARHGEF7/SRC
GO:0043624	cellular protein complex disassembly	34/1707	219/18214	0.00233	0.0485	0.042427	TAOK1/ARHGEF2/LMOD2/MICAL3/MRPL15/APC/MRPL34/MRPL9/SH3GL1/PPP1R9B/LMOD1/TRIM54/CARMIL1/KIF2A/GSPT2/PEX14/TTBK2/MRPS18B/TPX2/JMJD4/MRPS26/MRPS31/MRPL45/NAV3/MRPS18A/MRPL17/MRPL27/LMOD3/MRPL22/MRPL14/KIF18B/N6AMT1/MRPL36/NCKAP5
GO:0099558	maintenance of synapse structure	6/1707	16/18214	0.00235	0.0485	0.042427	OPHN1/CTBP2/ARF6/DLG1/RAB3A/BSN

GO Terms_AmrvsDMSO_Upregulated							
ID	Description	GeneRatio	BgRatio	pvalue	p.adjust	qvalue	geneID
GO:0008625	extrinsic apoptotic signaling pathway via death domain receptors	5/20	84/18214	2.72E-08	1.936E-05	1.16E-05	TNFRSF10C/FAS/TNFRSF10A/FGB/FGA
GO:1902042	negative regulation of extrinsic apoptotic signaling pathway via death domain receptors	4/20	41/18214	1.04E-07	3.718E-05	2.22E-05	FAS/TNFRSF10A/FGB/FGA
GO:1902041	regulation of extrinsic apoptotic signaling pathway via death domain receptors	4/20	59/18214	4.63E-07	0.00011	6.58E-05	FAS/TNFRSF10A/FGB/FGA
GO:0035860	glial cell-derived neurotrophic factor receptor signaling pathway	2/20	10/18214	5.13E-05	0.0040622	0.002429	GDF15/SULF2
GO:0072332	intrinsic apoptotic signaling pathway by p53 class mediator	3/20	75/18214	7.27E-05	0.0048683	0.002911	CDKN1A/MDM2/EDA2R
GO:0006919	activation of cysteine-type endopeptidase activity involved in apoptotic process	3/20	87/18214	0.000113	0.0067229	0.00402	BBC3/FAS/TNFRSF10A
GO:0045926	negative regulation of growth	4/20	245/18214	0.000131	0.0069364	0.004147	CDKN1A/GDF15/MT1G/NTN1
GO:0034116	positive regulation of heterotypic cell-cell adhesion	2/20	16/18214	0.000136	0.0069364	0.004147	FGB/FGA
GO:0048640	negative regulation of developmental growth	3/20	107/18214	0.000209	0.0095273	0.005697	CDKN1A/GDF15/NTN1
GO:0055093	response to hyperoxia	2/20	20/18214	0.000215	0.0095273	0.005697	CDKN1A/FAS
GO:0031639	plasminogen activation	2/20	23/18214	0.000286	0.0104651	0.006257	FGB/FGA
GO:0071496	cellular response to external stimulus	4/20	301/18214	0.000287	0.0104651	0.006257	CDKN1A/ALB/FAS/TNFRSF10A
GO:0002576	platelet degranulation	3/20	126/18214	0.000338	0.0104651	0.006257	ALB/FGB/FGA
GO:0042730	fibrinolysis	2/20	25/18214	0.000338	0.0104651	0.006257	FGB/FGA
GO:0034114	regulation of heterotypic cell-cell adhesion	2/20	26/18214	0.000366	0.0104651	0.006257	FGB/FGA
GO:0036296	response to increased oxygen levels	2/20	27/18214	0.000396	0.0104651	0.006257	CDKN1A/FAS
GO:0071214	response to increased oxygen levels	4/20	330/18214	0.000408	0.0104651	0.006257	CDKN1A/MDM2/FAS/TNFRSF10A
GO:0104004	cellular response to environmental stimulus	4/20	330/18214	0.000408	0.0104651	0.006257	CDKN1A/MDM2/FAS/TNFRSF10A
GO:0071480	cellular response to gamma radiation	2/20	28/18214	0.000426	0.0104651	0.006257	CDKN1A/MDM2
GO:0072376	protein activation cascade	2/20	28/18214	0.000426	0.0104651	0.006257	FGB/FGA
GO:0072378	blood coagulation, fibrin clot formation	2/20	28/18214	0.000426	0.0104651	0.006257	FGB/FGA
GO:0045907	positive regulation of vasoconstriction	2/20	31/18214	0.000523	0.01242	0.007426	FGB/FGA
GO:0009267	cellular response to starvation	3/20	155/18214	0.00062	0.0131952	0.00789	CDKN1A/ALB/FAS
GO:2000352	negative regulation of endothelial cell apoptotic process	2/20	34/18214	0.000629	0.0131952	0.00789	FGB/FGA
GO:1900026	positive regulation of substrate adhesion-dependent cell spreading	2/20	41/18214	0.000915	0.0186499	0.011151	FGB/FGA
GO:0042594	response to starvation	3/20	194/18214	0.001187	0.0216563	0.012949	CDKN1A/ALB/FAS
GO:1990928	response to amino acid starvation	2/20	48/18214	0.001254	0.0216563	0.012949	CDKN1A/FAS
GO:0031667	response to nutrient levels	4/20	447/18214	0.001269	0.0216563	0.012949	CDKN1A/GDF15/ALB/FAS
GO:1900047	negative regulation of hemostasis	2/20	49/18214	0.001306	0.0216563	0.012949	FGB/FGA
GO:0043281	regulation of cysteine-type endopeptidase activity involved in apoptotic process	3/20	205/18214	0.001391	0.0220343	0.013175	BBC3/FAS/TNFRSF10A
GO:0031669	cellular response to nutrient levels	3/20	208/18214	0.00145	0.0222964	0.013331	CDKN1A/ALB/FAS

GO:0050819	negative regulation of coagulation	2/20	52/18214	0.00147	0.0222964	0.013331	FGB/FGA
GO:0010332	response to gamma radiation	2/20	53/18214	0.001526	0.0224947	0.01345	CDKN1A/MDM2
GO:0006977	DNA damage response, signal transduction by p53 class mediator resulting in cell cycle arre:	2/20	56/18214	0.001702	0.0224947	0.01345	CDKN1A/MDM2
GO:0019731	antibacterial humoral response	2/20	57/18214	0.001763	0.0224947	0.01345	FGB/FGA
GO:0072431	signal transduction involved in mitotic G1 DNA damage checkpoint	2/20	57/18214	0.001763	0.0224947	0.01345	CDKN1A/MDM2
GO:1900024	regulation of substrate adhesion-dependent cell spreading	2/20	57/18214	0.001763	0.0224947	0.01345	FGB/FGA
GO:1902400	intracellular signal transduction involved in G1 DNA damage checkpoint	2/20	57/18214	0.001763	0.0224947	0.01345	CDKN1A/MDM2
GO:2000351	regulation of endothelial cell apoptotic process	2/20	58/18214	0.001825	0.0224947	0.01345	FGB/FGA
GO:1902402	signal transduction involved in mitotic DNA damage checkpoint	2/20	59/18214	0.001888	0.0224947	0.01345	CDKN1A/MDM2
GO:1902403	signal transduction involved in mitotic DNA integrity checkpoint	2/20	59/18214	0.001888	0.0224947	0.01345	CDKN1A/MDM2
GO:0034113	heterotypic cell-cell adhesion	2/20	60/18214	0.001952	0.0224947	0.01345	FGB/FGA
GO:0072413	signal transduction involved in mitotic cell cycle checkpoint	2/20	61/18214	0.002016	0.0224947	0.01345	CDKN1A/MDM2
GO:0009636	response to toxic substance	3/20	236/18214	0.00208	0.0224947	0.01345	CDKN1A/MT1G/ALB
GO:0031571	mitotic G1 DNA damage checkpoint	2/20	62/18214	0.002082	0.0224947	0.01345	CDKN1A/MDM2
GO:0070527	platelet aggregation	2/20	62/18214	0.002082	0.0224947	0.01345	FGB/FGA
GO:0044783	G1 DNA damage checkpoint	2/20	63/18214	0.002149	0.0225341	0.013474	CDKN1A/MDM2
GO:0072577	endothelial cell apoptotic process	2/20	64/18214	0.002217	0.0229088	0.013698	FGB/FGA
GO:0030193	regulation of blood coagulation	2/20	66/18214	0.002356	0.023657	0.014145	FGB/FGA
GO:0050818	regulation of coagulation	2/20	71/18214	0.00272	0.0265685	0.015886	FGB/FGA
GO:0031100	animal organ regeneration	2/20	72/18214	0.002796	0.0266028	0.015906	CDKN1A/SULF2
GO:0072331	signal transduction by p53 class mediator	3/20	263/18214	0.002829	0.0266028	0.015906	CDKN1A/MDM2/EDA2R
GO:0072401	signal transduction involved in DNA integrity checkpoint	2/20	73/18214	0.002873	0.0266028	0.015906	CDKN1A/MDM2
GO:0072422	signal transduction involved in DNA damage checkpoint	2/20	73/18214	0.002873	0.0266028	0.015906	CDKN1A/MDM2
GO:0061045	negative regulation of wound healing	2/20	74/18214	0.002951	0.026632	0.015924	FGB/FGA
GO:0071260	cellular response to mechanical stimulus	2/20	74/18214	0.002951	0.026632	0.015924	FAS/TNFRSF10A
GO:0010770	positive regulation of cell morphogenesis involved in differentiation	2/20	79/18214	0.003355	0.0291714	0.017442	FGB/FGA
GO:0042310	vasoconstriction	2/20	79/18214	0.003355	0.0291714	0.017442	FGB/FGA
GO:0071158	positive regulation of cell cycle arrest	2/20	82/18214	0.003609	0.0310038	0.018538	CDKN1A/MDM2
GO:0010720	positive regulation of cell development	3/20	291/18214	0.003762	0.0319309	0.019092	FGB/FGA/NTN1
GO:0034109	homotypic cell-cell adhesion	2/20	85/18214	0.003872	0.0321024	0.019195	FGB/FGA
GO:0045921	positive regulation of exocytosis	2/20	85/18214	0.003872	0.0321024	0.019195	FGB/FGA
GO:1903035	negative regulation of response to wounding	2/20	90/18214	0.00433	0.0354834	0.021216	FGB/FGA
GO:0051222	positive regulation of protein transport	3/20	310/18214	0.004491	0.0363847	0.021755	BBC3/FGB/FGA

GO:0010769	regulation of cell morphogenesis involved in differentiation	2/20	95/18214	0.004811	0.0373668	0.022342	FGB/FGA
GO:0090277	positive regulation of peptide hormone secretion	2/20	95/18214	0.004811	0.0373668	0.022342	FGB/FGA
GO:0048638	regulation of developmental growth	3/20	318/18214	0.004822	0.0373668	0.022342	CDKN1A/GDF15/NTN1
GO:1904951	positive regulation of establishment of protein localization	3/20	326/18214	0.005167	0.0396127	0.023685	BBC3/FGB/FGA
GO:0044773	mitotic DNA damage checkpoint	2/20	102/18214	0.005525	0.0419068	0.025057	CDKN1A/MDM2
GO:0030330	DNA damage response, signal transduction by p53 class mediator	2/20	106/18214	0.005953	0.0442155	0.026437	CDKN1A/MDM2
GO:0044774	mitotic DNA integrity checkpoint	2/20	106/18214	0.005953	0.0442155	0.026437	CDKN1A/MDM2
GO:0034446	substrate adhesion-dependent cell spreading	2/20	108/18214	0.006173	0.0446006	0.026668	FGB/FGA
GO:0010038	response to metal ion	3/20	348/18214	0.006193	0.0446006	0.026668	MT1G/FGB/FGA
GO:0022612	gland morphogenesis	2/20	111/18214	0.00651	0.0464132	0.027751	SULF2/NTN1

GO Terms_AMRvsDMSO_Downregulated							
ID	Description	GeneRatio	BgRatio	pvalue	p.adjust	qvalue	geneID
GO:0007059	chromosome segregation	66/205	329/18214	6.4E-65	1.2E-61	9.48E-62	MKI67/CENPF/PRC1/BUB1/BIRC5/BUB1B/CDC6/KNL1/TOP2A/NUSAP1/SPAG5/CCNB1/KIF4A/KIF23/KIF2C/CDC20/PLK1/KIFC1/CENPE/CDCA8/KIF14/NDC80/UBE2C/NCAPG/RACGAP1/KIF18B/HJURP/NCAPH/AURKB/ZWINT/TTK/SKA1/TRIP13/NEK2/CDT1/CDCA5/FANCD2/FEN1/CDCA2/DLGAP5/RMI2/NUF2/BRCA1/FAM83D/NCAPG2/ESPL1/KIF22/BRIP1/SPC25/DSN1/PTTG1/SKA3/ESCO2/KIF18A/SGO1/FBXO5/CENPH/CCNE2/MAD2L1/PSRC1/OIP5/HASPIN/CENPK/EME1/DSCC1/CENPW
GO:0000280	nuclear division	67/205	432/18214	4.6E-58	4.2E-55	3.45E-55	MKI67/CENPF/ANLN/TPX2/PRC1/BUB1/BUB1B/CDC6/TOP2A/NUSAP1/SPAG5/CCNB1/KIF4A/KIF23/KIF2C/MYBL2/CDC20/PLK1/KIFC1/CENPE/CDCA8/KIF14/NDC80/UBE2C/NCAPG/RACGAP1/KIF18B/KIF11/CDK1/CCNB2/NCAPH/AURKB/ZWINT/TTK/TRIP13/FANCA/NEK2/CDT1/CDCA5/FANCD2/DLGAP5/NUF2/RAD51/NCAPG2/ESPL1/KIF22/BRIP1/PKMYT1/KIF20B/BRCA2/DSN1/PTTG1/CHEK1/KIF18A/RAD51AP1/SGO1/FBXO5/CCNE2/MAD2L1/PSRC1/RAD54L/HASPIN/CENPK/EME1/DSCC1/MND1/CDC25C
GO:0098813	nuclear chromosome segregation	55/205	266/18214	2E-54	9.1E-52	7.36E-52	CENPF/PRC1/BUB1/BUB1B/CDC6/KNL1/TOP2A/NUSAP1/SPAG5/CCNB1/KIF4A/KIF23/KIF2C/CDC20/PLK1/KIFC1/CENPE/CDCA8/KIF14/NDC80/UBE2C/NCAPG/RACGAP1/KIF18B/NCAPH/AURKB/ZWINT/TTK/TRIP13/NEK2/CDT1/CDCA5/FANCD2/FEN1/DLGAP5/RMI2/NUF2/FAM83D/NCAPG2/ESPL1/KIF22/BRIP1/DSN1/PTTG1/ESCO2/KIF18A/SGO1/FBXO5/CCNE2/MAD2L1/PSRC1/HASPIN/CENPK/EME1/DSCC1
GO:0140014	mitotic nuclear division	56/205	296/18214	3.8E-53	1.4E-50	1.14E-50	MKI67/CENPF/ANLN/TPX2/PRC1/BUB1/BUB1B/CDC6/NUSAP1/SPAG5/CCNB1/KIF4A/KIF23/KIF2C/MYBL2/CDC20/PLK1/KIFC1/CENPE/CDCA8/KIF14/NDC80/UBE2C/NCAPG/RACGAP1/KIF18B/KIF11/CDK1/CCNB2/NCAPH/AURKB/ZWINT/TTK/TRIP13/NEK2/CDT1/CDCA5/DLGAP5/NUF2/NCAPG2/ESPL1/KIF22/PKMYT1/KIF20B/DSN1/PTTG1/CHEK1/KIF18A/SGO1/FBXO5/MAD2L1/PSRC1/HASPIN/CENPK/DSCC1/CDC25C
GO:0000819	sister chromatid segregation	49/205	194/18214	5.4E-53	1.6E-50	1.33E-50	CENPF/PRC1/BUB1/BUB1B/CDC6/TOP2A/NUSAP1/SPAG5/CCNB1/KIF4A/KIF23/KIF2C/CDC20/PLK1/KIFC1/CENPE/CDCA8/KIF14/NDC80/UBE2C/NCAPG/RACGAP1/KIF18B/NCAPH/AURKB/ZWINT/TTK/TRIP13/NEK2/CDT1/CDCA5/FEN1/DLGAP5/RMI2/NUF2/NCAPG2/ESPL1/KIF22/DSN1/PTTG1/ESCO2/KIF18A/SGO1/FBXO5/MAD2L1/PSRC1/HASPIN/CENPK/DSCC1
GO:0000070	mitotic sister chromatid segregation	45/205	164/18214	2E-50	5.4E-48	4.36E-48	CENPF/PRC1/BUB1/BUB1B/CDC6/NUSAP1/SPAG5/CCNB1/KIF4A/KIF23/KIF2C/CDC20/PLK1/KIFC1/CENPE/CDCA8/KIF14/NDC80/UBE2C/NCAPG/RACGAP1/KIF18B/NCAPH/AURKB/ZWINT/TTK/TRIP13/NEK2/CDT1/CDCA5/DLGAP5/NUF2/NCAPG2/ESPL1/KIF22/DSN1/PTTG1/KIF18A/SGO1/FBXO5/MAD2L1/PSRC1/HASPIN/CENPK/DSCC1
GO:0071103	DNA conformation change	46/205	347/18214	4.3E-36	9.9E-34	8.07E-34	H3C2/MCM6/H1-5/MCM5/H3C8/MCM2/MCM7/KNL1/TOP2A/NUSAP1/CCNB1/NCAPG/HJURP/CHAF1A/POLQ/H2BC17/H3C7/NCAPH/H2BC9/H4C4/H2BC11/CDCA5/RAD51/NCAPG2/ERCC6L/H3C12/BRIP1/BLM/H4C9/ASF1B/CENPU/CENPA/H2BC10/CHAF1B/HELLS/CENPM/CENPI/CENPH/GPER1/H2BC13/RAD54L/OIP5/CENPK/SUV39H1/DSCC1/CENPW
GO:0006260	DNA replication	39/205	280/18214	2.1E-31	4.3E-29	3.49E-29	MCM6/CLSPN/DTL/MCM5/CDC6/MCM2/MCM7/CCNA2/FAM111B/CDK1/RRM2/MCM10/CHAF1A/POLQ/GINS2/TICRR/EXO1/CDT1/CDC45/FEN1/RMI2/BRCA1/RAD51/BRIP1/BLM/PCLAF/BRCA2/CHAF1B/GINS1/GINS4/ESCO2/CHEK1/ORC1/FBXO5/CCNE2/EME1/DSCC1/POLE2/TRAIP
GO:0065004	protein-DNA complex assembly	36/205	238/18214	2.6E-30	4.4E-28	3.55E-28	CENPF/H3C2/MCM6/H1.5/MCM5/H3C8/MCM2/MCM7/KNL1/CENPE/HJURP/CHAF1A/H2BC17/H3C7/H2BC9/H4C4/H2BC11/CDT1/CDC45/DLGAP5/RAD51/H3C12/H4C9/ASF1B/CENPU/CENPA/H2BC10/CHAF1B/HELLS/CENPM/CENPI/CENPH/H2BC13/OIP5/CENPK/CENPW
GO:0071824	protein-DNA complex subunit organization	36/205	278/18214	7.4E-28	1.1E-25	9.22E-26	CENPF/H3C2/MCM6/H1-5/MCM5/H3C8/MCM2/MCM7/KNL1/CENPE/HJURP/CHAF1A/H2BC17/H3C7/H2BC9/H4C4/H2BC11/CDT1/CDC45/DLGAP5/RAD51/H3C12/H4C9/ASF1B/CENPU/CENPA/H2BC10/CHAF1B/HELLS/CENPM/CENPI/CENPH/H2BC13/OIP5/CENPK/CENPW
GO:1902850	microtubule cytoskeleton organization involved in mitosis	27/205	143/18214	1.4E-25	1.9E-23	1.58E-23	TPX2/PRC1/NUSAP1/CCNB1/KIF4A/KIF23/MYBL2/CDC20/PLK1/KIFC1/CENPE/NDC80/RACGAP1/KIF11/AURKB/TTK/NEK2/DLGAP5/NUF2/ESPL1/CENPA/SAPCD2/SPC25/CENPH/MAD2L1/PSRC1/STIL
GO:0051983	regulation of chromosome segregation	23/205	87/18214	1.5E-25	1.9E-23	1.58E-23	MKI67/CENPF/BUB1/BUB1B/CDC6/CCNB1/KIF2C/CDC20/PLK1/CENPE/NDC80/UBE2C/AURKB/ZWINT/TTK/TRIP13/CDT1/DLGAP5/RMI2/ESPL1/PTTG1/FBXO5/MAD2L1
GO:0033045	regulation of sister chromatid segregation	21/205	71/18214	1.4E-24	1.7E-22	1.41E-22	CENPF/BUB1/BUB1B/CDC6/CCNB1/CDC20/PLK1/CENPE/NDC80/UBE2C/AURKB/ZWINT/TTK/TRIP13/CDT1/DLGAP5/RMI2/ESPL1/PTTG1/FBXO5/MAD2L1
GO:0006334	nucleosome assembly	26/205	143/18214	3.3E-24	3.8E-22	3.09E-22	H3C2/H1.5/H3C8/MCM2/KNL1/HJURP/CHAF1A/H2BC17/H3C7/H2BC9/H4C4/H2BC11/H3C12/H4C9/ASF1B/CENPU/CENPA/H2BC10/CHAF1B/CENPM/CENPI/CENPH/H2BC13/OIP5/CENPK/CENPW

GO:0010965	regulation of mitotic sister chromatid separation	20/205	64/18214	5.3E-24	5.7E-22	4.63E-22	CENPF/BUB1/BUB1B/CDC6/CCNB1/CDC20/PLK1/CENPE/NDC80/UBE2C/AURKB/ZWINT/TTK/TRIP13/CDT1/DLGAP5/ESPL1/PTTG1/FBXO5/MAD2L1
GO:0000075	cell cycle checkpoint	29/205	208/18214	1.8E-23	1.7E-21	1.4E-21	CENPF/CLSPN/BUB1/DTL/BUB1B/CDC6/CCNB1/GTSE1/CDC20/PLK1/NDC80/CDK1/AURKB/WDR76/ZWINT/TTK/TICRR/TRIP13/CDT1/CDC45/FANCD2/BRCA1/BRIP1/BLM/CHEK1/ORC1/MAD2L1/EME1/CDC25C
GO:0031497	chromatin assembly	28/205	193/18214	3.5E-23	3.2E-21	2.62E-21	H3C2/H1.5/H3C8/MCM2/KNL1/HJURP/CHAF1A/H2BC17/H3C7/H2BC9/H4C4/H2BC11/H3C12/H4C9/ASF1B/CENPU/CENPA/H2BC10/CHAF1B/HELLS/CENPM/CENPI/CENPH/H2BC13/OIP5/CENPK/SUV39H1/CENPW
GO:0007088	regulation of mitotic nuclear division	23/205	109/18214	4.3E-23	3.4E-21	2.79E-21	MKI67/CENPF/BUB1/BUB1B/NUSAP1/CCNB1/CDC20/PLK1/NDC80/AURKB/ZWINT/TTK/TRIP13/NEK2/CDT1/DLGAP5/ESPL1/PKMYT1/KIF20B/CHEK1/FBXO5/MAD2L1/CDC25C
GO:0051304	chromosome separation	22/205	95/18214	4.3E-23	3.4E-21	2.79E-21	CENPF/BUB1/BUB1B/CDC6/TOP2A/CCNB1/CDC20/PLK1/CENPE/NDC80/UBE2C/AURKB/ZWINT/TTK/TRIP13/CDT1/DLGAP5/ESPL1/PTTG1/FBXO5/MAD2L1/EME1
GO:1905818	regulation of chromosome separation	20/205	71/18214	5.7E-23	4.3E-21	3.52E-21	CENPF/BUB1/BUB1B/CDC6/CCNB1/CDC20/PLK1/CENPE/NDC80/UBE2C/AURKB/ZWINT/TTK/TRIP13/CDT1/DLGAP5/ESPL1/PTTG1/FBXO5/MAD2L1
GO:0007051	spindle organization	27/205	181/18214	1E-22	7.3E-21	5.93E-21	TPX2/PRC1/ASPM/SPAG5/CCNB1/KIF4A/KIF23/MYBL2/CDC20/PLK1/KIFC1/CENPE/NDC80/RACGAP1/KIF11/CCNB2/AURKB/TTK/NEK2/DLGAP5/NUF2/ESPL1/SPC25/FBXO5/CENPH/PSRC1/STIL
GO:1901987	regulation of cell cycle phase transition	39/205	476/18214	1.1E-22	7.4E-21	6.05E-21	CENPF/ANLN/TPX2/CLSPN/BUB1/DTL/BUB1B/CDC6/CCNB1/GTSE1/CDC20/PLK1/CENPE/KIF14/NDC80/UBE2C/CDK1/SUSD2/HMMR/AURKB/ZWINT/TTK/TICRR/CDKN2C/TRIP13/NEK2/CDT1/CDCA5/PLK4/DLGAP5/BRCA1/FAM83D/ESPL1/BLM/CHEK1/ORC1/FBXO5/MAD2L1/CDC25C
GO:0051783	regulation of nuclear division	24/205	134/18214	3E-22	1.8E-20	1.49E-20	MKI67/CENPF/BUB1/BUB1B/NUSAP1/CCNB1/CDC20/PLK1/NDC80/AURKB/ZWINT/TTK/TRIP13/NEK2/CDT1/DLGAP5/ESPL1/PKMYT1/KIF20B/CHEK1/RAD51AP1/FBXO5/MAD2L1/CDC25C
GO:1901990	regulation of mitotic cell cycle phase transition	37/205	437/18214	5.1E-22	3E-20	2.44E-20	CENPF/ANLN/TPX2/CLSPN/BUB1/DTL/BUB1B/CDC6/CCNB1/GTSE1/CDC20/PLK1/CENPE/KIF14/NDC80/UBE2C/CDK1/HMMR/AURKB/ZWINT/TTK/TICRR/CDKN2C/TRIP13/NEK2/CDT1/CDCA5/PLK4/DLGAP5/BRCA1/ESPL1/BLM/CHEK1/ORC1/FBXO5/MAD2L1/CDC25C
GO:0007093	mitotic cell cycle checkpoint	25/205	158/18214	9.5E-22	5.5E-20	4.45E-20	CENPF/CLSPN/BUB1/BUB1B/CDC6/CCNB1/GTSE1/CDC20/PLK1/NDC80/CDK1/AURKB/ZWINT/TTK/TICRR/TRIP13/CDT1/FANCD2/BRCA1/BLM/CHEK1/ORC1/MAD2L1/EME1/CDC25C
GO:0006333	chromatin assembly or disassembly	28/205	218/18214	1.1E-21	6E-20	4.86E-20	H3C2/H1.5/H3C8/MCM2/KNL1/HJURP/CHAF1A/H2BC17/H3C7/H2BC9/H4C4/H2BC11/H3C12/H4C9/ASF1B/CENPU/CENPA/H2BC10/CHAF1B/HELLS/CENPM/CENPI/CENPH/H2BC13/OIP5/CENPK/SUV39H1/CENPW
GO:0034728	nucleosome organization	26/205	181/18214	1.8E-21	9.7E-20	7.9E-20	H3C2/H1.5/H3C8/MCM2/KNL1/HJURP/CHAF1A/H2BC17/H3C7/H2BC9/H4C4/H2BC11/H3C12/H4C9/ASF1B/CENPU/CENPA/H2BC10/CHAF1B/CENPM/CENPI/CENPH/H2BC13/OIP5/CENPK/CENPW
GO:0051321	meiotic cell cycle	29/205	246/18214	2.2E-21	1.2E-19	9.55E-20	BUB1/BUB1B/TOP2A/CDC20/PLK1/CCNB2/TTK/TRIP13/FANCA/NEK2/EXO1/FANCD2/NUF2/XRCC2/RAD51/ESPL1/BRIP1/PKMYT1/BRCA2/PTTG1/KIF18A/RAD51AP1/SGO1/FBXO5/CCNE2/RAD54L/EME1/MND1/CDC25C
GO:0010948	negative regulation of cell cycle process	33/205	357/18214	7.1E-21	3.5E-19	2.88E-19	CENPF/CLSPN/BUB1/DTL/BUB1B/CDC6/CCNB1/GTSE1/CDC20/PLK1/NDC80/CDK1/RRM2/SUSD2/AURKB/ZWINT/TTK/TICRR/CDKN2C/TRIP13/NEK2/CDT1/BRCA1/RAD51/ESPL1/BLM/PTTG1/CHEK1/ORC1/FBXO5/GPER1/MAD2L1/CDC25C
GO:0140013	meiotic nuclear division	24/205	170/18214	1E-19	4.8E-18	3.89E-18	BUB1/BUB1B/TOP2A/CDC20/PLK1/CCNB2/TTK/TRIP13/FANCA/FANCD2/NUF2/RAD51/ESPL1/BRIP1/BRCA2/PTTG1/KIF18A/RAD51AP1/SGO1/FBXO5/CCNE2/RAD54L/EME1/MND1
GO:0006302	double-strand break repair	28/205	266/18214	2.5E-19	1.1E-17	8.99E-18	FOXM1/MCM6/MCM5/MCM2/MCM7/POLQ/GINS2/TRIP13/H4C4/CDC45/CDCA5/FANCD2/FEN1/RMI2/BRCA1/XRCC2/RAD51/BRIP1/BLM/H4C9/BRCA2/GINS4/ESCO2/CHEK1/RAD51AP1/RAD54L/EME1/PARPBP
GO:0033046	negative regulation of sister	15/205	42/18214	2.7E-19	1.1E-17	8.99E-18	CENPF/BUB1/BUB1B/CCNB1/CDC20/PLK1/NDC80/AURKB/ZWINT/TTK/TRIP13/CDT1/PTTG1/FBXO5/MAD2L1
GO:2000816	negative regulation of mitotic sister chromatid	15/205	42/18214	2.7E-19	1.1E-17	8.99E-18	CENPF/BUB1/BUB1B/CCNB1/CDC20/PLK1/NDC80/AURKB/ZWINT/TTK/TRIP13/CDT1/PTTG1/FBXO5/MAD2L1
GO:0051310	metaphase plate congression	17/205	64/18214	3.3E-19	1.3E-17	1.1E-17	CENPF/SPAG5/CCNB1/KIF2C/KIFC1/CENPE/CDCA8/KIF14/NDC80/AURKB/CDT1/CDCA5/NUF2/FAM83D/KIF22/KIF18A/PSRC1
GO:0051303	establishment of	18/205	78/18214	4.6E-19	1.8E-17	1.5E-17	CENPF/SPAG5/CCNB1/KIF2C/KIFC1/CENPE/CDCA8/KIF14/NDC80/AURKB/CDT1/CDCA5/DLGAP5/NUF2/FAM83D/KIF22/KIF18A/PSRC1
GO:0033044	regulation of chromosome	28/205	273/18214	5.1E-19	2E-17	1.6E-17	CENPF/BUB1/H1-
GO:0051985	negative regulation of	15/205	44/18214	6.1E-19	2.3E-17	1.84E-17	CENPF/BUB1/BUB1B/CCNB1/CDC20/PLK1/NDC80/AURKB/ZWINT/TTK/TRIP13/CDT1/PTTG1/FBXO5/MAD2L1
GO:1905819	negative regulation of	15/205	44/18214	6.1E-19	2.3E-17	1.84E-17	CENPF/BUB1/BUB1B/CCNB1/CDC20/PLK1/NDC80/AURKB/ZWINT/TTK/TRIP13/CDT1/PTTG1/FBXO5/MAD2L1
GO:0050000	chromosome localization	18/205	80/18214	7.6E-19	2.8E-17	2.27E-17	CENPF/SPAG5/CCNB1/KIF2C/KIFC1/CENPE/CDCA8/KIF14/NDC80/AURKB/CDT1/CDCA5/DLGAP5/NUF2/FAM83D/KIF22/KIF18A/PSRC1
GO:0034508	centromere complex assembly	16/205	56/18214	1E-18	3.6E-17	2.95E-17	CENPF/KNL1/CENPE/HJURP/H4C4/DLGAP5/H4C9/CENPU/CENPA/HELLS/CENPM/CENPI/CENPH/OIP5/CENPK/CENPW

GO:0045787	positive regulation of cell	32/205	393/18214	1.3E-18	4.5E-17	3.69E-17	DTL/CDC6/NUSAP1/SPAG5/CCNB1/GTSE1/KIF23/CIT/KIF14/NDC80/UBE2C/RACGAP1/CDK1/AURKB/CDT1/CDCA5/FEN1/PLK4/DLGAP5/BRCA1/F
GO:0045839	negative regulation of	15/205	47/18214	1.9E-18	6.5E-17	5.31E-17	CENPF/BUB1/BUB1B/CCNB1/CDC20/PLK1/NDC80/AURKB/ZWINT/TTK/TRIP13/CDT1/CHEK1/FBXO5/MAD2L1
GO:0045841	negative regulation of	14/205	39/18214	3.9E-18	1.3E-16	1.05E-16	CENPF/BUB1/BUB1B/CCNB1/CDC20/PLK1/NDC80/AURKB/ZWINT/TTK/TRIP13/CDT1/FBXO5/MAD2L1
GO:0044839	cell cycle G2/M phase transition	27/205	273/18214	5.8E-18	1.9E-16	1.51E-16	CENPF/TPX2/FOXM1/CLSPN/DTL/CDC6/CCNA2/CCNB1/GTSE1/PLK1/KIF14/CDK1/CCNB2/HMMR/AURKB/TICRR/NEK2/PLK4/BRCA1/MELK/BLM/ PKMYT1/FBXL22/CHEK1/ORC1/FBXO5/CDC25C
GO:2001251	negative regulation of chromosome organization	18/205	91/18214	9.1E-18	2.8E-16	2.3E-16	CENPF/BUB1/BUB1B/MCM2/TOP2A/CCNB1/CDC20/PLK1/NDC80/AURKB/ZWINT/TTK/TRIP13/CDT1/ESPL1/PTTG1/FBXO5/MAD2L1
GO:0000086	G2/M transition of mitotic	26/205	255/18214	1.2E-17	3.5E-16	2.88E-16	CENPF/TPX2/FOXM1/CLSPN/DTL/CDC6/CCNA2/CCNB1/PLK1/KIF14/CDK1/CCNB2/HMMR/AURKB/TICRR/NEK2/PLK4/BRCA1/MELK/BLM/PKMYT1
GO:0051784	negative regulation of	15/205	54/18214	2.1E-17	6.2E-16	5.06E-16	CENPF/BUB1/BUB1B/CCNB1/CDC20/PLK1/NDC80/AURKB/ZWINT/TTK/TRIP13/CDT1/CHEK1/FBXO5/MAD2L1
GO:0006310	DNA recombination	27/205	290/18214	2.7E-17	8E-16	6.53E-16	MCM6/H1-
GO:0045930	negative regulation of	28/205	320/18214	3.4E-17	1E-15	8.11E-16	CENPF/CLSPN/BUB1/BUB1B/CDC6/CCNB1/GTSE1/CDC20/PLK1/NDC80/CDK1/AURKB/ZWINT/TTK/TICRR/CDKN2C/TRIP13/CDT1/FANCD2/BRCA1
GO:1901991	negative regulation of	25/205	249/18214	7.4E-17	2.1E-15	1.72E-15	CENPF/CLSPN/BUB1/BUB1B/CDC6/CCNB1/GTSE1/CDC20/PLK1/NDC80/CDK1/AURKB/ZWINT/TTK/TICRR/CDKN2C/TRIP13/CDT1/BRCA1/BLM/CH
GO:0090068	positive regulation of cell cycle process	27/205	302/18214	7.6E-17	2.1E-15	1.75E-15	DTL/CDC6/NUSAP1/SPAG5/CCNB1/GTSE1/KIF23/CIT/KIF14/NDC80/UBE2C/RACGAP1/CDK1/AURKB/CDT1/CDCA5/FEN1/PLK4/DLGAP5/BRCA1/F AM83D/ESPL1/KIF20B/RAD51AP1/FBXO5/MAD2L1/CDC25C
GO:0007080	mitotic metaphase plate	14/205	50/18214	2.2E-16	5.8E-15	4.68E-15	CCNB1/KIF2C/KIFC1/CENPE/CDCA8/KIF14/NDC80/AURKB/CDT1/CDCA5/NUF2/KIF22/KIF18A/PSRC1
GO:0031055	chromatin remodeling at centromere	13/205	47/18214	3.2E-15	7.8E-14	6.34E-14	KNL1/HJURP/H4C4/H4C9/CENPU/CENPA/HELLS/CENPM/CENPI/CENPH/OIP5/CENPK/CENPW
GO:0044843	cell cycle G1/S phase	25/205	297/18214	4.7E-15	1.1E-13	9.18E-14	MCM6/MCM5/CDC6/MCM2/MCM7/CCNA2/IQGAP3/CCNB1/GTSE1/KIF14/CDK1/RRM2/MCM10/SUSD2/CDKN2C/CDT1/CDC45/CDK18/FAM83D
GO:0006270	DNA replication initiation	12/205	40/18214	1.3E-14	3E-13	2.45E-13	MCM6/MCM5/CDC6/MCM2/MCM7/MCM10/TICRR/CDT1/CDC45/ORC1/CCNE2/POLE2
GO:0051383	kinetochore organization	10/205	22/18214	1.5E-14	3.5E-13	2.84E-13	CENPF/CENPE/NDC80/CDT1/DLGAP5/NUF2/CENPA/CENPH/CENPK/CENPW
GO:0034080	CENP-A containing nucleosome assembly	12/205	43/18214	3.4E-14	7.4E-13	6E-13	KNL1/HJURP/H4C4/H4C9/CENPU/CENPA/CENPM/CENPI/CENPH/OIP5/CENPK/CENPW
GO:0061641	CENP-A containing chromatin organization	12/205	43/18214	3.4E-14	7.4E-13	6E-13	KNL1/HJURP/H4C4/H4C9/CENPU/CENPA/CENPM/CENPI/CENPH/OIP5/CENPK/CENPW
GO:0000910	cytokinesis	19/205	171/18214	7.1E-14	1.5E-12	1.23E-12	ANLN/KIF20A/PRC1/CDC6/NUSAP1/KIF4A/KIF23/PLK1/CIT/CEP55/KIF14/RACGAP1/KLHL13/AURKB/PLK4/ESPL1/CENPA/KIF20B/BRCA2
GO:0000082	G1/S transition of mitotic cell cycle	22/205	274/18214	5.9E-13	1.2E-11	9.95E-12	MCM6/MCM5/CDC6/MCM2/MCM7/IQGAP3/CCNB1/GTSE1/KIF14/CDK1/RRM2/MCM10/CDKN2C/CDT1/CDC45/CDK18/ORC1/FBXO5/CCNE2/CD KN3/POLE2/CDC25C
GO:0034502	protein localization to	14/205	91/18214	1.7E-12	3.4E-11	2.74E-11	H1-5/BUB1B/KNL1/PLK1/NDC80/CDK1/AURKB/TTK/CDT1/CDCA5/CENPA/BRCA2/ESCO2/HASPIN
GO:0043486	histone exchange	12/205	58/18214	1.7E-12	3.4E-11	2.75E-11	KNL1/HJURP/H4C4/H4C9/CENPU/CENPA/CENPM/CENPI/CENPH/OIP5/CENPK/CENPW
GO:0051225	spindle assembly	15/205	113/18214	2.4E-12	4.8E-11	3.88E-11	TPX2/PRC1/SPAG5/KIF4A/KIF23/MYBL2/CDC20/PLK1/KIFC1/RACGAP1/KIF11/CCNB2/AURKB/NEK2/FBXO5
GO:0090307	mitotic spindle assembly	12/205	65/18214	7.1E-12	1.4E-10	1.11E-10	TPX2/PRC1/KIF4A/KIF23/MYBL2/CDC20/PLK1/KIFC1/RACGAP1/KIF11/AURKB/NEK2
GO:0034401	chromatin organization involved in regulation of	16/205	152/18214	1.6E-11	3.1E-10	2.51E-10	H3C2/H1-5/H3C8/H2AC11/H3C7/H4C4/CDC45/H2AC13/H2AC12/H3C12/H4C9/H2AC4/HELLS/CHEK1/SUV39H1/H2AC16
GO:0000281	mitotic cytokinesis	12/205	70/18214	1.8E-11	3.3E-10	2.71E-10	ANLN/KIF20A/NUSAP1/KIF4A/KIF23/PLK1/CIT/CEP55/RACGAP1/ESPL1/CENPA/KIF20B
GO:0032465	regulation of cytokinesis	13/205	90/18214	2.4E-11	4.4E-10	3.6E-10	KIF20A/PRC1/CDC6/KIF23/PLK1/CIT/KIF14/RACGAP1/KLHL13/AURKB/PLK4/KIF20B/BRCA2
GO:0000079	regulation of cyclin-dependent protein	13/205	99/18214	8.4E-11	1.4E-09	1.17E-09	CDC6/CCNA2/CCNB1/PLK1/CCNB2/CDKN2C/BLM/PKMYT1/CCNE2/PSRC1/CDKN3/CKS1B/CDC25C
GO:0051656	establishment of organelle localization	24/205	427/18214	9.8E-11	1.7E-09	1.35E-09	CENPF/NUSAP1/SPAG5/CCNB1/KIF2C/PLK1/KIFC1/CENPE/CDCA8/KIF14/NDC80/AURKB/CDT1/CDCA5/DLGAP5/NUF2/FAM83D/ESPL1/KIF22/CE NPA/SAPCD2/KIF18A/MAD2L1/PSRC1
GO:0051302	regulation of cell division	16/205	171/18214	9.9E-11	1.7E-09	1.35E-09	KIF20A/PRC1/CDC6/KIF23/PLK1/CIT/KIF14/RACGAP1/KIF18B/SUSD2/KLHL13/AURKB/PLK4/BLM/KIF20B/BRCA2
GO:0000077	DNA damage checkpoint	15/205	148/18214	1.2E-10	2.1E-09	1.69E-09	CLSPN/DTL/CCNB1/GTSE1/PLK1/CDK1/WDR76/TICRR/FANCD2/BRCA1/BRIP1/BLM/CHEK1/EME1/CDC25C
GO:0032200	telomere organization	15/205	174/18214	1.2E-09	1.9E-08	1.57E-08	H3C2/H3C8/H3C7/AURKB/H4C4/NEK2/EXO1/FEN1/RAD51/H3C12/BLM/H4C9/BRCA2/CCNE2/POLE2
GO:0045814	negative regulation of gene	13/205	124/18214	1.5E-09	2.3E-08	1.86E-08	H3C2/H1-5/H3C8/H2AC11/H3C7/H4C4/CDC45/H2AC13/H2AC12/H3C12/H4C9/H2AC4/H2AC16
GO:0007098	centrosome cycle	13/205	128/18214	2.2E-09	3.3E-08	2.71E-08	PLK1/NDC80/KIF11/CDK1/NEK2/PLK4/BRCA1/XRCC2/PCLAF/BRCA2/CHEK1/SGO1/STIL
GO:1901992	positive regulation of mitotic cell cycle phase transition	11/205	91/18214	6.1E-09	8.8E-08	7.16E-08	DTL/CDC6/CCNB1/UBE2C/CDK1/CDT1/CDCA5/DLGAP5/ESPL1/FBXO5/CDC25C
GO:0040029	regulation of gene expression, epigenetic	15/205	201/18214	8.9E-09	1.3E-07	1.03E-07	H3C2/H1-5/H3C8/H2AC11/H3C7/H4C4/CDC45/H2AC13/H2AC12/BRCA1/H3C12/H4C9/H2AC4/CHEK1/H2AC16
GO:0045931	positive regulation of mitotic cell cycle	12/205	118/18214	9E-09	1.3E-07	1.04E-07	DTL/CDC6/CCNB1/UBE2C/CDK1/CDT1/CDCA5/DLGAP5/ESPL1/BRCA2/FBXO5/CDC25C
GO:0007131	reciprocal meiotic recombination	9/205	57/18214	1.4E-08	1.9E-07	1.56E-07	TOP2A/TRIP13/FANCD2/RAD51/BRIP1/RAD51AP1/RAD54L/EME1/MND1

GO:0031145	anaphase-promoting complex-dependent catabolic process	10/205	83/18214	3.1E-08	4.2E-07	3.42E-07	BUB1B/CCNB1/CDC20/PLK1/UBE2C/CDK1/AURKB/PTTG1/FBXO5/MAD2L1
GO:0040001	establishment of mitotic spindle localization	7/205	33/18214	6.9E-08	9.1E-07	7.44E-07	NUSAP1/PLK1/NDC80/ESPL1/CENPA/SAPCD2/MAD2L1
GO:0000083	regulation of transcription involved in G1/S transition of mitotic cell cycle	7/205	34/18214	8.6E-08	1.1E-06	9.21E-07	CDC6/RRM2/CDT1/CDC45/CDK18/ORC1/FBXO5
GO:0051653	spindle localization	8/205	52/18214	1.1E-07	1.4E-06	1.18E-06	ASPM/NUSAP1/PLK1/NDC80/ESPL1/CENPA/SAPCD2/MAD2L1
GO:0051988	regulation of attachment of spindle microtubules to kinetochore	5/205	13/18214	2.1E-07	2.7E-06	2.16E-06	SPAG5/CCNB1/RACGAP1/AURKB/NEK2
GO:0032467	positive regulation of cytokinesis	7/205	40/18214	2.8E-07	3.5E-06	2.87E-06	CDC6/KIF23/CIT/KIF14/RACGAP1/AURKB/KIF20B
GO:0072331	signal transduction by p53 class mediator	15/205	263/18214	3.1E-07	3.9E-06	3.14E-06	TPX2/FOXM1/CCNB1/GTSE1/CDK1/AURKB/EXO1/E2F2/RMI2/BRCA1/BRIP1/BLM/BRCA2/CHEK1/CDC25C
GO:0070192	chromosome organization involved in meiotic cell cycle	8/205	64/18214	5.8E-07	7.1E-06	5.8E-06	BUB1/BUB1B/TRIP13/FANCD2/RAD51/BRIP1/SGO1/CCNE2
GO:0006890	retrograde vesicle-mediated transport, Golgi to endoplasmic reticulum	9/205	87/18214	5.8E-07	7.1E-06	5.81E-06	KIF4A/KIF23/KIF2C/CENPE/RACGAP1/KIF11/KIF22/KIF18A/KIF15
GO:0006268	DNA unwinding involved in DNA replication	5/205	16/18214	6.8E-07	8.3E-06	6.71E-06	MCM6/MCM2/MCM7/RAD51/BLM
GO:0072401	signal transduction involved in DNA integrity checkpoint	8/205	73/18214	1.6E-06	1.9E-05	1.55E-05	DTL/CCNB1/GTSE1/PLK1/CDK1/BRCA1/CHEK1/CDC25C
GO:0072422	signal transduction involved in DNA damage checkpoint	8/205	73/18214	1.6E-06	1.9E-05	1.55E-05	DTL/CCNB1/GTSE1/PLK1/CDK1/BRCA1/CHEK1/CDC25C
GO:0019886	antigen processing and presentation of exogenous peptide antigen via MHC class II	9/205	99/18214	1.8E-06	2.1E-05	1.67E-05	KIF4A/KIF23/KIF2C/CENPE/RACGAP1/KIF11/KIF22/KIF18A/KIF15
GO:0000723	telomere maintenance	11/205	161/18214	2.1E-06	2.5E-05	2.02E-05	AURKB/H4C4/NEK2/EXO1/FEN1/RAD51/BLM/H4C9/BRCA2/CCNE2/POLE2
GO:0044773	mitotic DNA damage checkpoint	9/205	102/18214	2.3E-06	2.6E-05	2.1E-05	CLSPN/CCNB1/GTSE1/CDK1/TICRR/FANCD2/BLM/EME1/CDC25C
GO:0042770	signal transduction in response to DNA damage	10/205	131/18214	2.3E-06	2.6E-05	2.15E-05	FOXM1/DTL/CCNB1/GTSE1/PLK1/CDK1/BRCA1/BRCA2/CHEK1/CDC25C

GO:0002495	antigen processing and presentation of peptide antigen via MHC class II	9/205	103/18214	2.4E-06	2.8E-05	2.25E-05	KIF4A/KIF23/KIF2C/CENPE/RACGAP1/KIF11/KIF22/KIF18A/KIF15
GO:0002504	antigen processing and presentation of peptide or polysaccharide antigen via MHC class II	9/205	104/18214	2.7E-06	3E-05	2.43E-05	KIF4A/KIF23/KIF2C/CENPE/RACGAP1/KIF11/KIF22/KIF18A/KIF15
GO:0070828	heterochromatin organization	8/205	79/18214	3E-06	3.3E-05	2.67E-05	H3C2/H3C8/H3C7/H4C4/H3C12/H4C9/HELLS/SUV39H1
GO:0006275	regulation of DNA replication	9/205	107/18214	3.4E-06	3.7E-05	3E-05	CDC6/CCNA2/TICRR/CDT1/BLM/BRCA2/ESCO2/FBXO5/DSCC1
GO:0016572	histone phosphorylation	6/205	39/18214	4.5E-06	4.9E-05	3.99E-05	CCNA2/CCNB1/CDK1/AURKB/CHEK1/HASPIN
GO:1904666	regulation of ubiquitin protein ligase activity	5/205	23/18214	4.9E-06	5.3E-05	4.31E-05	CDC20/PLK1/UBE2C/FBXO5/MAD2L1
GO:0007077	mitotic nuclear envelope disassembly	4/205	12/18214	7.2E-06	7.7E-05	6.27E-05	CCNB1/PLK1/CDK1/CCNB2
GO:0001833	inner cell mass cell proliferation	4/205	13/18214	1E-05	0.00011	8.87E-05	NCAPG2/BRCA2/GINS1/GINS4
GO:0051984	positive regulation of chromosome segregation	4/205	13/18214	1E-05	0.00011	8.87E-05	CDC6/CCNB1/AURKB/CDT1
GO:0051052	regulation of DNA metabolic process	15/205	351/18214	1.1E-05	0.00012	9.38E-05	FOXM1/H1-5/POLQ/AURKB/TICRR/NEK2/CDT1/RMI2/BRCA1/RAD51/BLM/CHEK1/RAD51AP1/DSCC1/PARPBP
GO:0007018	microtubule-based movement	15/205	354/18214	1.2E-05	0.00013	0.000102	KIF20A/KIF4A/KIF23/KIF2C/KIFC1/CENPE/KIF14/RACGAP1/KIF18B/KIF11/DLGAP5/KIF22/KIF20B/KIF18A/KIF15
GO:0051445	regulation of meiotic cell cycle	6/205	46/18214	1.2E-05	0.00013	0.000102	CDC20/TTK/TRIP13/RAD51AP1/FBXO5/CDC25C
GO:0009314	response to radiation	17/205	445/18214	1.2E-05	0.00013	0.000102	DTL/PBK/AURKB/TICRR/FANCD2/FEN1/BRCA1/XRCC2/RAD51/BLM/PCLAF/BRCA2/FBXL22/CHEK1/RAD51AP1/FANCG/RAD54L
GO:0010569	regulation of double-strand break repair via homologous recombination	6/205	47/18214	1.4E-05	0.00014	0.000113	POLQ/RMI2/RAD51/CHEK1/RAD51AP1/PARPBP
GO:0007050	cell cycle arrest	12/205	234/18214	1.4E-05	0.00014	0.000116	FOXM1/PRR11/CCNB1/GTSE1/CDK1/CDKN2C/BRCA1/KIF20B/GPER1/CDKN3/DDIAS/CDC25C
GO:0072425	signal transduction involved in G2 DNA damage checkpoint	4/205	14/18214	1.4E-05	0.00014	0.000116	DTL/PLK1/BRCA1/CHEK1
GO:0030397	membrane disassembly	4/205	15/18214	1.9E-05	0.00019	0.000152	CCNB1/PLK1/CDK1/CCNB2
GO:0051081	nuclear envelope disassembly	4/205	15/18214	1.9E-05	0.00019	0.000152	CCNB1/PLK1/CDK1/CCNB2
GO:0060968	regulation of gene silencing	9/205	142/18214	3.3E-05	0.00031	0.000254	H3C2/H1-5/H3C8/H3C7/RMRP/H4C4/CDC45/H3C12/H4C9
GO:2001020	regulation of response to DNA damage stimulus	11/205	221/18214	4.3E-05	0.0004	0.000323	FOXM1/POLQ/WDR76/RMI2/BRCA1/RAD51/CHEK1/RAD51AP1/FBXO5/DDIAS/PARPBP

GO:0031109	microtubule polymerization or depolymerization	8/205	114/18214	4.5E-05	0.00041	0.000334	TPX2/KIF2C/KIF18B/SKA1/SKA3/KIF18A/FBXO5/PSRC1
GO:0051781	positive regulation of cell division	7/205	86/18214	5.2E-05	0.00048	0.000388	CDC6/KIF23/CIT/KIF14/RACGAP1/AURKB/KIF20B
GO:0071897	DNA biosynthetic process	9/205	189/18214	0.0003	0.00241	0.001961	CENPF/DTL/TK1/POLQ/AURKB/NEK2/PCLAF/DSCC1/POLE2
GO:0038111	interleukin-7-mediated signaling pathway	4/205	30/18214	0.00034	0.00272	0.002211	H3C2/H3C8/H3C7/H3C12
GO:0040020	regulation of meiotic nuclear division	4/205	30/18214	0.00034	0.00272	0.002211	CDC20/TRIP13/RAD51AP1/FBXO5
GO:0048002	antigen processing and presentation of peptide antigen	9/205	193/18214	0.00035	0.00275	0.002235	KIF4A/KIF23/KIF2C/CENPE/RACGAP1/KIF11/KIF22/KIF18A/KIF15
GO:0072698	protein localization to microtubule cytoskeleton	5/205	54/18214	0.00035	0.00275	0.002235	SPAG5/TTK/FAM83D/KIF20B/STIL
GO:1904668	positive regulation of ubiquitin protein ligase activity	3/205	13/18214	0.00037	0.00291	0.002367	CDC20/PLK1/UBE2C
GO:0051785	positive regulation of nuclear division	5/205	56/18214	0.00041	0.00322	0.002617	NUSAP1/CDT1/DLGAP5/ESPL1/RAD51AP1
GO:0007100	mitotic centrosome separation	3/205	14/18214	0.00047	0.00358	0.002913	KIF11/NEK2/CHEK1
GO:0045842	positive regulation of mitotic metaphase/anaphase transition	3/205	14/18214	0.00047	0.00358	0.002913	CDT1/DLGAP5/ESPL1
GO:0008156	negative regulation of DNA replication	4/205	34/18214	0.00055	0.00417	0.003391	CDC6/BLM/BRCA2/FBXO5
GO:0001824	blastocyst development	6/205	92/18214	0.0006	0.00448	0.003642	NEK2/NCAPG2/ASF1B/BRCA2/GINS1/GINS4
GO:0007096	regulation of exit from mitosis	3/205	16/18214	0.00071	0.00522	0.004247	ANLN/UBE2C/CDCA5
GO:0051447	negative regulation of meiotic cell cycle	3/205	17/18214	0.00085	0.00616	0.005014	TTK/TRIP13/FBXO5
GO:0051782	negative regulation of cell division	3/205	17/18214	0.00085	0.00616	0.005014	SUSD2/AURKB/BLM
GO:1901796	regulation of signal transduction by p53 class mediator	8/205	177/18214	0.0009	0.00652	0.005303	TPX2/AURKB/EXO1/RMI2/BRCA1/BRIP1/BLM/CHEK1
GO:0070507	regulation of microtubule cytoskeleton organization	7/205	137/18214	0.00093	0.00665	0.005412	TPX2/SPAG5/PLK1/SKA1/SKA3/KIF18A/PSRC1
GO:0090224	regulation of spindle organization	4/205	39/18214	0.00094	0.00672	0.005465	TPX2/SPAG5/PLK1/PSRC1

GO:0098760	response to interleukin-7	4/205	40/18214	0.00104	0.00729	0.005927	H3C2/H3C8/H3C7/H3C12
GO:0098761	cellular response to interleukin-7	4/205	40/18214	0.00104	0.00729	0.005927	H3C2/H3C8/H3C7/H3C12
GO:0007019	microtubule depolymerization	4/205	44/18214	0.00149	0.0101	0.008217	TPX2/KIF2C/KIF18B/KIF18A
GO:0046599	regulation of centriole replication	3/205	21/18214	0.00161	0.01089	0.008856	PLK4/BRCA1/STIL
GO:0061647	histone H3-K9 modification	4/205	45/18214	0.00162	0.01091	0.008874	H1-5/BRCA1/CHEK1/SUV39H1
GO:0048146	positive regulation of fibroblast proliferation	4/205	46/18214	0.00176	0.01172	0.00953	CDC6/CCNA2/CCNB1/AGT
GO:0070316	regulation of G0 to G1 transition	4/205	46/18214	0.00176	0.01172	0.00953	RRM2/BRCA1/RAD51/CHEK1
GO:0045023	G0 to G1 transition	4/205	47/18214	0.0019	0.01256	0.010214	RRM2/BRCA1/RAD51/CHEK1
GO:0018107	peptidyl-threonine phosphorylation	6/205	117/18214	0.0021	0.0138	0.011221	PLK1/CIT/CDK1/TTK/CHEK1/HASPIN
GO:0043462	regulation of ATPase activity	5/205	81/18214	0.0022	0.01439	0.011704	MCM2/NCAPH/UHRF1/TNNT3/TNNI3
GO:0060964	regulation of gene silencing by miRNA	6/205	120/18214	0.00238	0.0154	0.012522	H3C2/H3C8/H3C7/H4C4/H3C12/H4C9
GO:2001252	positive regulation of chromosome organization	7/205	164/18214	0.0026	0.0167	0.013586	H1-5/CCNB1/AURKB/NEK2/CDT1/FEN1/BRCA1
GO:0018210	peptidyl-threonine modification	6/205	126/18214	0.00304	0.01924	0.015645	PLK1/CIT/CDK1/TTK/CHEK1/HASPIN
GO:0006977	DNA damage response, signal transduction by p53 class mediator resulting in cell cycle arrest	4/205	56/18214	0.00362	0.02261	0.018386	CCNB1/GTSE1/CDK1/CDC25C
GO:1902400	intracellular signal transduction involved in G1 DNA damage checkpoint	4/205	57/18214	0.00386	0.02386	0.019405	CCNB1/GTSE1/CDK1/CDC25C
GO:1902402	signal transduction involved in mitotic DNA damage checkpoint	4/205	59/18214	0.00437	0.02684	0.021825	CCNB1/GTSE1/CDK1/CDC25C
GO:0030010	establishment of cell polarity	6/205	136/18214	0.00443	0.02706	0.022009	PLK1/NDC80/CENPA/KIF20B/SAPCD2/MAD2L1
GO:0016569	covalent chromatin modification	12/205	461/18214	0.00615	0.03672	0.029864	H1-5/CCNA2/CCNB1/CDK1/UHRF1/AURKB/BRCA1/BRCA2/HELLS/CHEK1/HASPIN/SUV39H1
GO:2000615	regulation of histone H3-K9 acetylation	2/205	11/18214	0.00649	0.03799	0.030893	BRCA1/CHEK1
GO:0051567	histone H3-K9 methylation	3/205	34/18214	0.0065	0.03799	0.030893	H1-5/BRCA1/SUV39H1
